# Supplementary material for: Accurate multi-population imputation of MICA, MICB, HLA-E, HLA-F and HLA-G alleles from genome SNP data
Source: PLoS Comput Biol. 2024 Sep 16;20(9):e1011718. doi: 10.1371/journal.pcbi.1011718 (PMC11426482; doi:10.1371/journal.pcbi.1011718)
Supplement: S1 Text — (PDF) [file pcbi.1011718.s001.pdf]

Supplementary Tables and Figures

Accurate multi-population imputation of *MICA*, *MICB*, *HLA-E*, *HLA-F* and *HLA-G* alleles from genome SNP data

Silja Tammi[1], Satu Koskela[1,2], Blood Service Biobank[2], Kati Hyvärinen[1], Jukka Partanen[1,2], Jarmo Ritari[1]

[1]Finnish Red Cross Blood Service, Research and Development, Helsinki, Finland

[2] Finnish Red Cross Blood Service, Blood Service Biobank, Vantaa, Finland

Supplementary Tables:

Table A. Alleles and their frequencies in the Finnish reference

Table B. Alleles and their frequencies in the 1000 Genomes reference

Table C. Sensitivity, specificity, positive predictive value (PPV) and negative predictive value (NPV) for MICA, MICB, HLA-E and HLA-F alleles in the combined Finnish and 1000G reference (model VI)

Table D. Sensitivity, specificity, positive predictive value (PPV) and negative predictive value (NPV) for HLA-G alleles and HLA-G 3'UTR and 5' UTR haplotypes in the Finnish reference (model I)

Supplementary Figures:

Fig A. Effect of the flanking region size on out-of bag (OOB) and test accuracy

Fig B. Model properties of the 1000G/FIN models

Fig C. Overall imputation accuracies in the Finnish and the 1000 Genomes superpopulations when omitting the untrained alleles, i.e. alleles not present in the training data

Fig D. Difference in percentage point in the overall imputation accuracy when omitting untrained alleles (i.e. alleles not present in the training set) versus without omitting (i.e. all alleles present in the test set)

Fig E. Pearson's correlation (r) of imputed and true allele dosages versus allele frequency

Fig F. Cross-validation imputation accuracies and properties of the models fitted for GSA and PMRA SNP content

Table A. Alleles and their frequencies in the Finnish reference

MICA	Count	Freq	MICB	Count	Freq	HLA-E	Count	Freq	HLA-F	Count	Freq	HLA-G	Count	Freq	HLA-G 3'UTR	Count	Freq	HLA-G 5'UTR	Count	Freq
008:01/04[a]	660	0.434	004:01	470	0.309	01:01	424	0.481	01:01	311	0.737	01:01:01:01/02	305	0.351	UTR-1	308	0.354	010101a	312	0.359
002:01	249	0.164	005:02	463	0.304	01:03	368	0.417	01:03	107	0.254	01:01:01:05	212	0.244	UTR-4	212	0.244	010102a	245	0.282
010:01	195	0.128	002:01	257	0.169	01:06	89	0.101	01:04	4	0.009	01:01:02:01	151	0.174	UTR-2	210	0.241	010101b	143	0.164
007:01	125	0.082	008:01	141	0.093	01:11	1	0.001	Total	422	1.000	01:04:01:01	71	0.082	UTR-3	77	0.089	010104a	75	0.086
009:01	84	0.055	005:03	52	0.034	Total	882	1.000				01:01:03:03	37	0.043	UTR-7	37	0.043	010101c	69	0.079
018:01	59	0.039	014:01	39	0.026							01:06:01:01	32	0.037	UTR-5	14	0.016	0103a	12	0.014
027:01	38	0.025	039	33	0.022							01:01:14	20	0.023	UTR-18	6	0.007	010101f	7	0.008
017	27	0.018	003:01	29	0.019							01:03:01:02	14	0.016	UTR-6	5	0.006	010104b	3	0.003
004:01	22	0.014	013:01	13	0.009							01:01:22	8	0.009	other	1	0.001	other	3	0.003
049:01	18	0.012	028	13	0.009							01:01:01:04	5	0.006	Total	870	1.000	0103e	1	0.001
012:03	16	0.011	005:01	3	0.002							01:04:04	4	0.005				Total	870	1.000
012:01	12	0.008	005:08	3	0.002							01:01:01:09	2	0.002						
016	5	0.003	005:06	2	0.001							01:21N	2	0.002						
011:01	3	0.002	006	1	0.001							01:01:01:12	1	0.001						
001	2	0.001	009:01N	1	0.001							01:01:01:13	1	0.001						
009:02	2	0.001	053	1	0.001							01:01:01:g.173G>A	1	0.001						
006	1	0.001	052	1	0.001							01:01:01:g.188C>T	1	0.001						
019:01	1	0.001	Total	1,522	1.003							01:01:01:g.636C>T	1	0.001						
052	1	0.001										01:05N	1	0.001						
193	1	0.001										01:L-14P	1	0.001						
285	1	0.001										Total	870	1.001						
Total	1,522	1.002																		

[a] Ambiguous result due to incomplete sequence data

Table B. Alleles and their frequencies in the 1000 Genomes reference

MICA												
	ALL		EUR		AFR		EAS		SAS		AMR	
Allele	Count	Freq	Count	Freq	Count	Freq	Count	Freq	Count	Freq	Count	Freq
008:01/04 [a]	699	0.225	248	0.379	171	0.193	75	0.168	123	0.194	82	0.167
002:01	634	0.204	87	0.133	258	0.291	72	0.161	91	0.144	126	0.257
004:01	380	0.122	63	0.096	192	0.217	20	0.045	47	0.074	58	0.118
009:01	209	0.067	46	0.070	7	0.008	31	0.070	99	0.156	26	0.053
010:01	189	0.061	25	0.038	2	0.002	82	0.184	35	0.055	45	0.092
027:01	136	0.044	6	0.009	2	0.002	35	0.078	54	0.085	39	0.080
019:01	105	0.034	12	0.018	4	0.005	37	0.083	46	0.073	6	0.012
017	103	0.033	27	0.041	NA	NA	6	0.013	60	0.095	10	0.020
018:01	76	0.024	19	0.029	32	0.036	1	0.002	12	0.019	12	0.024
012:01	72	0.023	16	0.024	18	0.020	25	0.056	11	0.017	2	0.004
009:02	54	0.017	12	0.018	18	0.020	2	0.004	10	0.016	12	0.024
007:01	50	0.016	31	0.047	3	0.003	1	0.002	7	0.011	8	0.016
016	49	0.016	18	0.028	NA	NA	NA	NA	12	0.019	19	0.039
015:01	48	0.015	2	0.003	43	0.049	NA	NA	NA	NA	3	0.006
008:02	40	0.013	2	0.003	18	0.020	11	0.025	6	0.009	3	0.006
001	38	0.012	10	0.015	18	0.020	NA	NA	NA	NA	10	0.020
049:01	35	0.011	11	0.017	NA	NA	15	0.034	8	0.013	1	0.002
011:01	34	0.011	7	0.011	8	0.009	1	0.002	NA	NA	18	0.037
045:01	34	0.011	NA	NA	7	0.008	17	0.038	10	0.016	NA	NA
041	26	0.008	NA	NA	26	0.029	NA	NA	NA	NA	NA	NA
008:13	23	0.007	NA	NA	22	0.025	1	0.002	NA	NA	NA	NA
110	18	0.006	NA	NA	17	0.019	NA	NA	NA	NA	1	0.002
006	7	0.002	3	0.005	NA	NA	NA	NA	3	0.005	1	0.002
012:03	6	0.002	4	0.006	NA	NA	NA	NA	NA	NA	2	0.004
030	6	0.002	NA	NA	6	0.007	NA	NA	NA	NA	NA	NA
043	4	0.001	NA	NA	2	0.002	NA	NA	NA	NA	2	0.004
028	4	0.001	NA	NA	1	0.001	2	0.004	NA	NA	1	0.002
035	4	0.001	NA	NA	1	0.001	2	0.004	NA	NA	1	0.002
068:01	3	0.001	2	0.003	1	0.001	NA	NA	NA	NA	NA	NA
024	3	0.001	NA	NA	NA	NA	3	0.007	NA	NA	NA	NA
076	3	0.001	NA	NA	NA	NA	3	0.007	NA	NA	NA	NA
141	2	0.001	1	0.002	1	0.001	NA	NA	NA	NA	NA	NA
185	2	0.001	1	0.002	NA	NA	NA	NA	NA	NA	1	0.002
046	2	0.001	NA	NA	2	0.002	NA	NA	NA	NA	NA	NA
056	2	0.001	NA	NA	2	0.002	NA	NA	NA	NA	NA	NA
029:01	2	0.001	NA	NA	1	0.001	1	0.002	NA	NA	NA	NA
033	2	0.001	NA	NA	NA	NA	2	0.004	NA	NA	NA	NA
119:01	1	0.000	1	0.002	NA	NA	NA	NA	NA	NA	NA	NA
057:01	1	0.000	NA	NA	1	0.001	NA	NA	NA	NA	NA	NA
151:01	1	0.000	NA	NA	1	0.001	NA	NA	NA	NA	NA	NA
158	1	0.000	NA	NA	1	0.001	NA	NA	NA	NA	NA	NA
008:10	1	0.000	NA	NA	NA	NA	1	0.002	NA	NA	NA	NA
052	1	0.000	NA	NA	NA	NA	NA	NA	NA	NA	1	0.002
Total	3110	0.998	654	0.999	886	0.997	446	0.997	634	1.001	490	0.997

MICB

	ALL		EUR		AFR		EAS		SAS		AMR	
Allele	Count	Freq	Count	Freq	Count	Freq	Count	Freq	Count	Freq	Count	Freq
005:02	1,383	0.431	272	0.406	384	0.410	242	0.506	198	0.320	287	0.563
002:01	627	0.195	107	0.160	248	0.265	74	0.155	118	0.191	80	0.157
004:01	441	0.137	139	0.207	117	0.125	42	0.088	82	0.133	61	0.120
008:01	252	0.078	63	0.094	107	0.114	37	0.077	23	0.037	22	0.043
005:03	169	0.053	14	0.021	1	0.001	38	0.079	107	0.173	9	0.018
003:01	142	0.044	33	0.049	25	0.027	7	0.015	66	0.107	11	0.022
014:01	54	0.017	9	0.013	15	0.016	25	0.052	4	0.006	1	0.002
005:06	34	0.011	7	0.010	11	0.012	3	0.006	8	0.013	5	0.010
005:01	24	0.007	9	0.013	1	0.001	NA	NA	3	0.005	11	0.022
009:01N	20	0.006	NA	NA	1	0.001	6	0.013	NA	NA	13	0.025
024:01	17	0.005	4	0.006	12	0.013	NA	NA	NA	NA	1	0.002
005:08	14	0.004	5	0.007	NA	NA	NA	NA	7	0.011	2	0.004
013:01	10	0.003	4	0.006	NA	NA	2	0.004	1	0.002	3	0.006
028	9	0.003	4	0.006	2	0.002	NA	NA	1	0.002	2	0.004
006	7	0.002	NA	NA	7	0.007	NA	NA	NA	NA	NA	NA
033	6	0.002	NA	NA	5	0.005	NA	NA	NA	NA	1	0.002
019:01	1	0.000	NA	NA	NA	NA	1	0.002	NA	NA	NA	NA
029	1	0.000	NA	NA	NA	NA	1	0.002	NA	NA	NA	NA
021:01N	1	0.000	NA	NA	NA	NA	NA	NA	NA	NA	1	0.002
Total	3,212	0.998	670	0.998	936	0.999	478	0.999	618	1	510	1.002

HLA-E

	ALL		EUR		AFR		EAS		SAS		AMR	
Allele	Count	Freq	Count	Freq	Count	Freq	Count	Freq	Count	Freq	Count	Freq
01:01	2,044	0.502	543	0.568	545	0.563	315	0.337	338	0.528	303	0.526
01:03	1,950	0.479	372	0.389	404	0.417	606	0.649	299	0.467	269	0.467
01:06	40	0.010	33	0.035	NA	NA	1	0.001	3	0.005	3	0.005
01:05	17	0.004	3	0.003	14	0.014	NA	NA	NA	NA	NA	NA
01:12	11	0.003	NA	NA	NA	NA	11	0.012	NA	NA	NA	NA
01:09	4	0.001	2	0.002	1	0.001	NA	NA	NA	NA	1	0.002
01:11	3	0.001	3	0.003	NA	NA	NA	NA	NA	NA	NA	NA
01:13	3	0.001	NA	NA	3	0.003	NA	NA	NA	NA	NA	NA
01:77	1	0.000	NA	NA	1	0.001	NA	NA	NA	NA	NA	NA
01:10	1	0.000	NA	NA	NA	NA	1	0.001	NA	NA	NA	NA
Total	4,074	1.001	956	1	968	0.999	934	1	640	1	576	1

HLA-F

	ALL		EUR		AFR		EAS		SAS		AMR	
Allele	Count	Freq	Count	Freq	Count	Freq	Count	Freq	Count	Freq	Count	Freq
01:01	2,227	0.861	480	0.811	601	0.801	332	0.971	533	0.916	281	0.878
01:03	337	0.130	105	0.177	141	0.188	5	0.015	48	0.082	38	0.119
01:02	9	0.003	NA	NA	8	0.011	NA	NA	NA	NA	1	0.003
01:04	8	0.003	7	0.012	NA	NA	NA	NA	1	0.002	NA	NA
01:05	5	0.002	NA	NA	NA	NA	5	0.015	NA	NA	NA	NA
Total	2,586	0.999	592	1	750	1	342	1.001	582	1	320	1

[a] Ambiguous result due to incomplete sequence data

EUR, European; EAS, East Asian; SAS, South Asian; AMR, mixed American

Table C. Sensitivity, specificity, positive predictive value (PPV) and negative predictive value (NPV) for MICA, MICB, HLA-E and HLA-F alleles in the combined Finnish and 1000G reference (model VI).

MICA

	allele	train.num	train.freq	valid.num	valid.freq	call.rate	accuracy	sensitivity	specificity	ppv	npv	miscall	miscall.prop
EUR	008:01/04	903	0.289	80	0.370	1	1.000	1.000	1.000	1.000	1.000		
	002:01	579	0.185	29	0.134	1	1.000	1.000	1.000	1.000	1.000		
	004:01	278	0.089	20	0.093	1	0.991	0.950	0.995	0.950	0.995	019:01	1
	010:01	264	0.084	8	0.037	1	1.000	1.000	1.000	1.000	1.000		
	009:01	203	0.065	18	0.083	1	0.995	0.944	1.000	1.000	0.995	004:01	1
	007:01	120	0.038	11	0.051	1	1.000	1.000	1.000	1.000	1.000		
	027:01	118	0.038	1	0.005	1	1.000	1.000	1.000	1.000	1.000		
	018:01	93	0.030	9	0.042	1	1.000	1.000	1.000	1.000	1.000		
	017	87	0.028	5	0.023	1	0.995	1.000	0.995	0.833	1.000		
	019:01	71	0.023	5	0.023	1	0.995	1.000	0.995	0.833	1.000		
	012:01	57	0.018	5	0.023	1	0.995	0.800	1.000	1.000	0.995	012:03	1
	009:02	37	0.012	4	0.019	1	1.000	1.000	1.000	1.000	1.000		
	016	37	0.012	7	0.032	1	1.000	1.000	1.000	1.000	1.000		
	049:01	36	0.012	6	0.028	1	1.000	1.000	1.000	1.000	1.000		
	015:01	33	0.011	1	0.005	1	1.000	1.000	1.000	1.000	1.000		
	001	28	0.009	2	0.009	1	1.000	1.000	1.000	1.000	1.000		
	011:01	25	0.008	2	0.009	1	1.000	1.000	1.000	1.000	1.000		
	012:03	16	0.005	1	0.005	1	0.995	1.000	0.995	0.500	1.000		
	006	6	0.002	1	0.005	1	1.000	1.000	1.000	1.000	1.000		
	068:01	2	0.001	1	0.005	1	0.995	0.000	1.000		0.995	017	1

	allele	train.num	train.freq	valid.num	valid.freq	call.rate	accuracy	sensitivity	specificity	ppv	npv	miscall	miscall.prop
AFR	008:01/04	903	0.289	52	0.202	1	0.996	1.000	0.995	0.981	1.000		
	002:01	579	0.185	96	0.372	1	0.996	0.990	1.000	1.000	0.994	015:01	1
	004:01	278	0.089	45	0.174	1	1.000	1.000	1.000	1.000	1.000		
	009:01	203	0.065	1	0.004	1	1.000	1.000	1.000	1.000	1.000		
	018:01	93	0.030	7	0.027	1	1.000	1.000	1.000	1.000	1.000		
	012:01	57	0.018	3	0.012	1	1.000	1.000	1.000	1.000	1.000		

	009:02	37	0.012	6	0.023	1	1.000	1.000	1.000	1.000	1.000		
	015:01	33	0.011	13	0.050	1	0.996	1.000	0.996	0.929	1.000		
	001	28	0.009	5	0.019	1	1.000	1.000	1.000	1.000	1.000		
	008:02	25	0.008	5	0.019	1	0.984	0.200	1.000	1.000	0.984	008:13	1
	011:01	25	0.008	1	0.004	1	1.000	1.000	1.000	1.000	1.000		
	045:01	24	0.008	2	0.008	1	1.000	1.000	1.000	1.000	1.000		
	041	18	0.006	8	0.031	1	1.000	1.000	1.000	1.000	1.000		
	008:13	16	0.005	6	0.023	1	0.981	0.833	0.984	0.556	0.996	008:01/04	1
	110	12	0.004	6	0.023	1	1.000	1.000	1.000	1.000	1.000		
	030	4	0.001	2	0.008	1	1.000	1.000	1.000	1.000	1.000		

	allele	train.num	train.freq	valid.num	valid.freq	call.rate	accuracy	sensitivity	specificity	ppv	npv	miscall	miscall.prop
EAS	008:01/04	903	0.289	29	0.186	1	0.994	1.000	0.992	0.967	1.000		
	002:01	579	0.185	31	0.199	1	0.994	1.000	0.992	0.969	1.000		
	004:01	278	0.089	12	0.077	1	1.000	1.000	1.000	1.000	1.000		
	010:01	264	0.084	20	0.128	1	1.000	1.000	1.000	1.000	1.000		
	009:01	203	0.065	4	0.026	1	1.000	1.000	1.000	1.000	1.000		
	027:01	118	0.038	15	0.096	1	0.994	1.000	0.993	0.938	1.000		
	018:01	93	0.030	1	0.006	1	1.000	1.000	1.000	1.000	1.000		
	017	87	0.028	4	0.026	1	1.000	1.000	1.000	1.000	1.000		
	019:01	71	0.023	13	0.083	1	1.000	1.000	1.000	1.000	1.000		
	012:01	57	0.018	9	0.058	1	1.000	1.000	1.000	1.000	1.000		
	049:01	36	0.012	3	0.019	1	0.994	1.000	0.993	0.750	1.000		
	008:02	25	0.008	6	0.038	1	1.000	1.000	1.000	1.000	1.000		
	045:01	24	0.008	4	0.026	1	1.000	1.000	1.000	1.000	1.000		
	008:13	16	0.005	1	0.006	1	1.000	1.000	1.000	1.000	1.000		
	028	3	0.001	1	0.006	1	0.994	0.000	1.000		0.994	002:01	0.5
	035	3	0.001	1	0.006	1	0.994	0.000	1.000		0.994	002:01	0.5
	024	2	0.001	1	0.006	1	0.994	0.000	1.000		0.994	027:01	0.5
	076	2	0.001	1	0.006	1	0.994	0.000	1.000		0.994	027:01	0.5

	allele	train.num	train.freq	valid.num	valid.freq	call.rate	accuracy	sensitivity	specificity	ppv	npv	miscall	miscall.prop
SAS	008:01/04	903	0.289	49	0.225	1	1.000	1.000	1.000	1.000	1.000		

	002:01	579	0.185	24	0.110	1	1.000	1.000	1.000	1.000	1.000		
	004:01	278	0.089	16	0.073	1	1.000	1.000	1.000	1.000	1.000		
	010:01	264	0.084	12	0.055	1	0.995	0.917	1.000	1.000	0.995	027:01	1
	009:01	203	0.065	34	0.156	1	1.000	1.000	1.000	1.000	1.000		
	007:01	120	0.038	3	0.014	1	1.000	1.000	1.000	1.000	1.000		
	027:01	118	0.038	19	0.087	1	0.991	1.000	0.990	0.905	1.000		
	018:01	93	0.030	4	0.018	1	1.000	1.000	1.000	1.000	1.000		
	017	87	0.028	19	0.087	1	1.000	1.000	1.000	1.000	1.000		
	019:01	71	0.023	16	0.073	1	0.995	0.938	1.000	1.000	0.995	027:01	1
	012:01	57	0.018	5	0.023	1	1.000	1.000	1.000	1.000	1.000		
	009:02	37	0.012	4	0.018	1	1.000	1.000	1.000	1.000	1.000		
	016	37	0.012	4	0.018	1	1.000	1.000	1.000	1.000	1.000		
	049:01	36	0.012	2	0.009	1	1.000	1.000	1.000	1.000	1.000		
	008:02	25	0.008	2	0.009	1	1.000	1.000	1.000	1.000	1.000		
	045:01	24	0.008	4	0.018	1	1.000	1.000	1.000	1.000	1.000		
	006	6	0.002	1	0.005	1	1.000	1.000	1.000	1.000	1.000		

	allele	train.num	train.freq	valid.num	valid.freq	call.rate	accuracy	sensitivity	specificity	ppv	npv	miscall	miscall.prop
AMR	008:01/04	903	0.289	21	0.131	1	1.000	1.000	1.000	1.000	1.000		
	002:01	579	0.185	42	0.263	1	1.000	1.000	1.000	1.000	1.000		
	004:01	278	0.089	25	0.156	1	0.994	0.960	1.000	1.000	0.993	009:02	1
	010:01	264	0.084	17	0.106	1	1.000	1.000	1.000	1.000	1.000		
	009:01	203	0.065	6	0.038	1	1.000	1.000	1.000	1.000	1.000		
	007:01	120	0.038	4	0.025	1	1.000	1.000	1.000	1.000	1.000		
	027:01	118	0.038	10	0.063	1	1.000	1.000	1.000	1.000	1.000		
	018:01	93	0.030	1	0.006	1	1.000	1.000	1.000	1.000	1.000		
	017	87	0.028	6	0.038	1	1.000	1.000	1.000	1.000	1.000		
	019:01	71	0.023	1	0.006	1	1.000	1.000	1.000	1.000	1.000		
	012:01	57	0.018	1	0.006	1	1.000	1.000	1.000	1.000	1.000		
	009:02	37	0.012	4	0.025	1	0.994	1.000	0.994	0.800	1.000		
	016	37	0.012	5	0.031	1	1.000	1.000	1.000	1.000	1.000		
	015:01	33	0.011	1	0.006	1	1.000	1.000	1.000	1.000	1.000		

	001	28	0.009	4	0.025	1	1.000	1.000	1.000	1.000	1.000		
	008:02	25	0.008	2	0.013	1	1.000	1.000	1.000	1.000	1.000		
	011:01	25	0.008	8	0.050	1	1.000	1.000	1.000	1.000	1.000		
	012:03	16	0.005	1	0.006	1	1.000	1.000	1.000	1.000	1.000		
	043	3	0.001	1	0.006	1	1.000	1.000	1.000	1.000	1.000		

	allele	train.num	train.freq	valid.num	valid.freq	call.rate	accuracy	sensitivity	specificity	ppv	npv	miscall	miscall.prop
FIN	008:01/04	903	0.289	225	0.452	1	1.000	1.000	1.000	1.000	1.000		
	002:01	579	0.185	82	0.165	1	1.000	1.000	1.000	1.000	1.000		
	004:01	278	0.089	6	0.012	1	1.000	1.000	1.000	1.000	1.000		
	010:01	264	0.084	63	0.127	1	1.000	1.000	1.000	1.000	1.000		
	009:01	203	0.065	27	0.054	1	1.000	1.000	1.000	1.000	1.000		
	007:01	120	0.038	37	0.074	1	1.000	1.000	1.000	1.000	1.000		
	027:01	118	0.038	11	0.022	1	1.000	1.000	1.000	1.000	1.000		
	018:01	93	0.030	20	0.040	1	1.000	1.000	1.000	1.000	1.000		
	017	87	0.028	9	0.018	1	1.000	1.000	1.000	1.000	1.000		
	012:01	57	0.018	4	0.008	1	1.000	1.000	1.000	1.000	1.000		
	009:02	37	0.012	1	0.002	1	1.000	1.000	1.000	1.000	1.000		
	016	37	0.012	1	0.002	1	1.000	1.000	1.000	1.000	1.000		
	049:01	36	0.012	6	0.012	1	1.000	1.000	1.000	1.000	1.000		
	001	28	0.009	1	0.002	1	1.000	1.000	1.000	1.000	1.000		
	011:01	25	0.008	1	0.002	1	1.000	1.000	1.000	1.000	1.000		
	012:03	16	0.005	4	0.008	1	1.000	1.000	1.000	1.000	1.000		

MICB

	allele	train.num	train.freq	valid.num	valid.freq	call.rate	accuracy	sensitivity	specificity	ppv	npv	miscall	miscall.prop
EUR	005:02	1232	0.388	95	0.432	1	0.995	1.000	0.992	0.990	1.000		
	004:01	613	0.193	39	0.177	1	0.995	1.000	0.994	0.975	1.000		
	002:01	594	0.187	38	0.173	1	1.000	1.000	1.000	1.000	1.000		
	008:01	265	0.083	18	0.082	1	1.000	1.000	1.000	1.000	1.000		
	005:03	145	0.046	4	0.018	1	1.000	1.000	1.000	1.000	1.000		
	003:01	116	0.037	12	0.055	1	1.000	1.000	1.000	1.000	1.000		
	014:01	62	0.020	2	0.009	1	0.995	0.500	1.000	1.000	0.995	005:02	1

	005:06	25	0.008	3	0.014	1	1.000	1.000	1.000	1.000	1.000		
	005:01	18	0.006	4	0.018	1	1.000	1.000	1.000	1.000	1.000		
	028	15	0.005	2	0.009	1	1.000	1.000	1.000	1.000	1.000		
	005:08	12	0.004	2	0.009	1	1.000	1.000	1.000	1.000	1.000		
	024:01	12	0.004	1	0.005	1	0.995	0.000	1.000		0.995	004:01	1

	allele	train.num	train.freq	valid.num	valid.freq	call.rate	accuracy	sensitivity	specificity	ppv	npv	miscall	miscall.prop
AFR	005:02	1232	0.388	120	0.405	1	0.993	1.000	0.989	0.984	1.000		
	004:01	613	0.193	38	0.128	1	0.986	1.000	0.984	0.905	1.000		
	002:01	594	0.187	77	0.260	1	1.000	1.000	1.000	1.000	1.000		
	008:01	265	0.083	35	0.118	1	1.000	1.000	1.000	1.000	1.000		
	005:03	145	0.046	1	0.003	1	1.000	1.000	1.000	1.000	1.000		
	003:01	116	0.037	7	0.024	1	1.000	1.000	1.000	1.000	1.000		
	014:01	62	0.020	8	0.027	1	1.000	1.000	1.000	1.000	1.000		
	005:06	25	0.008	2	0.007	1	1.000	1.000	1.000	1.000	1.000		
	024:01	12	0.004	4	0.014	1	0.986	0.000	1.000		0.986	004:01	1
	006	6	0.002	2	0.007	1	0.993	0.000	1.000		0.993	005:02	1
	033	4	0.001	2	0.007	1	1.000	1.000	1.000	1.000	1.000		

	allele	train.num	train.freq	valid.num	valid.freq	call.rate	accuracy	sensitivity	specificity	ppv	npv	miscall	miscall.prop
EAS	005:02	1232	0.388	74	0.474	1	1.000	1.000	1.000	1.000	1.000		
	004:01	613	0.193	18	0.115	1	1.000	1.000	1.000	1.000	1.000		
	002:01	594	0.187	24	0.154	1	0.987	0.917	1.000	1.000	0.985	005:03	0.5
	008:01	265	0.083	15	0.096	1	1.000	1.000	1.000	1.000	1.000		
	005:03	145	0.046	11	0.071	1	0.987	1.000	0.986	0.846	1.000		
	003:01	116	0.037	3	0.019	1	1.000	1.000	1.000	1.000	1.000		
	014:01	62	0.020	7	0.045	1	0.987	1.000	0.987	0.778	1.000		
	005:06	25	0.008	1	0.006	1	1.000	1.000	1.000	1.000	1.000		
	013:01	16	0.005	2	0.013	1	0.987	0.000	1.000		0.987	005:03	0.5
	009:01N	14	0.004	1	0.006	1	1.000	1.000	1.000	1.000	1.000		

	allele	train.num	train.freq	valid.num	valid.freq	call.rate	accuracy	sensitivity	specificity	ppv	npv	miscall	miscall.prop
SAS	005:02	1232	0.388	73	0.354	1	1.000	1.000	1.000	1.000	1.000		
	004:01	613	0.193	25	0.121	1	1.000	1.000	1.000	1.000	1.000		

	002:01	594	0.187	40	0.194	1	1.000	1.000	1.000	1.000	1.000		
	008:01	265	0.083	8	0.039	1	1.000	1.000	1.000	1.000	1.000		
	005:03	145	0.046	37	0.180	1	1.000	1.000	1.000	1.000	1.000		
	003:01	116	0.037	19	0.092	1	1.000	1.000	1.000	1.000	1.000		
	005:06	25	0.008	2	0.010	1	1.000	1.000	1.000	1.000	1.000		
	028	15	0.005	1	0.005	1	1.000	1.000	1.000	1.000	1.000		
	005:08	12	0.004	1	0.005	1	1.000	1.000	1.000	1.000	1.000		
	allele	train.num	train.freq	valid.num	valid.freq	call.rate	accuracy	sensitivity	specificity	ppv	npv	miscall	miscall.prop
AMR	005:02	1232	0.388	99	0.550	1	1.000	1.000	1.000	1.000	1.000		
	004:01	613	0.193	23	0.128	1	1.000	1.000	1.000	1.000	1.000		
	002:01	594	0.187	27	0.150	1	1.000	1.000	1.000	1.000	1.000		
	008:01	265	0.083	8	0.044	1	1.000	1.000	1.000	1.000	1.000		
	005:03	145	0.046	4	0.022	1	1.000	1.000	1.000	1.000	1.000		
	003:01	116	0.037	5	0.028	1	1.000	1.000	1.000	1.000	1.000		
	014:01	62	0.020	1	0.006	1	1.000	1.000	1.000	1.000	1.000		
	005:06	25	0.008	2	0.011	1	1.000	1.000	1.000	1.000	1.000		
	005:01	18	0.006	4	0.022	1	1.000	1.000	1.000	1.000	1.000		
	013:01	16	0.005	1	0.006	1	1.000	1.000	1.000	1.000	1.000		
	009:01N	14	0.004	5	0.028	1	1.000	1.000	1.000	1.000	1.000		
	005:08	12	0.004	1	0.006	1	1.000	1.000	1.000	1.000	1.000		
	allele	train.num	train.freq	valid.num	valid.freq	call.rate	accuracy	sensitivity	specificity	ppv	npv	miscall	miscall.prop
FIN	005:02	1232	0.388	153	0.307	1	1.000	1.000	1.000	1.000	1.000		
	004:01	613	0.193	155	0.311	1	1.000	1.000	1.000	1.000	1.000		
	002:01	594	0.187	84	0.169	1	1.000	1.000	1.000	1.000	1.000		
	008:01	265	0.083	44	0.088	1	0.998	0.977	1.000	1.000	0.998	039	1
	005:03	145	0.046	19	0.038	1	1.000	1.000	1.000	1.000	1.000		
	003:01	116	0.037	9	0.018	1	1.000	1.000	1.000	1.000	1.000		
	014:01	62	0.020	13	0.026	1	1.000	1.000	1.000	1.000	1.000		
	005:06	25	0.008	1	0.002	1	1.000	1.000	1.000	1.000	1.000		
	039	23	0.007	10	0.020	1	0.998	1.000	0.998	0.909	1.000		
	005:01	18	0.006	1	0.002	1	1.000	1.000	1.000	1.000	1.000		

	013:01	16	0.005	4	0.008	1	1.000	1.000	1.000	1.000	1.000		
	028	15	0.005	4	0.008	1	1.000	1.000	1.000	1.000	1.000		
	005:08	12	0.004	1	0.002	1	1.000	1.000	1.000	1.000	1.000		

HLA-E

	allele	train.num	train.freq	valid.num	valid.freq	call.rate	accuracy	sensitivity	specificity	ppv	npv	miscall	miscall.prop
EUR	01:01	1656	0.500	187	0.574	1	1.000	1.000	1.000	1.000	1.000		
	01:03	1538	0.465	128	0.393	1	0.997	1.000	0.995	0.992	1.000		
	01:06	86	0.026	10	0.031	1	1.000	1.000	1.000	1.000	1.000		
	01:11	3	0.001	1	0.003	1	0.997	0.000	1.000		0.997	01:03	1

	allele	train.num	train.freq	valid.num	valid.freq	call.rate	accuracy	sensitivity	specificity	ppv	npv	miscall	miscall.prop
AFR	01:01	1656	0.500	154	0.535	1	0.997	1.000	0.993	0.994	1.000		
	01:03	1538	0.465	128	0.444	1	0.997	0.992	1.000	1.000	0.994	01:05	1
	01:05	12	0.004	5	0.017	1	0.997	1.000	0.996	0.833	1.000		
	01:13	2	0.001	1	0.003	1	0.997	0.000	1.000		0.997	01:01	1

	allele	train.num	train.freq	valid.num	valid.freq	call.rate	accuracy	sensitivity	specificity	ppv	npv	miscall	miscall.prop
EAS	01:01	1656	0.500	102	0.327	1	0.987	1.000	0.981	0.962	1.000		
	01:03	1538	0.465	207	0.663	1	0.997	0.995	1.000	1.000	0.991	01:01	1
	01:12	8	0.002	3	0.010	1	0.990	0.000	1.000		0.990	01:01	1

	allele	train.num	train.freq	valid.num	valid.freq	call.rate	accuracy	sensitivity	specificity	ppv	npv	miscall	miscall.prop
SAS	01:01	1656	0.500	122	0.545	1	1.000	1.000	1.000	1.000	1.000		
	01:03	1538	0.465	100	0.446	1	1.000	1.000	1.000	1.000	1.000		
	01:06	86	0.026	2	0.009	1	1.000	1.000	1.000	1.000	1.000		

	allele	train.num	train.freq	valid.num	valid.freq	call.rate	accuracy	sensitivity	specificity	ppv	npv	miscall	miscall.prop
AMR	01:01	1656	0.500	109	0.534	1	0.995	1.000	0.989	0.991	1.000		
	01:03	1538	0.465	93	0.456	1	1.000	1.000	1.000	1.000	1.000		
	01:06	86	0.026	1	0.005	1	1.000	1.000	1.000	1.000	1.000		
	01:09	3	0.001	1	0.005	1	0.995	0.000	1.000		0.995	01:01	1

FIN	allele	train.num	train.freq	valid.num	valid.freq	call.rate	accuracy	sensitivity	specificity	ppv	npv	miscall	miscall.prop
	01:01	1656	0.500	138	0.473	1	1.000	1.000	1.000	1.000	1.000		
	01:03	1538	0.465	124	0.425	1	1.000	1.000	1.000	1.000	1.000		
	01:06	86	0.026	30	0.103	1	1.000	1.000	1.000	1.000	1.000		

HLA-F

	allele	train.num	train.freq	valid.num	valid.freq	call.rate	accuracy	sensitivity	specificity	ppv	npv	miscall	miscall.prop
EUR	01:01	1696	0.843	156	0.857	1	1.000	1.000	1.000	1.000	1.000		
	01:03	297	0.148	24	0.132	1	1.000	1.000	1.000	1.000	1.000		
	01:04	9	0.004	2	0.011	1	1.000	1.000	1.000	1.000	1.000		
	allele	train.num	train.freq	valid.num	valid.freq	call.rate	accuracy	sensitivity	specificity	ppv	npv	miscall	miscall.prop
AFR	01:01	1696	0.843	196	0.760	1	1.000	1.000	1.000	1.000	1.000		
	01:03	297	0.148	59	0.229	1	1.000	1.000	1.000	1.000	1.000		
	01:02	6	0.003	3	0.012	1	1.000	1.000	1.000	1.000	1.000		
	allele	train.num	train.freq	valid.num	valid.freq	call.rate	accuracy	sensitivity	specificity	ppv	npv	miscall	miscall.prop
EAS	01:01	1696	0.843	112	0.982	1	0.991	1.000	0.500	0.991	1.000		
	01:03	297	0.148	1	0.009	1	1.000	1.000	1.000	1.000	1.000		
	01:05	4	0.002	1	0.009	1	0.991	0.000	1.000		0.991	01:01	1
	allele	train.num	train.freq	valid.num	valid.freq	call.rate	accuracy	sensitivity	specificity	ppv	npv	miscall	miscall.prop
SAS	01:01	1696	0.843	192	0.923	1	1.000	1.000	1.000	1.000	1.000		
	01:03	297	0.148	16	0.077	1	1.000	1.000	1.000	1.000	1.000		
	allele	train.num	train.freq	valid.num	valid.freq	call.rate	accuracy	sensitivity	specificity	ppv	npv	miscall	miscall.prop
AMR	01:01	1696	0.843	84	0.875	1	1.000	1.000	1.000	1.000	1.000		
	01:03	297	0.148	12	0.125	1	1.000	1.000	1.000	1.000	1.000		
	allele	train.num	train.freq	valid.num	valid.freq	call.rate	accuracy	sensitivity	specificity	ppv	npv	miscall	miscall.prop
FIN	01:01	1696	0.843	102	0.739	1	1.000	1.000	1.000	1.000	1.000		
	01:03	297	0.148	35	0.254	1	1.000	1.000	1.000	1.000	1.000		
	01:04	9	0.004	1	0.007	1	1.000	1.000	1.000	1.000	1.000		

Table D. Sensitivity, specificity, positive predictive value (PPV) and negative predictive value (NPV) for HLA-G alleles and HLA-G 3'UTR and 5' UTR haplotypes in the Finnish reference (model I).

**HLA-G**												
allele	train.num	train.freq	valid.num	valid.freq	call.rate	accuracy	sensitivity	specificity	ppv	npv	miscall	miscall.prop
01:01:01:01/02	201	0.340	104	0.374	1	0.996	1.000	0.994	0.990	1.000		
01:01:01:05	148	0.250	64	0.230	1	1.000	1.000	1.000	1.000	1.000		
01:01:02:01	102	0.172	49	0.176	1	0.993	0.959	1.000	1.000	0.991	01:01:14	1
01:04:01:01	49	0.083	22	0.079	1	0.996	1.000	0.996	0.957	1.000		
01:01:03:03	24	0.041	13	0.047	1	1.000	1.000	1.000	1.000	1.000		
01:06:01:01	22	0.037	10	0.036	1	1.000	1.000	1.000	1.000	1.000		
01:01:14	14	0.024	6	0.022	1	0.993	1.000	0.993	0.750	1.000		
01:03:01:02	10	0.017	4	0.014	1	1.000	1.000	1.000	1.000	1.000		
01:01:22	6	0.010	2	0.007	1	1.000	1.000	1.000	1.000	1.000		
01:01:01:04	4	0.007	1	0.004	1	1.000	1.000	1.000	1.000	1.000		
01:04:04	3	0.005	1	0.004	1	1.000	1.000	1.000	1.000	1.000		
01:01:01:09	1	0.002	1	0.004	1	0.996	0.000	1.000		0.996	01:01:01:01/02	1
01:21N	1	0.002	1	0.004	1	0.996	0.000	1.000		0.996	01:04:01:01	1
**HLA-G 3'UTR**												
allele	train.num	train.freq	valid.num	valid.freq	call.rate	accuracy	sensitivity	specificity	ppv	npv	miscall	miscall.prop
UTR-1	210	0.358	98	0.345	1	1.000	1.000	1.000	1.000	1.000		
UTR-2	145	0.247	65	0.229	1	1.000	1.000	1.000	1.000	1.000		
UTR-4	137	0.234	75	0.264	1	1.000	1.000	1.000	1.000	1.000		
UTR-3	50	0.085	27	0.095	1	1.000	1.000	1.000	1.000	1.000		
UTR-7	25	0.043	12	0.042	1	1.000	1.000	1.000	1.000	1.000		
UTR-5	10	0.017	4	0.014	1	1.000	1.000	1.000	1.000	1.000		
UTR-18	4	0.007	2	0.007	1	1.000	1.000	1.000	1.000	1.000		
UTR-6	4	0.007	1	0.004	1	1.000	1.000	1.000	1.000	1.000		
**HLA-G 5'UTR**												
allele	train.num	train.freq	valid.num	valid.freq	call.rate	accuracy	sensitivity	specificity	ppv	npv	miscall	miscall.prop
010101a	210	0.358	102	0.359	1	1.000	1.000	1.000	1.000	1.000		

010102a	162	0.276	83	0.292	1	0.996	1.000	0.995	0.988	1.000		
010101b	99	0.169	44	0.155	1	1.000	1.000	1.000	1.000	1.000		
010104a	50	0.085	25	0.088	1	0.996	1.000	0.996	0.962	1.000		
010101c	47	0.080	22	0.077	1	1.000	1.000	1.000	1.000	1.000		
0103a	8	0.014	4	0.014	1	1.000	1.000	1.000	1.000	1.000		
010101f	5	0.009	2	0.007	1	1.000	1.000	1.000	1.000	1.000		
010104b	2	0.003	1	0.004	1	0.996	0.000	1.000		0.996	010104a	1
other	2	0.003	1	0.004	1	0.996	0.000	1.000		0.996	010102a	1

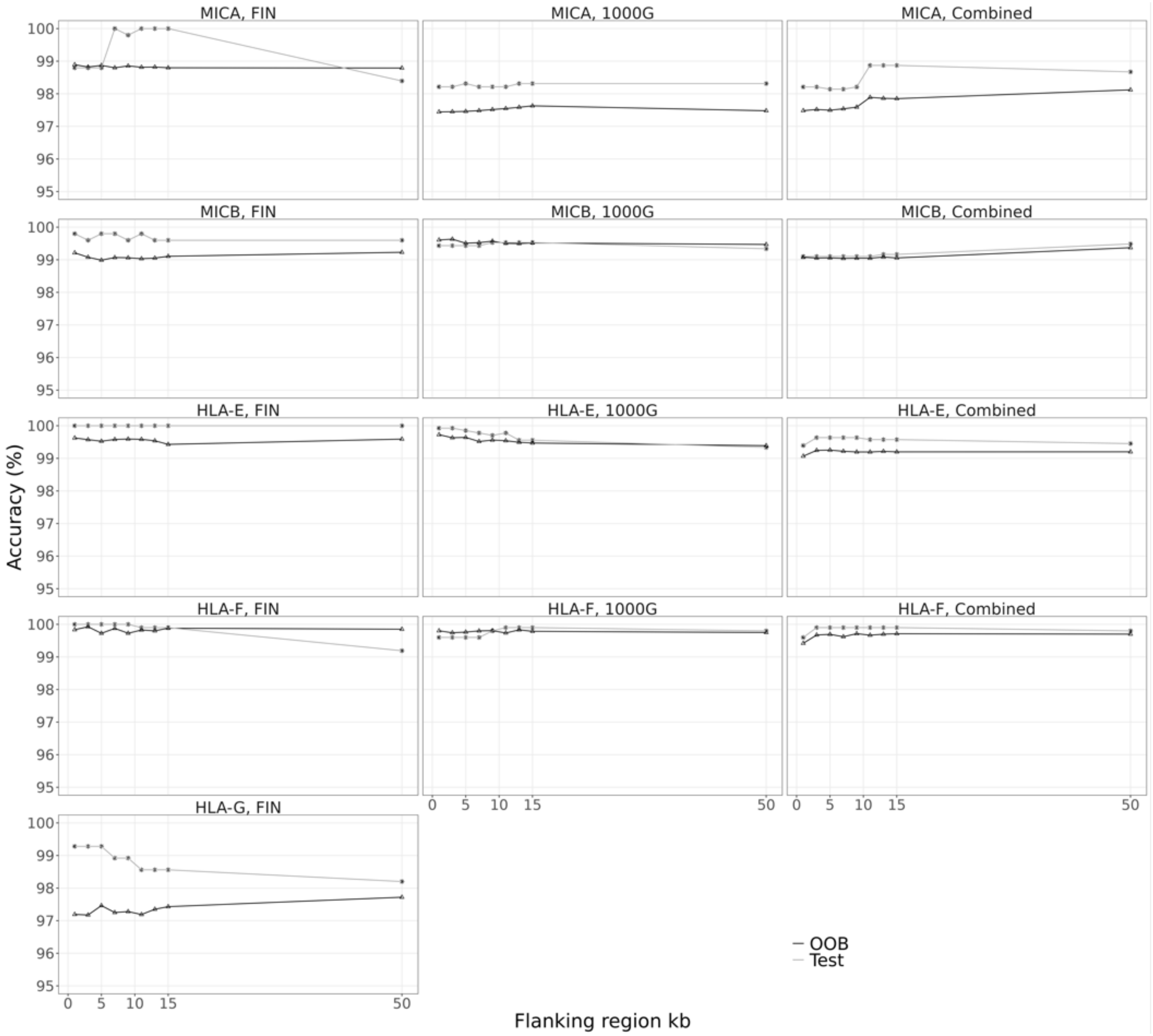

Fig A. Effect of the flanking region size on out-of bag (OOB) and test accuracy. OOB and test accuracies were evaluated in the Finnish (II), 1000 Genomes (V) and combined Finnish and 1000 Genomes (VI) references when using SNPs in the flanking regions from 1 to 15 and 50 kb on each side of the gene. Model for HLA-G was built using only the Finnish reference (I). A flanking region of 10 kb was chosen for the training and cross-validation of the imputation models.

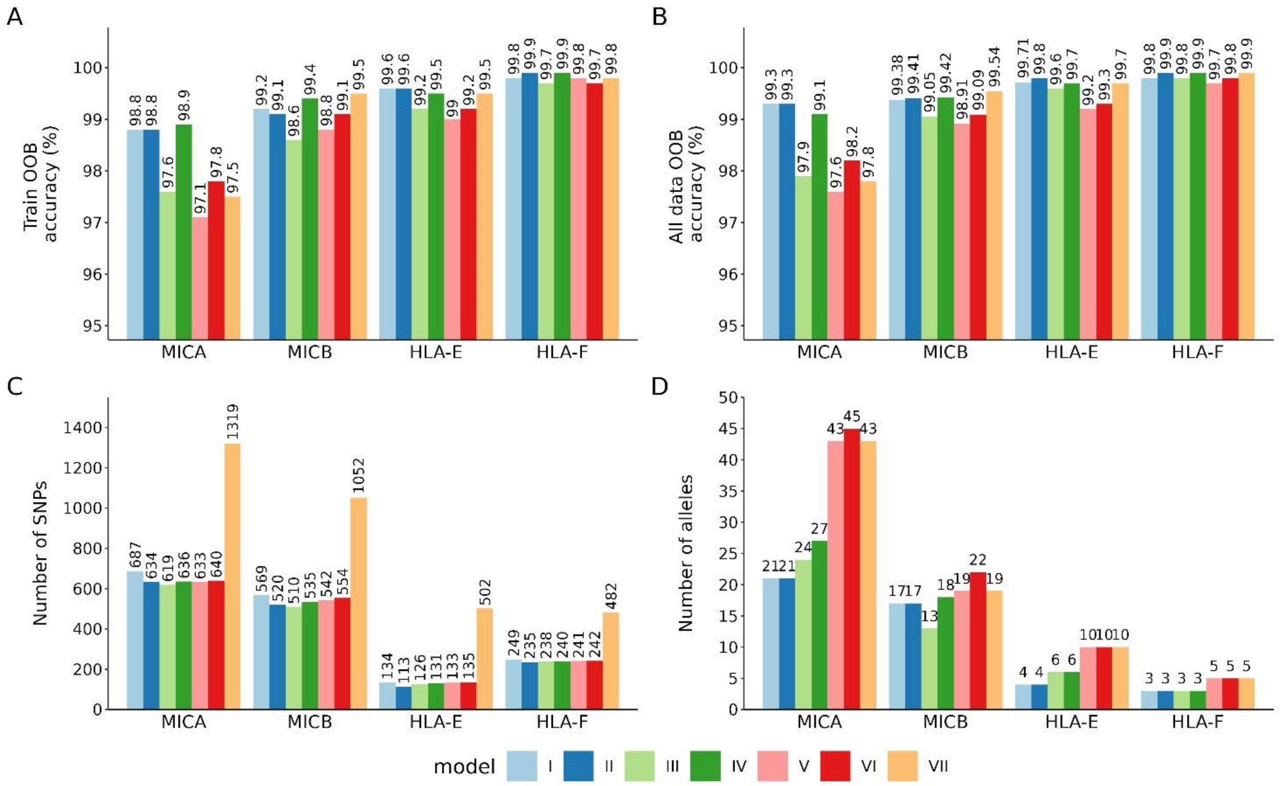

Fig B. Model properties of the 1000G/FIN models. A) Out-of-bag (OOB) accuracies of the models trained with the training data. B) OOB accuracies of the models trained with all reference data. C) Number of SNP markers within 10 kb flanking region used in the training of the models. D) Number of alleles present in the models.

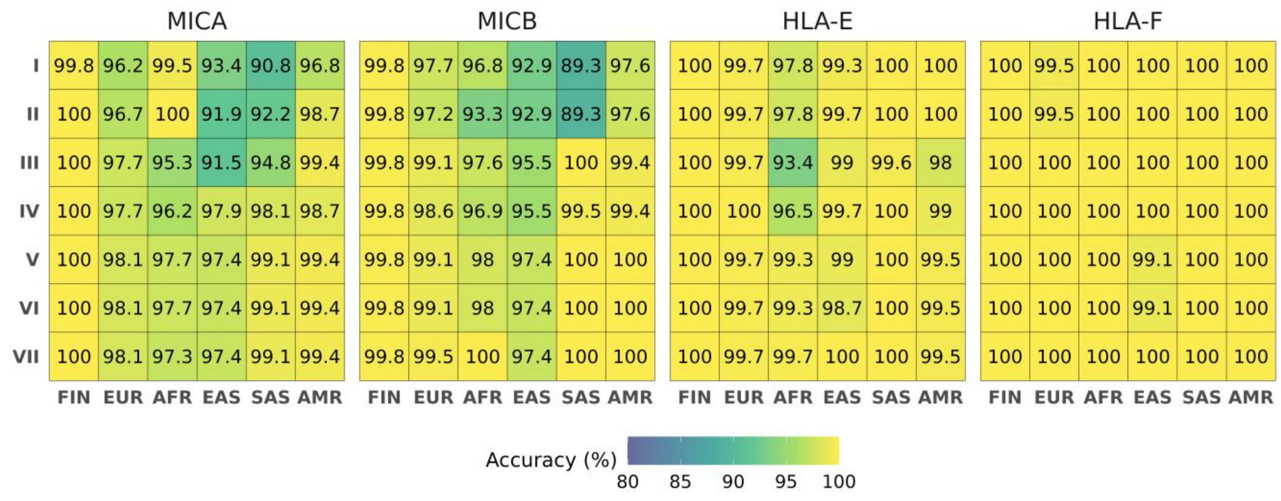

Fig C. Overall imputation accuracies in the Finnish and the 1000 Genomes superpopulations when omitting the untrained alleles, i.e. alleles not present in the training data.

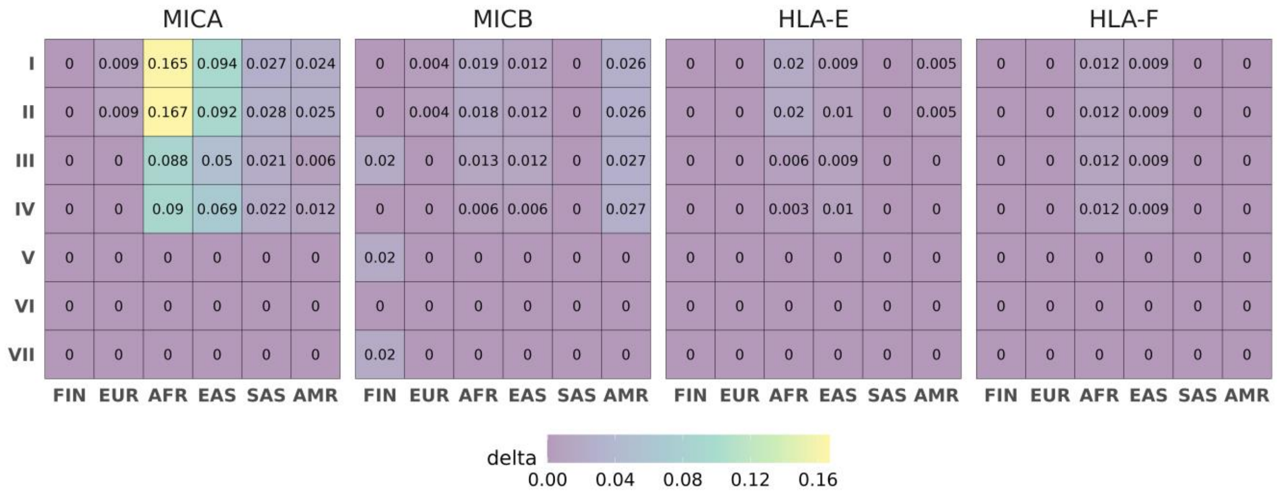

Fig D. Difference in percentage point in the overall imputation accuracy when omitting untrained alleles (i.e. alleles not present in the training set) versus without omitting (i.e. all alleles present in the test set).

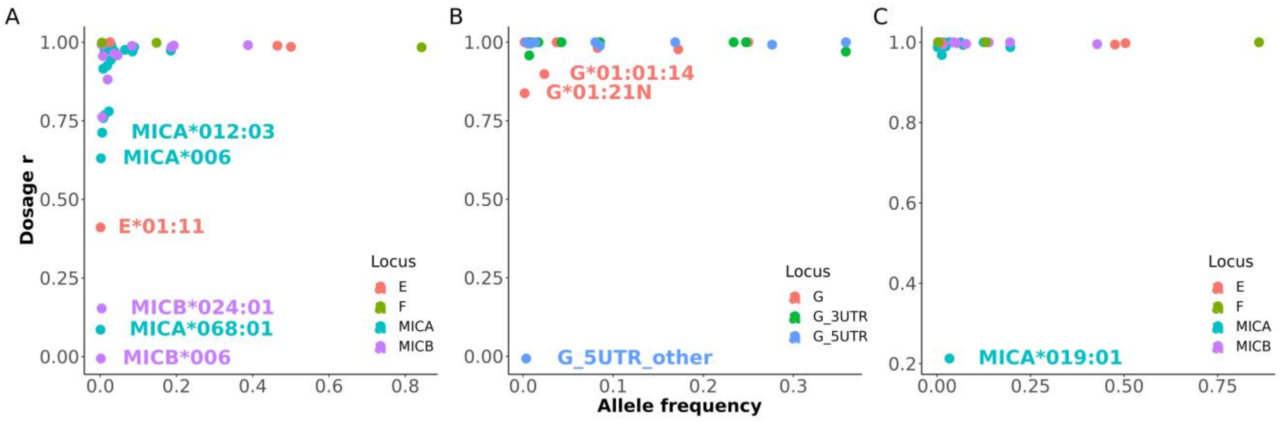

Fig E. Pearson's correlation (r) of imputed and true allele dosages versus allele frequency. The imputation posterior probability was considered in calculating the allele dosages. A) The combined 1000G and Finnish reference (model VI) for MICA, MICB, HLA-E and HLA-F. B) The Finnish reference (model I) for HLA-G, HLA-G 3'UTR and HLA-G 5'UTR. C) The cross-validation of 1000G reference with the Finnish reference as target. Model VII was trained using the 1000 Genomes reference and applied to the Finnish reference with clinical-grade typing quality.

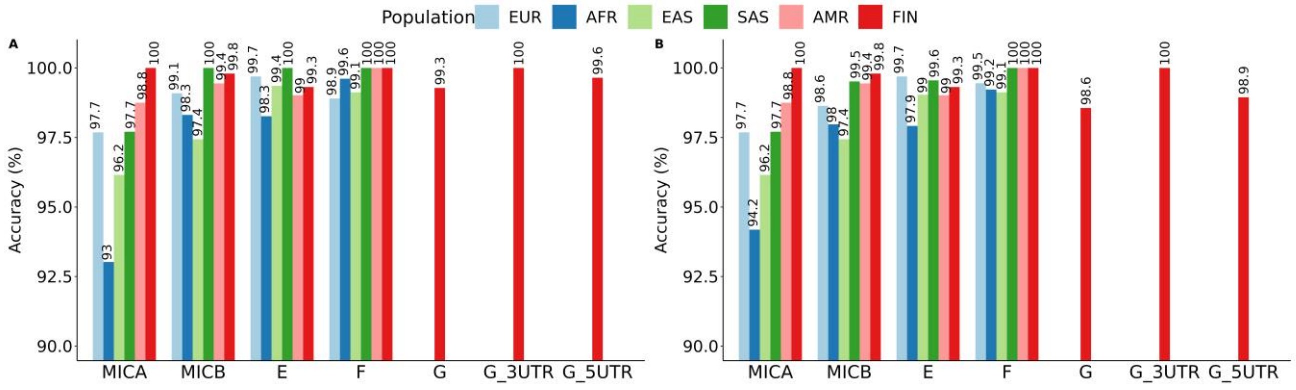

Model	Training_n	All_data_n	SNPs_total	Alleles	OOB_train	OOB_All_data
MICA	1563	2316	2075	45	97.2	97.6
MICB	1589	2367	2013	23	99.0	99.1
HLA-E	1655	2478	2035	10	99.2	99.4
HLA-F	1006	1504	1717	5	99.7	99.8
HLA-G	296	435	1796	20	97.8	98.4
HLA-G 3UTR	293	435	1800	9	99.5	99.7
HLA-G 5UTR	293	435	1803	10	99.1	99.4

Model	Training_n	All_data_n	SNPs_total	Alleles	OOB_train	OOB_All_data
MICA	1563	2316	1890	45	97.1	97.6
MICB	1589	2367	1790	23	98.6	98.9
HLA-E	1655	2478	1654	10	99.1	99.2
HLA-F	1006	1504	1703	5	99.7	99.8
HLA-G	296	435	1591	20	97.8	98.5
HLA-G 3UTR	293	435	1597	9	99.5	99.7
HLA-G 5UTR	293	435	1599	10	99.1	99.3

Fig F. Cross-validation imputation accuracies and properties of the models fitted for GSA and PMRA SNP content. The models were trained using the combined Finnish/1000 Genomes reference (reference combination VI) and the markers common in the Finnish/1000G reference and (A) Global Screening Array (GSA, Illumina, Inc.) or (B) Axiom Precision Medicine Research Array (PMRA, Thermo Fisher Scientific, Inc.).
