# Supplementary material for: Accurate multi-population imputation of MICA, MICB, HLA-E, HLA-F and HLA-G alleles from genome SNP data
Source: PLoS Comput Biol. 2024 Sep 16;20(9):e1011718. doi: 10.1371/journal.pcbi.1011718 (PMC11426482; doi:10.1371/journal.pcbi.1011718)

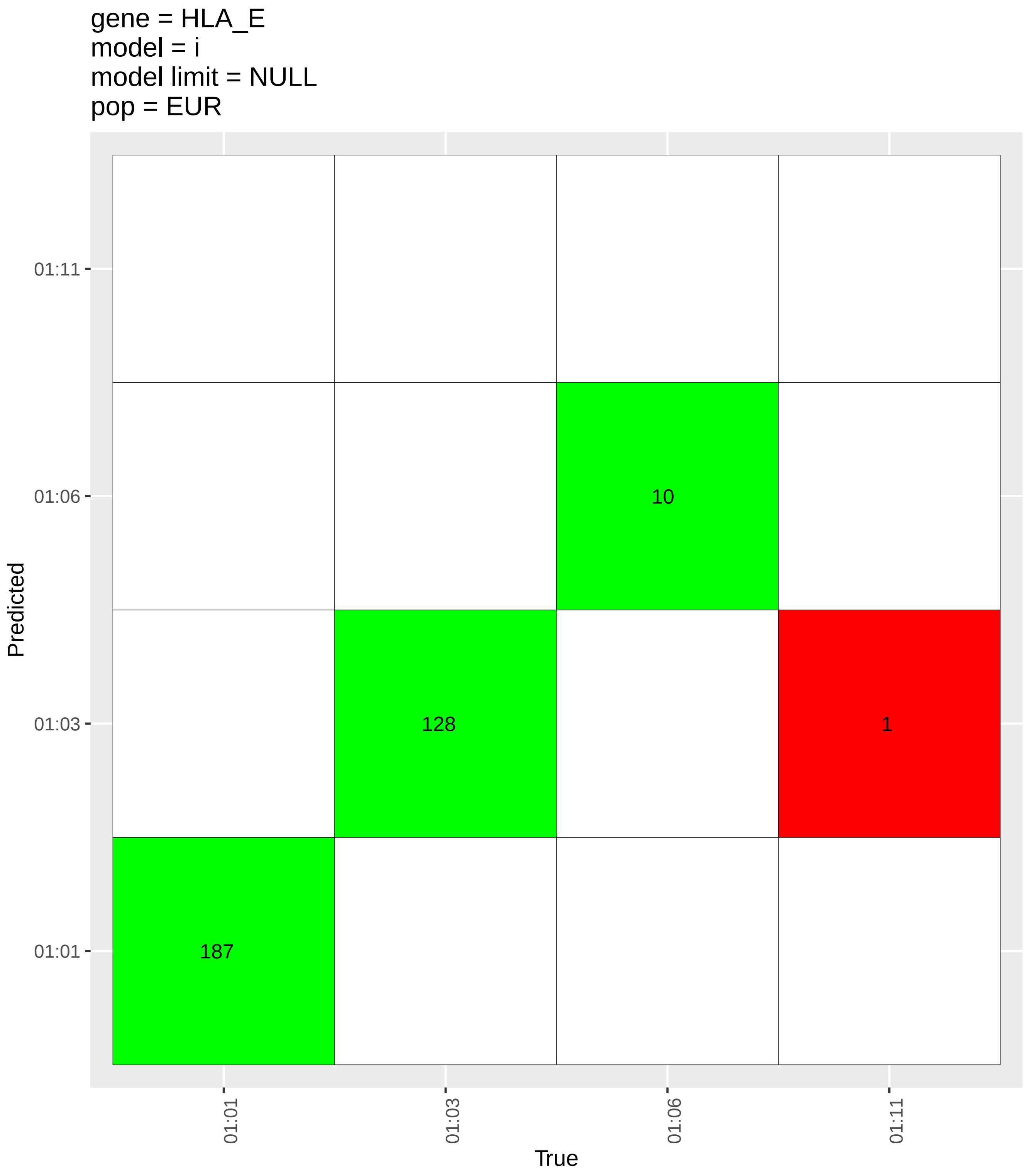
gene = HLA_E
model = i
model limit = NULL
pop = EUR
Predicted
01:11
01:06
01:03
01:01
10
128
1
187
01:01
01:03
01:06
01:11
True

gene = HLA_E
model = i
model limit = NULL
pop = AFR

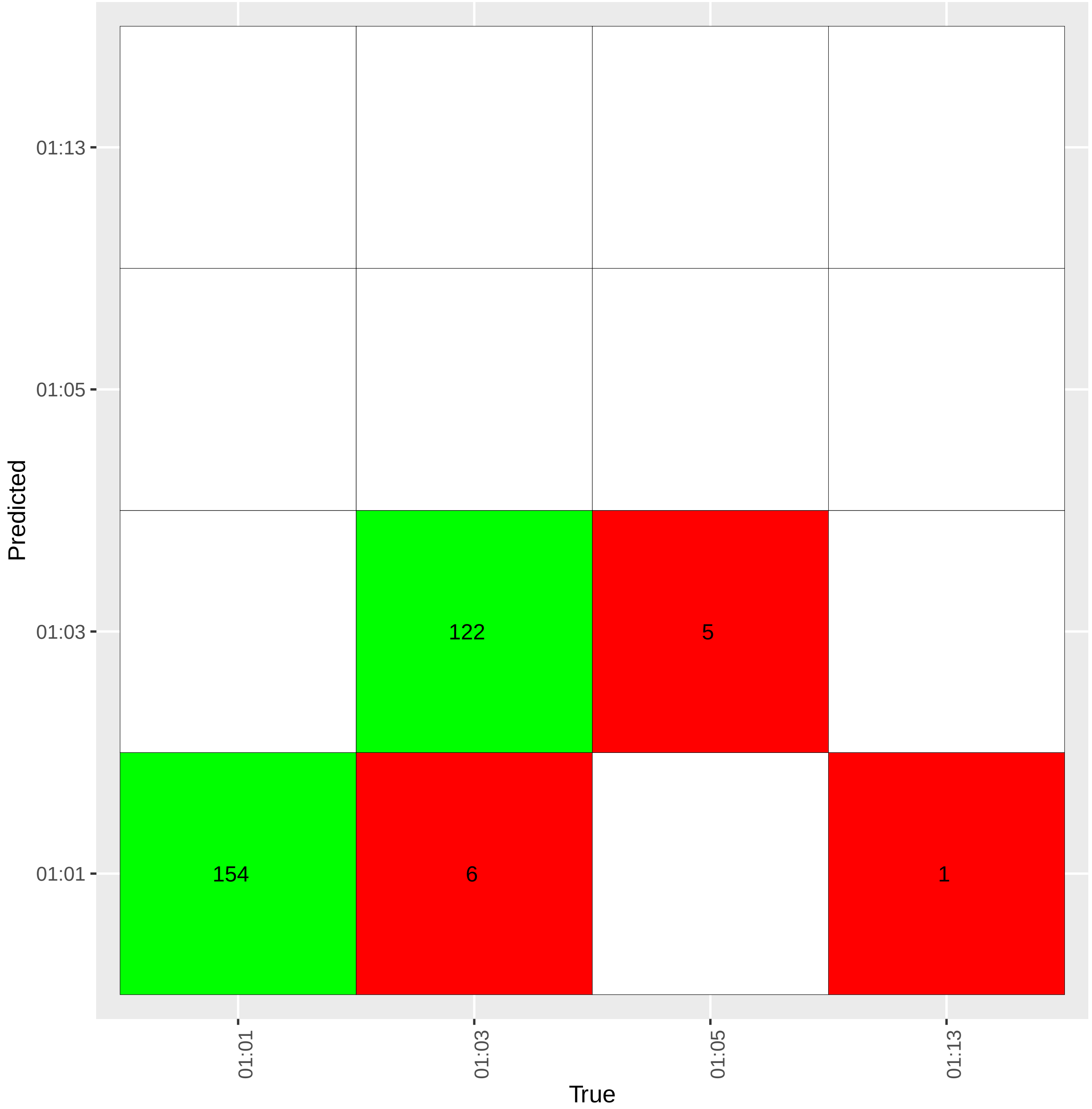

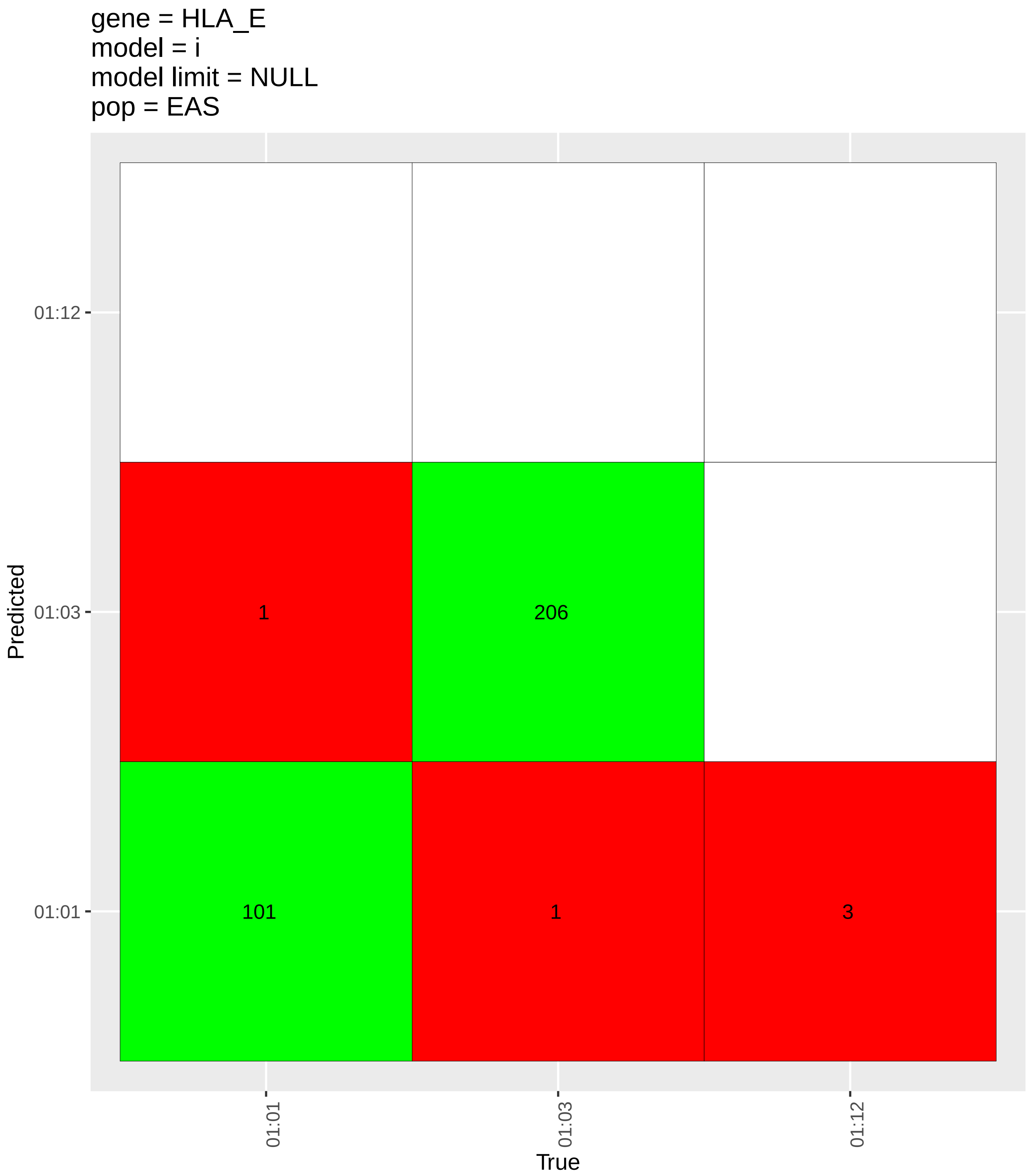
gene = HLA_E
model = i
model limit = NULL
pop = EAS
Predicted
01:12
01:03
01:01
1
206
101
1
3
01:01
01:03
01:12
True

gene = HLA_E
model = i
model limit = NULL
pop = SAS

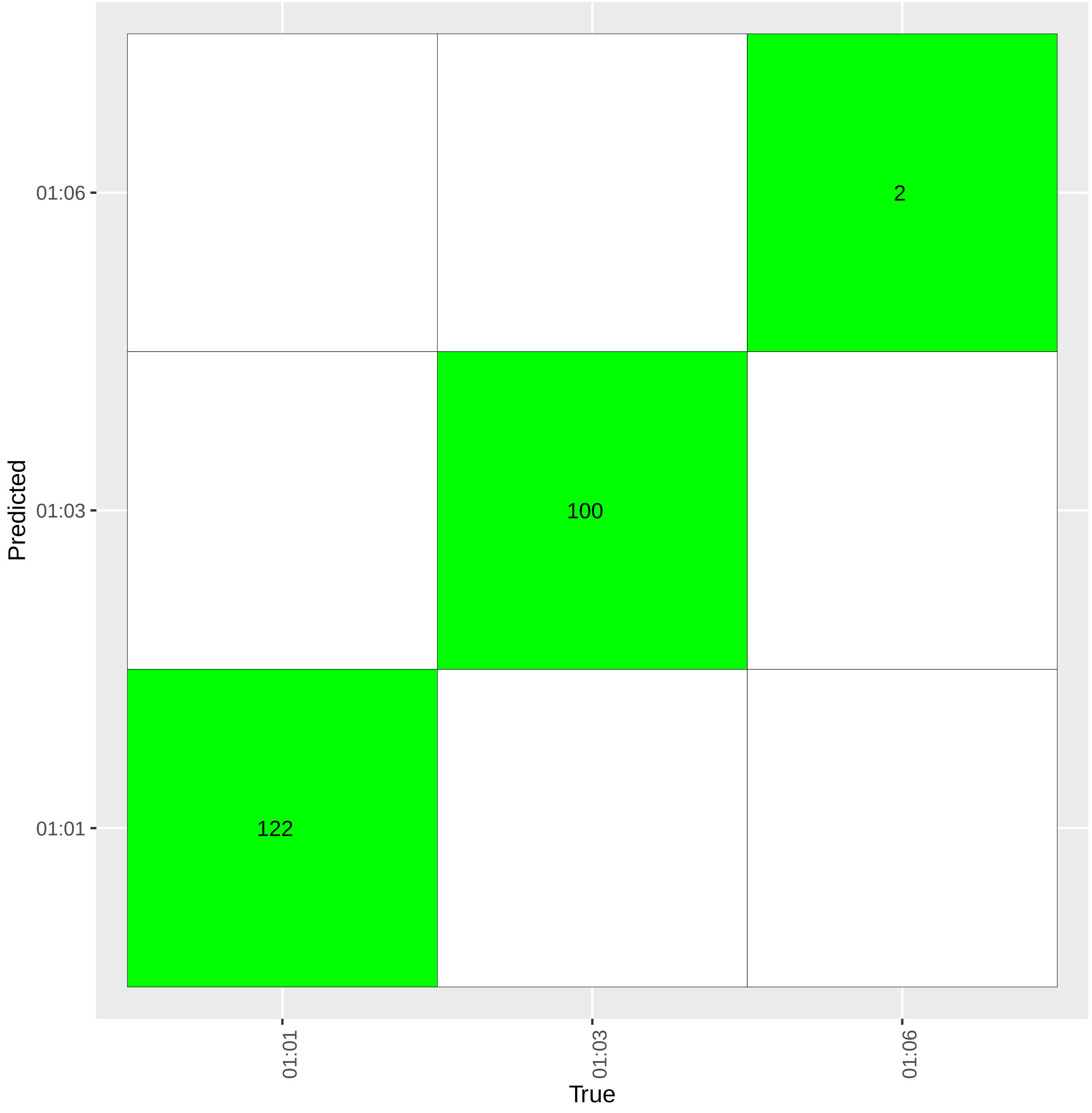

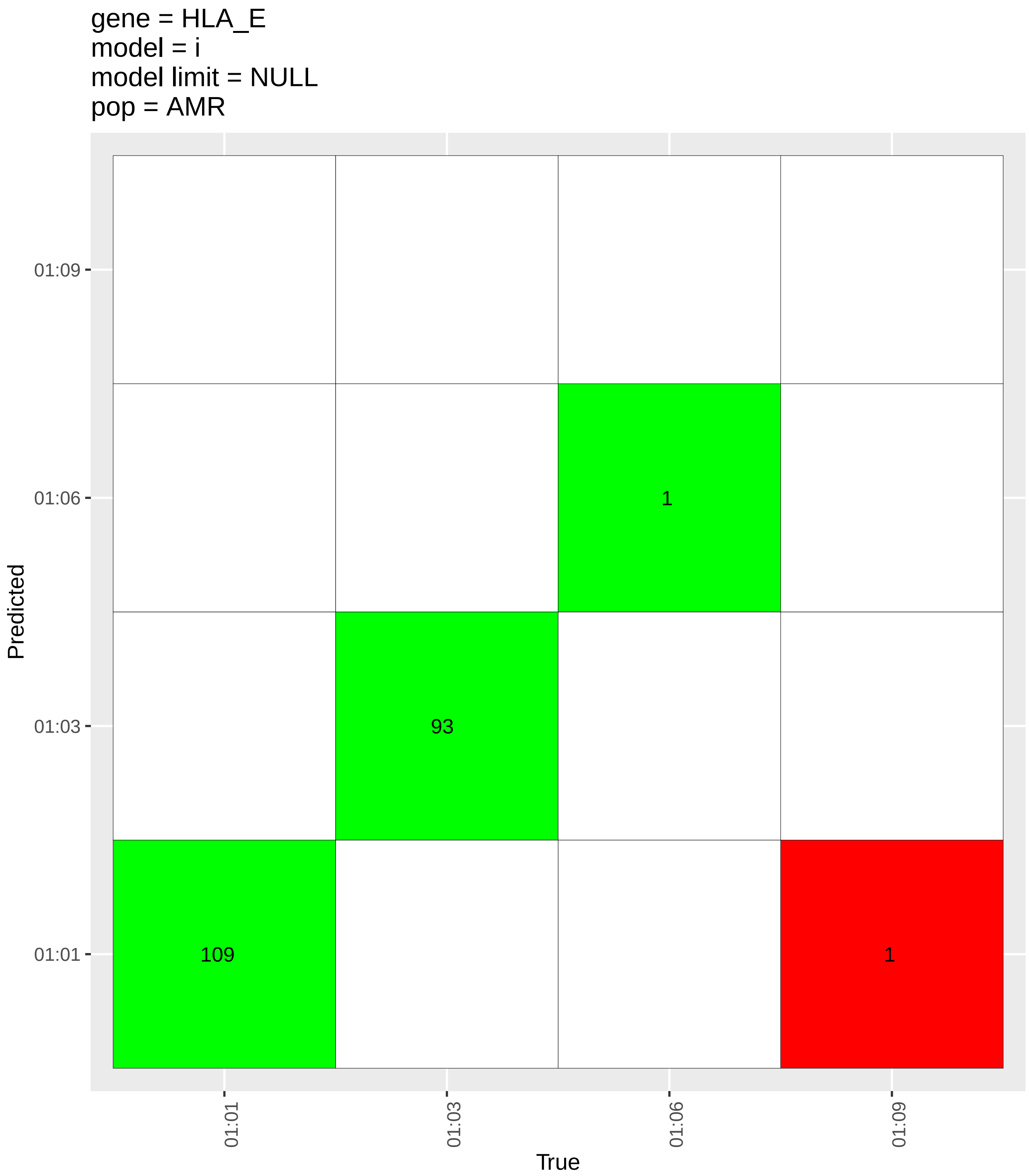
gene = HLA_E
model = i
model limit = NULL
pop = AMR
Predicted
01:09
01:06
01:03
01:01
1
93
109
1
01:01
01:03
01:06
01:09
True

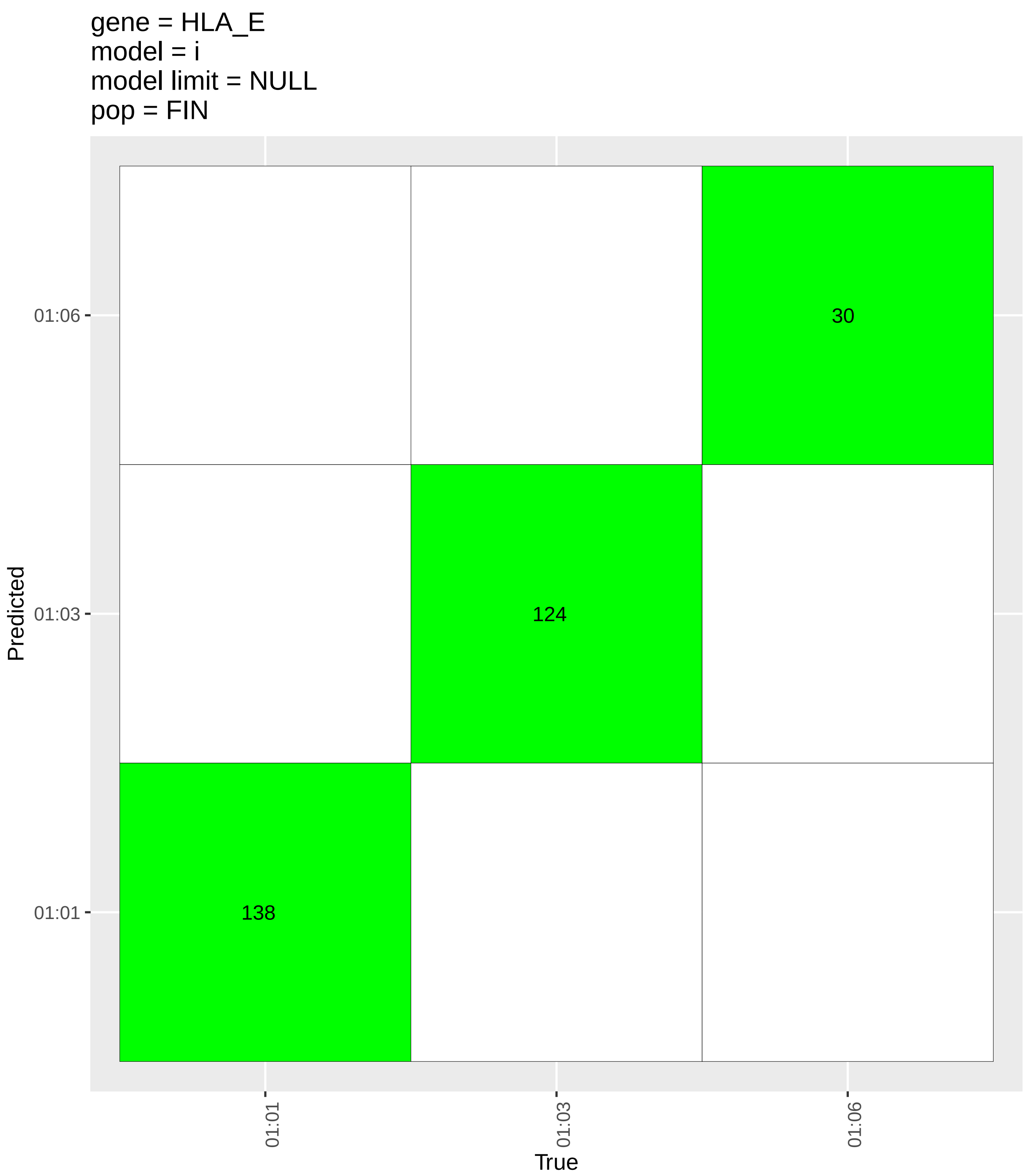
gene = HLA_E
model = i
model limit = NULL
pop = FIN
Predicted
01:06
01:03
01:01
30
124
138
01:01
01:03
01:06
True

gene = HLA_E
model = ii
model limit = NULL
pop = EUR

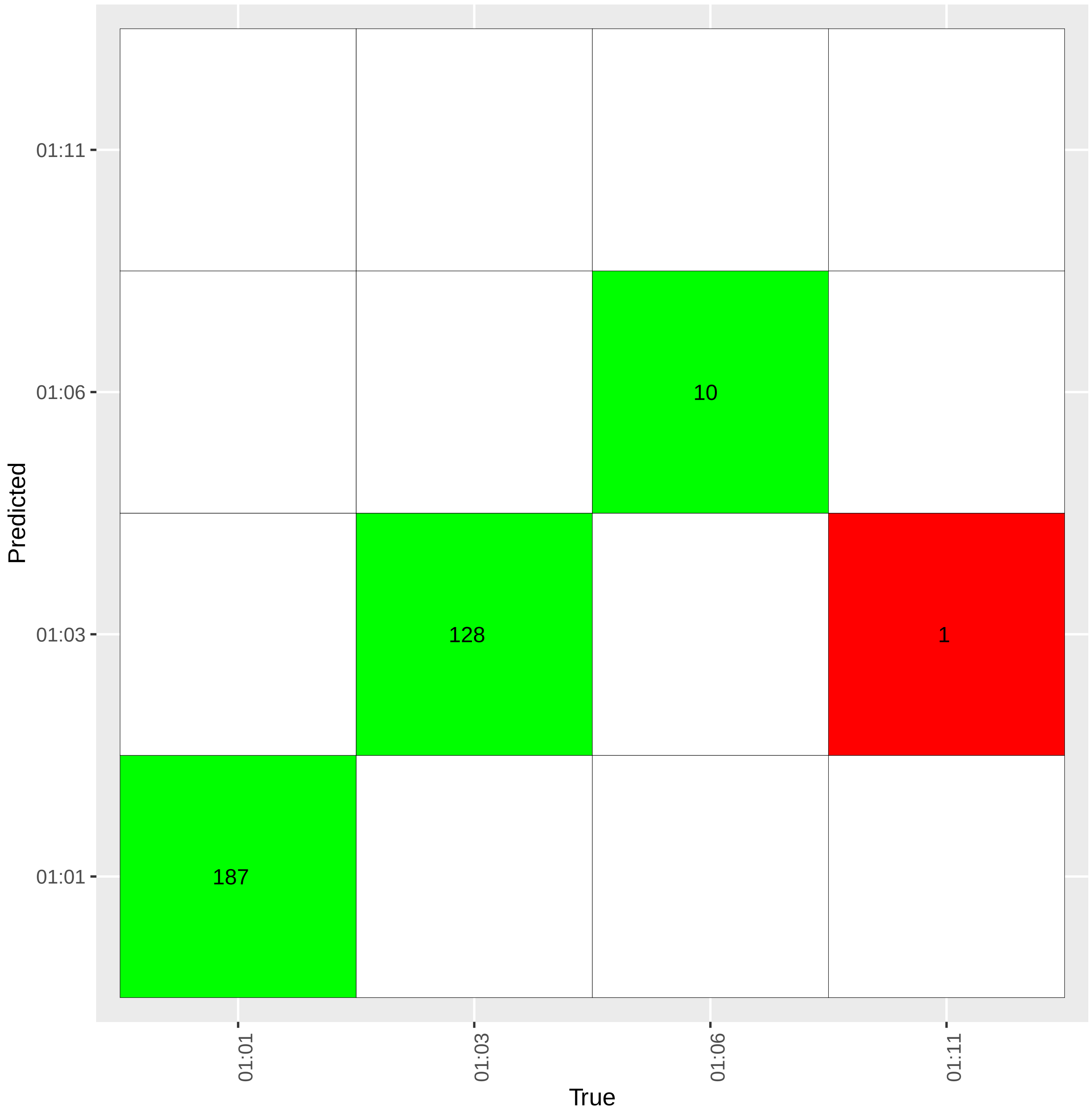

gene = HLA_E
model = ii
model limit = NULL
pop = AFR

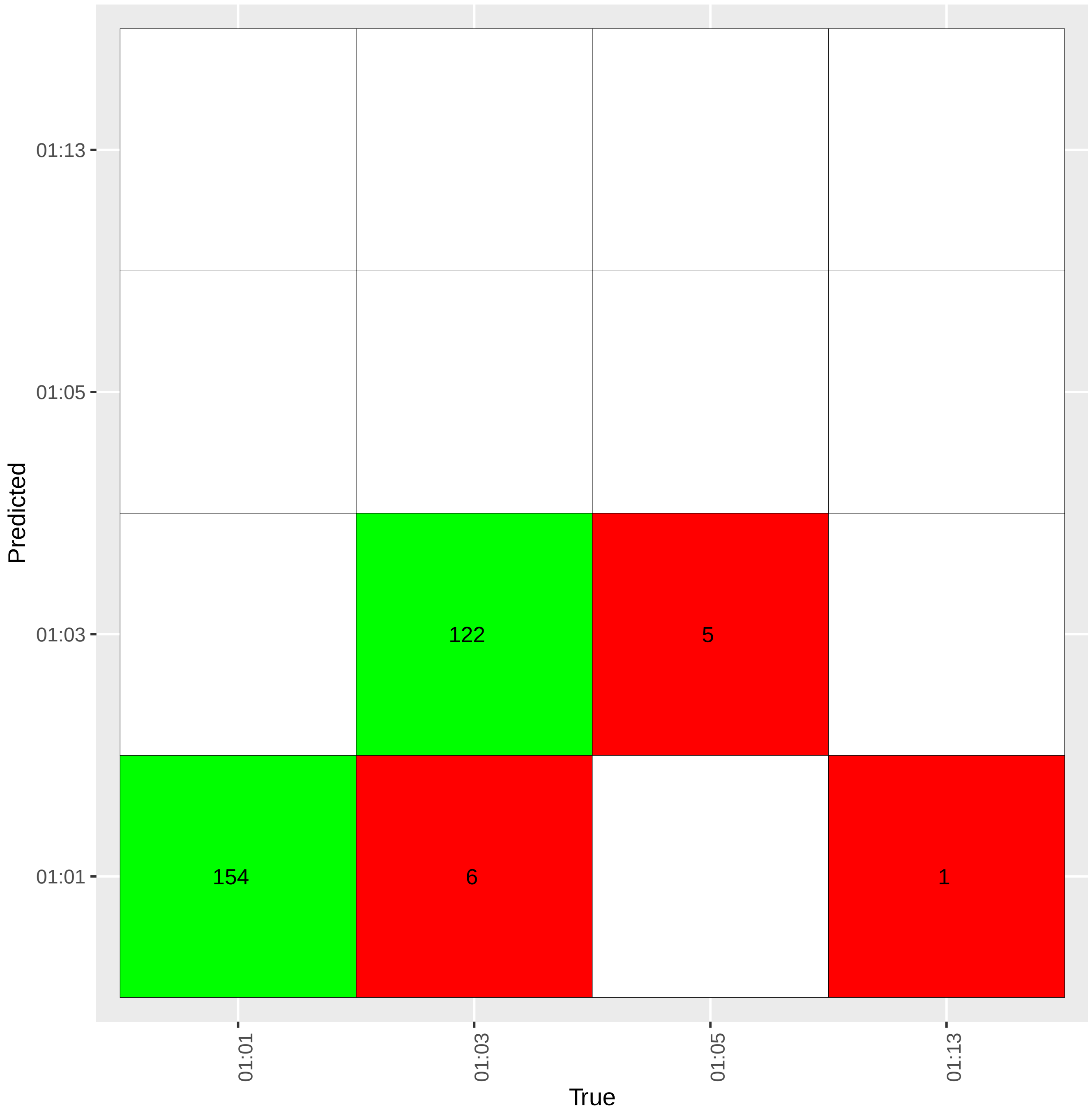

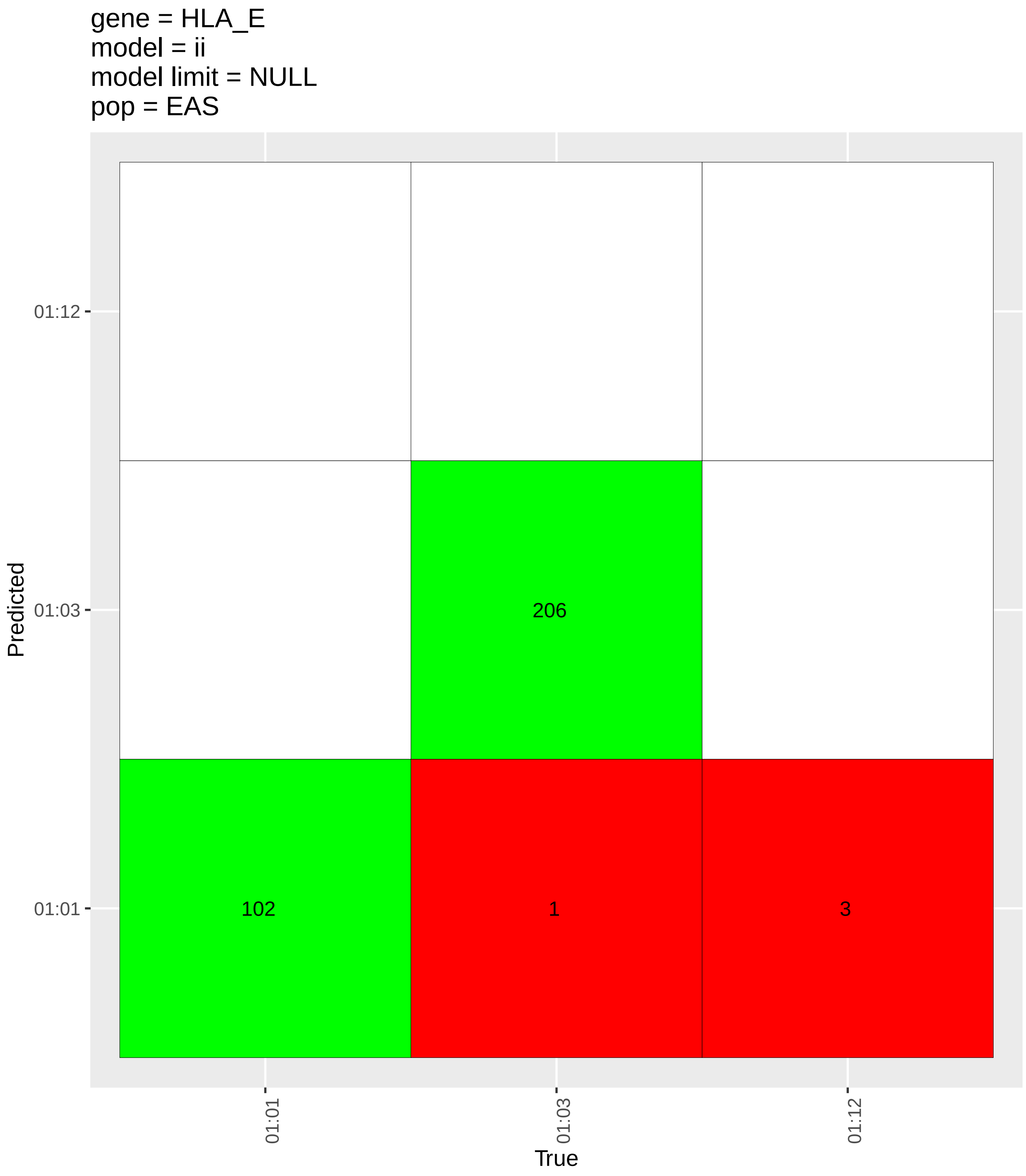
gene = HLA_E
model = ii
model limit = NULL
pop = EAS
Predicted
01:12
01:03
01:01
206
102
1
3
01:01
01:03
01:12
True

gene = HLA_E
model = ii
model limit = NULL
pop = SAS

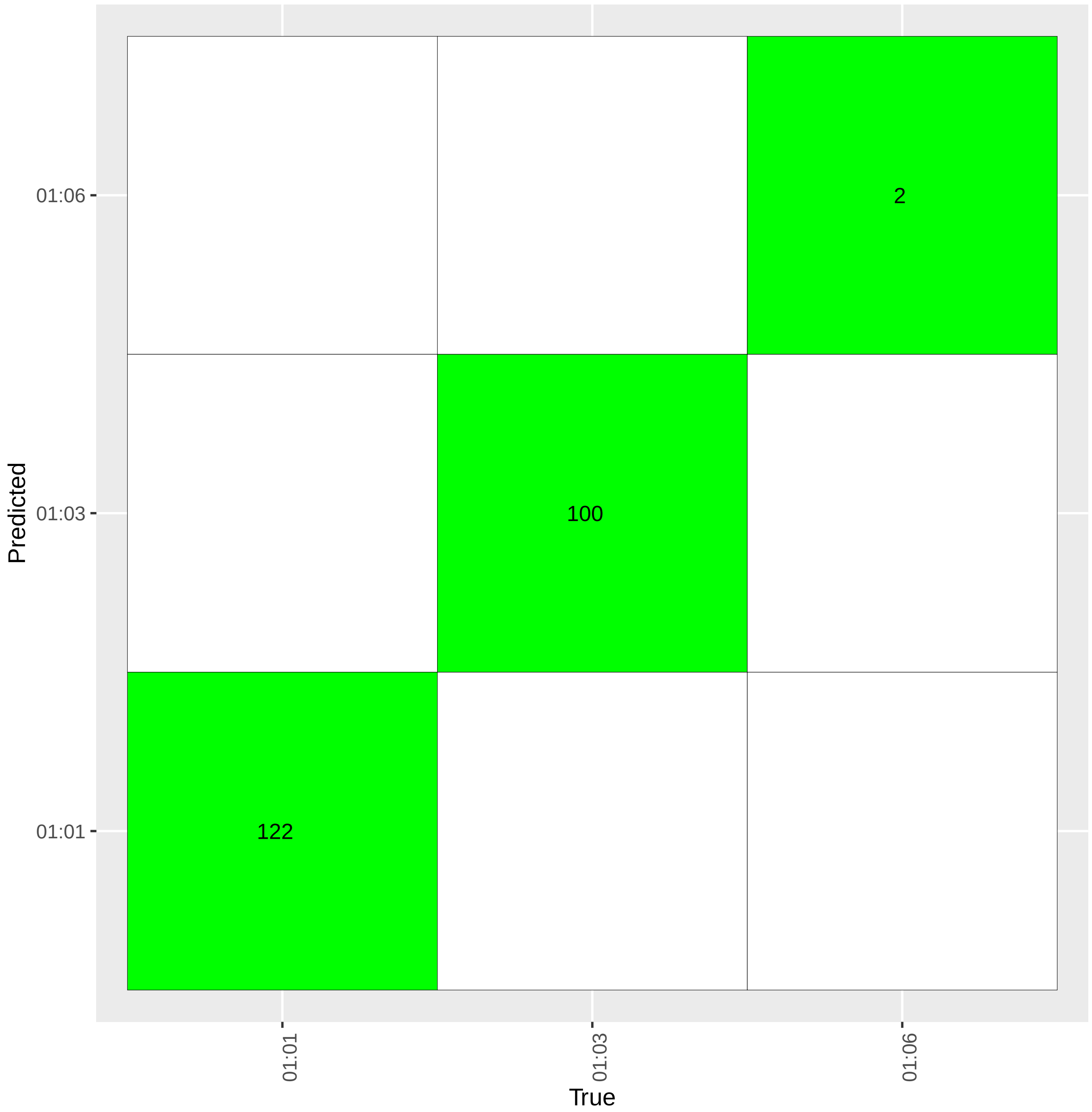

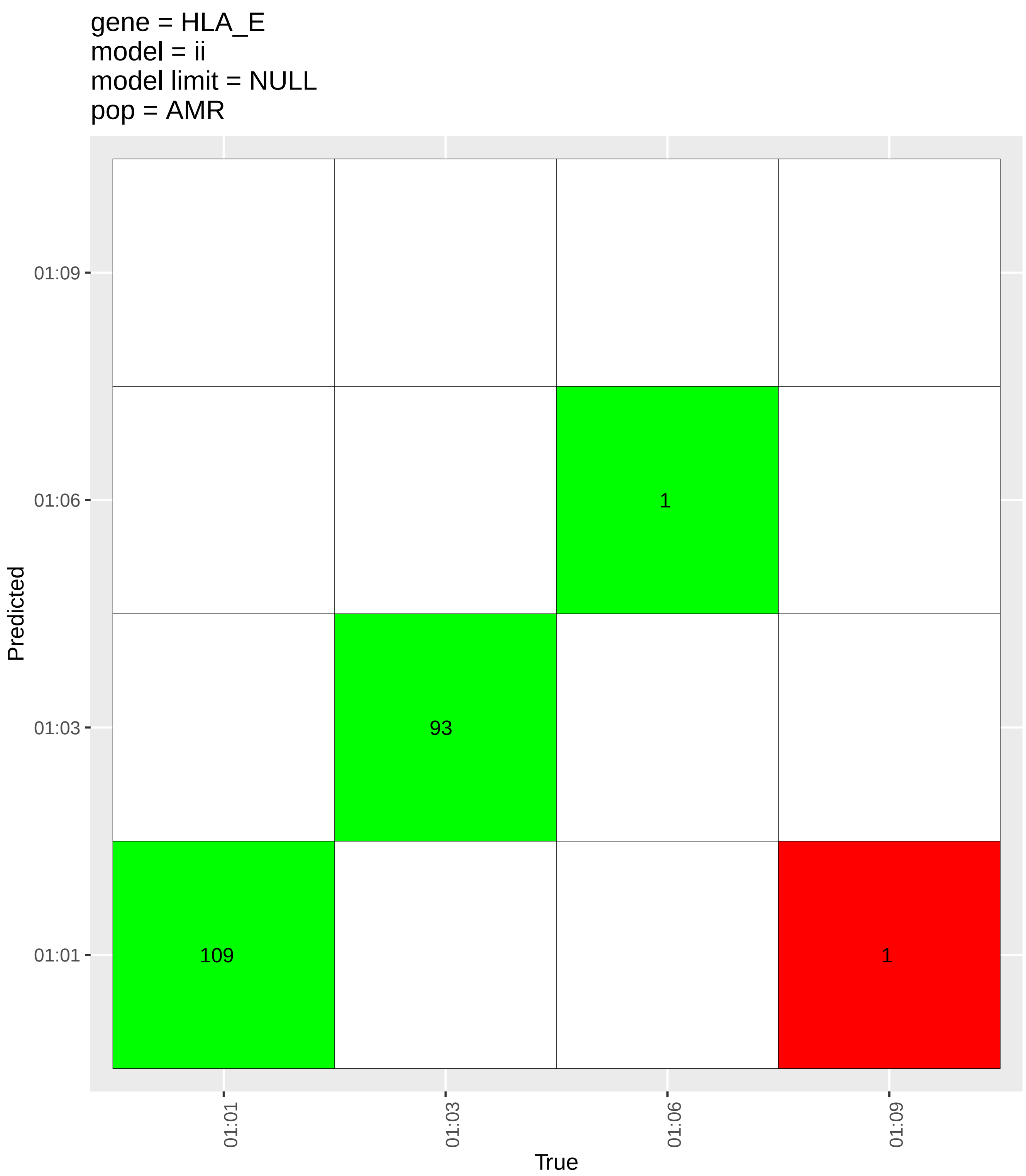
gene = HLA_E
model = ii
model limit = NULL
pop = AMR
Predicted
01:09
01:06
01:03
01:01
1
93
109
1
01:01
01:03
01:06
01:09
True

gene = HLA_E
model = ii
model limit = NULL
pop = FIN

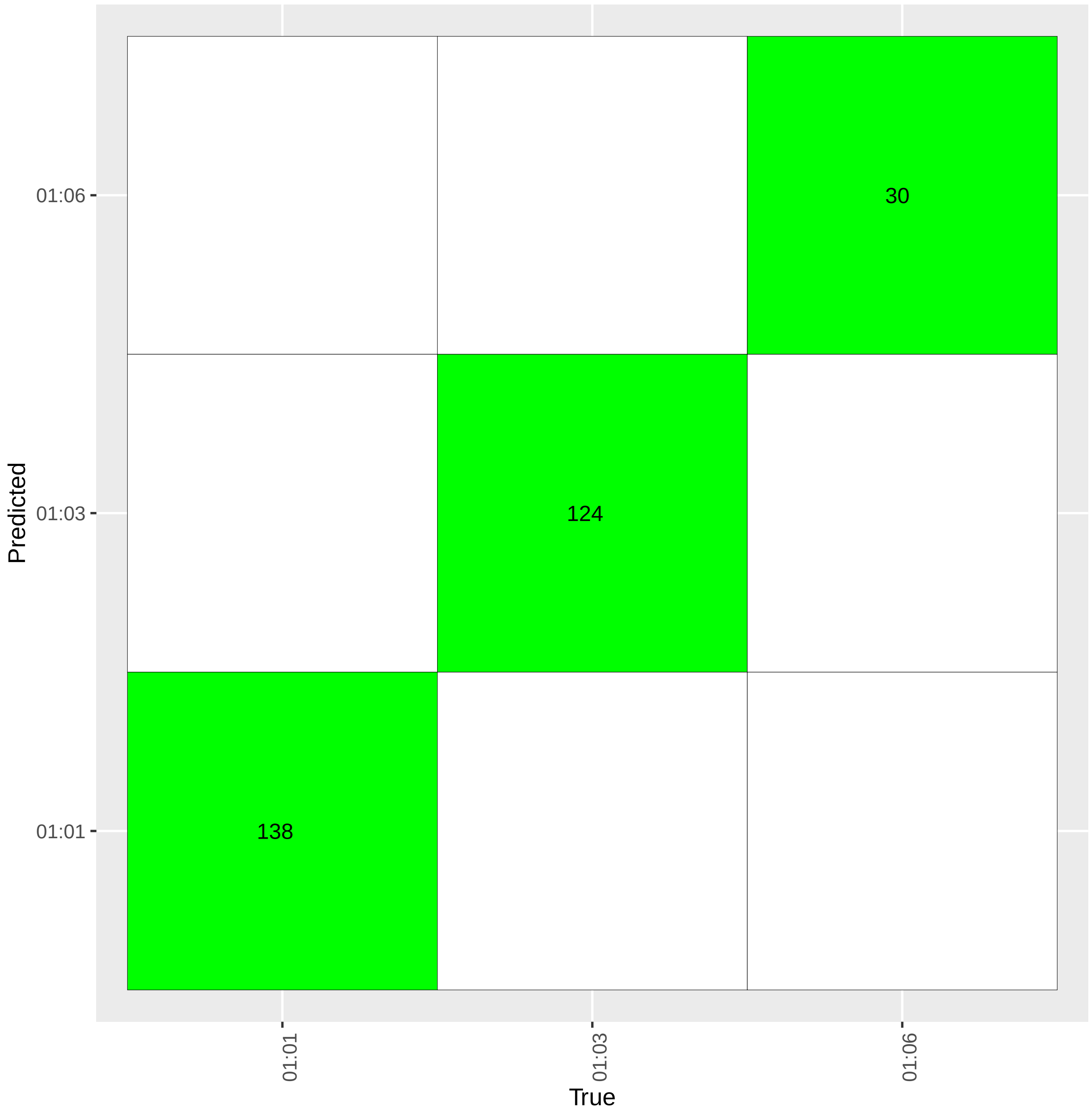

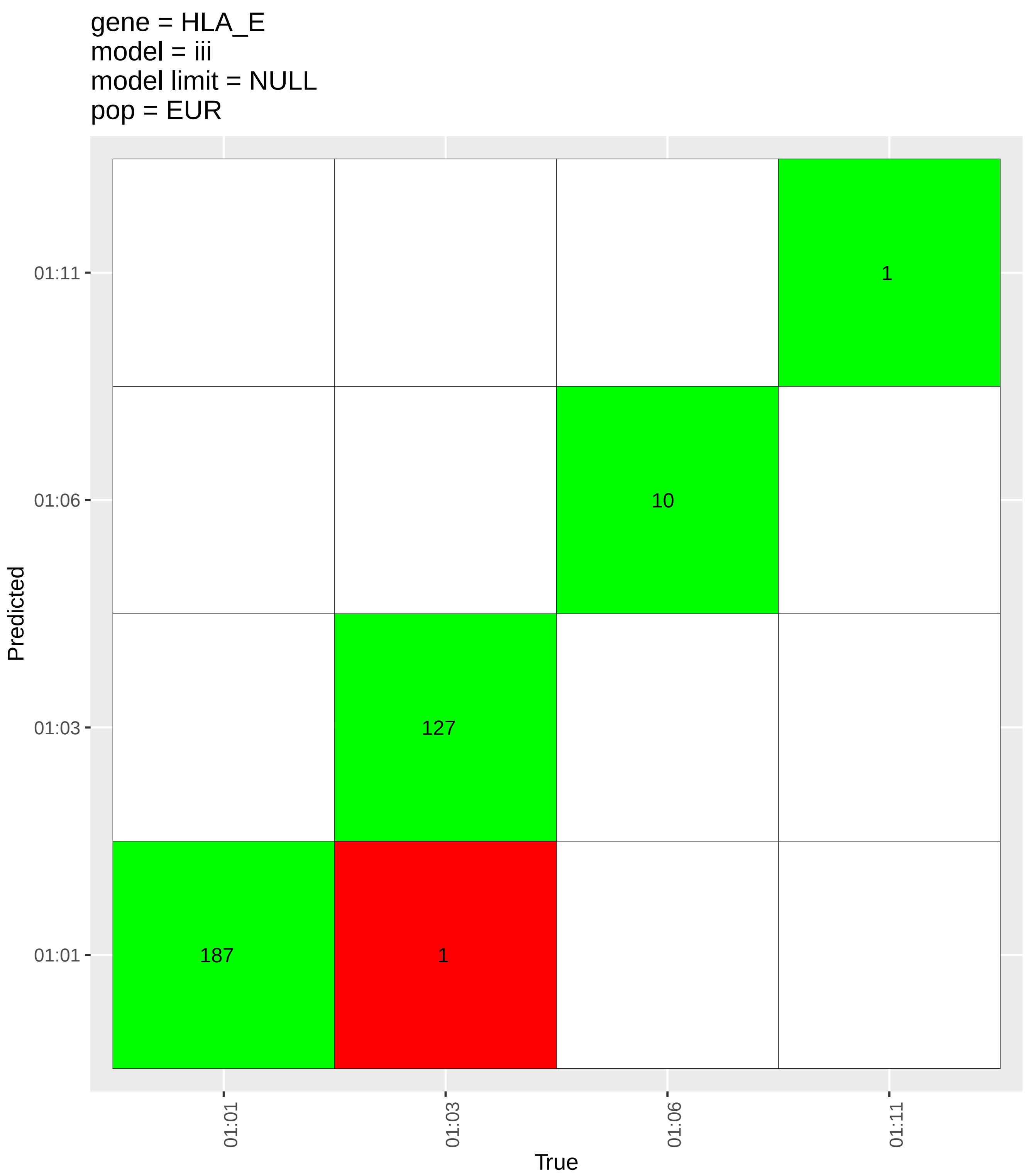
gene = HLA_E
model = iii
model limit = NULL
pop = EUR
Predicted
01:11
01:06
01:03
01:01
1
10
127
187
1
01:01
01:03
01:06
01:11
True

gene = HLA_E
model = iii
model limit = NULL
pop = AFR

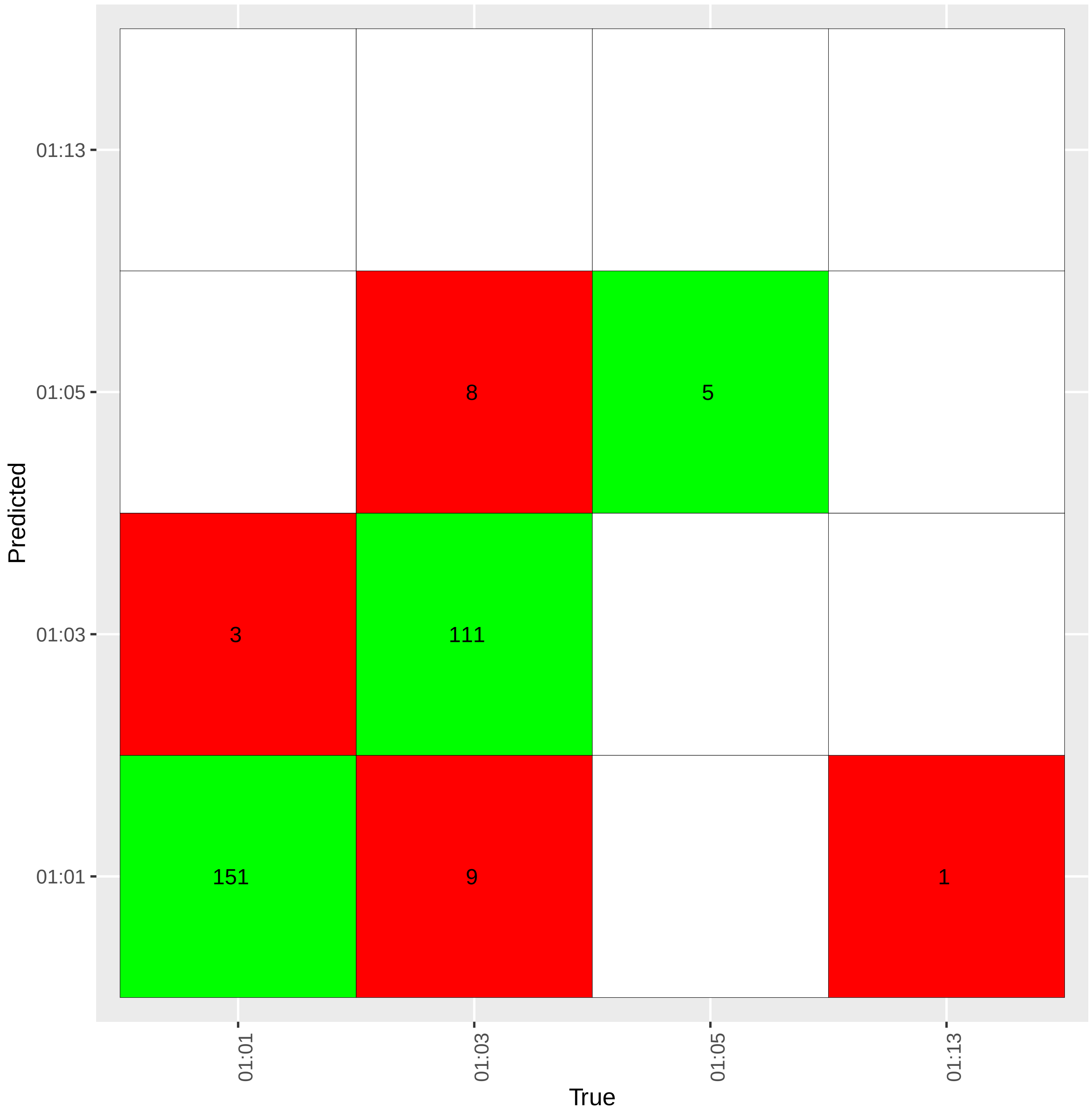

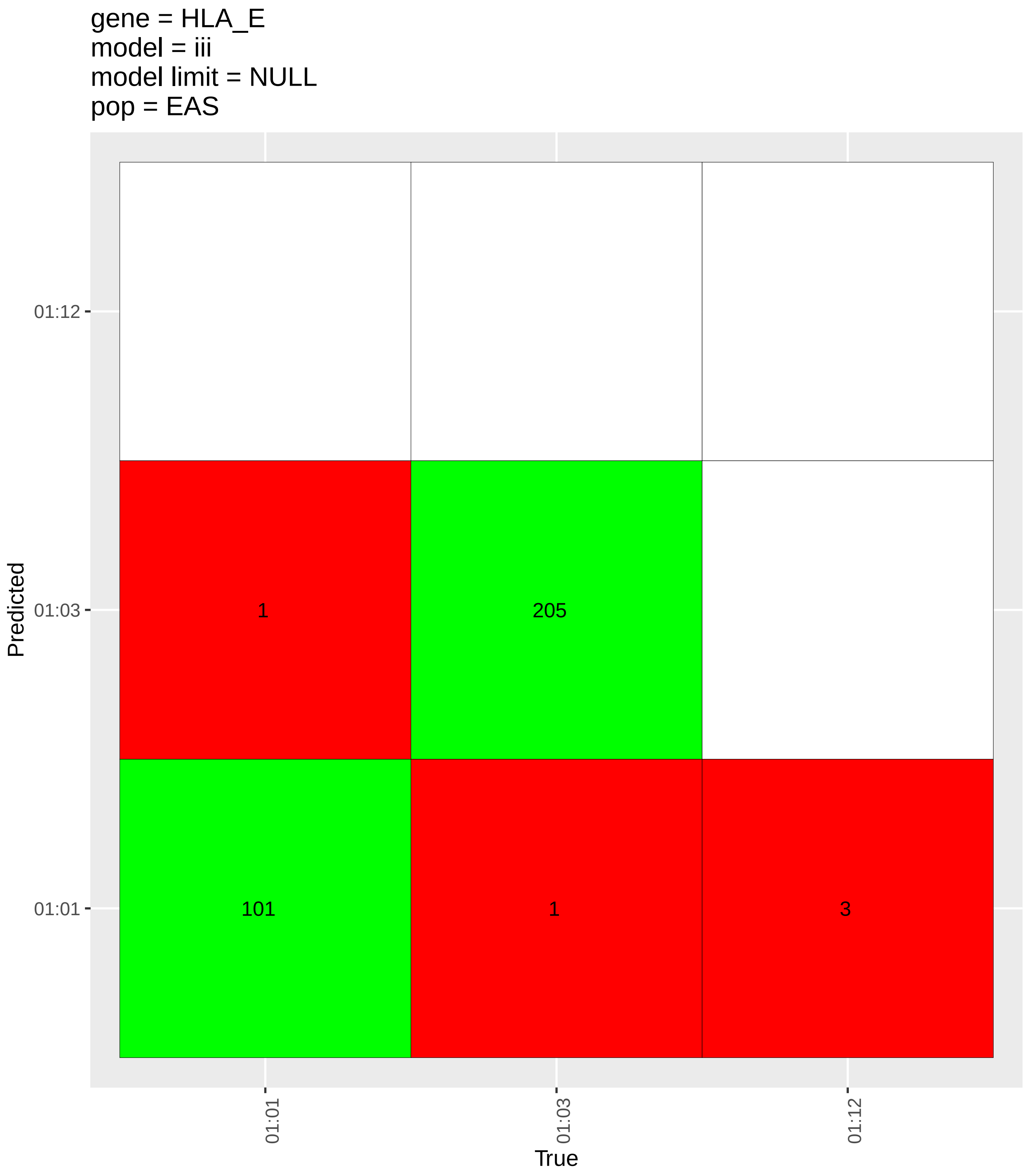
gene = HLA_E
model = iii
model limit = NULL
pop = EAS
Predicted
01:12
01:03
01:01
1
205
101
1
3
01:01
01:03
01:12
True

gene = HLA_E
model = iii
model limit = NULL
pop = SAS

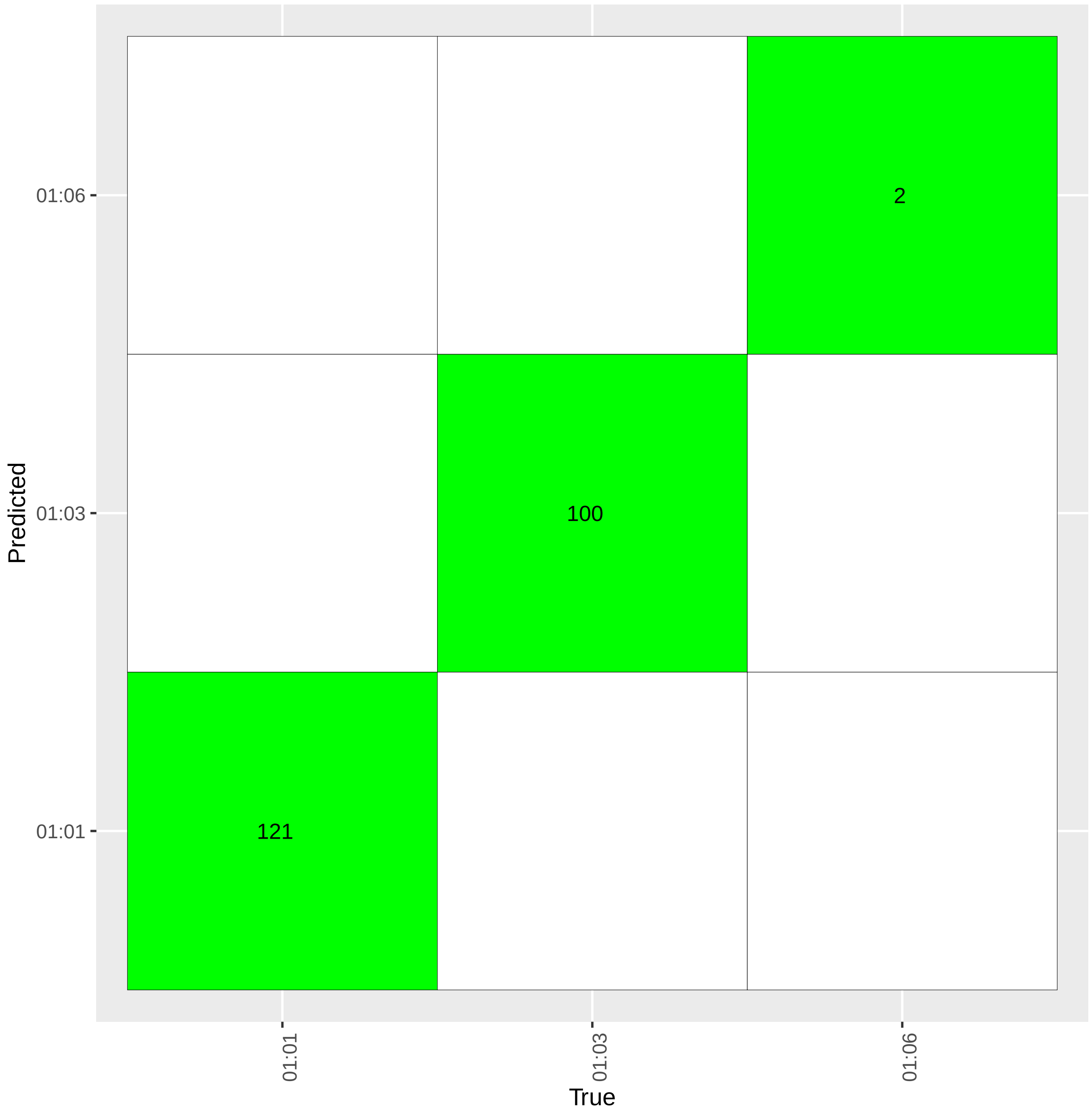

gene = HLA_E
model = iii
model limit = NULL
pop = AMR

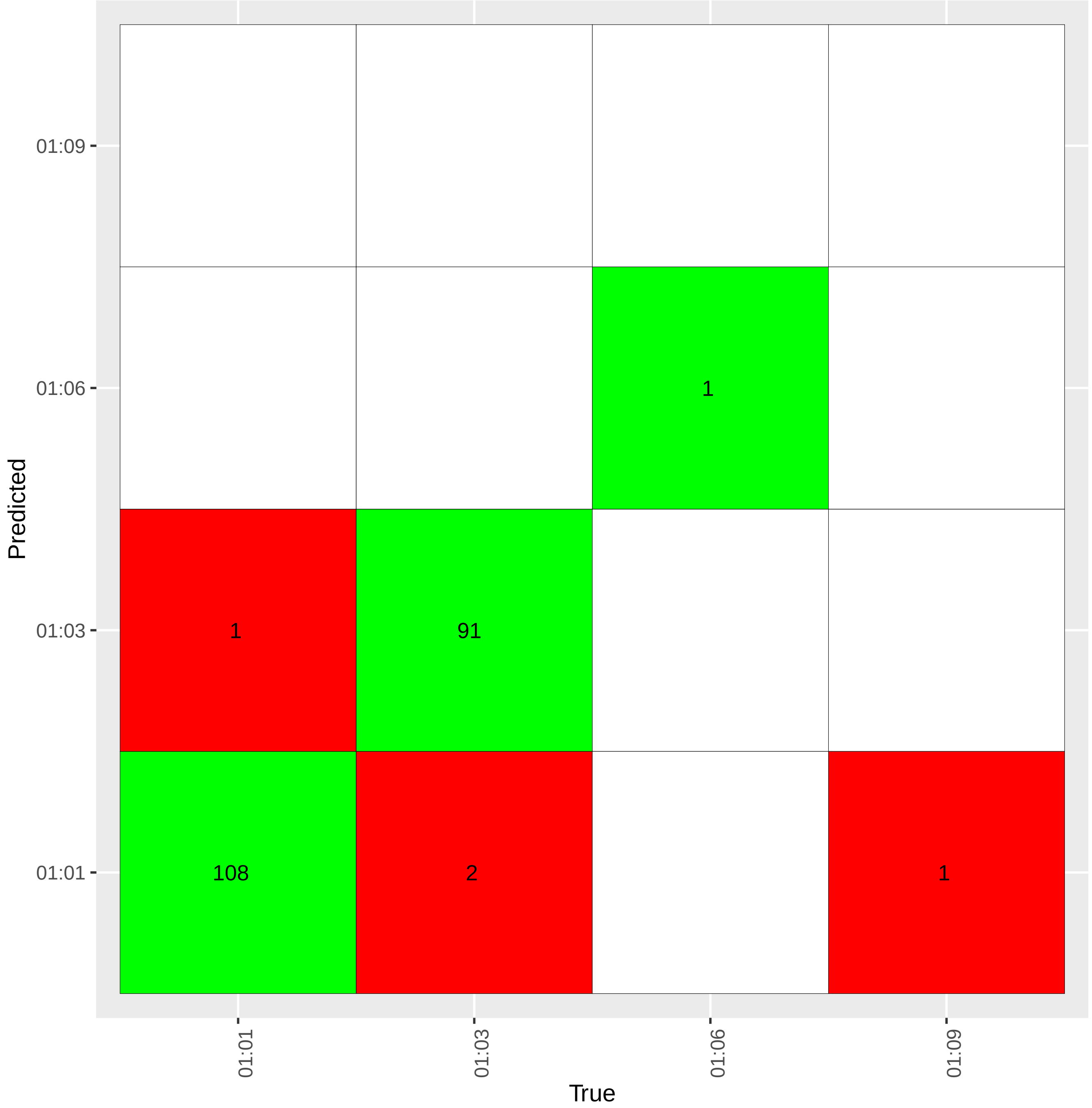

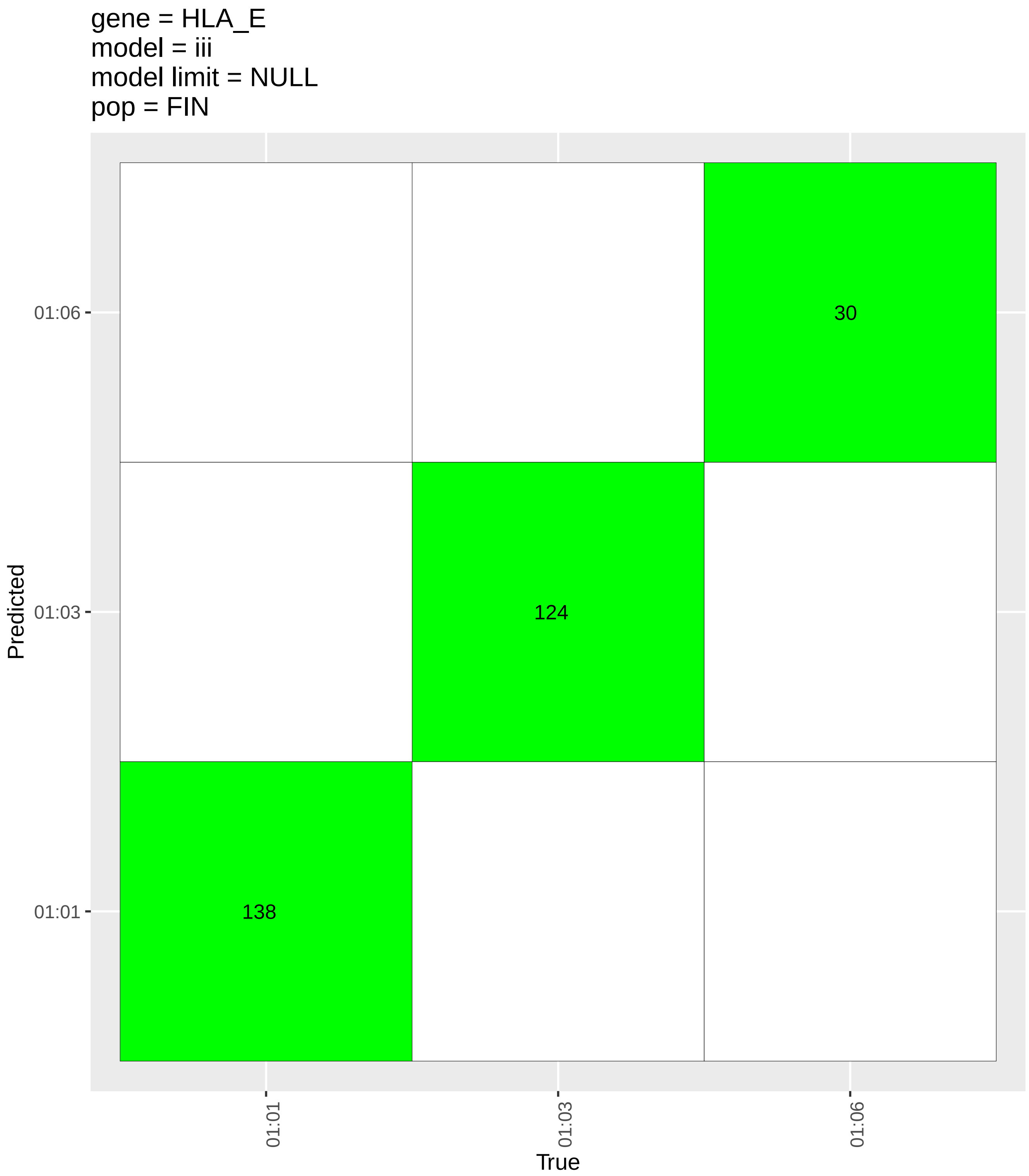
gene = HLA_E
model = iii
model limit = NULL
pop = FIN
30
124
138
01:06
01:03
01:01
Predicted
01:01
01:03
01:06
True

gene = HLA_E
model = iv
model limit = NULL
pop = EUR

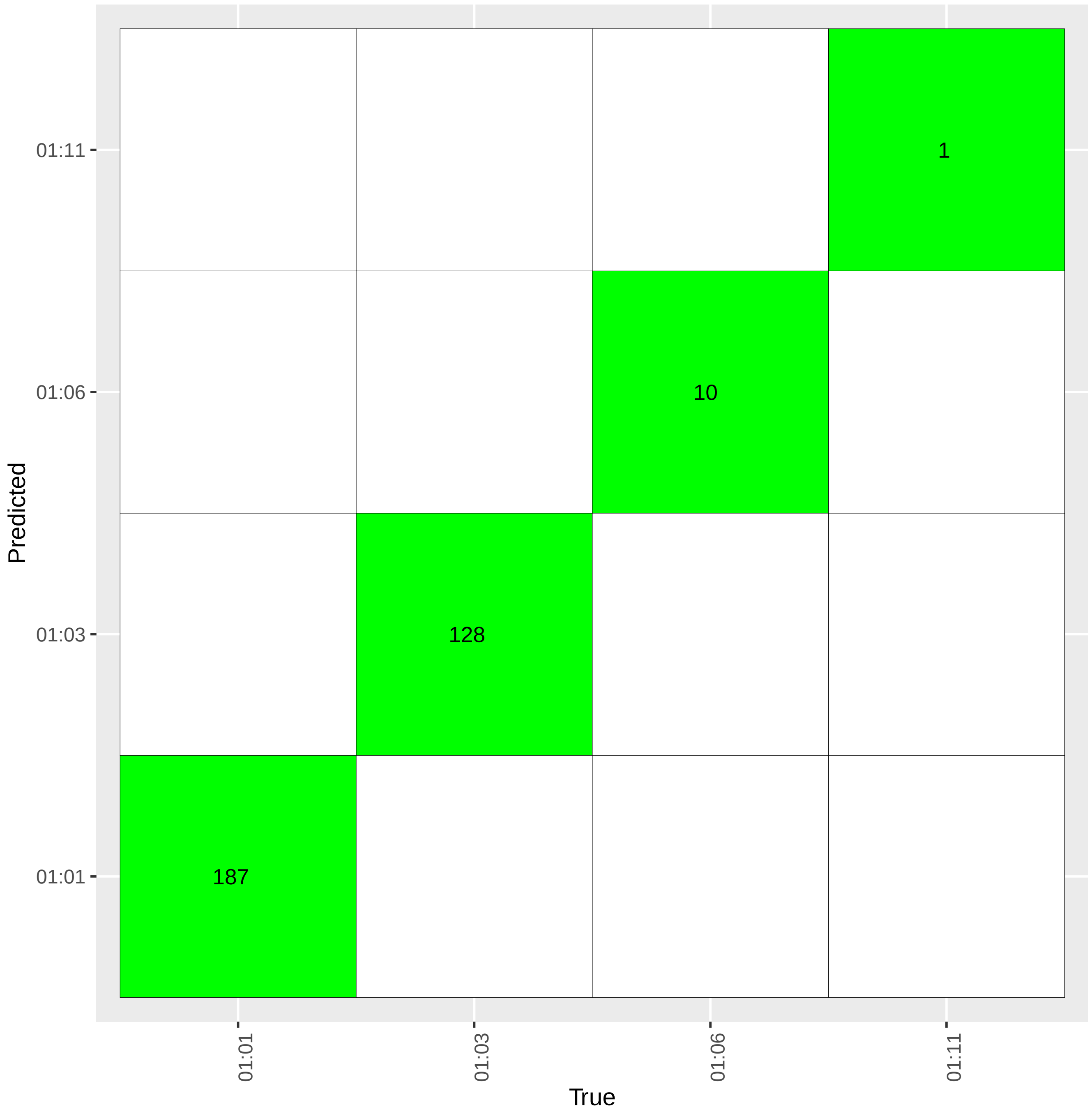

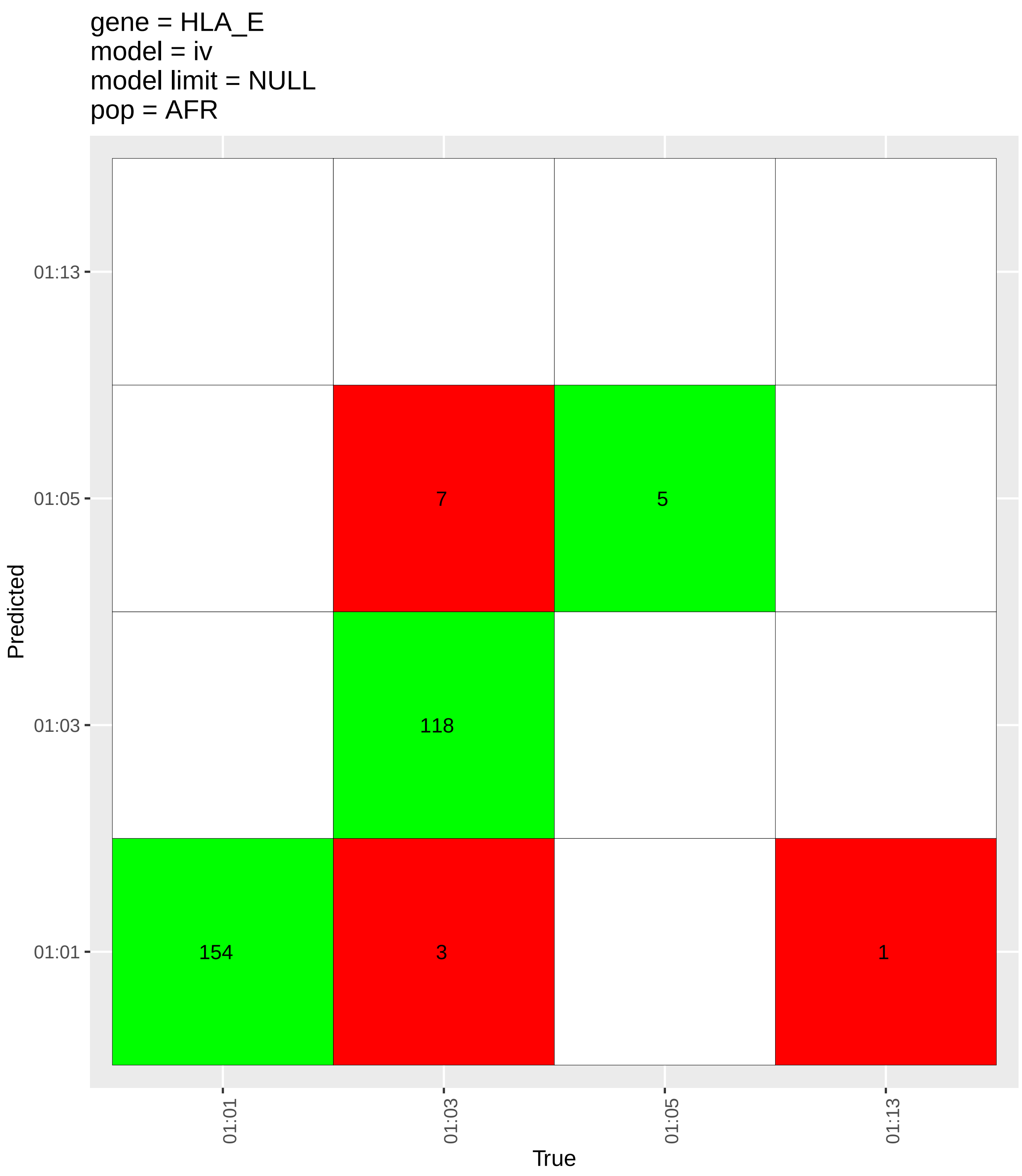
gene = HLA_E
model = iv
model limit = NULL
pop = AFR
Predicted
01:13
01:05
01:03
01:01
7
5
118
154
3
1
01:01
01:03
01:05
01:13
True

gene = HLA_E
model = iv
model limit = NULL
pop = EAS

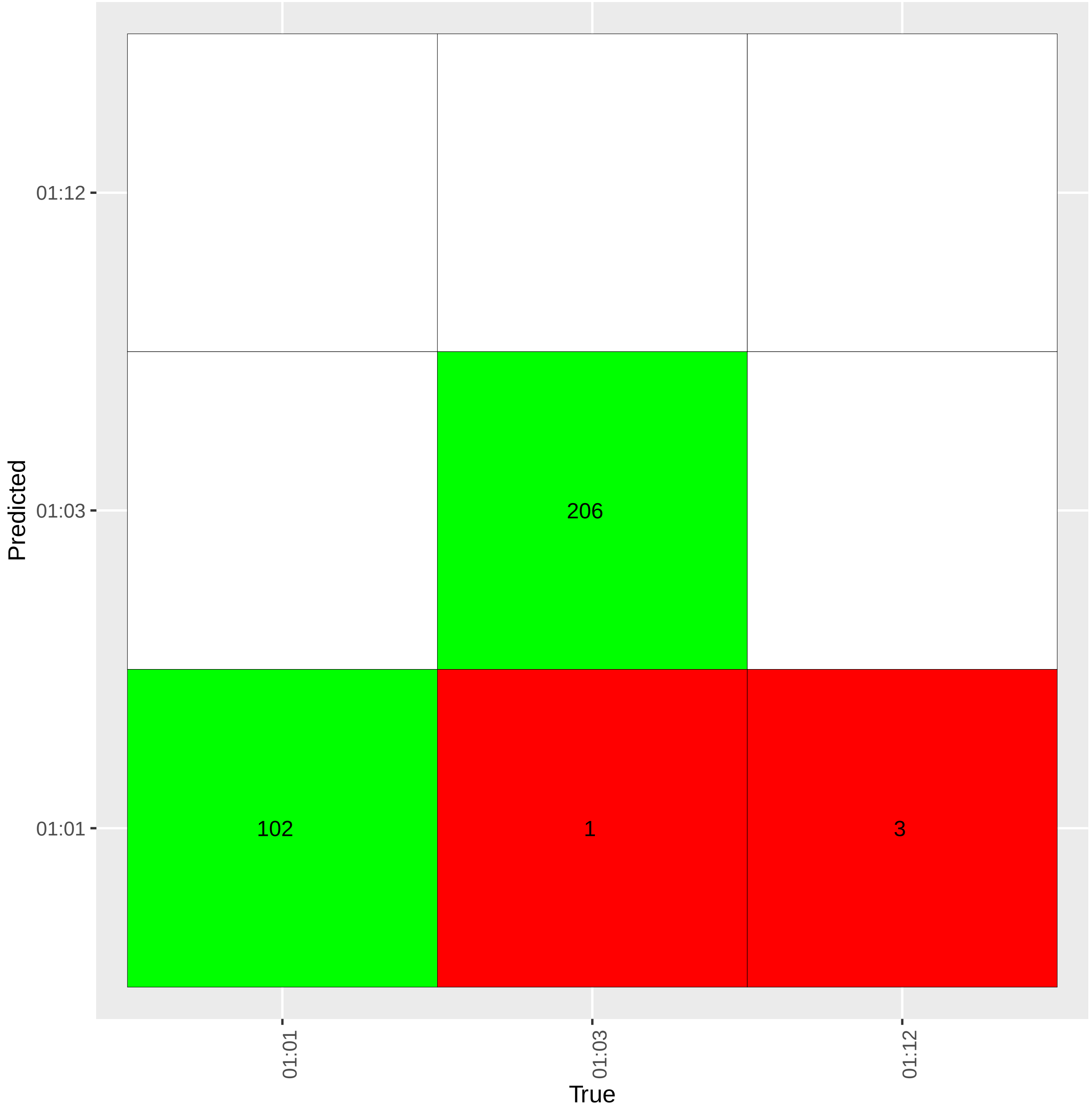

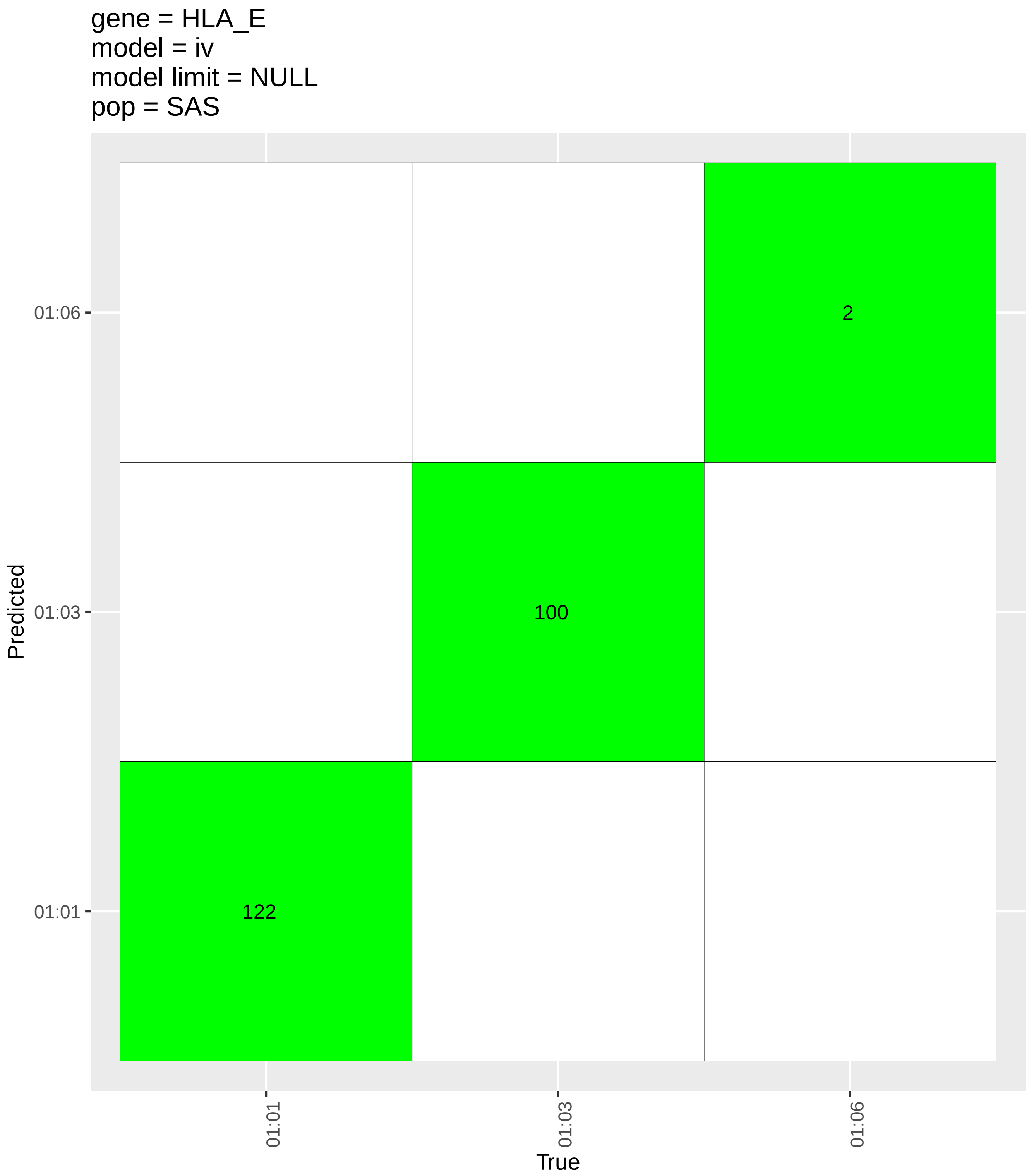
gene = HLA_E
model = iv
model limit = NULL
pop = SAS
Predicted
01:06
01:03
01:01
2
100
122
01:01
01:03
01:06
True

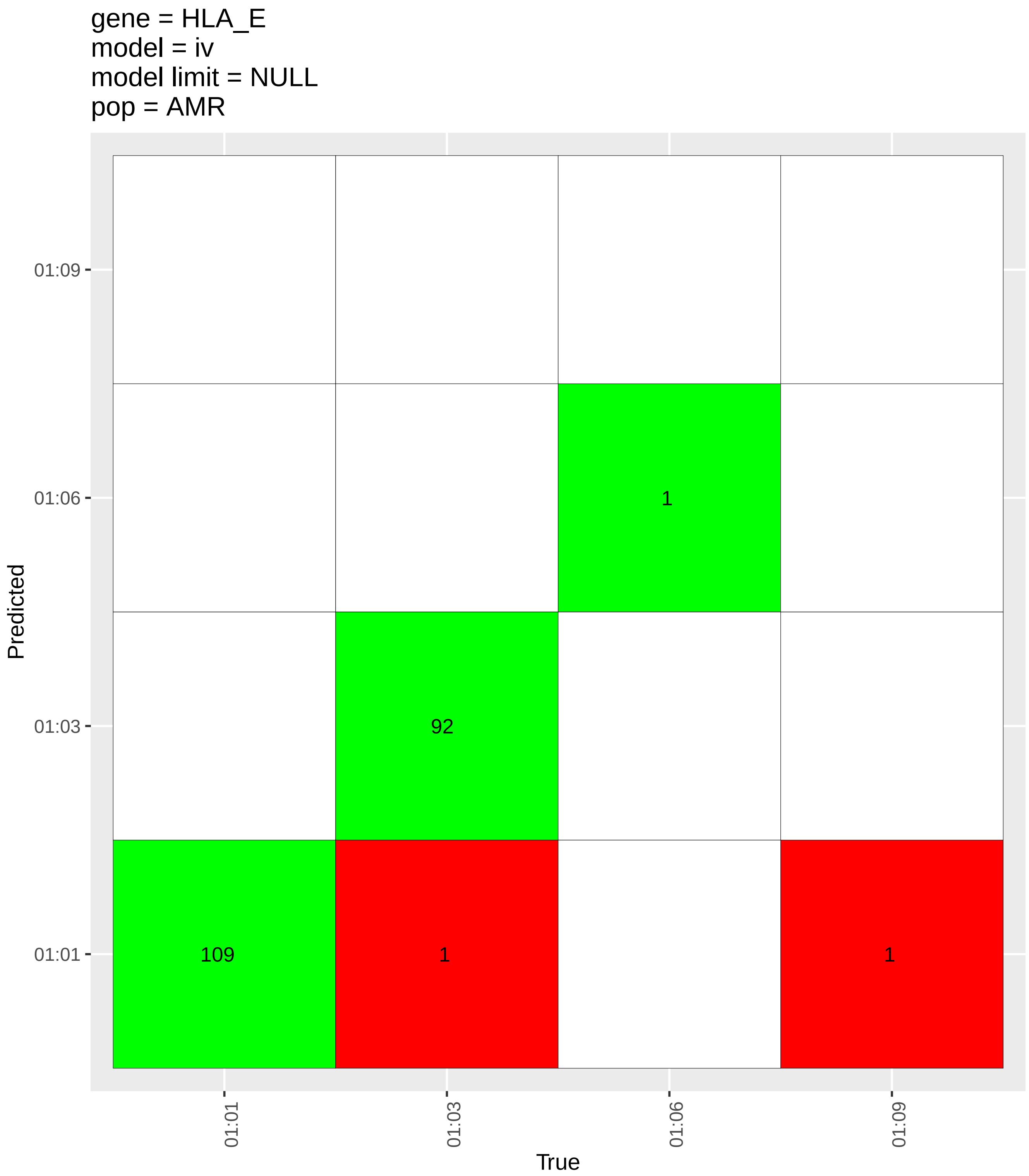
gene = HLA_E
model = iv
model limit = NULL
pop = AMR
Predicted
01:09
01:06
01:03
01:01
1
92
109
1
1
01:01
01:03
01:06
01:09
True

gene = HLA_E
model = iv
model limit = NULL
pop = FIN

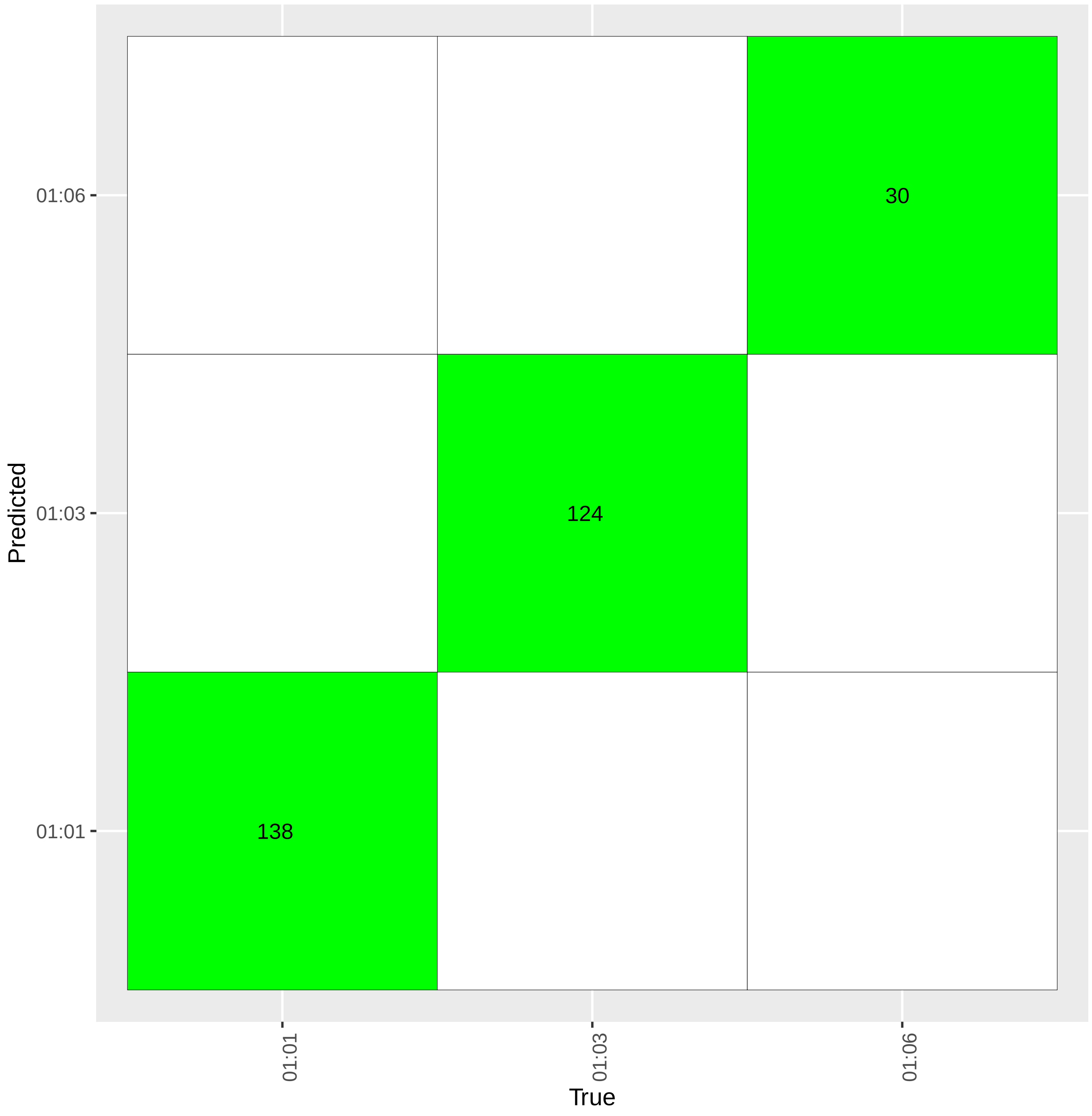

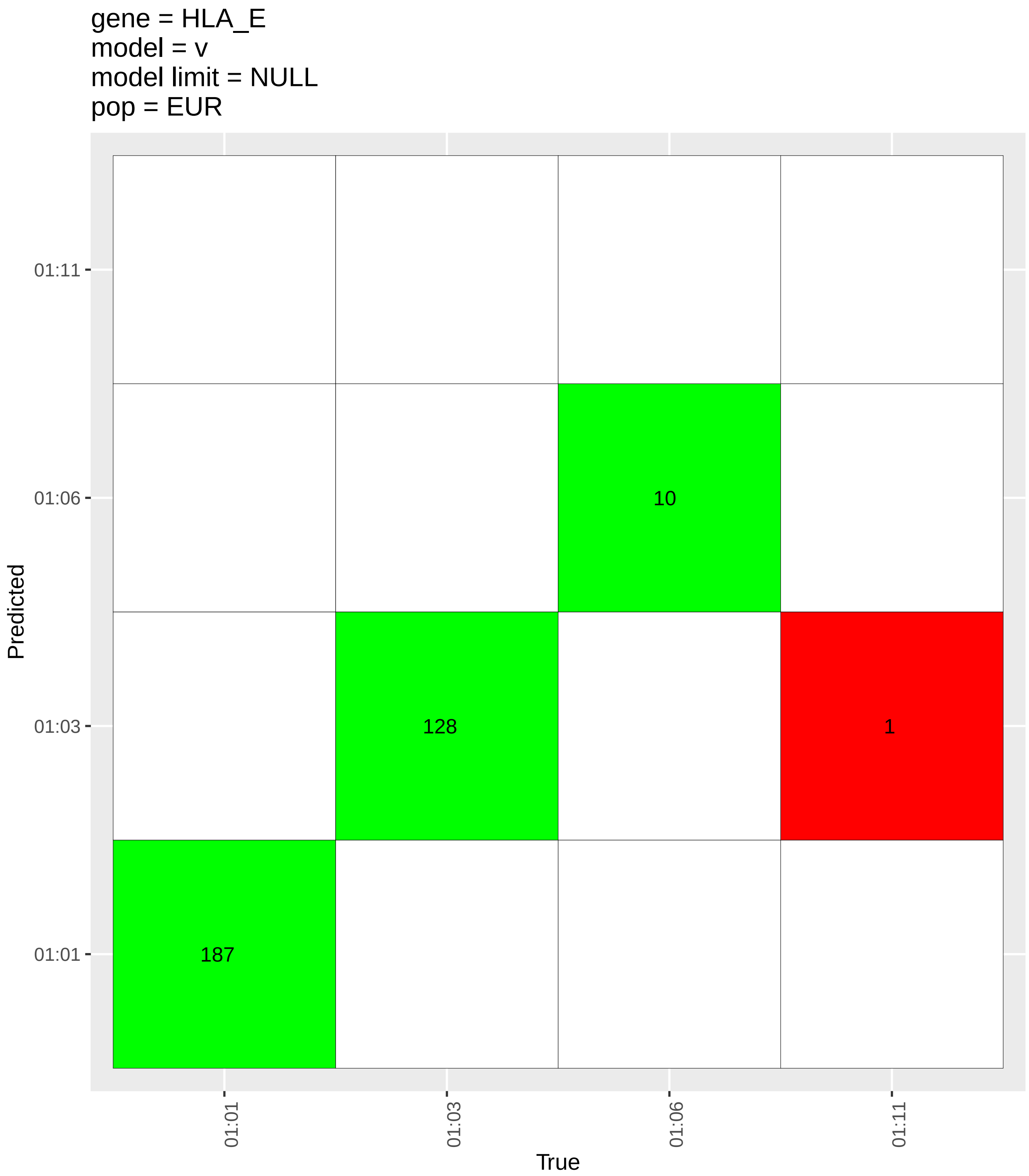
gene = HLA_E
model = v
model limit = NULL
pop = EUR
Predicted
01:11
01:06
01:03
01:01
10
128
1
187
01:01
01:03
01:06
01:11
True

gene = HLA_E
model = v
model limit = NULL
pop = AFR

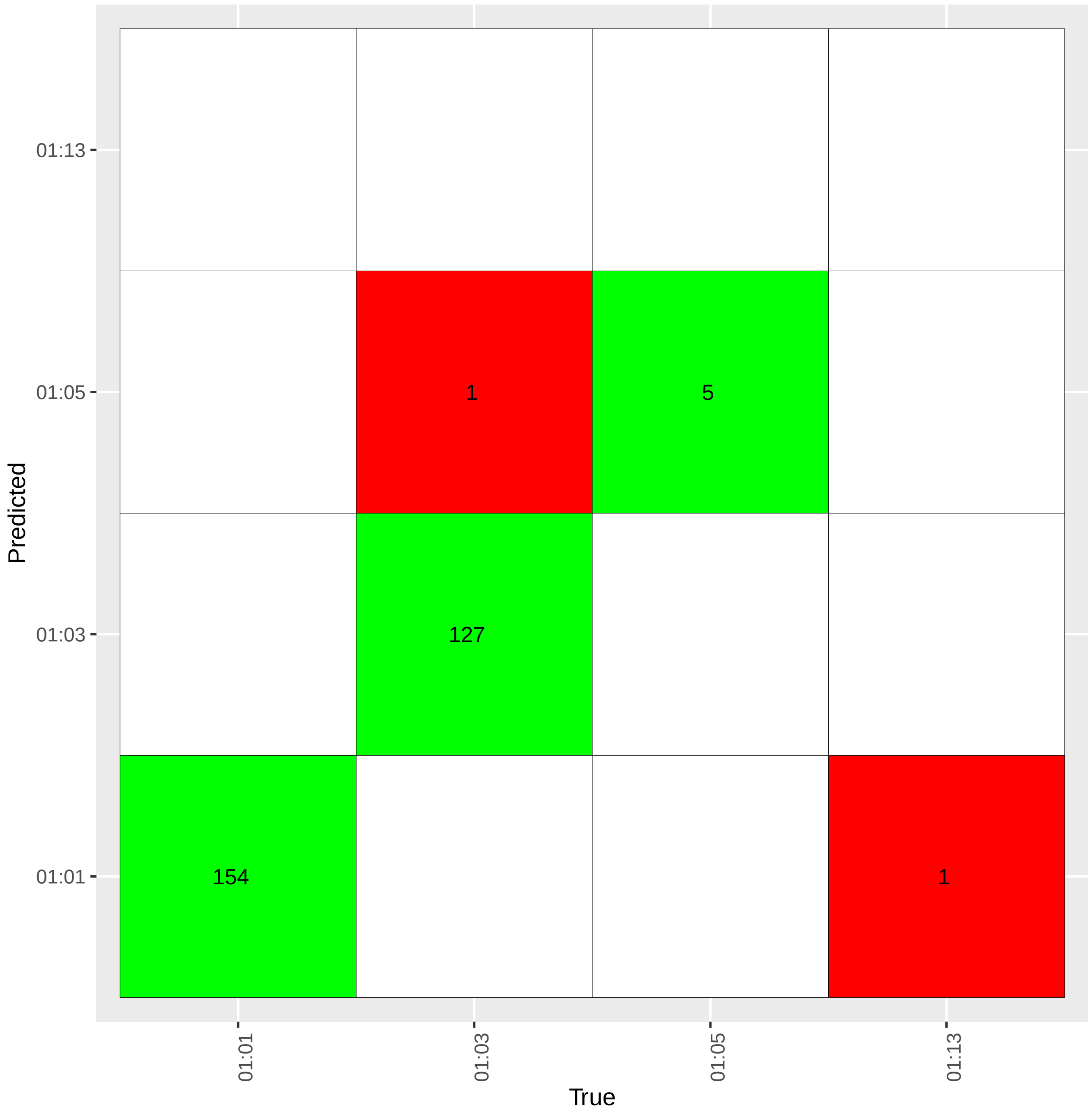

gene = HLA_E
model = v
model limit = NULL
pop = EAS

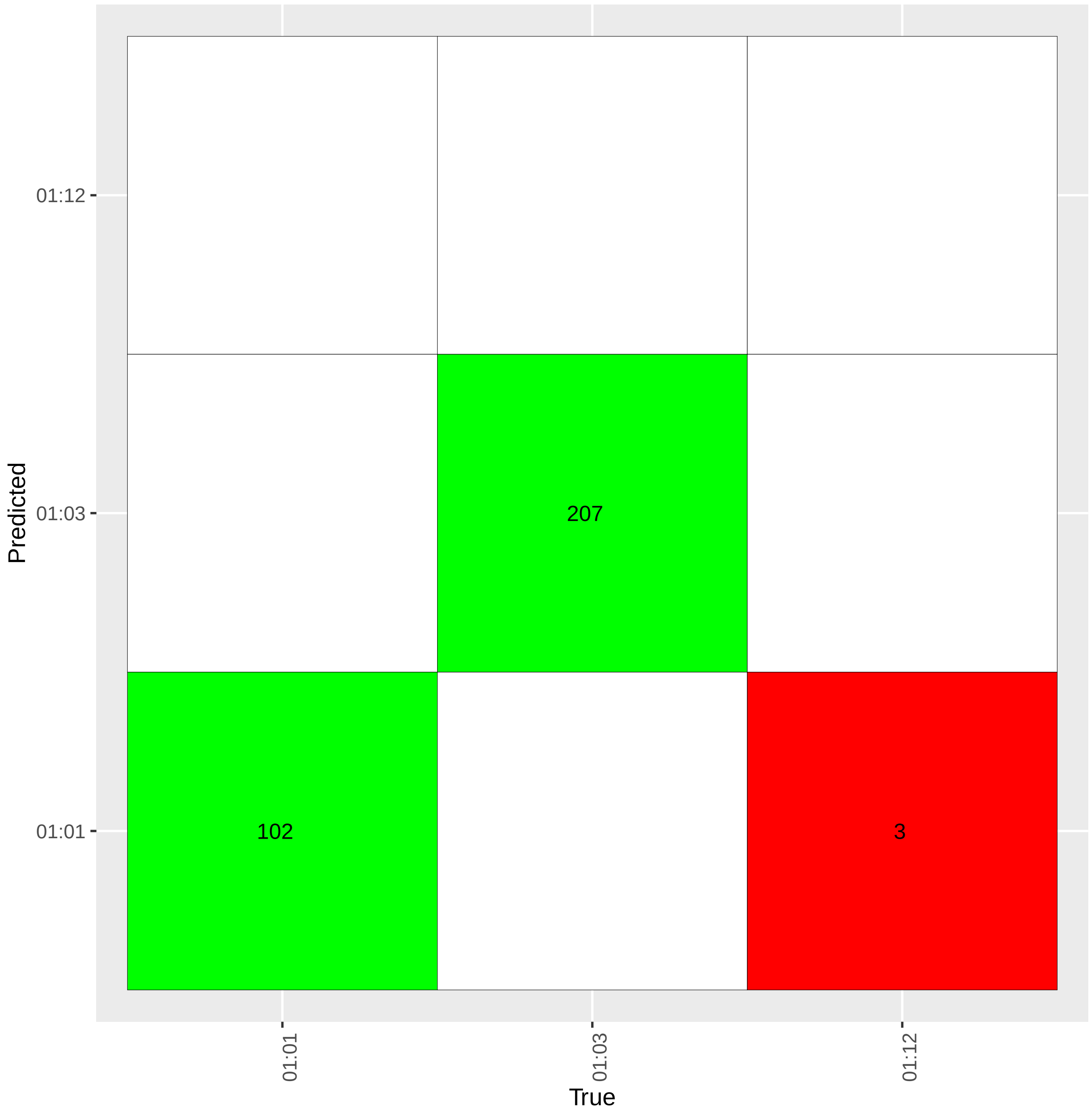

gene = HLA_E
model = v
model limit = NULL
pop = SAS

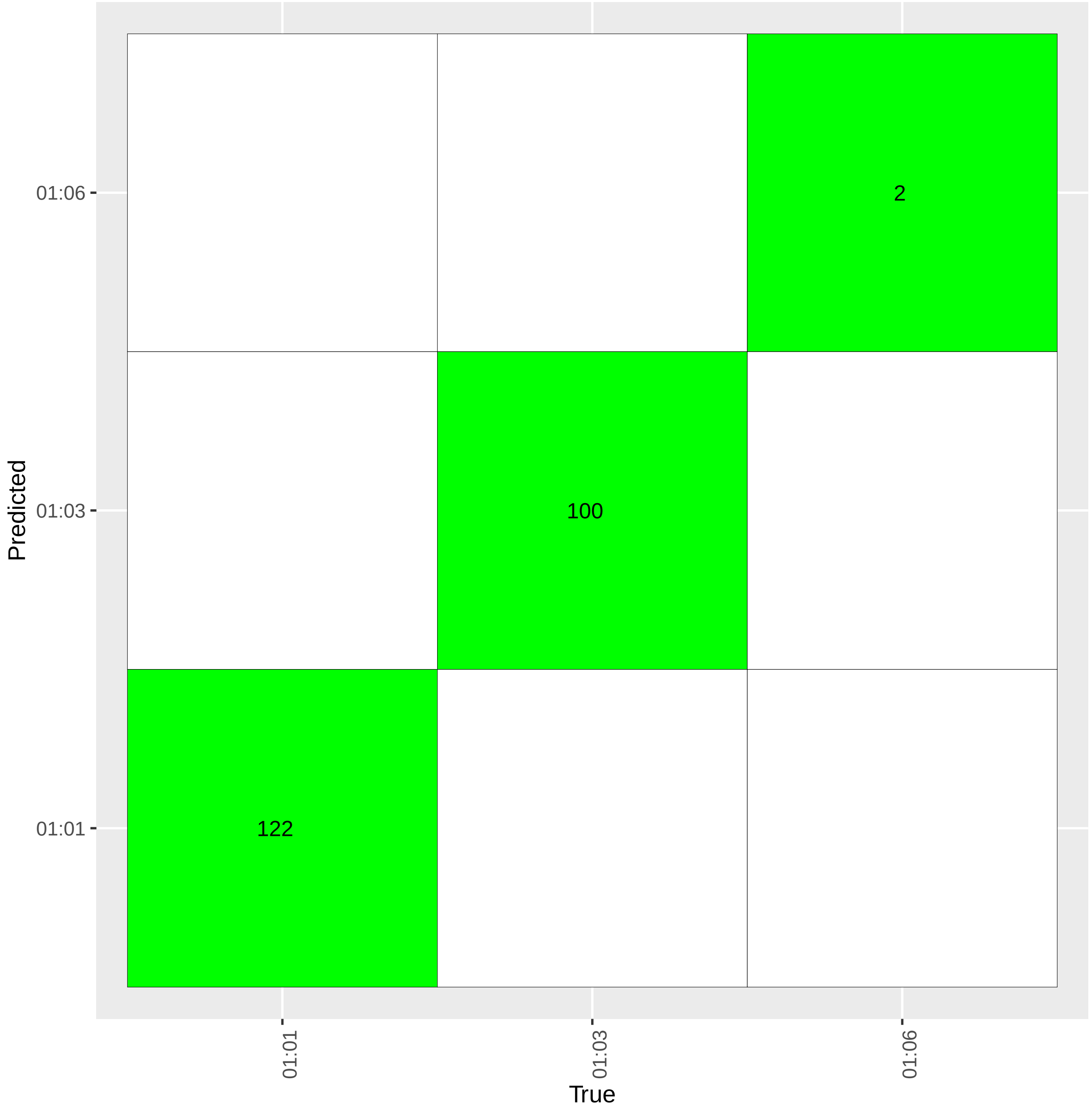

gene = HLA_E
model = v
model limit = NULL
pop = AMR

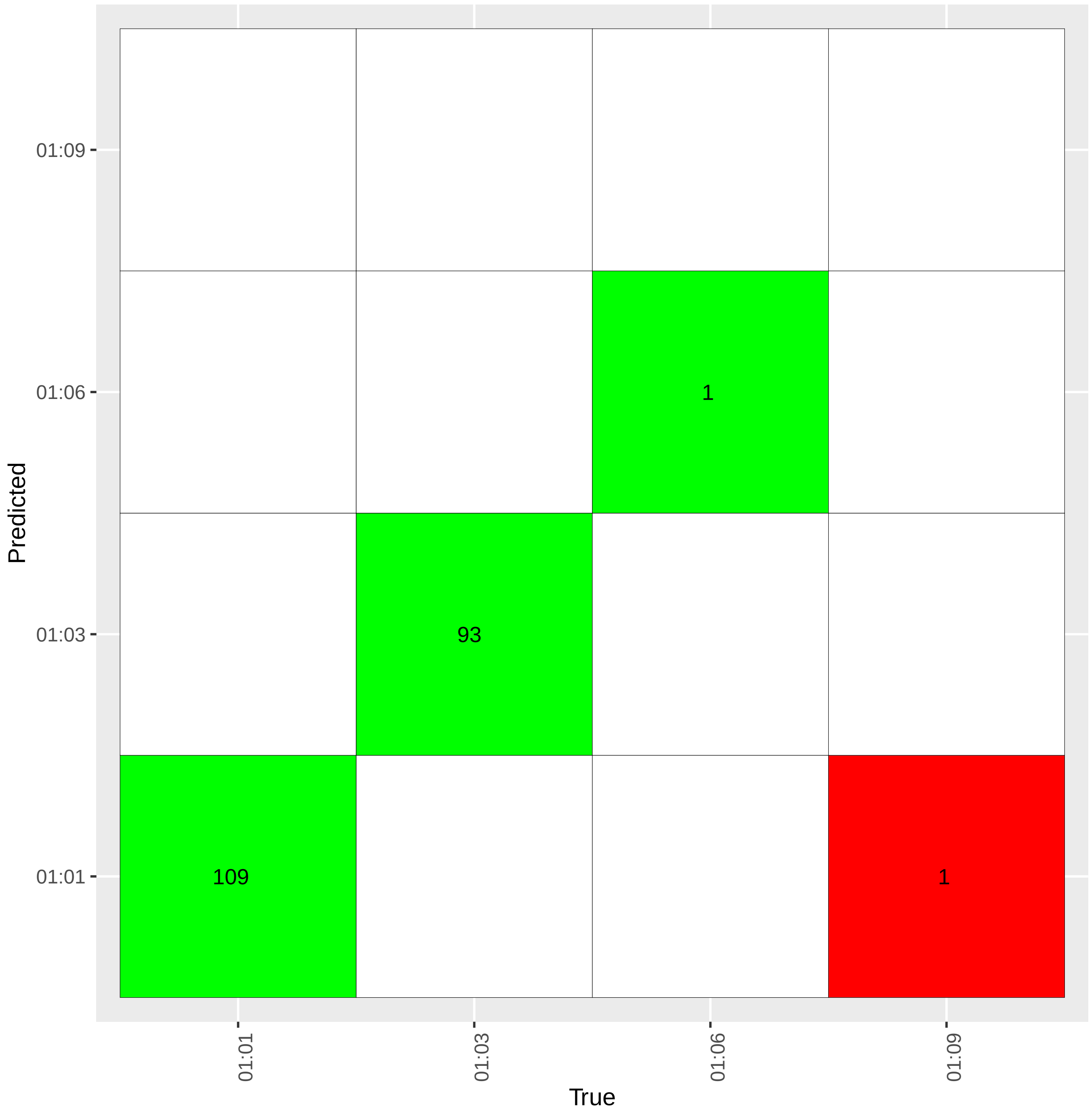

gene = HLA_E
model = v
model limit = NULL
pop = FIN

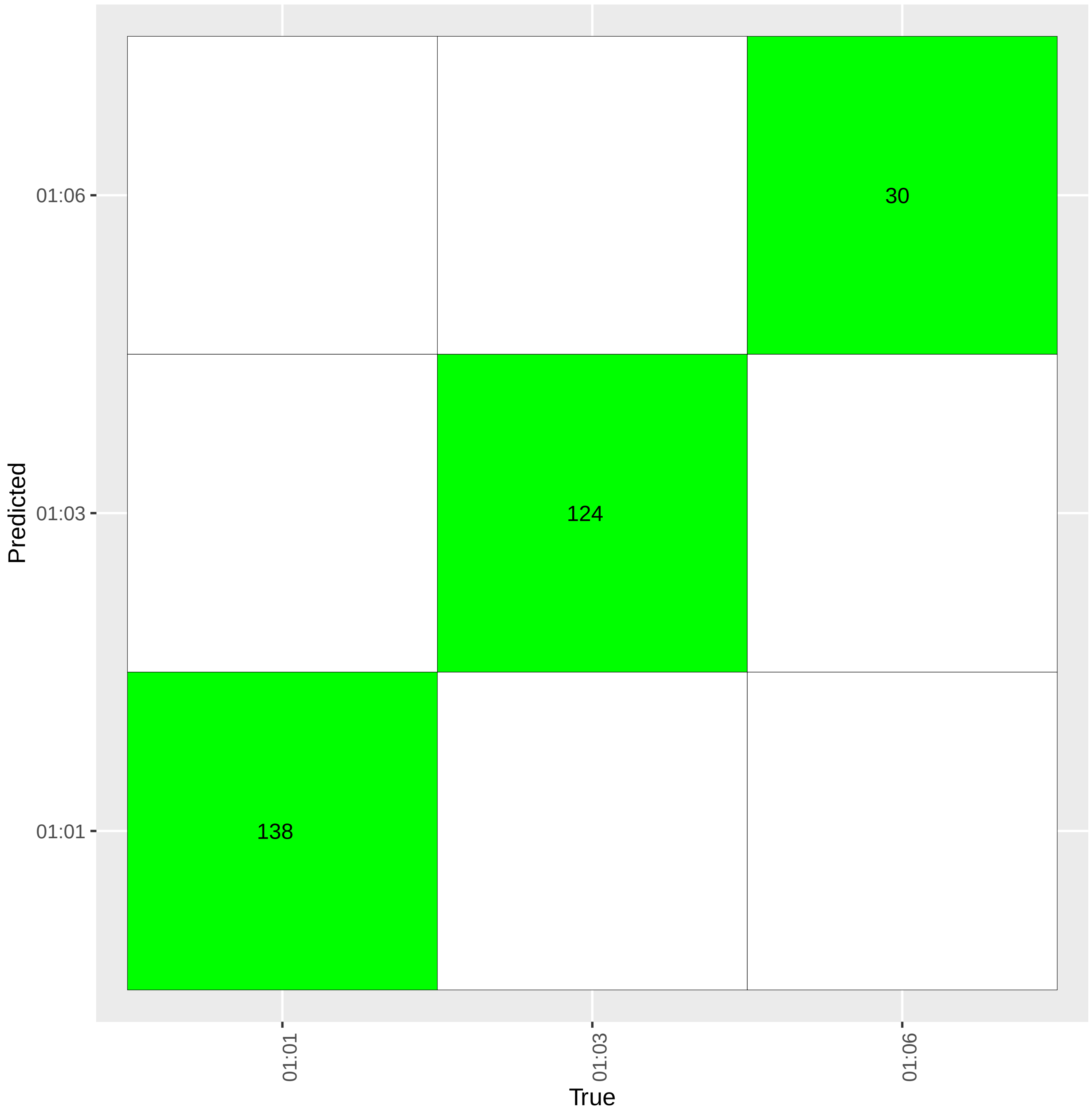

gene = HLA_E
model = vi
model limit = NULL
pop = EUR

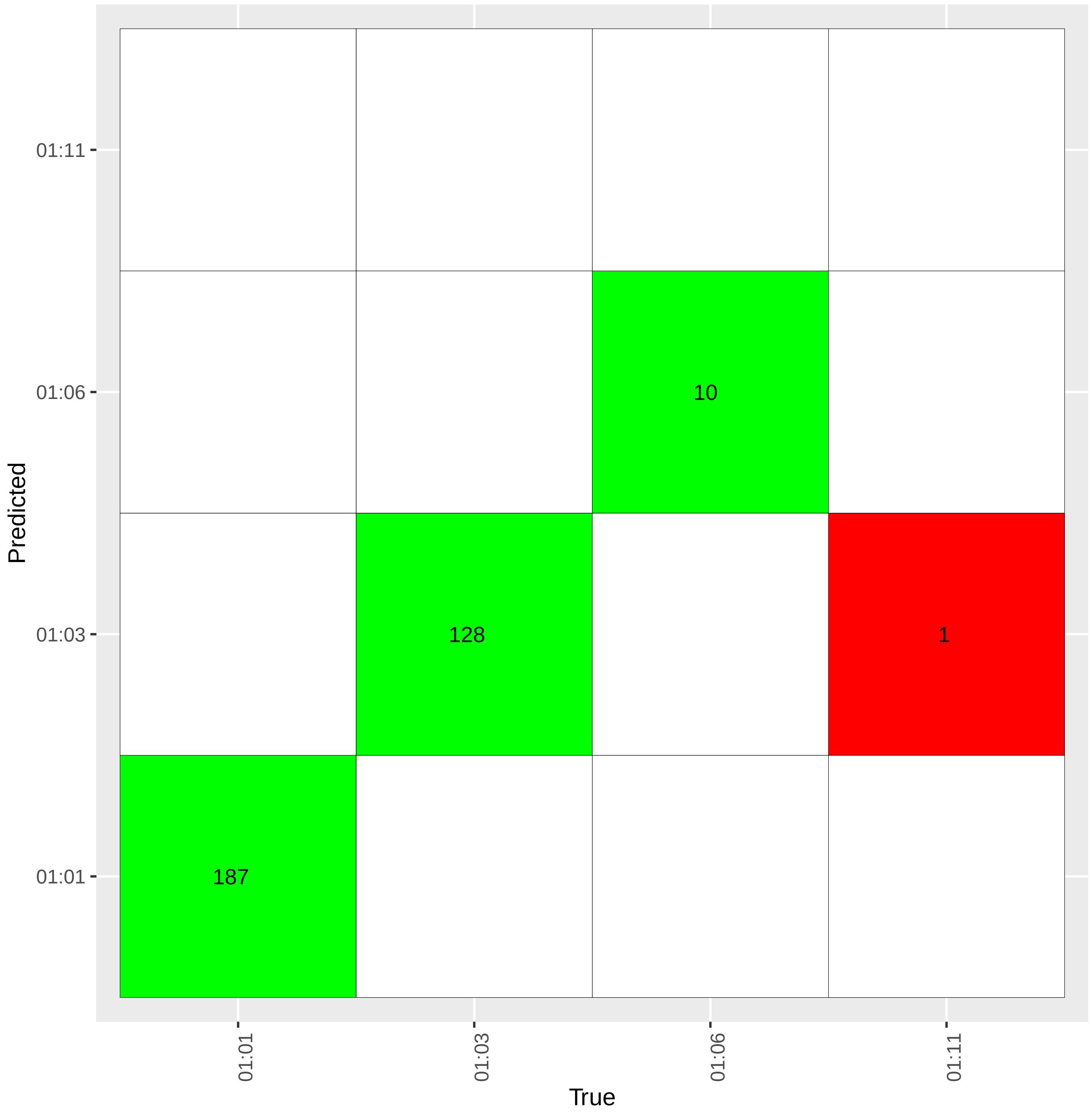

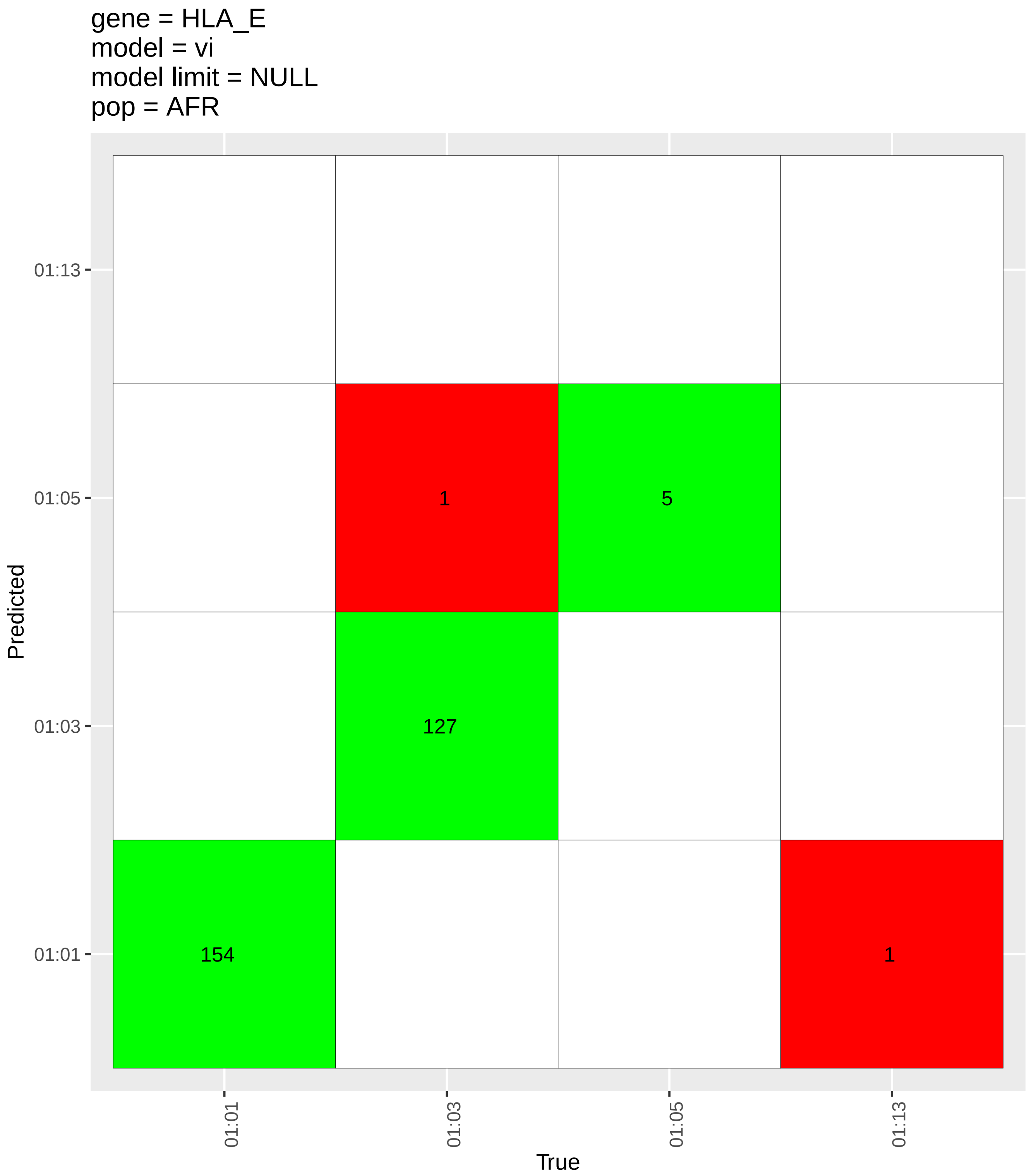
gene = HLA_E
model = vi
model limit = NULL
pop = AFR
Predicted
01:13
01:05
01:03
01:01
1
5
127
154
1
01:01
01:03
01:05
01:13
True

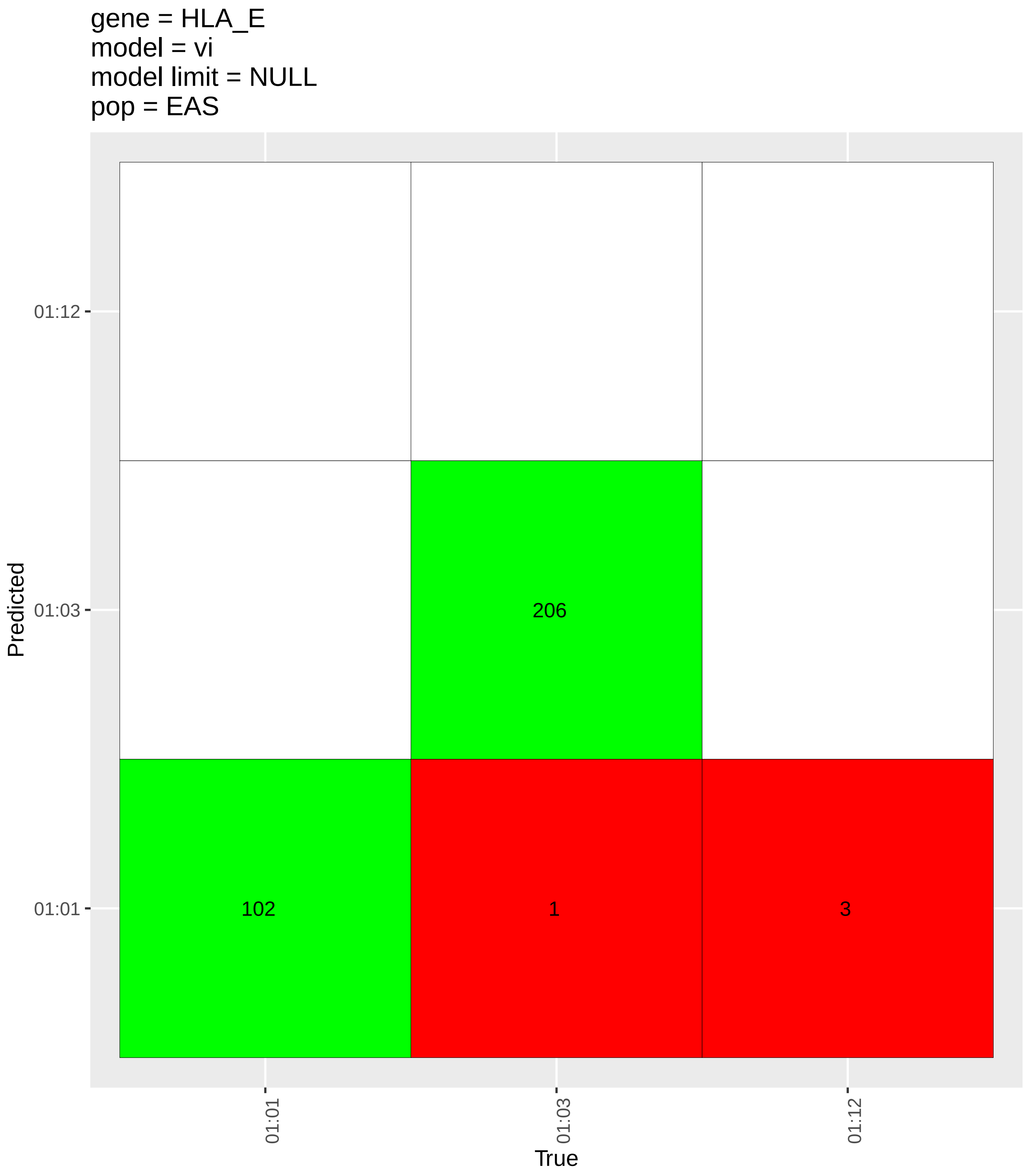
gene = HLA_E
model = vi
model limit = NULL
pop = EAS
Predicted
01:12
01:03
01:01
206
102
1
3
01:01
01:03
01:12
True

gene = HLA_E
model = vi
model limit = NULL
pop = SAS

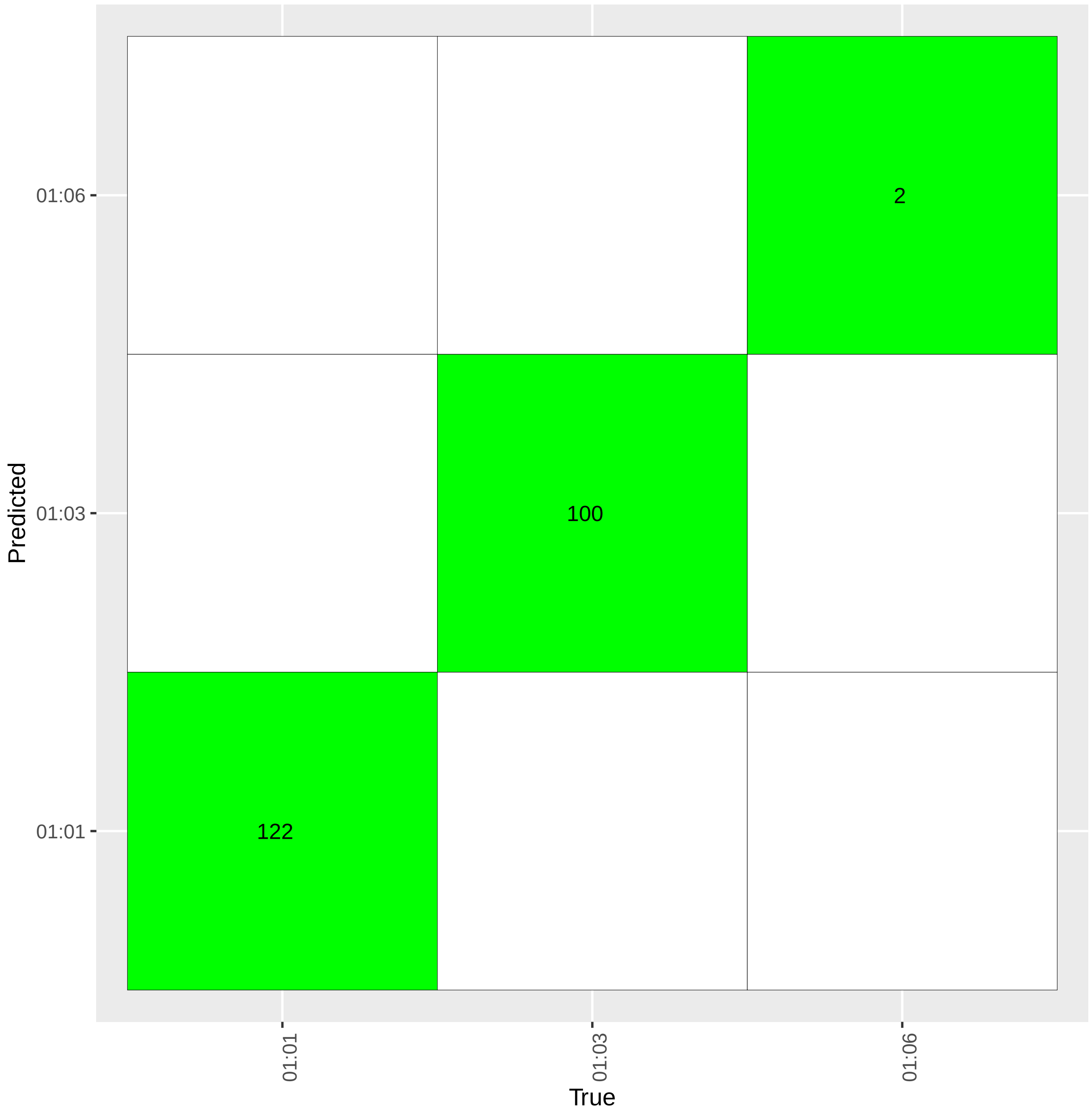

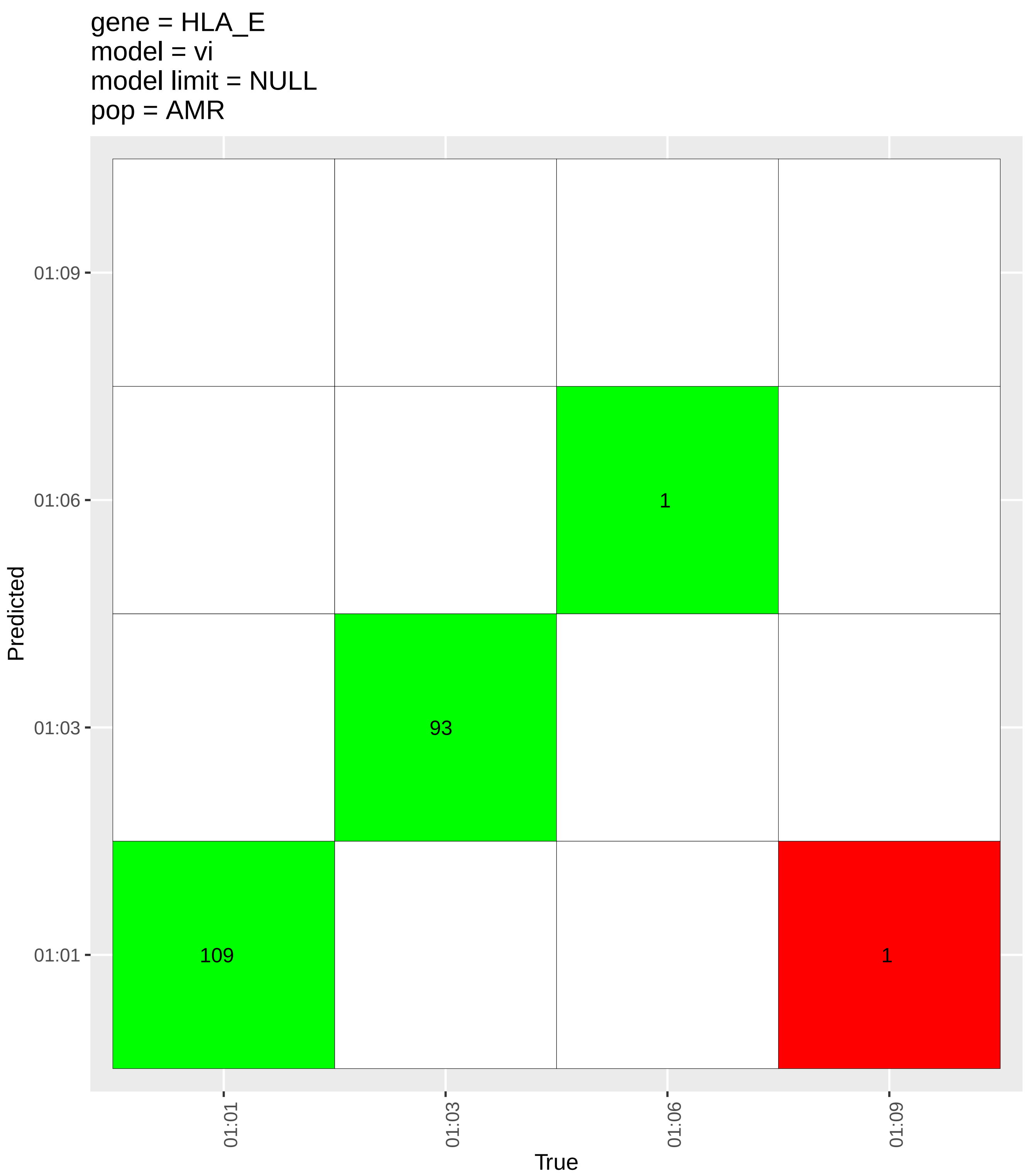

gene = HLA_E
model = vi
model limit = NULL
pop = AMR
Predicted
01:09
01:06
01:03
01:01
1
93
109
1
01:01
01:03
01:06
01:09
True

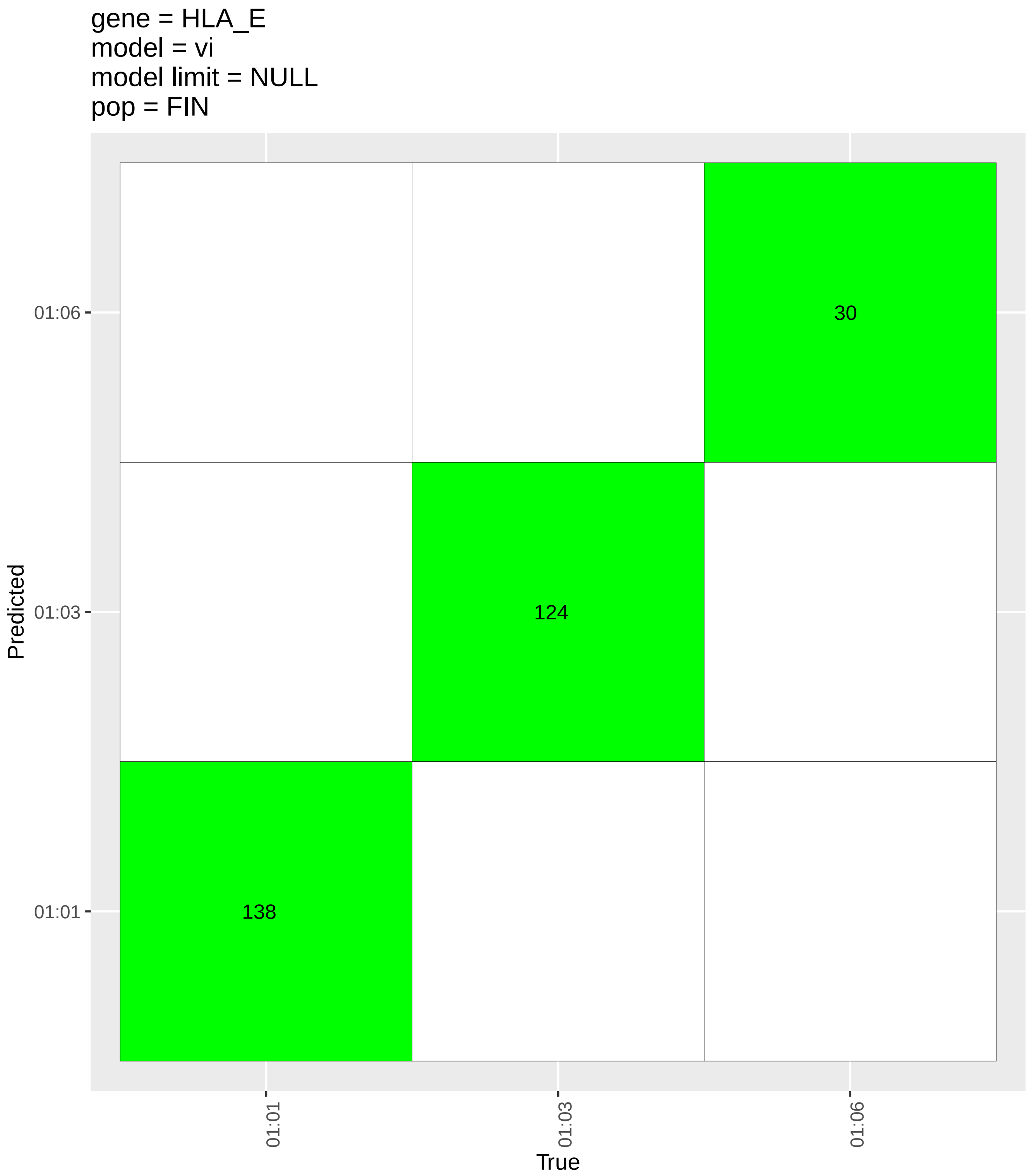
gene = HLA_E
model = vi
model limit = NULL
pop = FIN
Predicted
01:06
01:03
01:01
30
124
138
01:01
01:03
01:06
True

gene = HLA_E
model = vii
model limit = NULL
pop = EUR

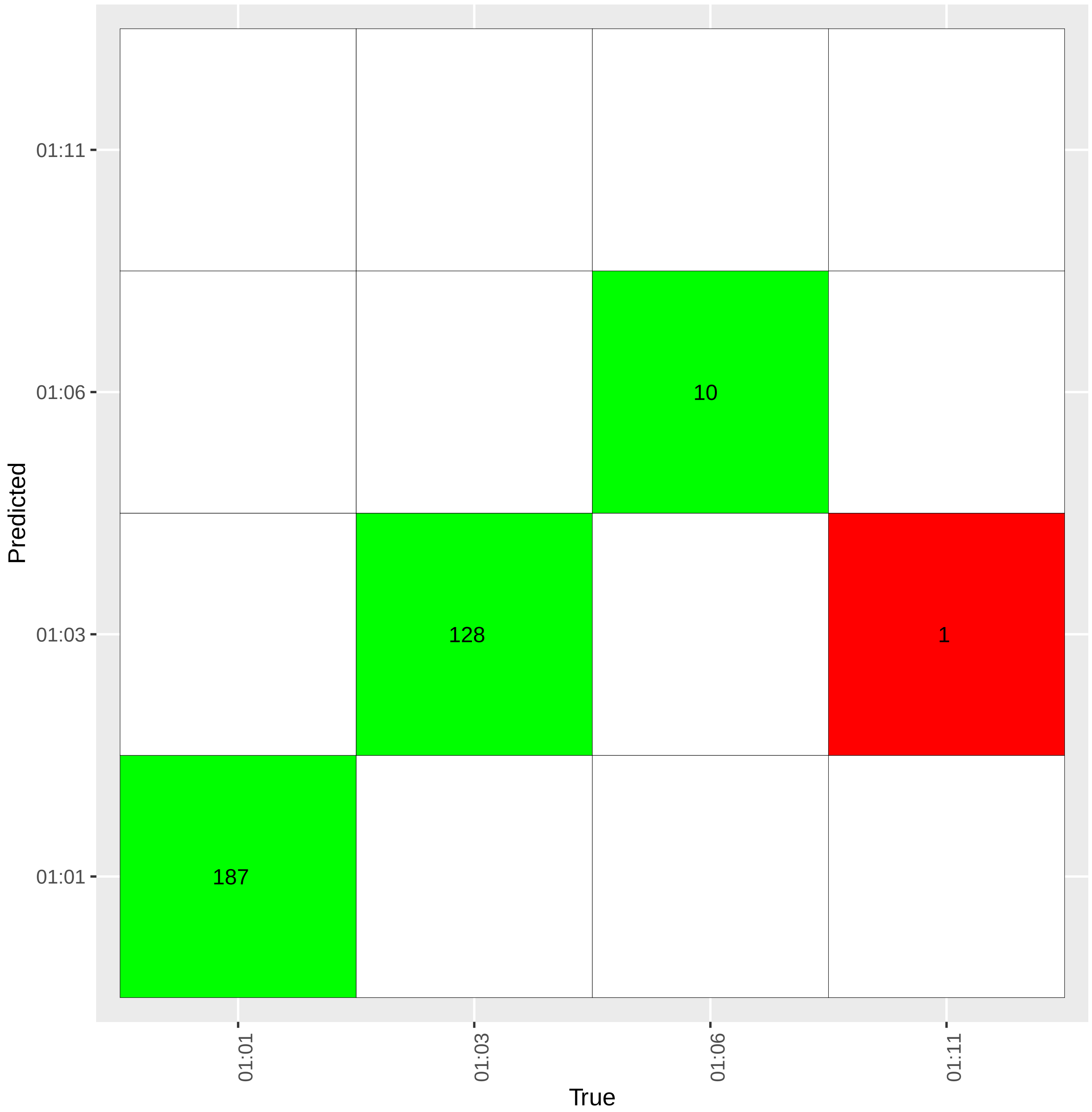

gene = HLA_E
model = vii
model limit = NULL
pop = AFR

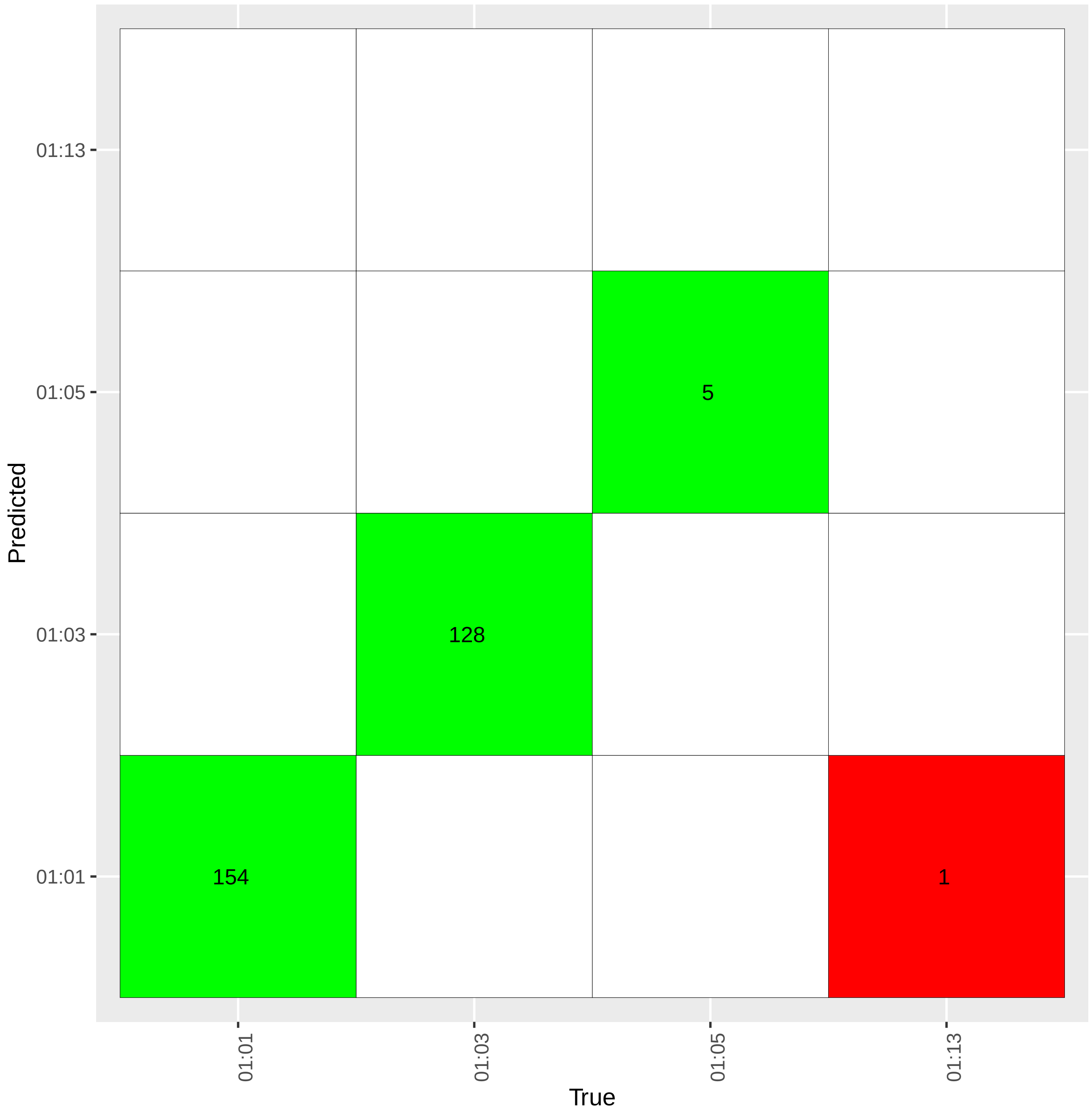

gene = HLA_E
model = vii
model limit = NULL
pop = EAS

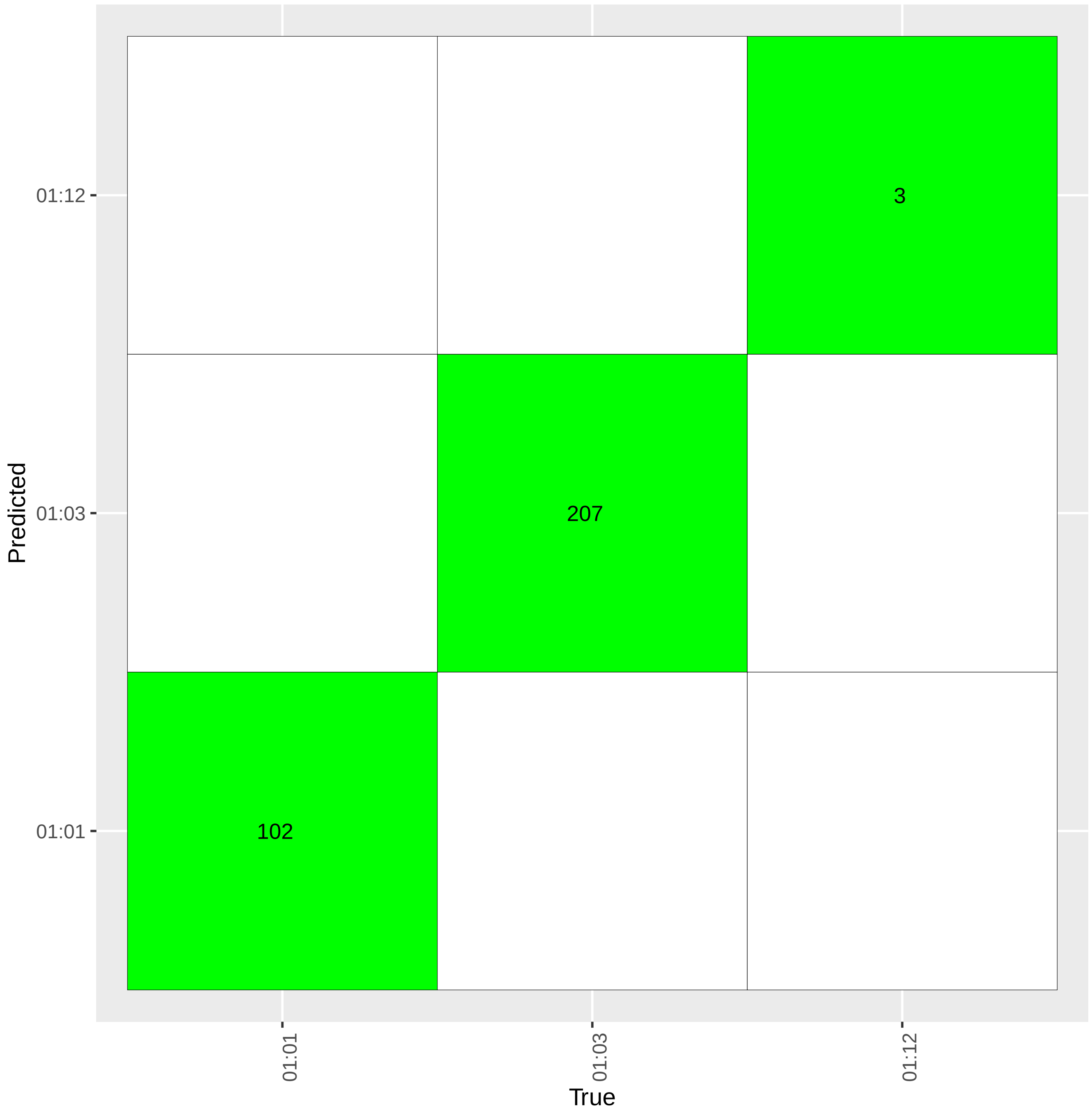

gene = HLA_E
model = vii
model limit = NULL
pop = SAS

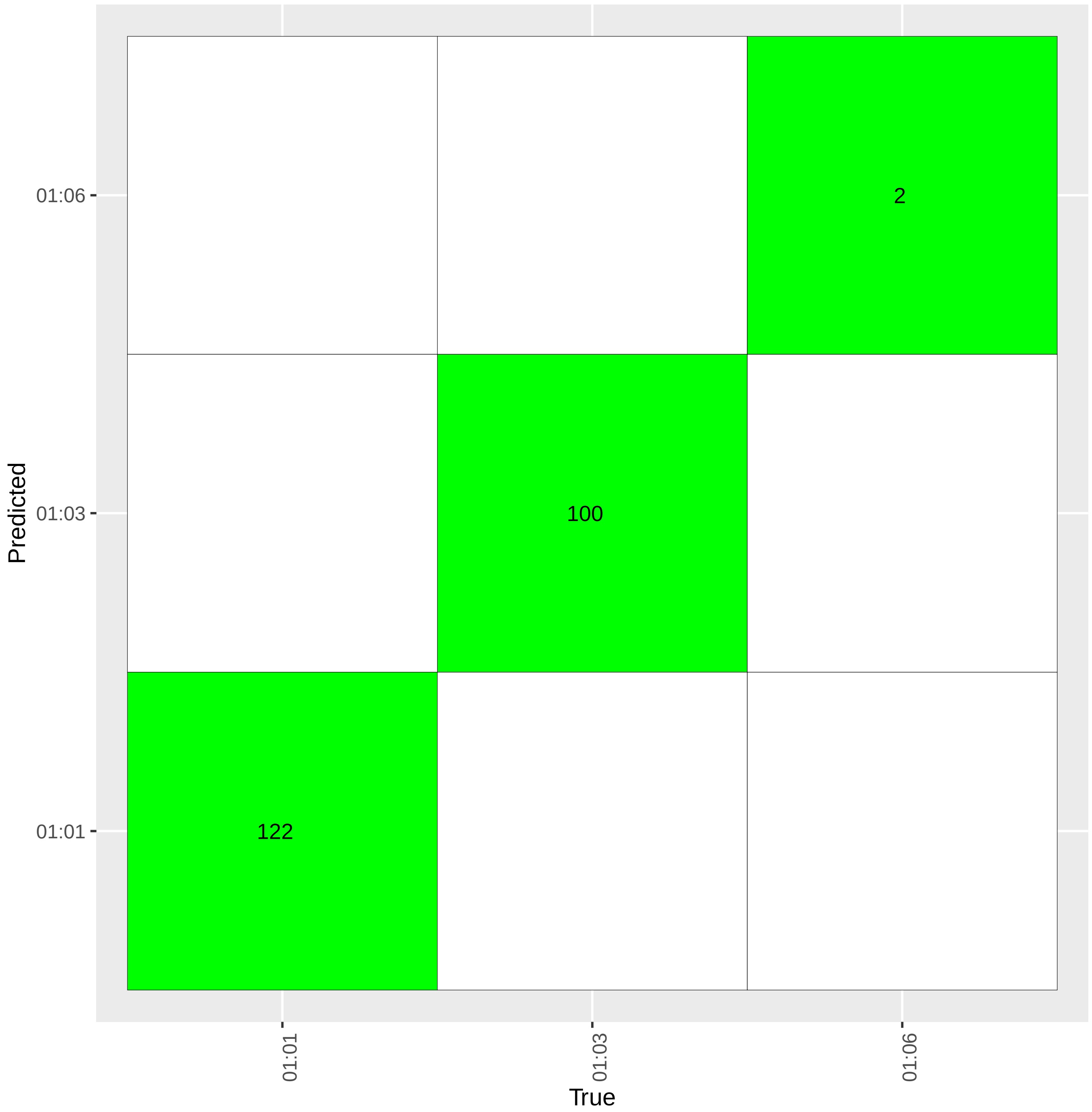

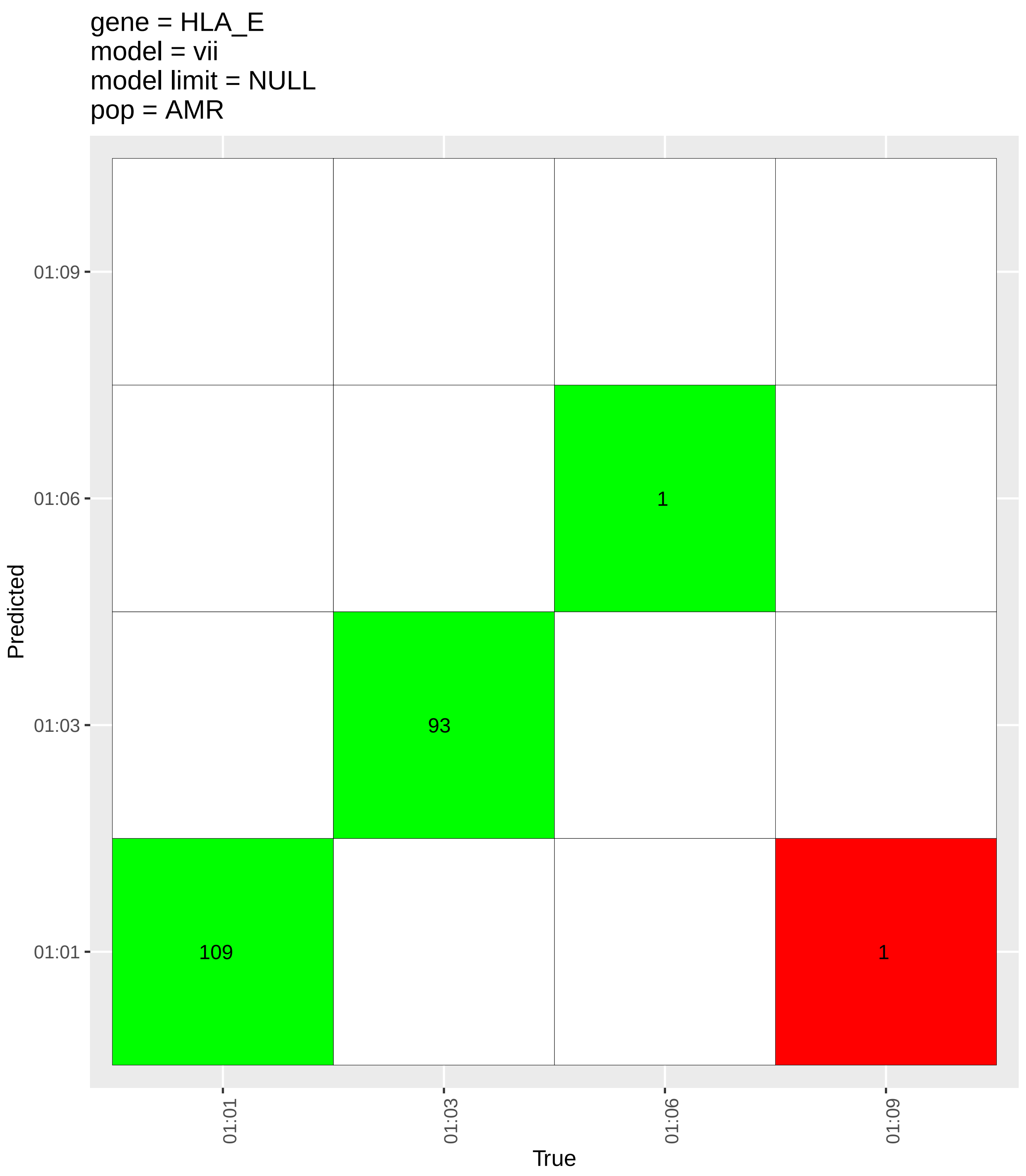
gene = HLA_E
model = vii
model limit = NULL
pop = AMR
Predicted
True
01:01
01:03
01:06
01:09
109
93
1
1

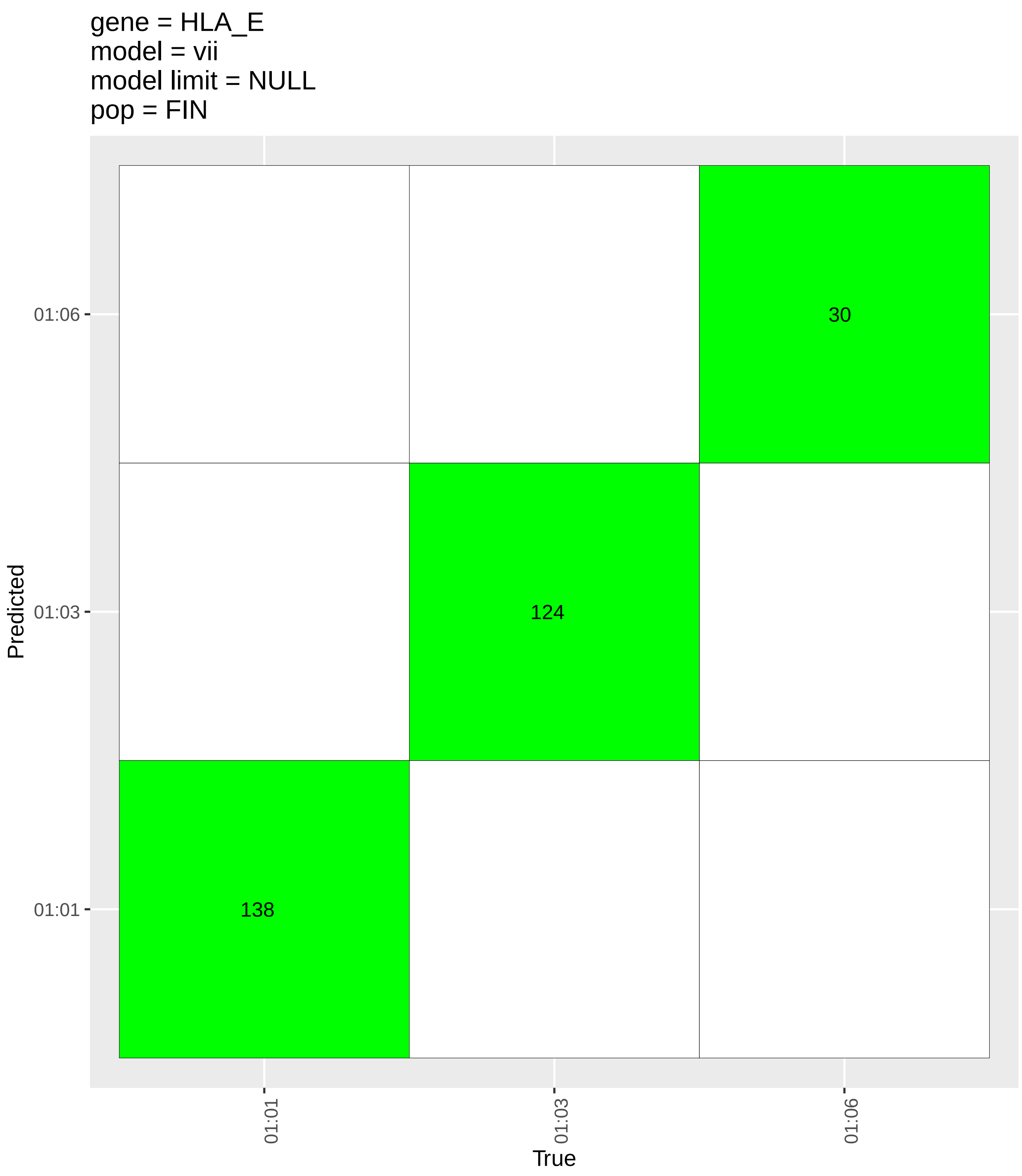
gene = HLA_E
model = vii
model limit = NULL
pop = FIN
Predicted
01:06
01:03
01:01
30
124
138
01:01
01:03
01:06
True

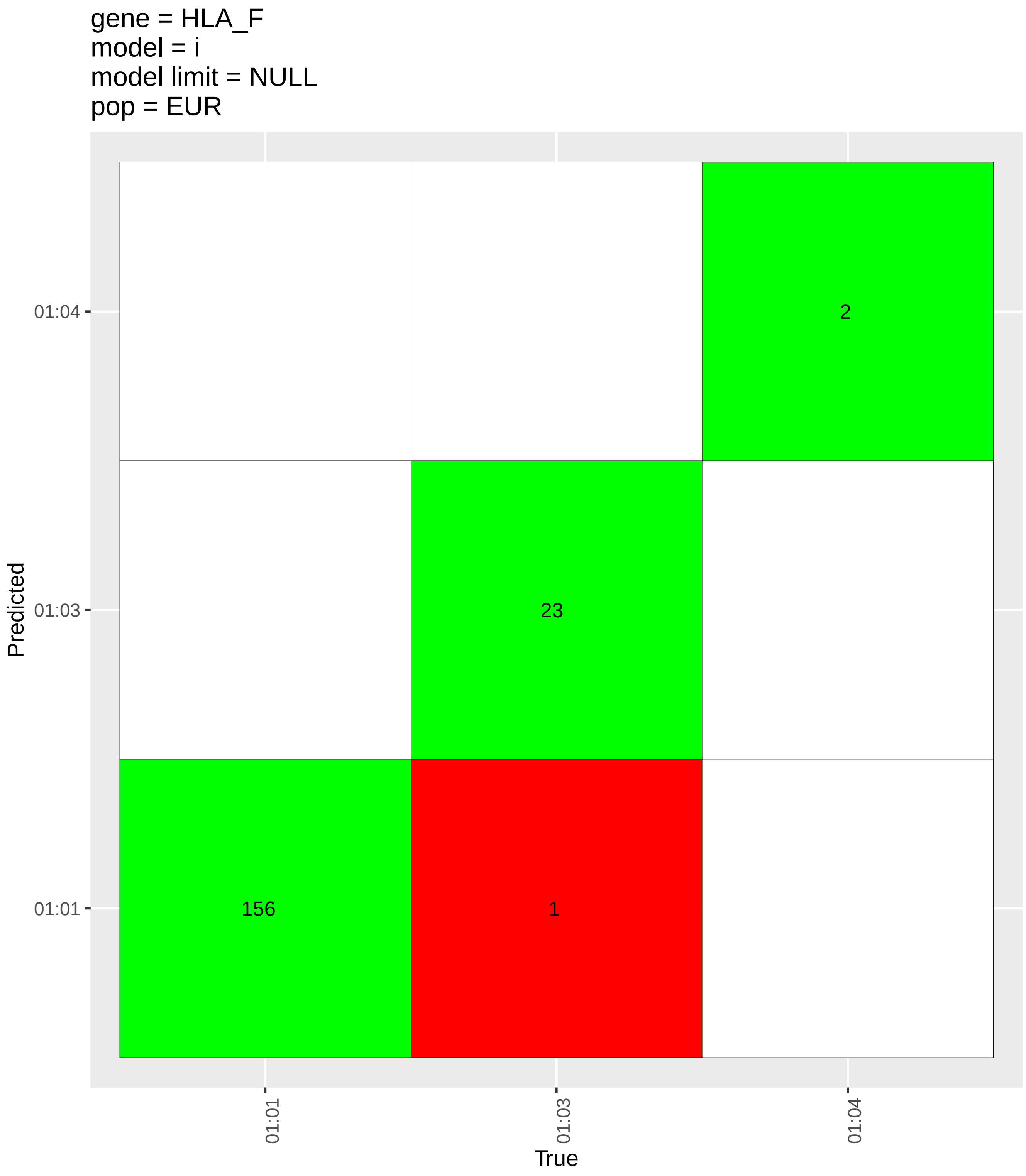
gene = HLA_F
model = i
model limit = NULL
pop = EUR
Predicted
01:04
01:03
01:01
2
23
156
1
01:01
01:03
01:04
True

gene = HLA_F
model = i
model limit = NULL
pop = AFR

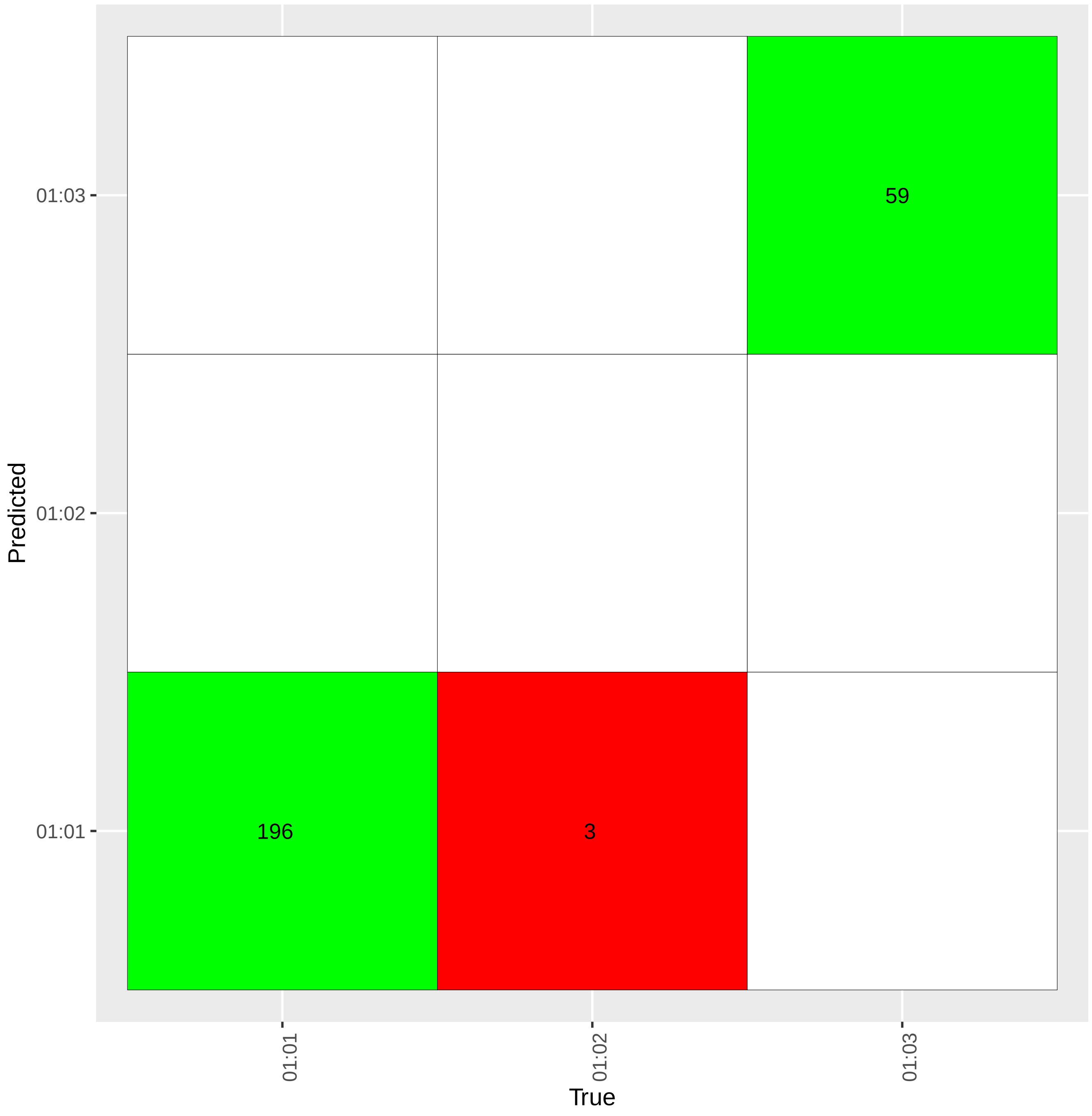

gene = HLA_F
model = i
model limit = NULL
pop = EAS

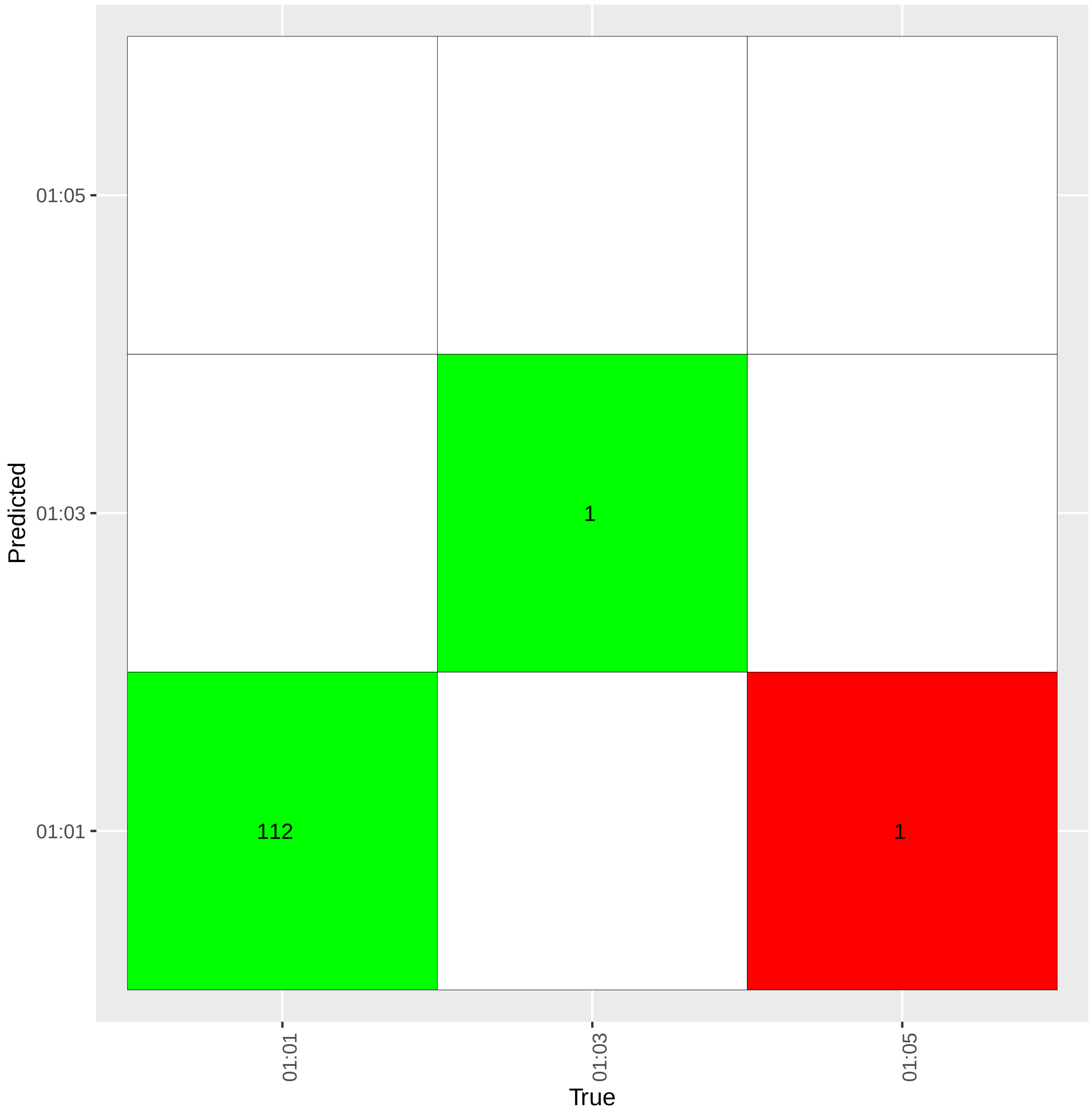

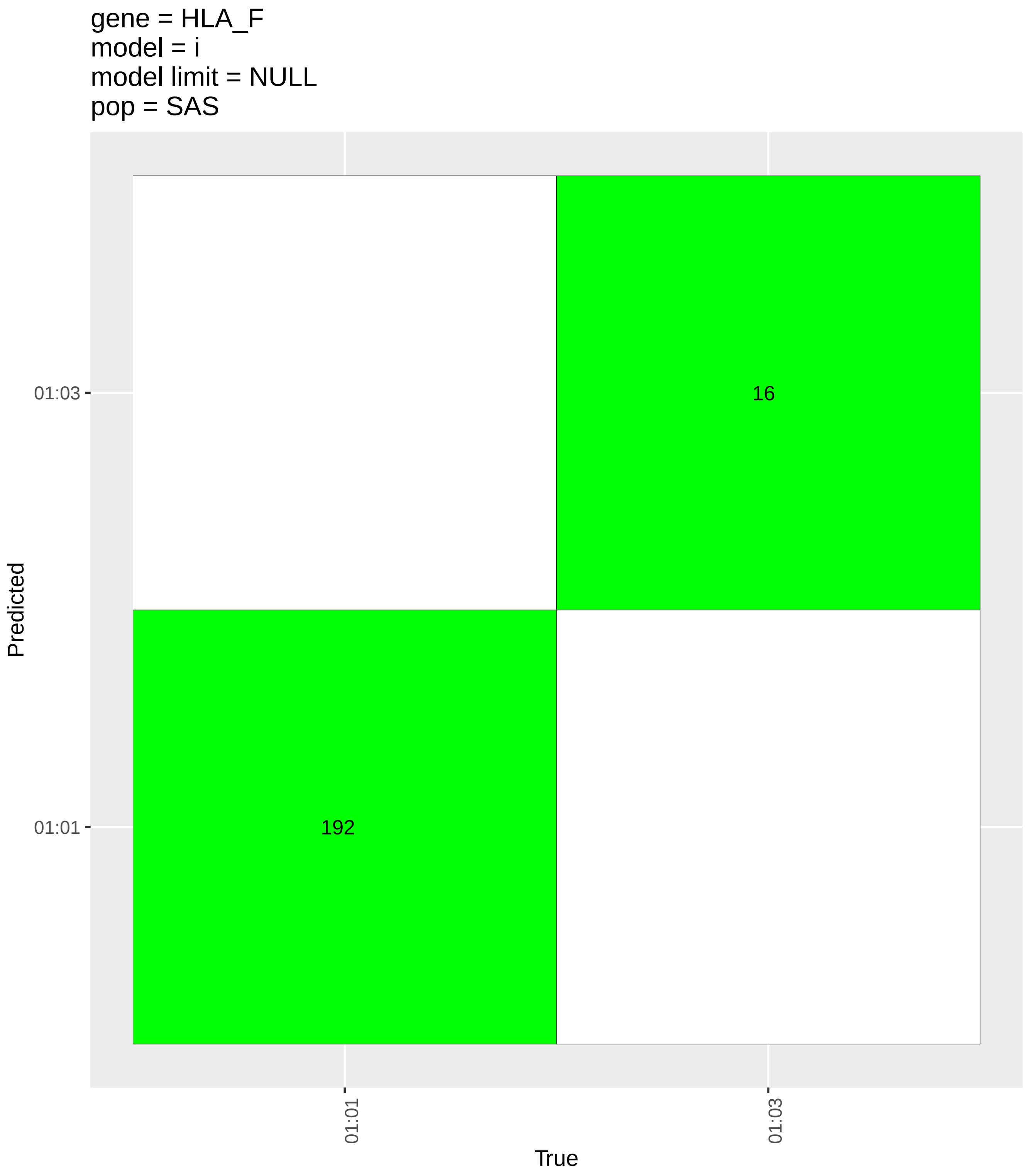
gene = HLA_F
model = i
model limit = NULL
pop = SAS
Predicted
01:03
01:01
16
192
01:01
01:03
True

gene = HLA_F
model = i
model limit = NULL
pop = AMR

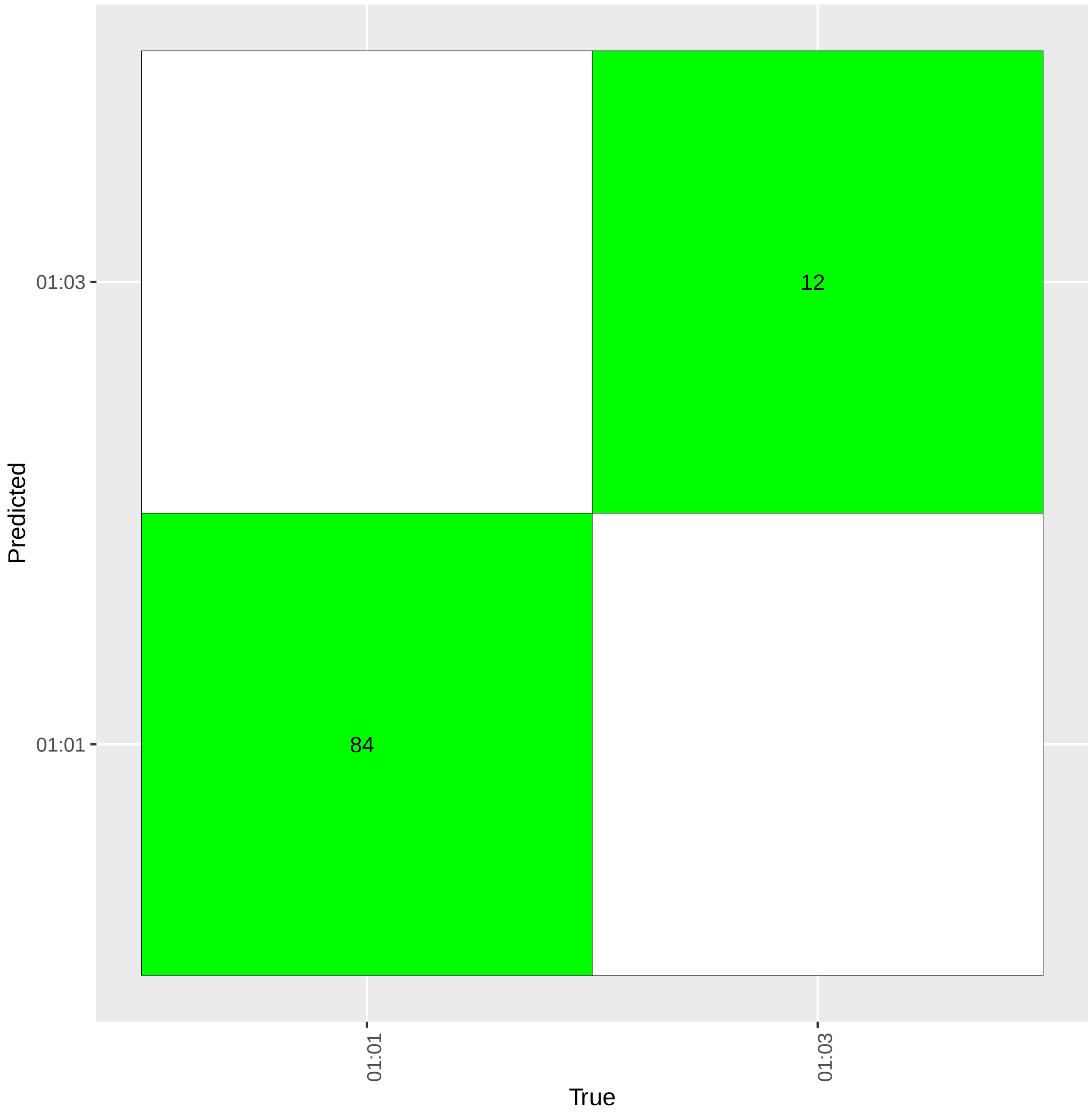

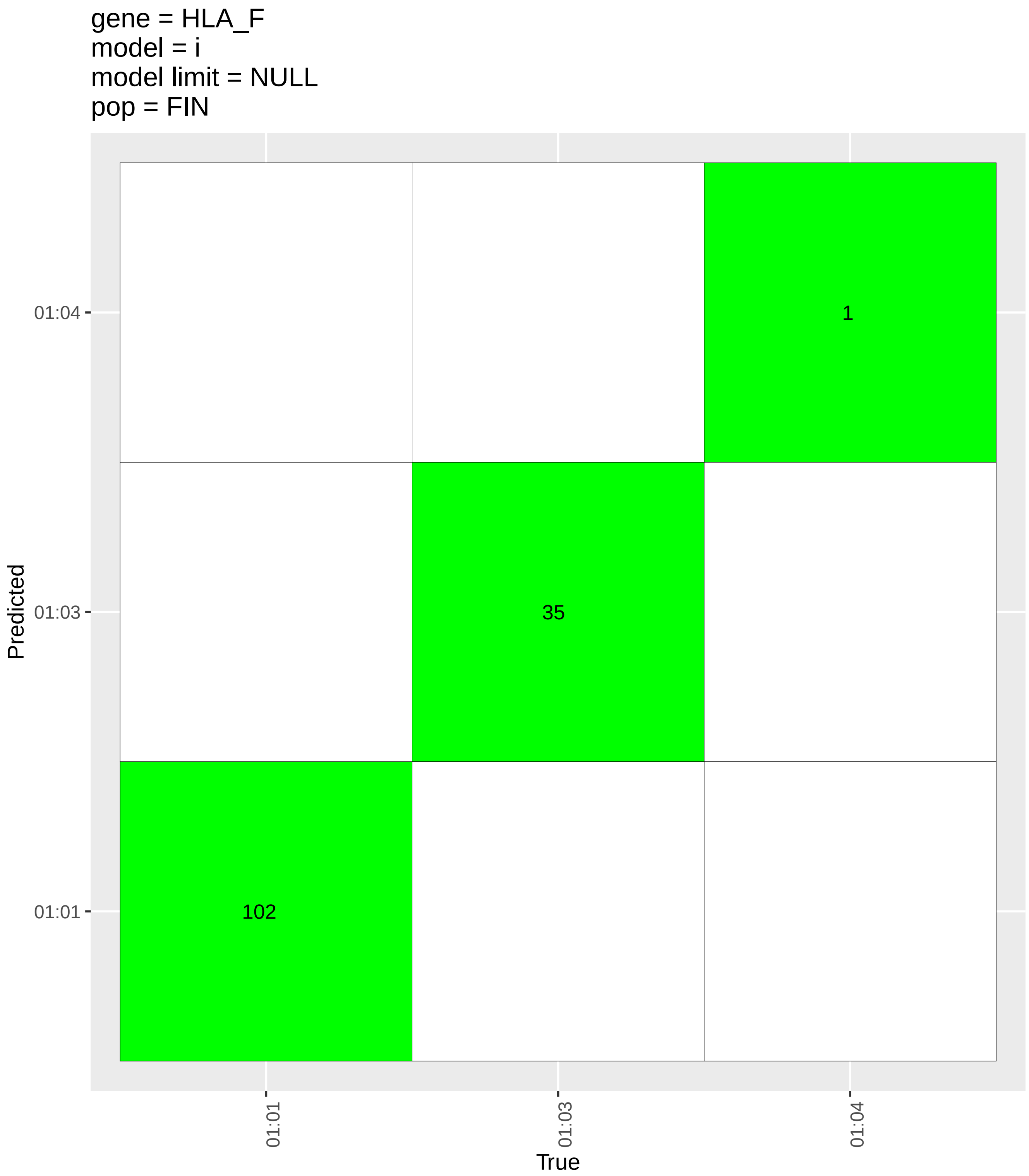
gene = HLA_F
model = i
model limit = NULL
pop = FIN
Predicted
01:04
01:03
01:01
1
35
102
01:01
01:03
01:04
True

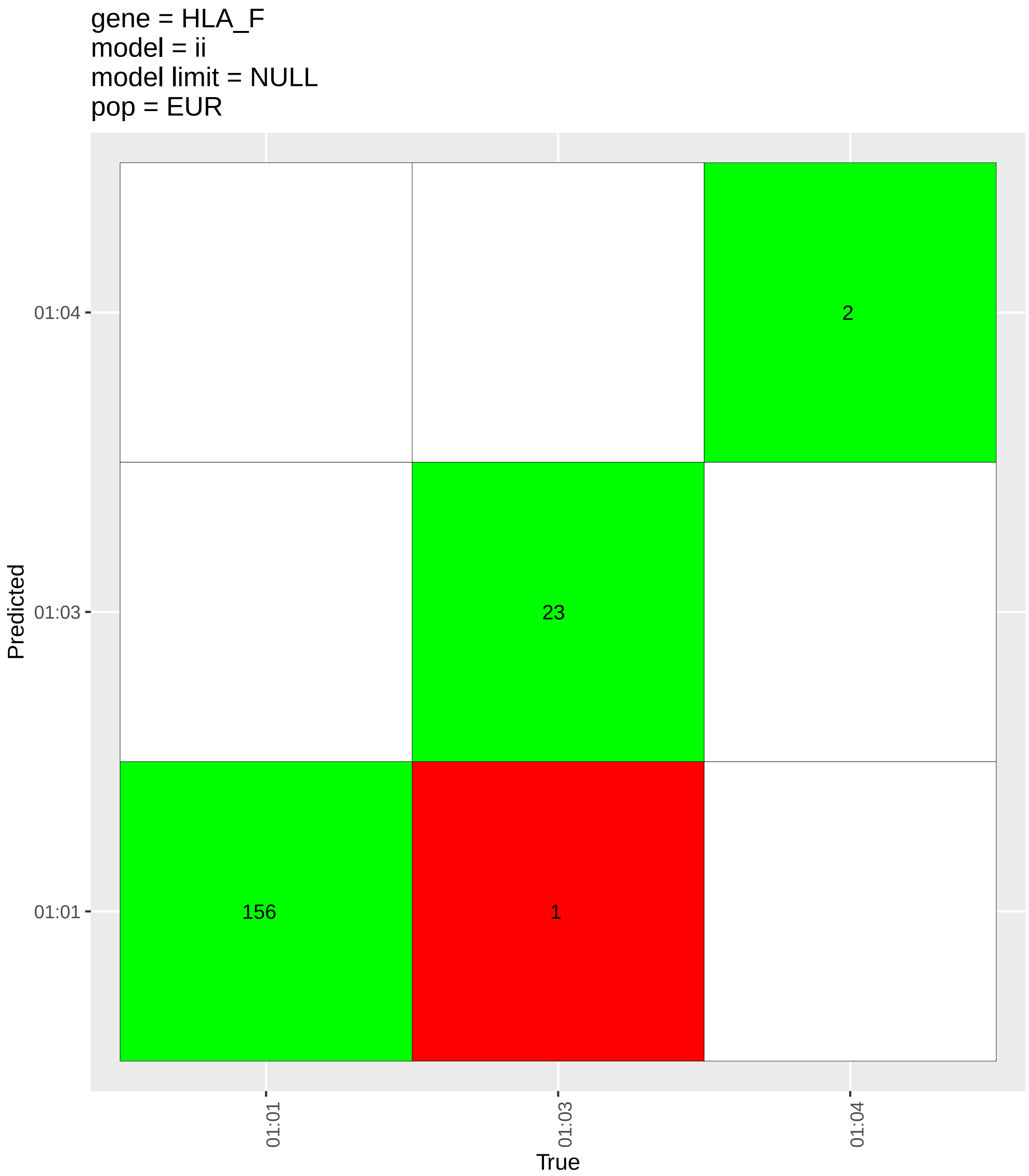

gene = HLA_F
model = ii
model limit = NULL
pop = EUR
Predicted
01:04
01:03
01:01
2
23
156
1
01:01
01:03
01:04
True

gene = HLA_F
model = ii
model limit = NULL
pop = AFR

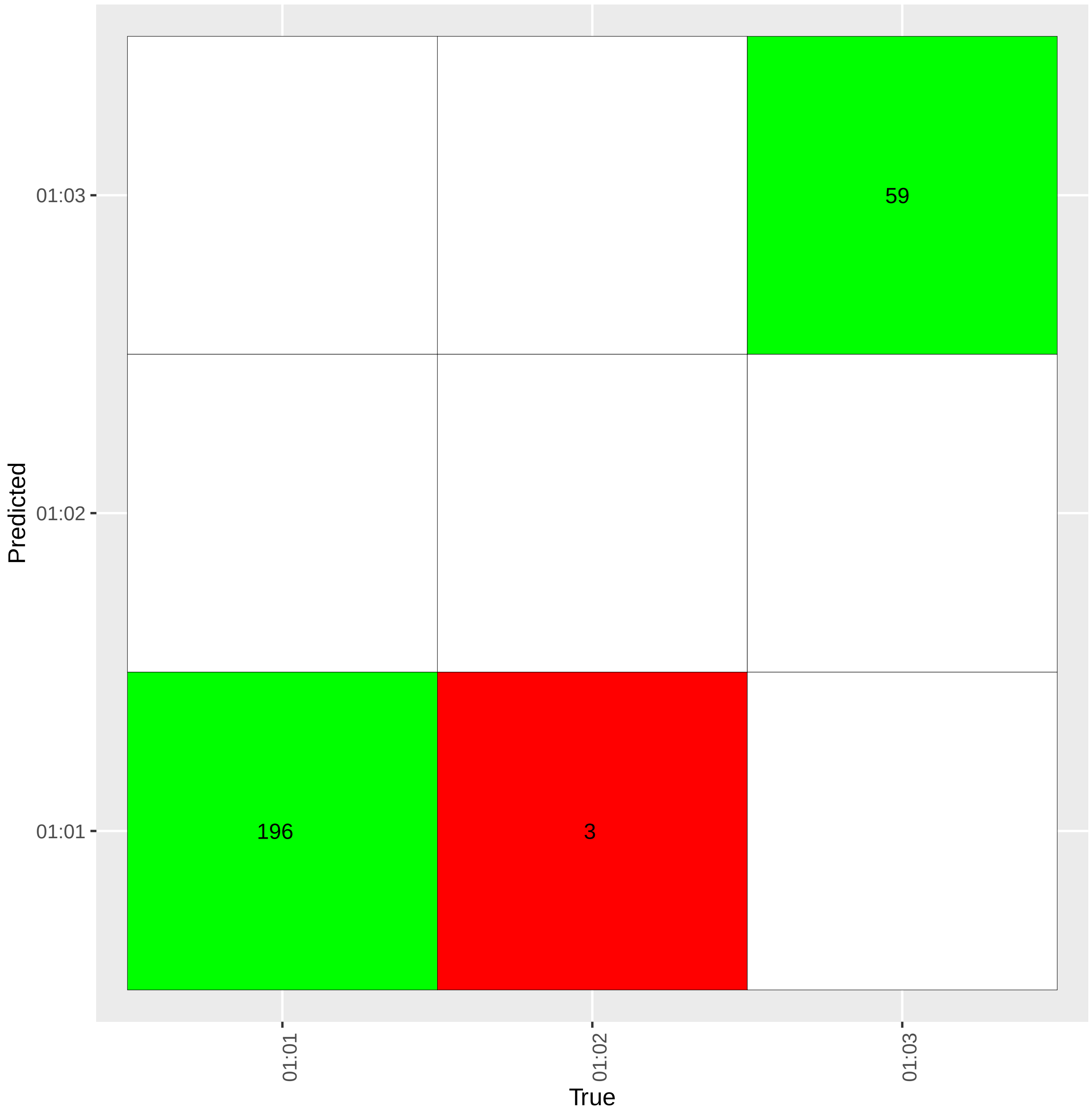

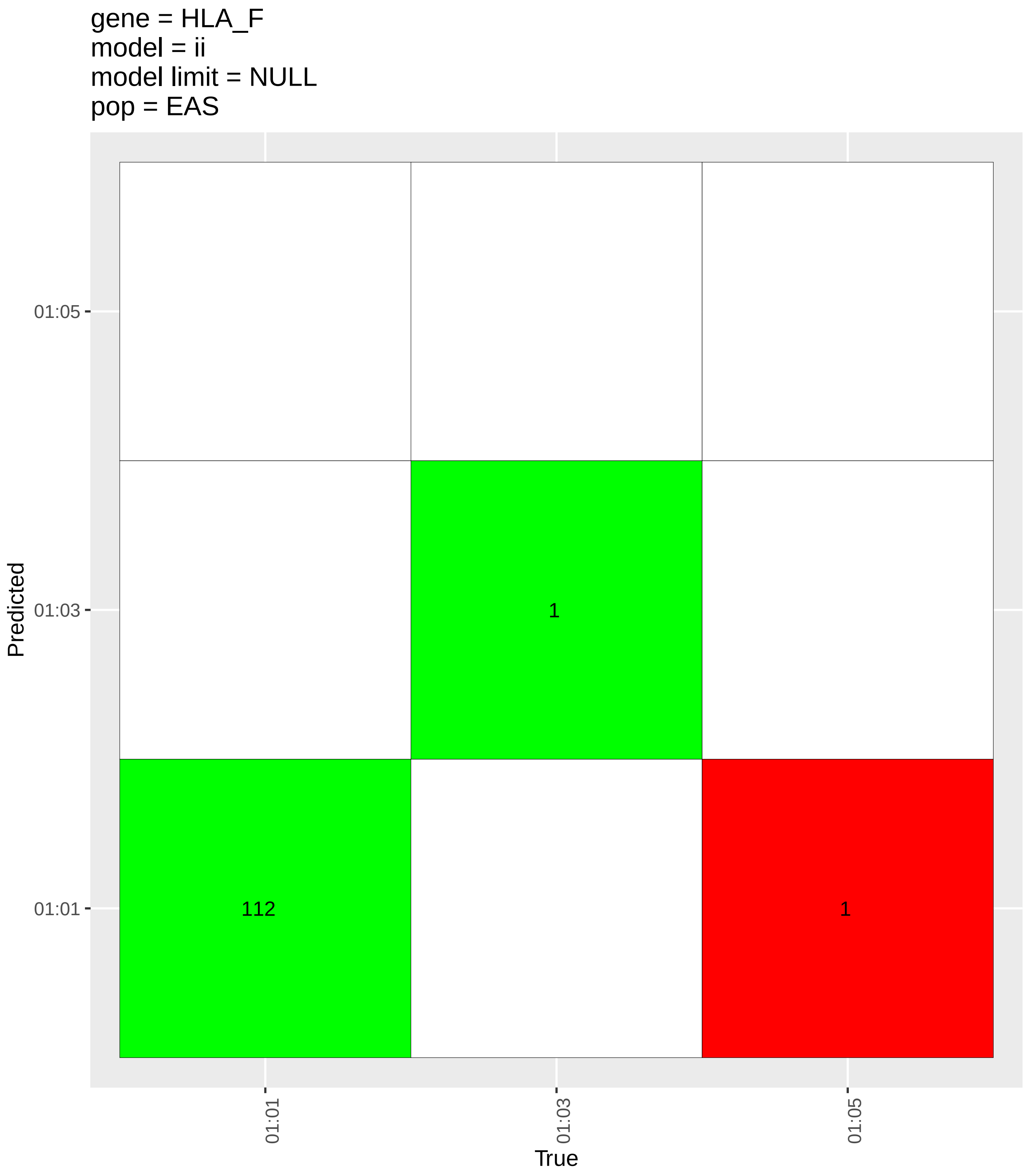
gene = HLA_F
model = ii
model limit = NULL
pop = EAS
Predicted
01:05
01:03
01:01
1
112
1
01:01
01:03
01:05
True

gene = HLA_F
model = ii
model limit = NULL
pop = SAS

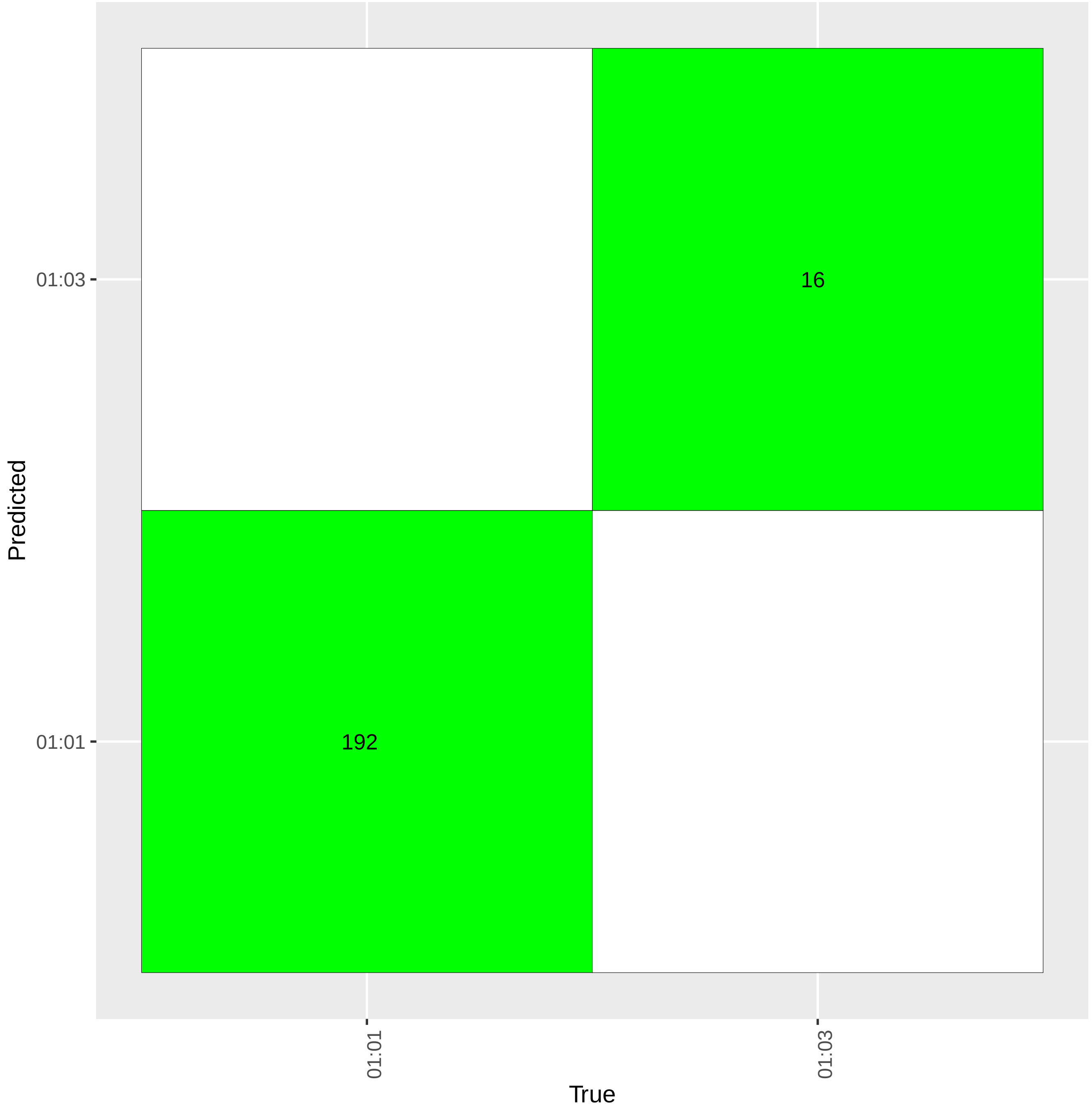

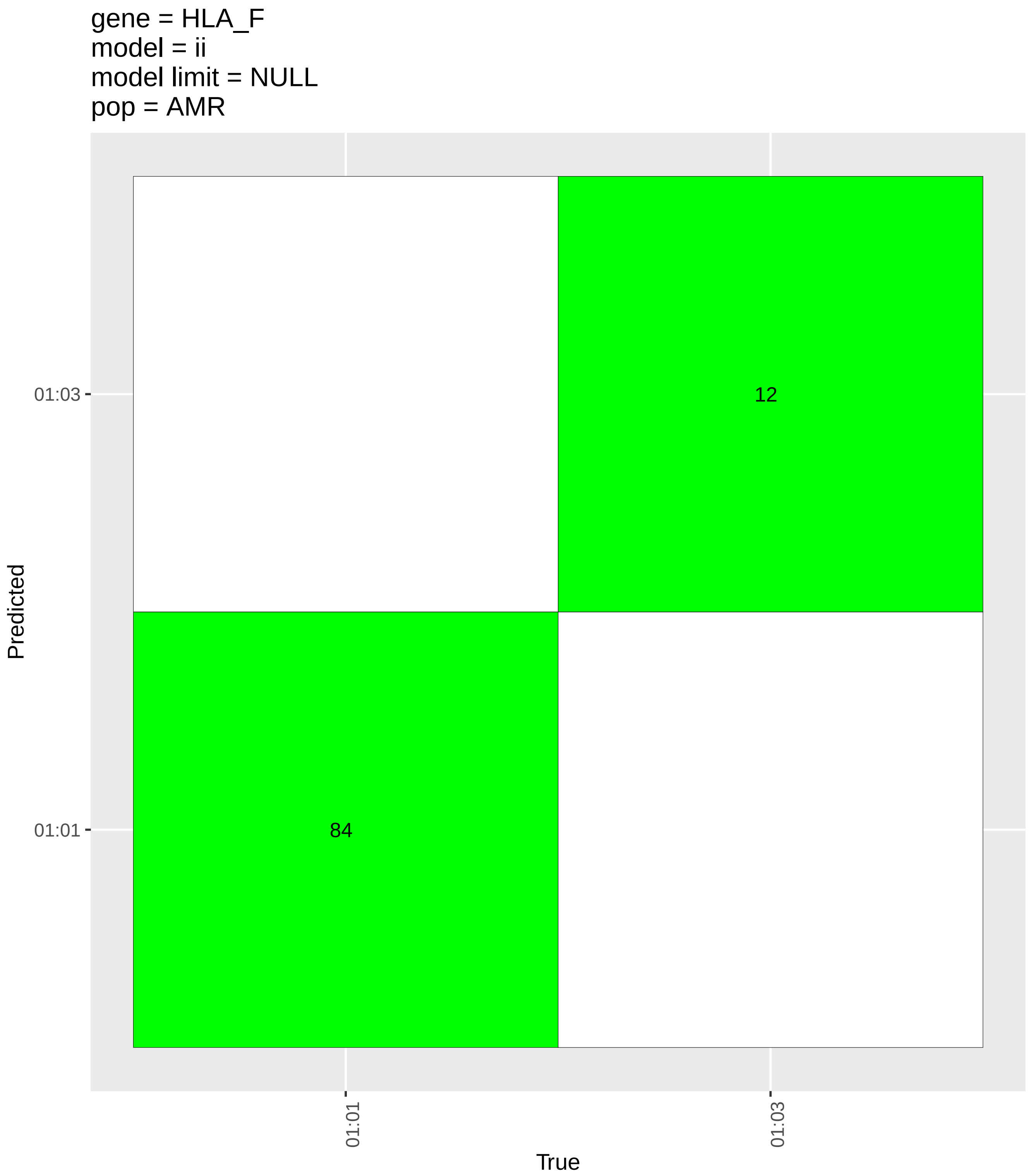
gene = HLA_F
model = ii
model limit = NULL
pop = AMR
Predicted
01:03
01:01
12
84
01:01
01:03
True

gene = HLA_F
model = ii
model limit = NULL
pop = FIN

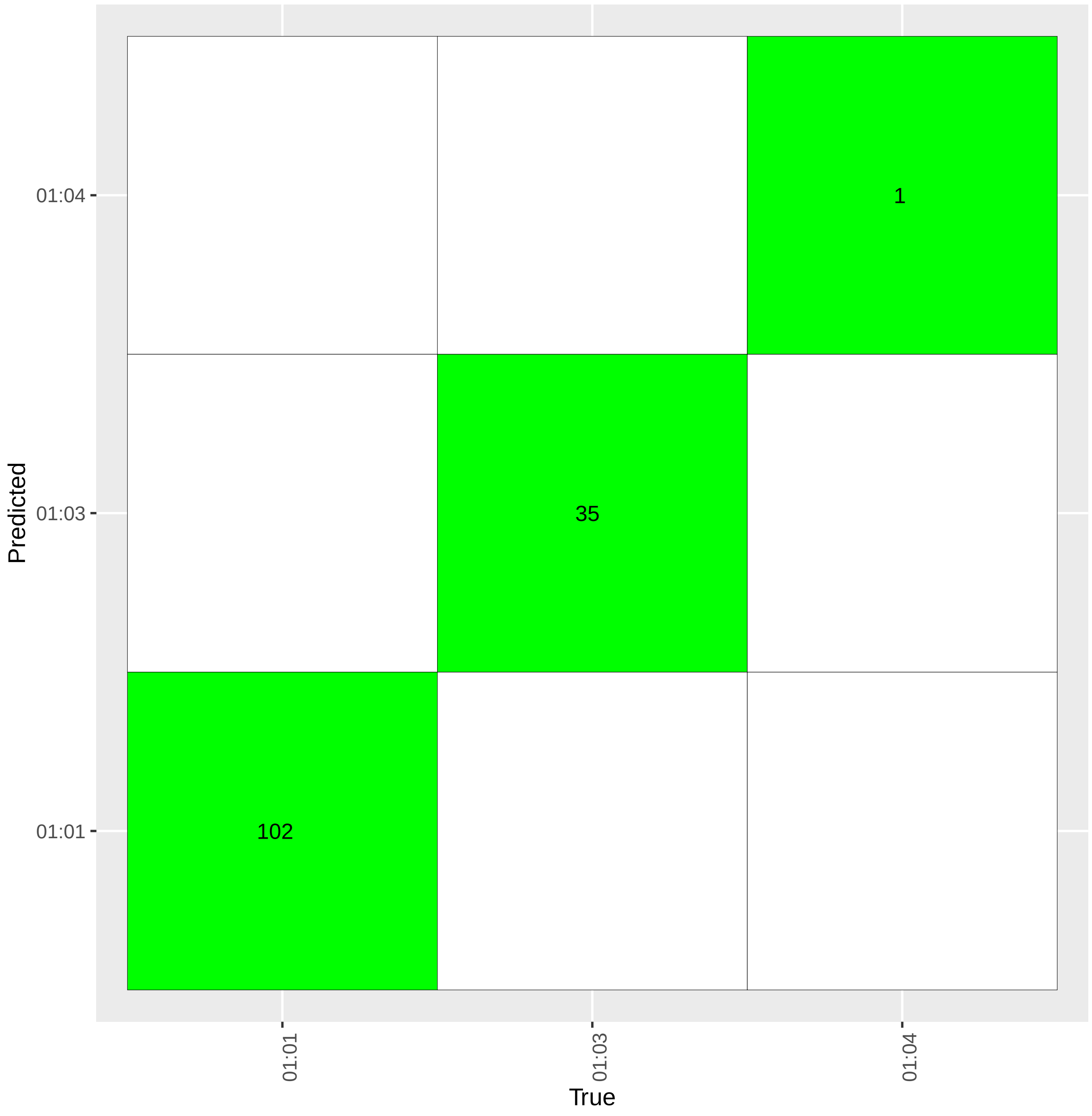

gene = HLA_F
model = iii
model limit = NULL
pop = EUR

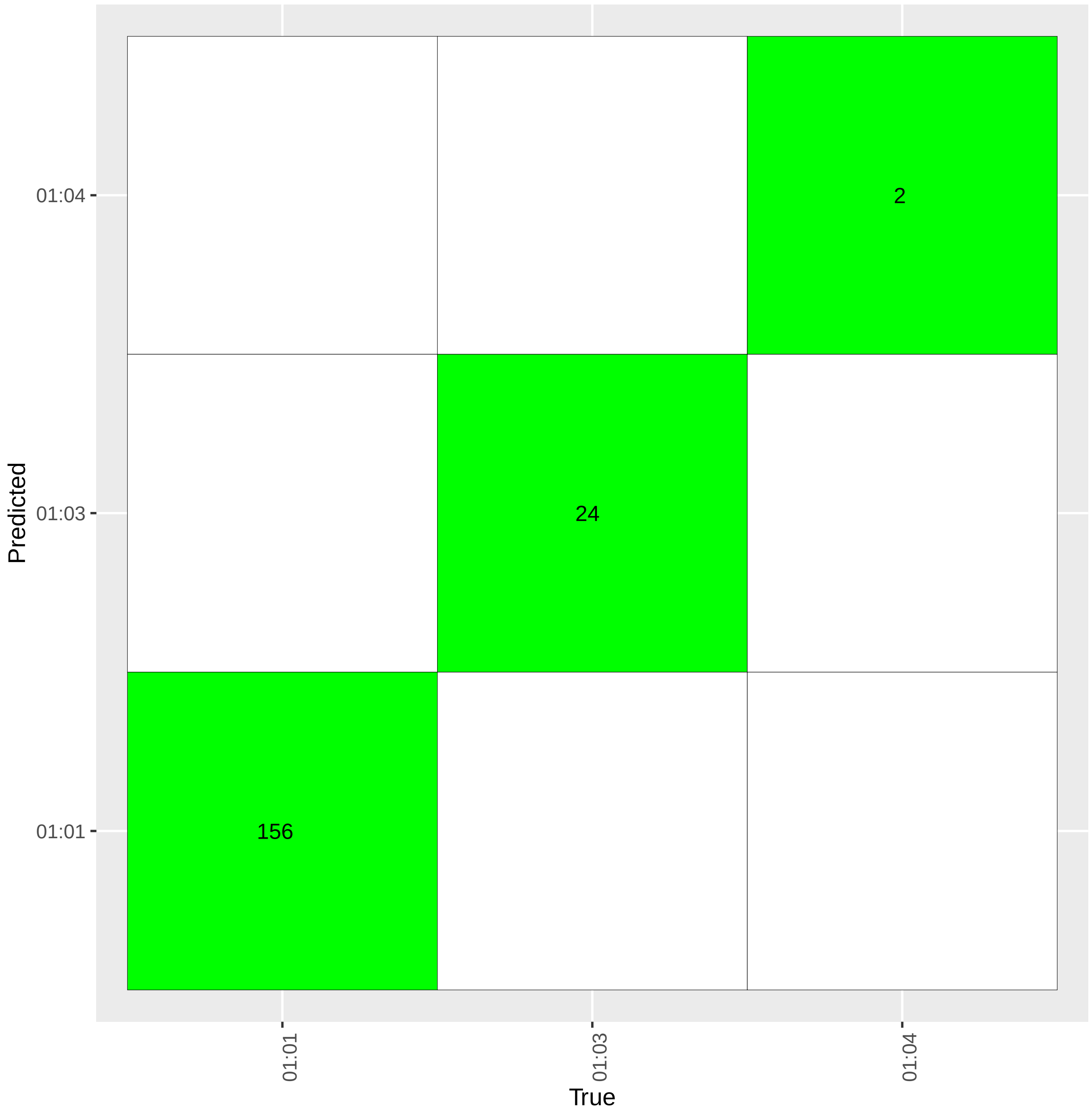

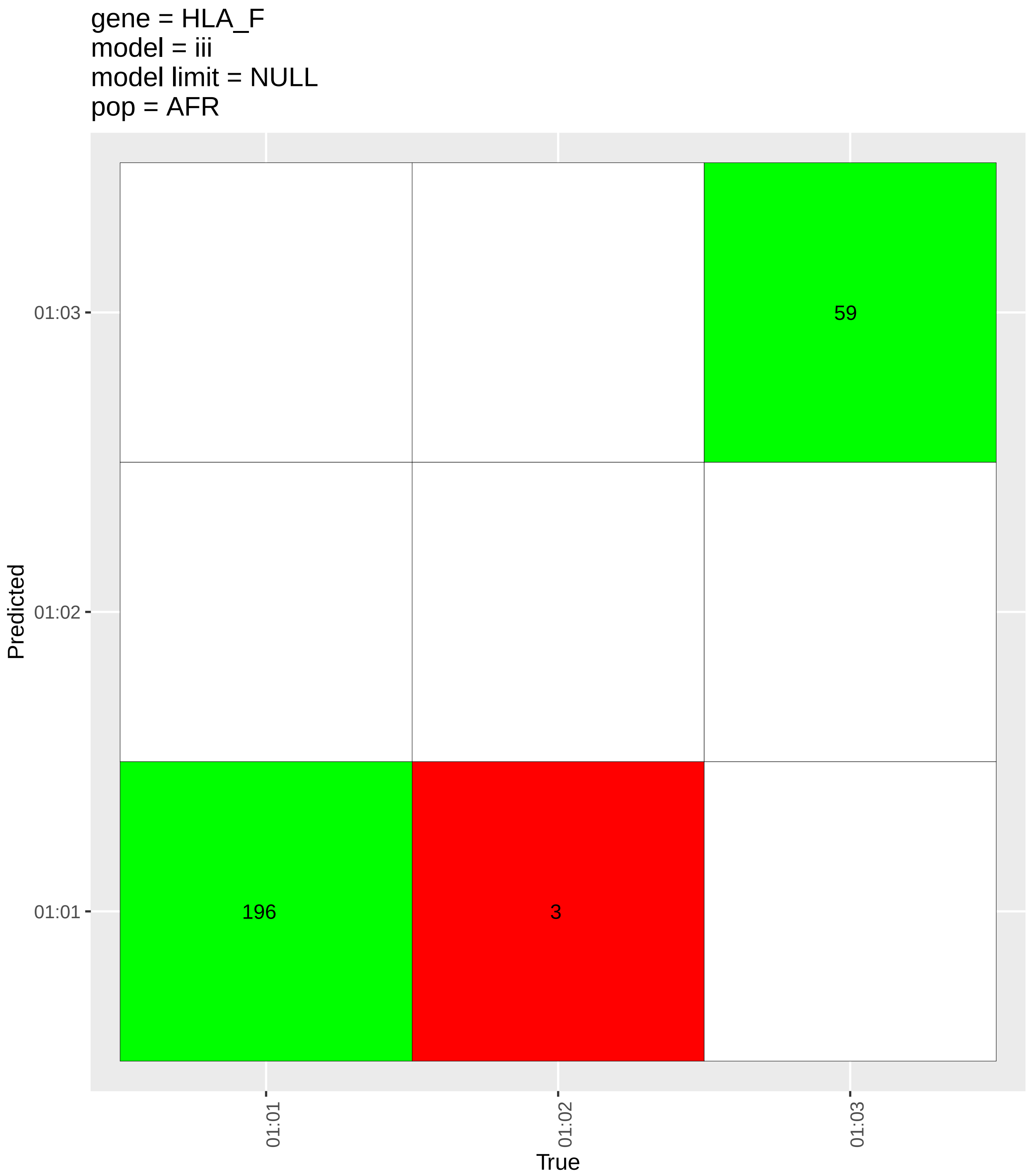

gene = HLA_F
model = iii
model limit = NULL
pop = AFR
Predicted
01:03
01:02
01:01
59
196
3
01:01
01:02
01:03
True

gene = HLA_F
model = iii
model limit = NULL
pop = EAS

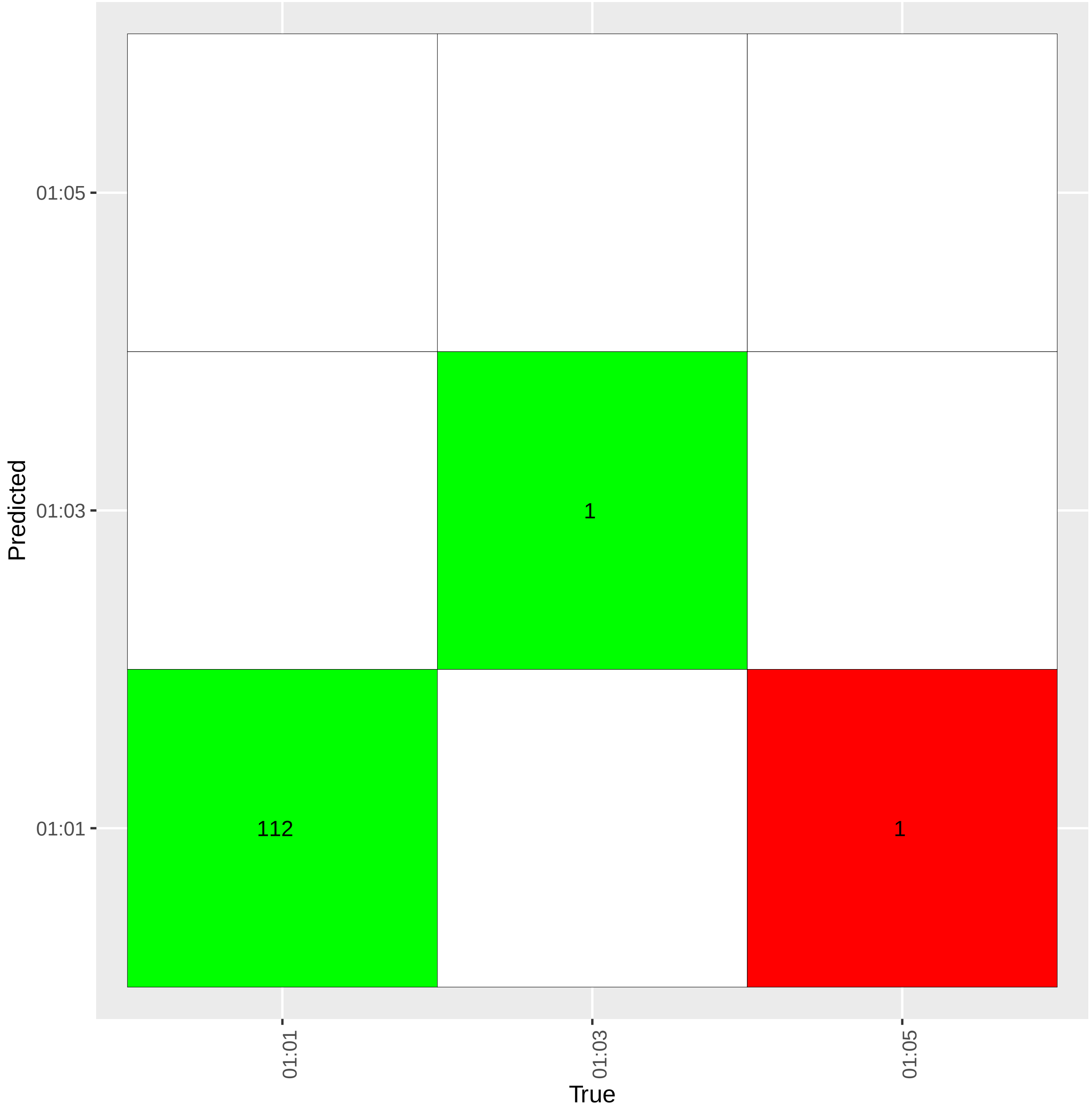

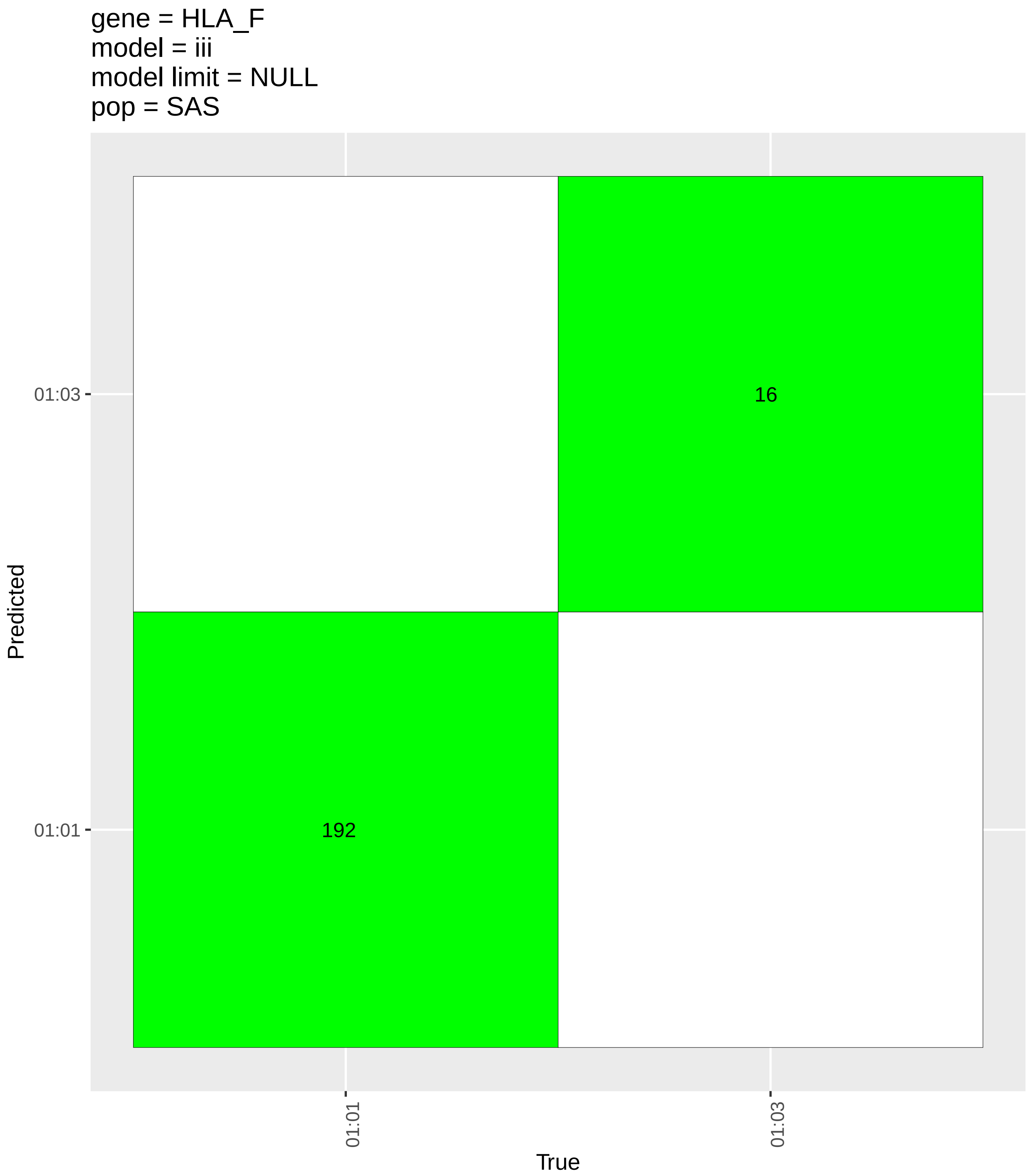
gene = HLA_F
model = iii
model limit = NULL
pop = SAS
Predicted
01:03
01:01
16
192
01:01
01:03
True

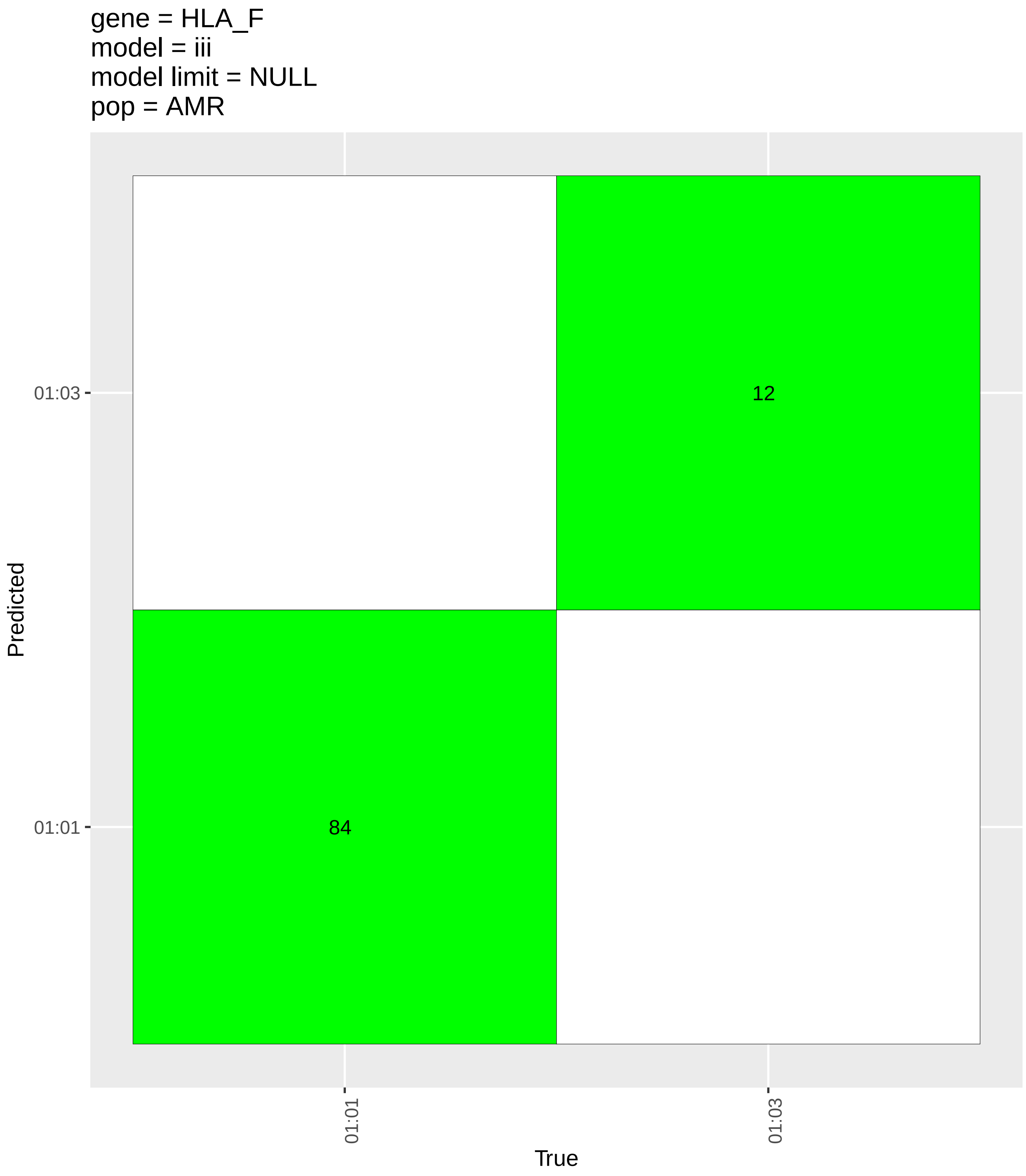
gene = HLA_F
model = iii
model limit = NULL
pop = AMR
Predicted
01:03
01:01
12
84
01:01
01:03
True

gene = HLA_F
model = iii
model limit = NULL
pop = FIN

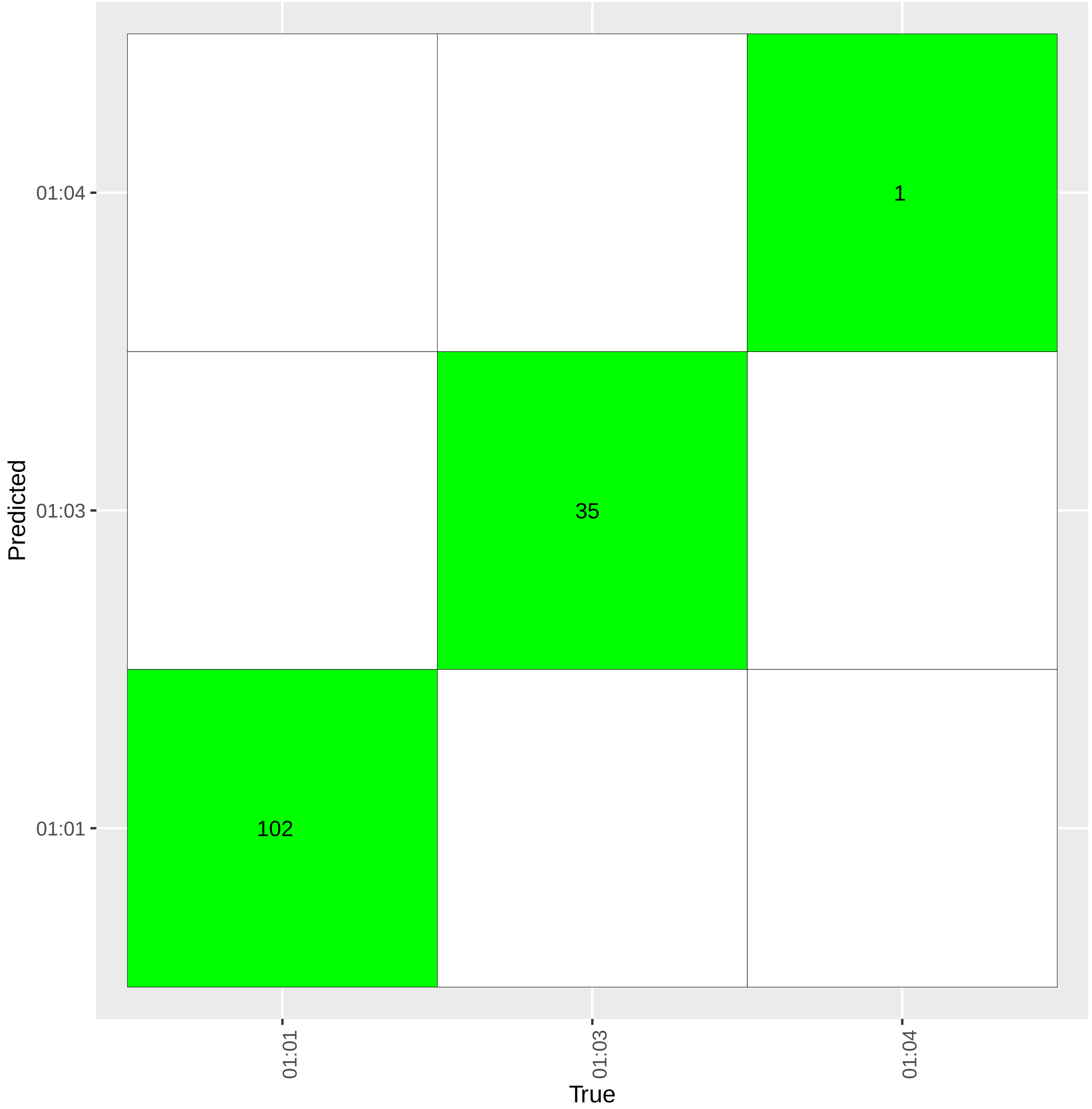

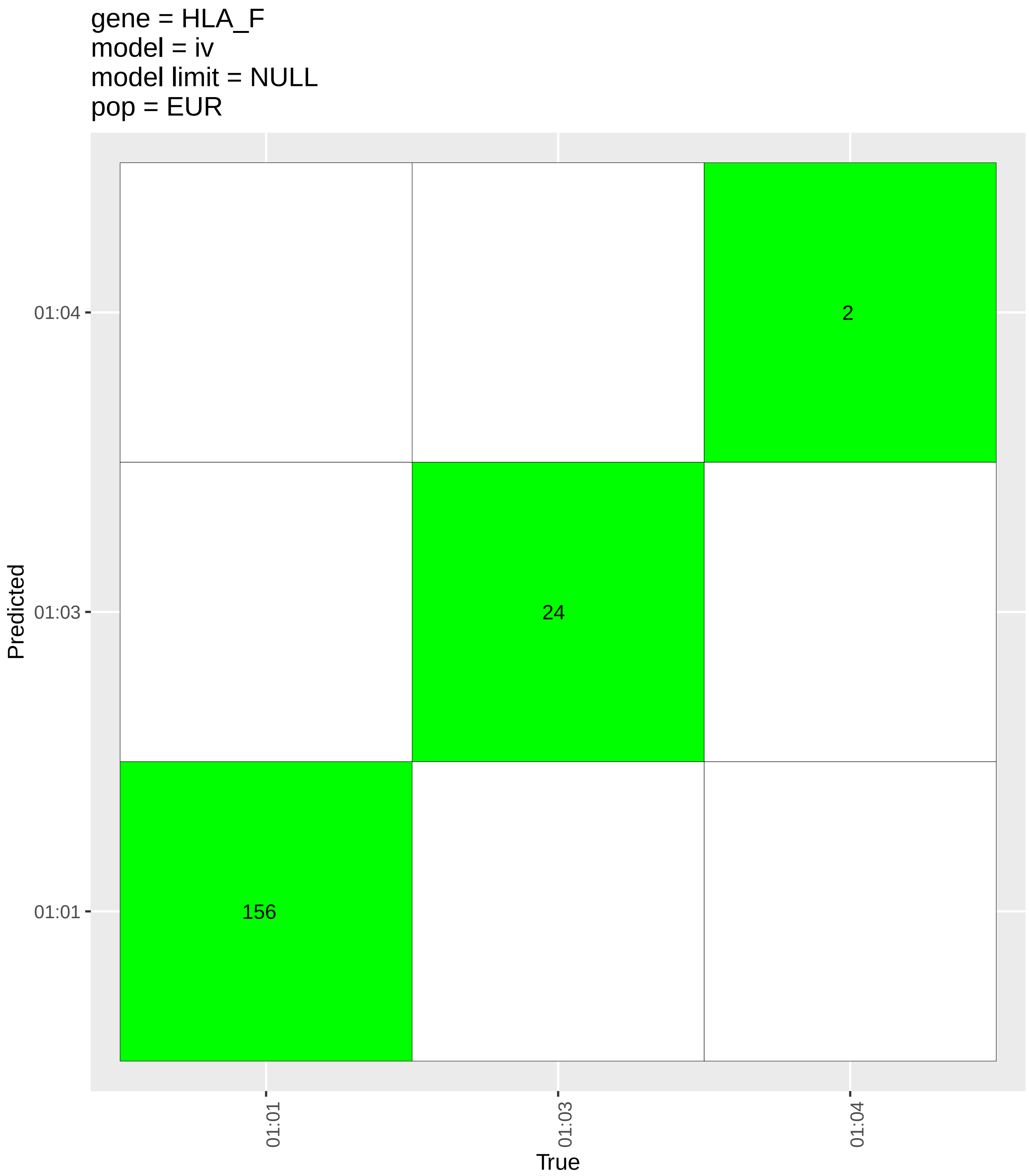
gene = HLA_F
model = iv
model limit = NULL
pop = EUR
Predicted
01:04
01:03
01:01
2
24
156
01:01
01:03
01:04
True

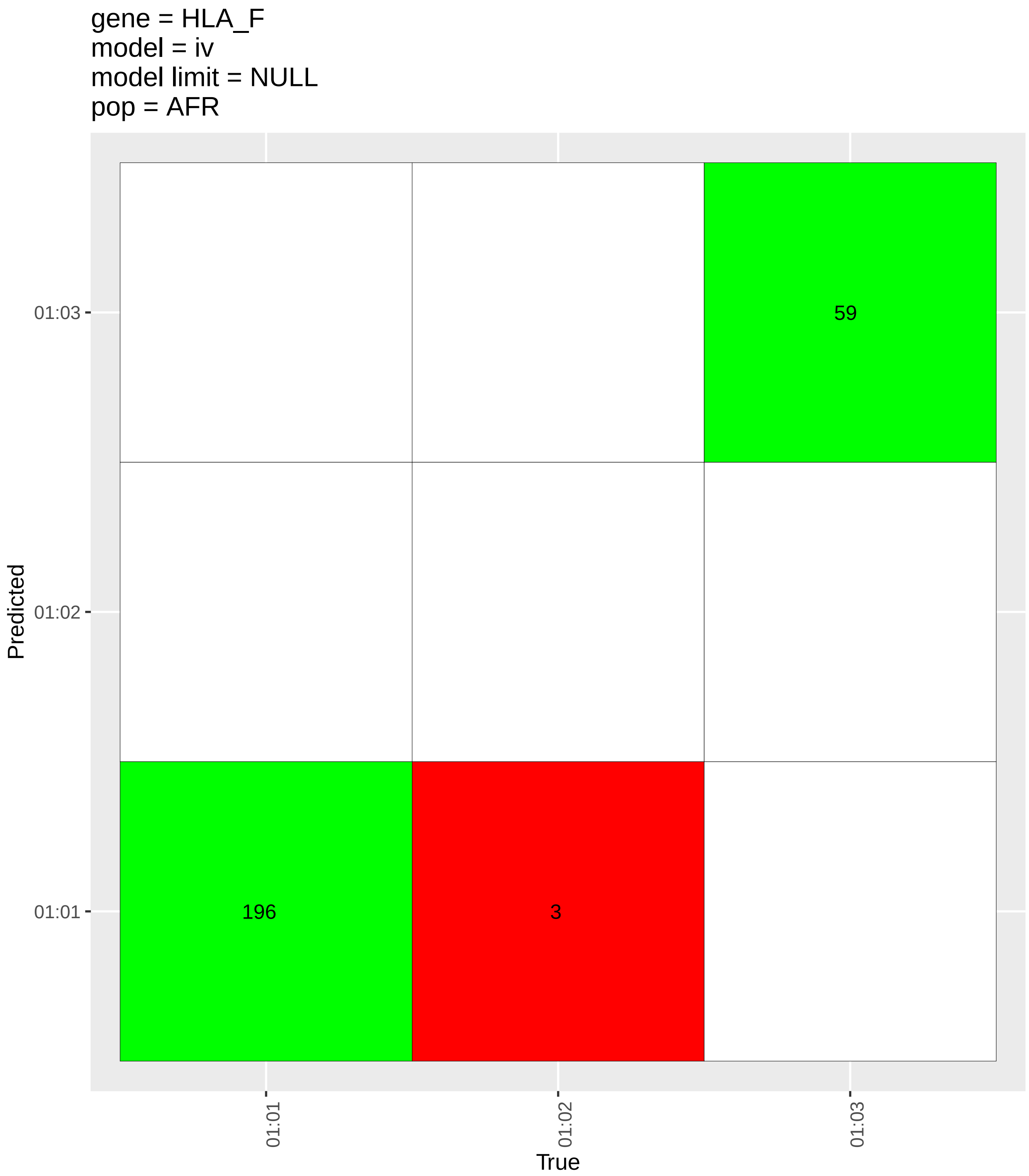
gene = HLA_F
model = iv
model limit = NULL
pop = AFR
Predicted
01:03
01:02
01:01
59
196
3
01:01
01:02
01:03
True

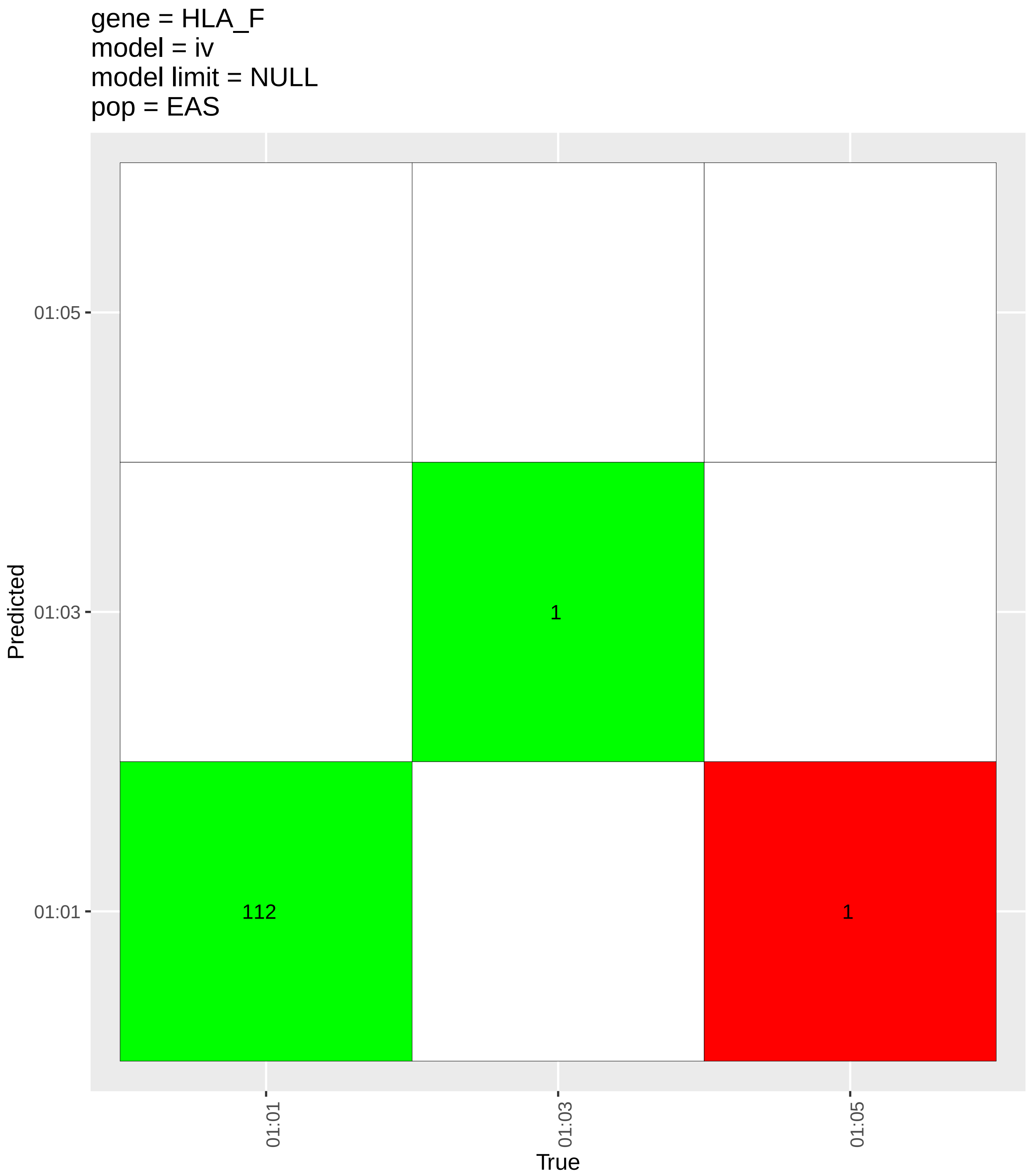
gene = HLA_F
model = iv
model limit = NULL
pop = EAS
Predicted
01:05
01:03
01:01
1
112
1
01:01
01:03
01:05
True

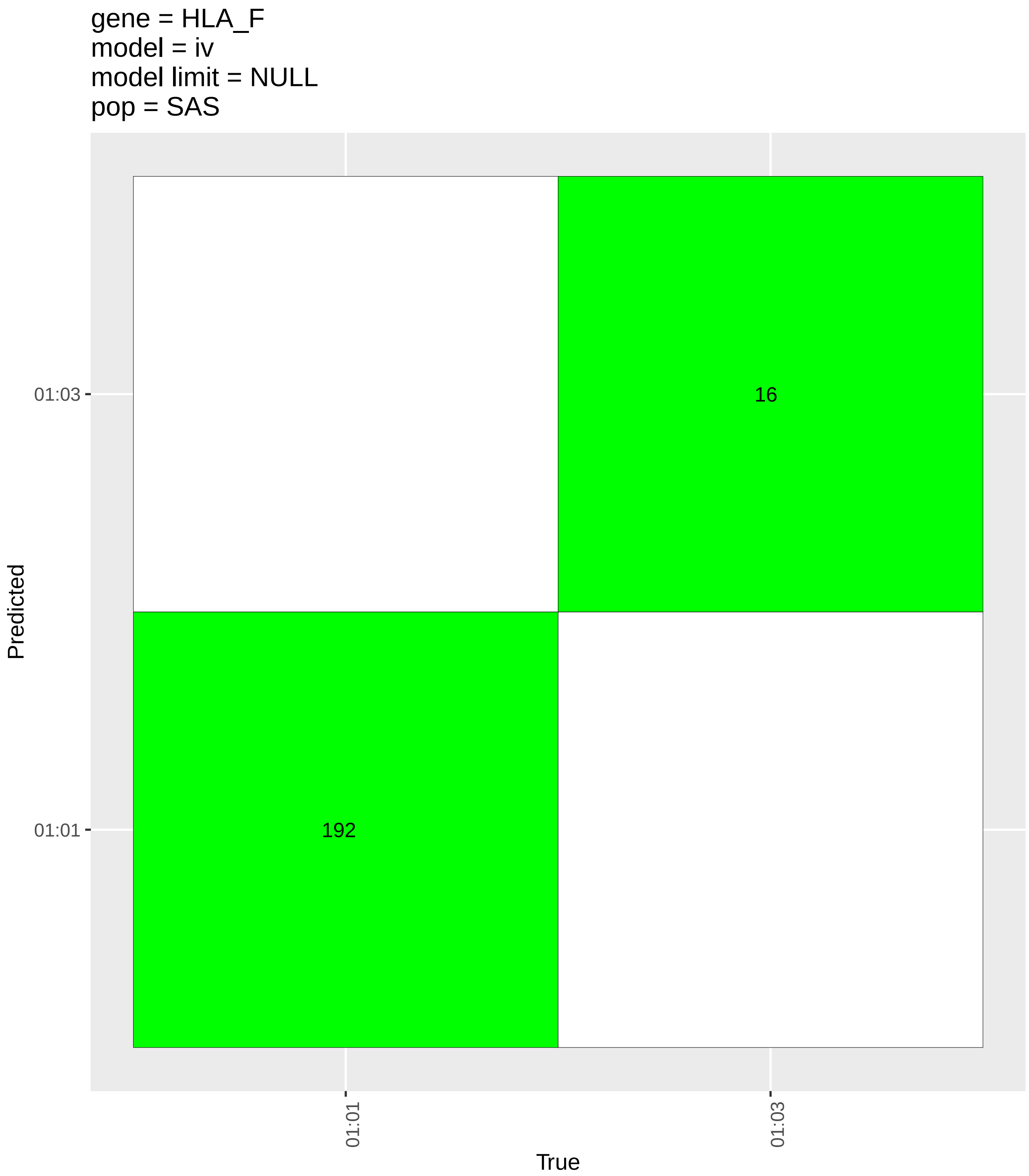
gene = HLA_F
model = iv
model limit = NULL
pop = SAS
Predicted
01:03
01:01
16
192
01:01
01:03
True

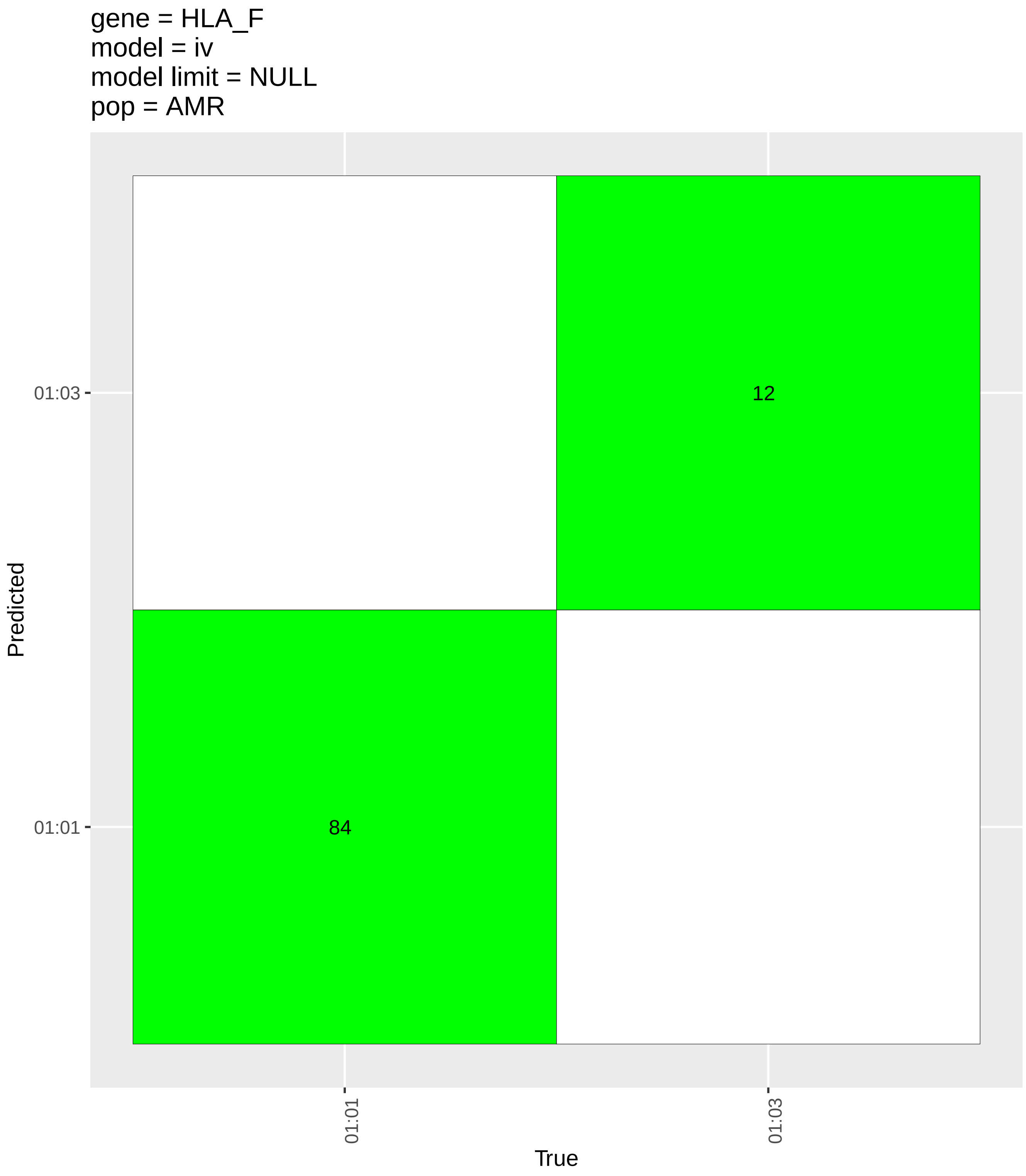

gene = HLA_F
model = iv
model limit = NULL
pop = AMR
Predicted
01:03
01:01
12
84
01:01
01:03
True

gene = HLA_F
model = iv
model limit = NULL
pop = FIN

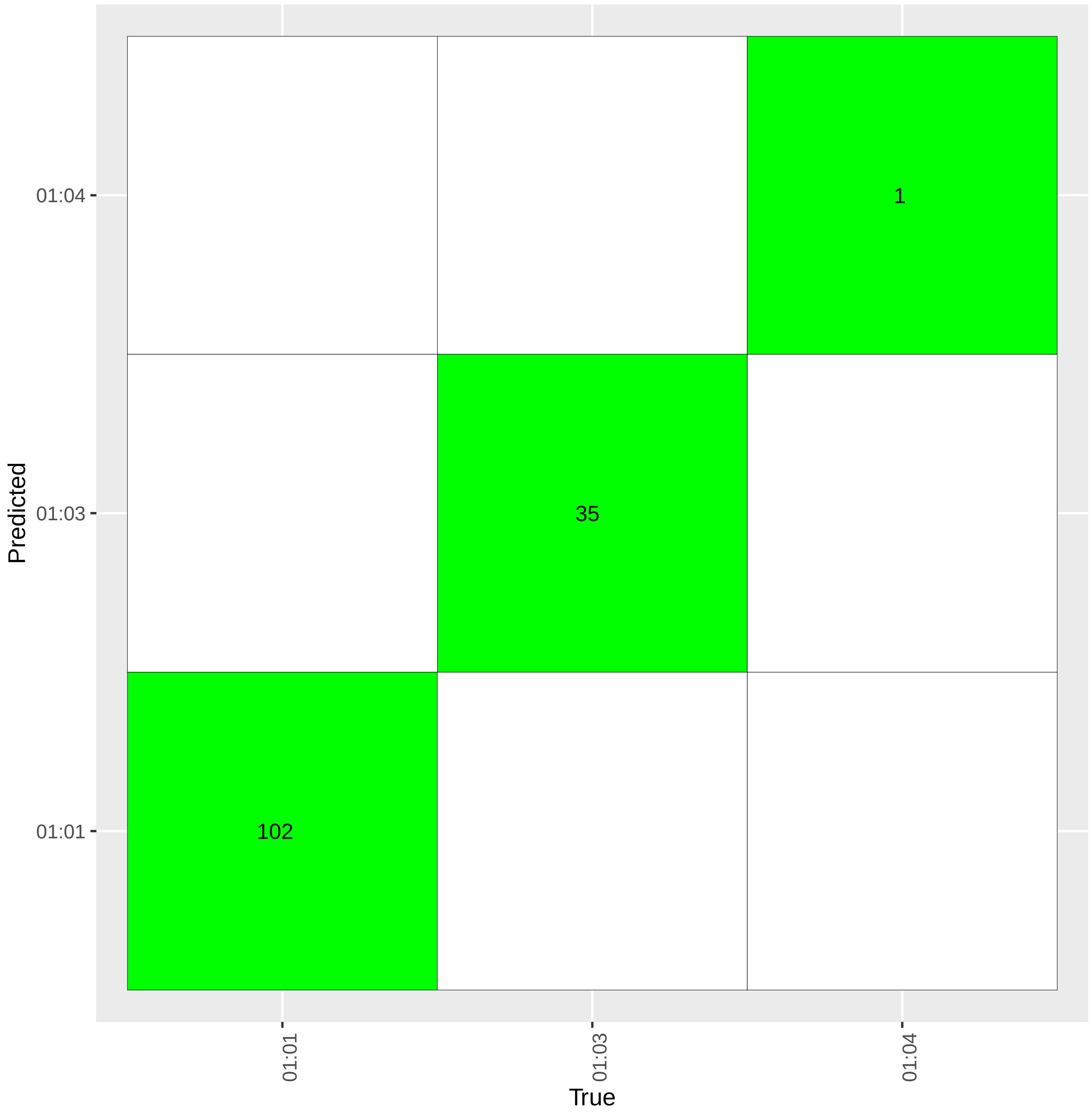

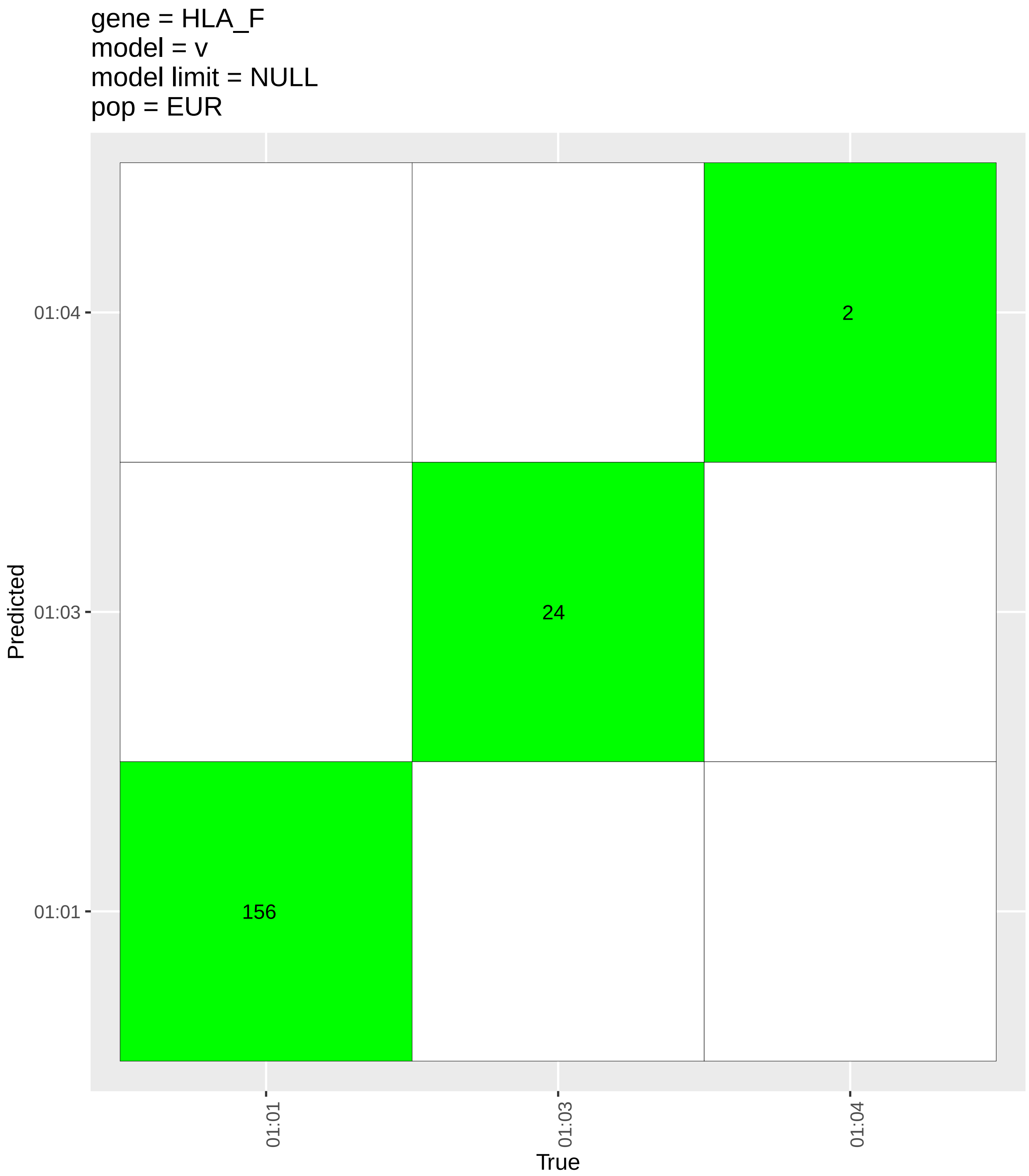
gene = HLA_F
model = v
model limit = NULL
pop = EUR
Predicted
01:04
01:03
01:01
2
24
156
01:01
01:03
01:04
True

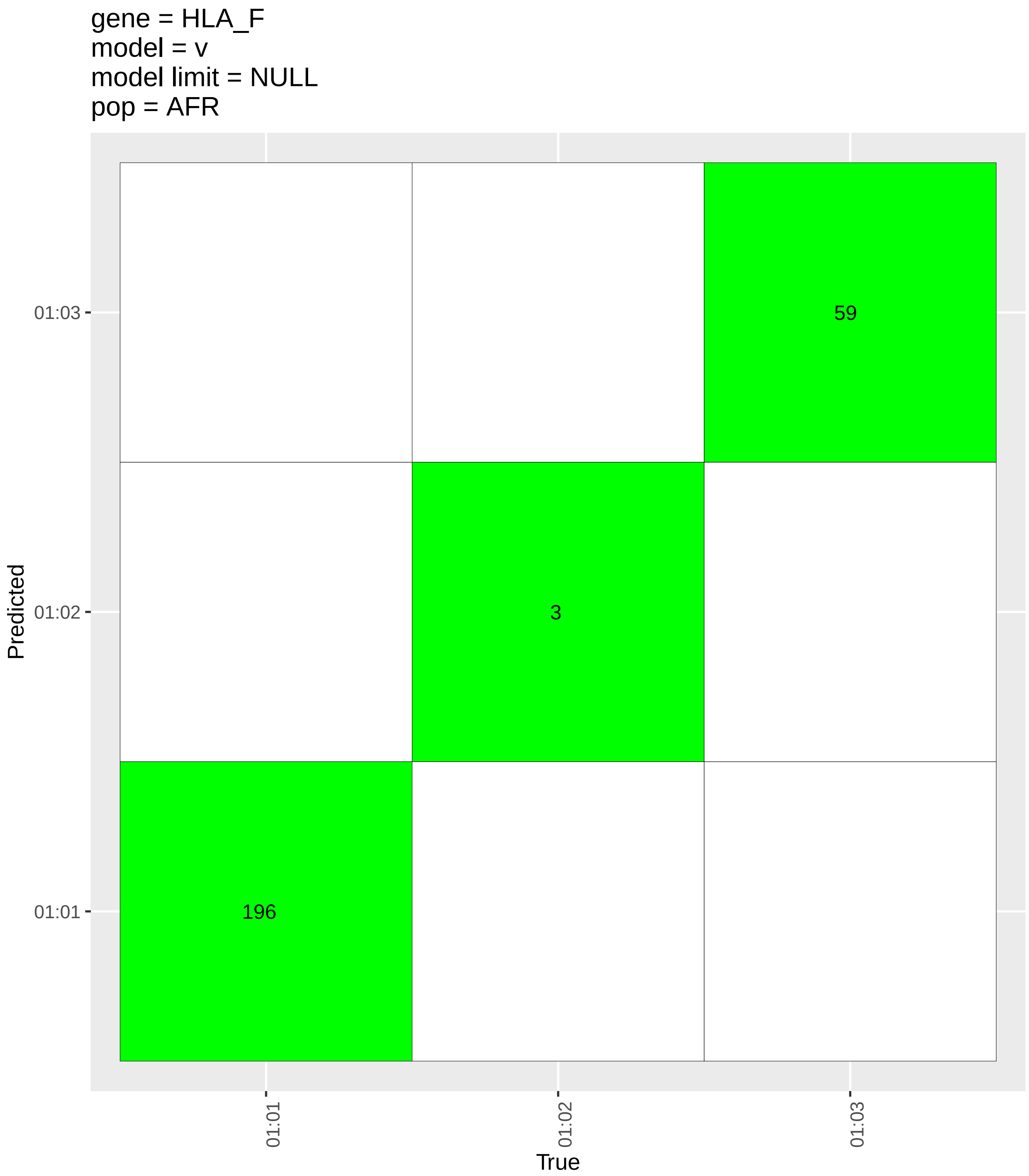
gene = HLA_F
model = v
model limit = NULL
pop = AFR
Predicted
01:03
01:02
01:01
59
3
196
01:01
01:02
01:03
True

gene = HLA_F
model = v
model limit = NULL
pop = EAS

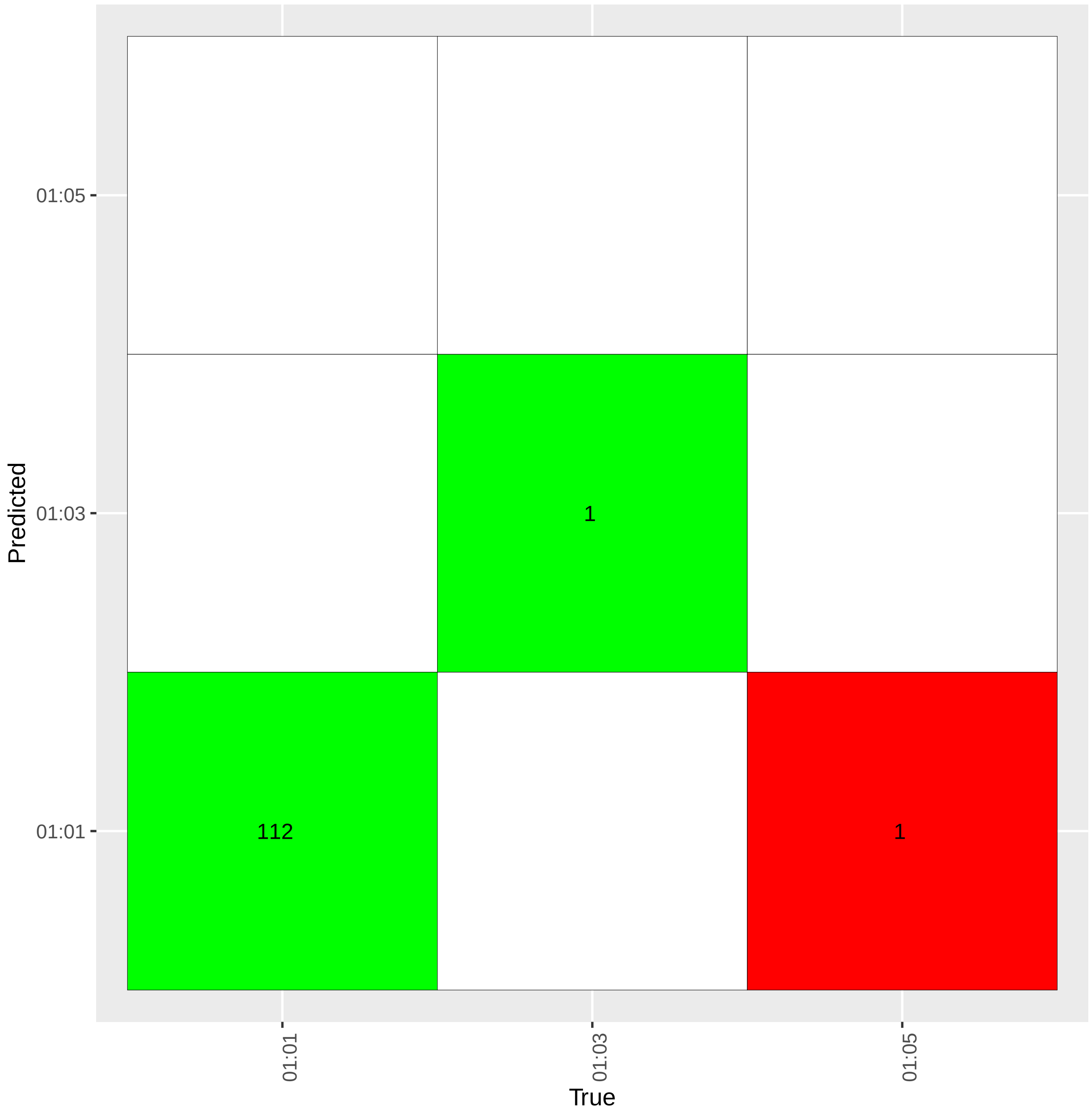

gene = HLA_F
model = v
model limit = NULL
pop = SAS

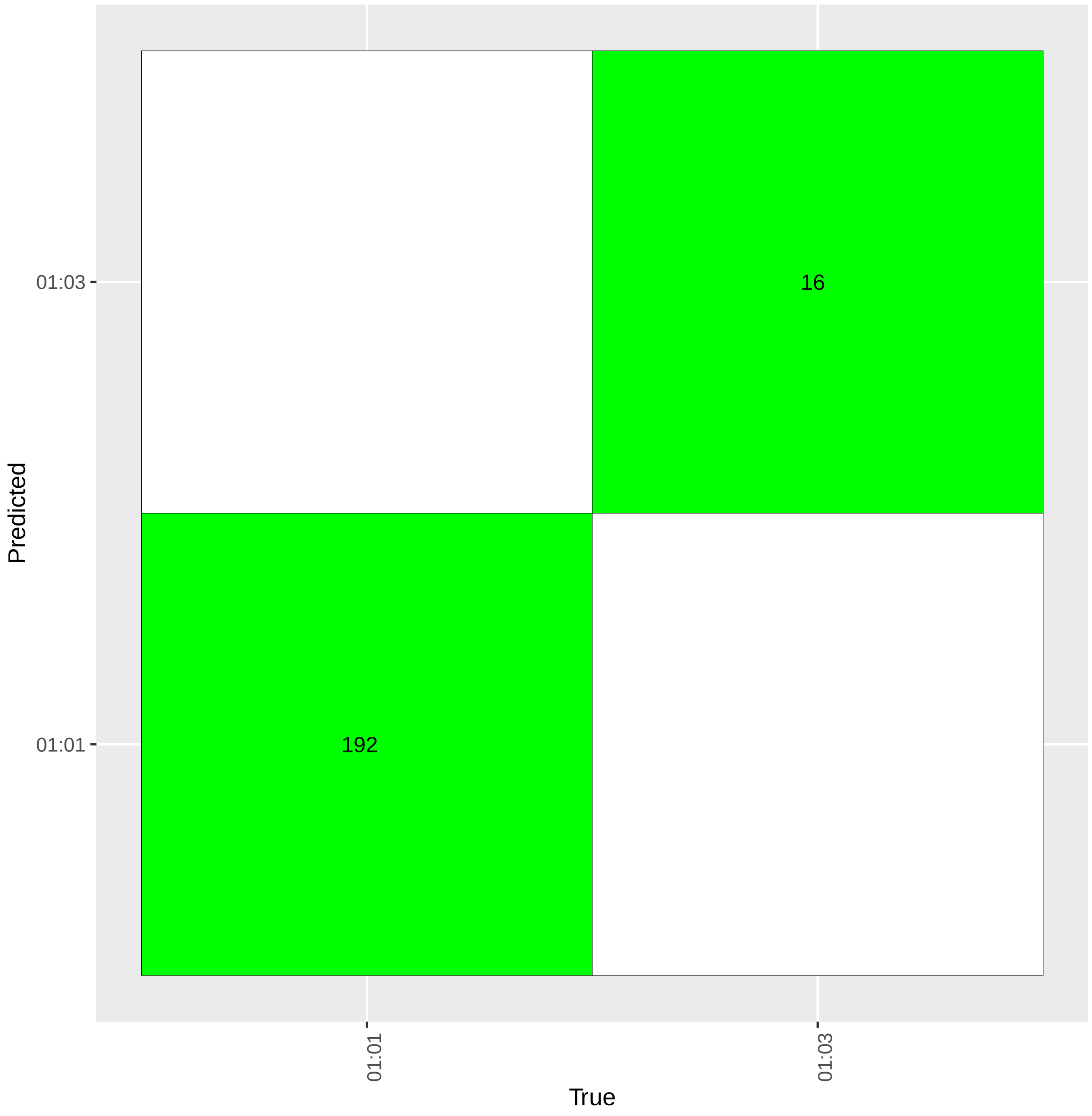

gene = HLA_F
model = v
model limit = NULL
pop = AMR

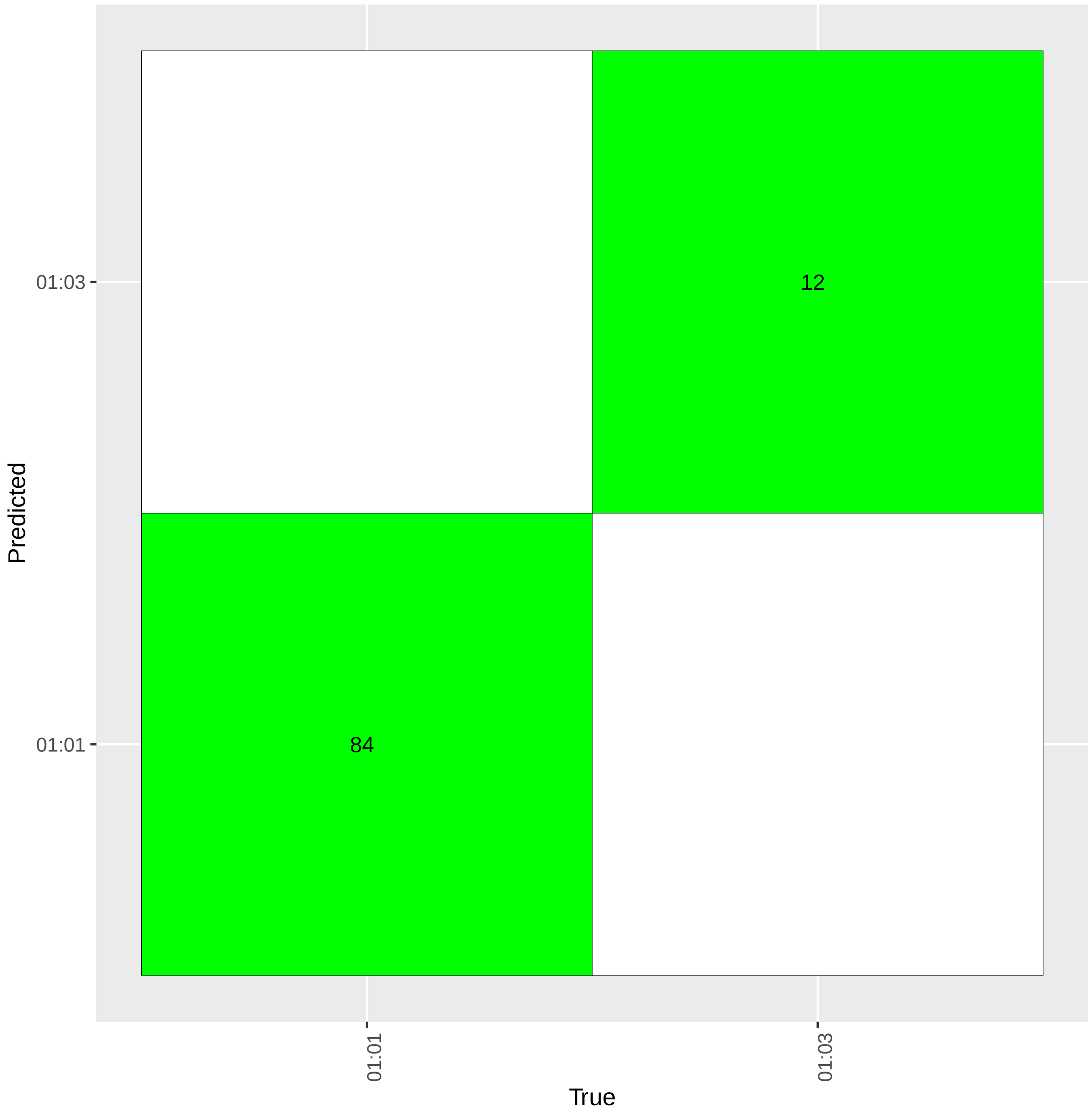

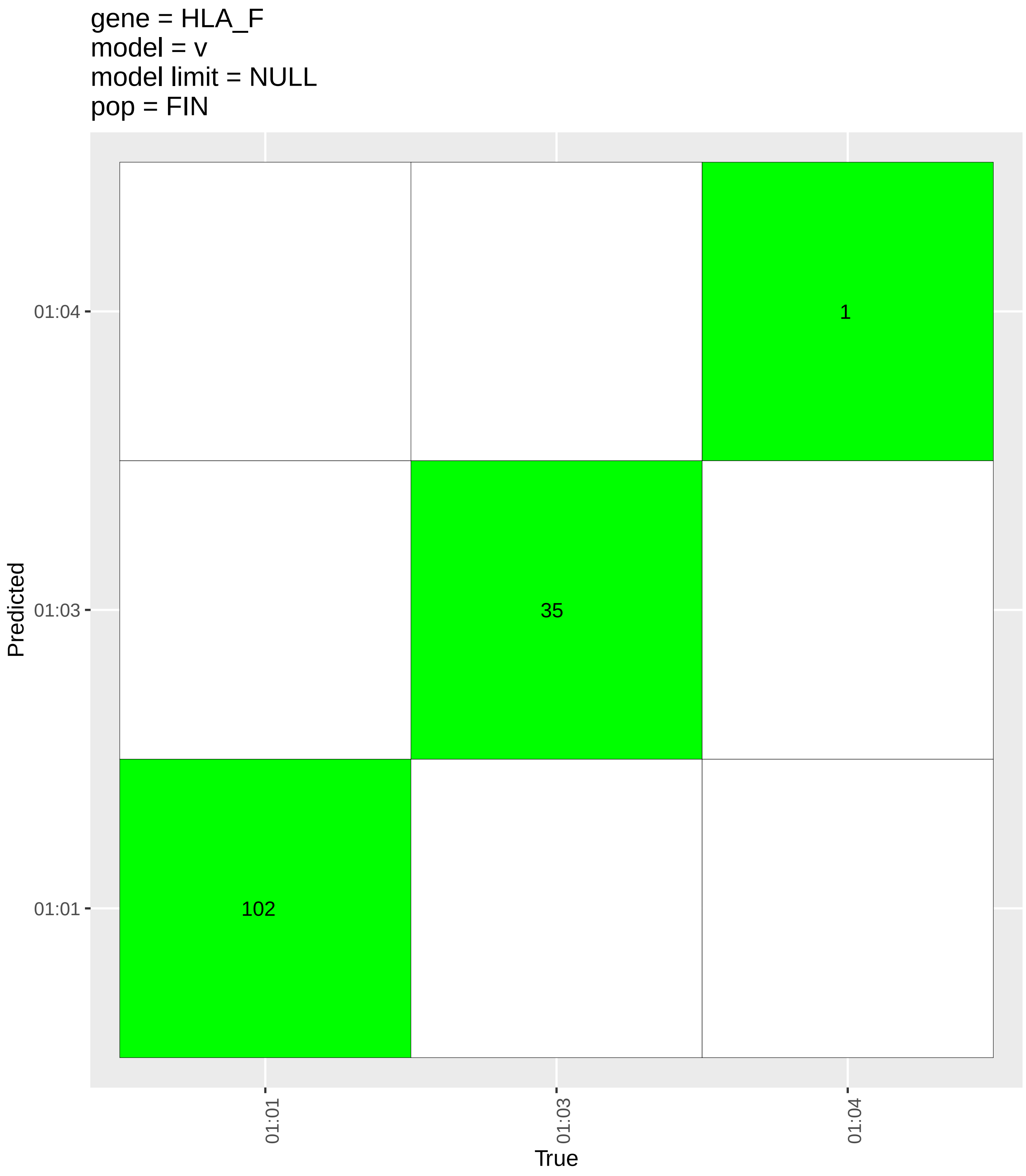
gene = HLA_F
model = v
model limit = NULL
pop = FIN
Predicted
01:04
01:03
01:01
1
35
102
01:01
01:03
01:04
True

gene = HLA_F
model = vi
model limit = NULL
pop = EUR

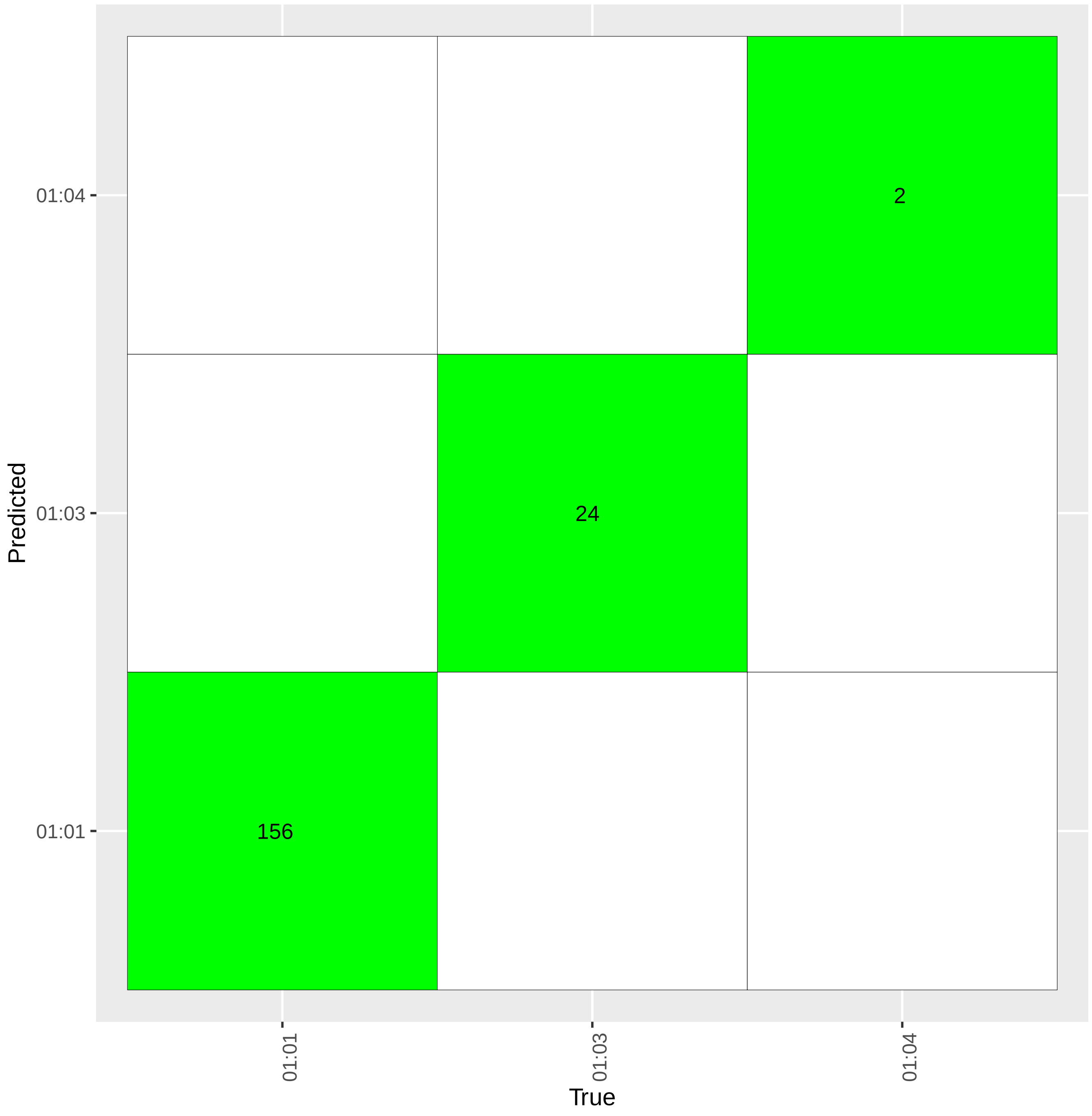

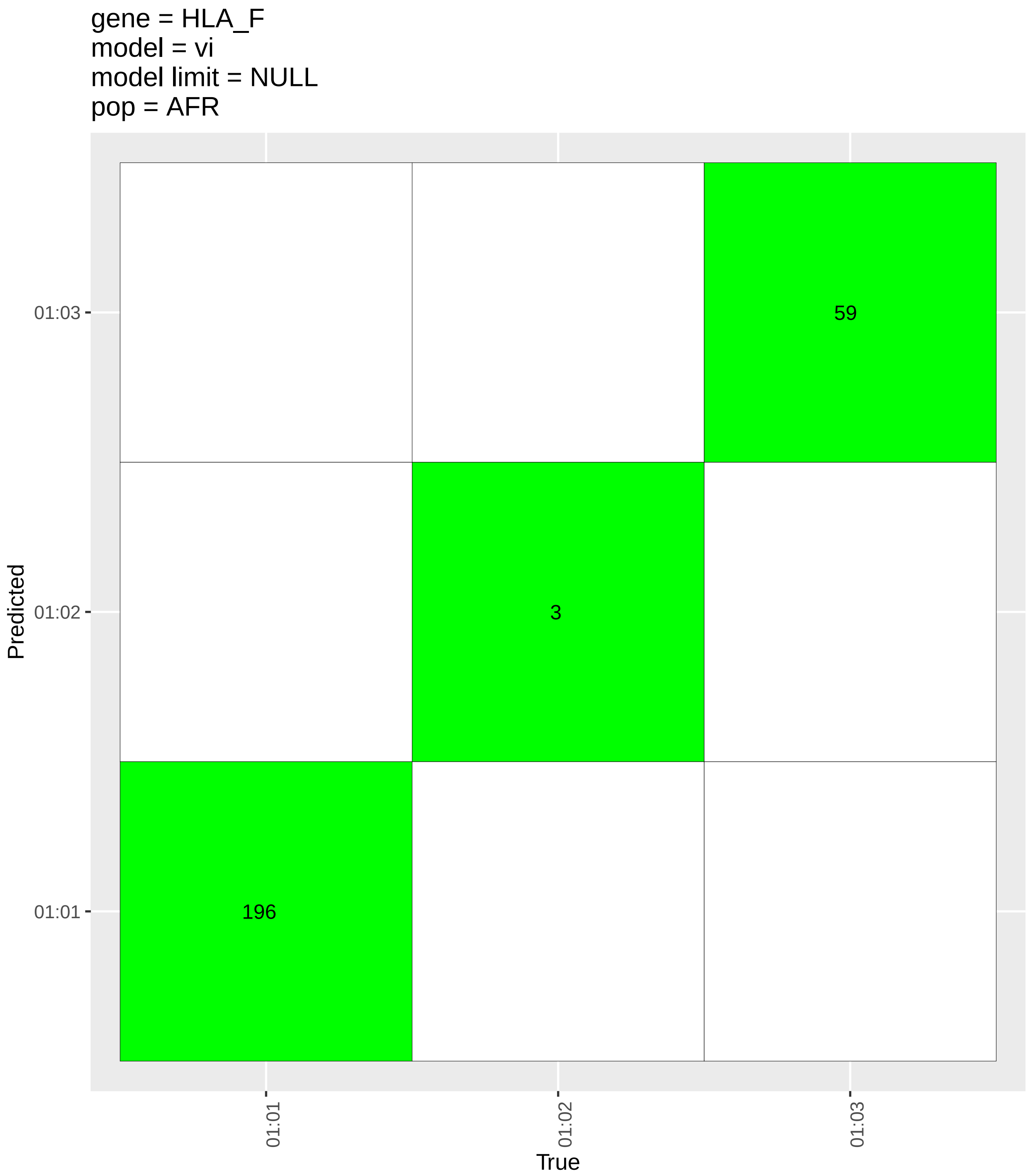
gene = HLA_F
model = vi
model limit = NULL
pop = AFR
Predicted
01:03
01:02
01:01
59
3
196
01:01
01:02
01:03
True

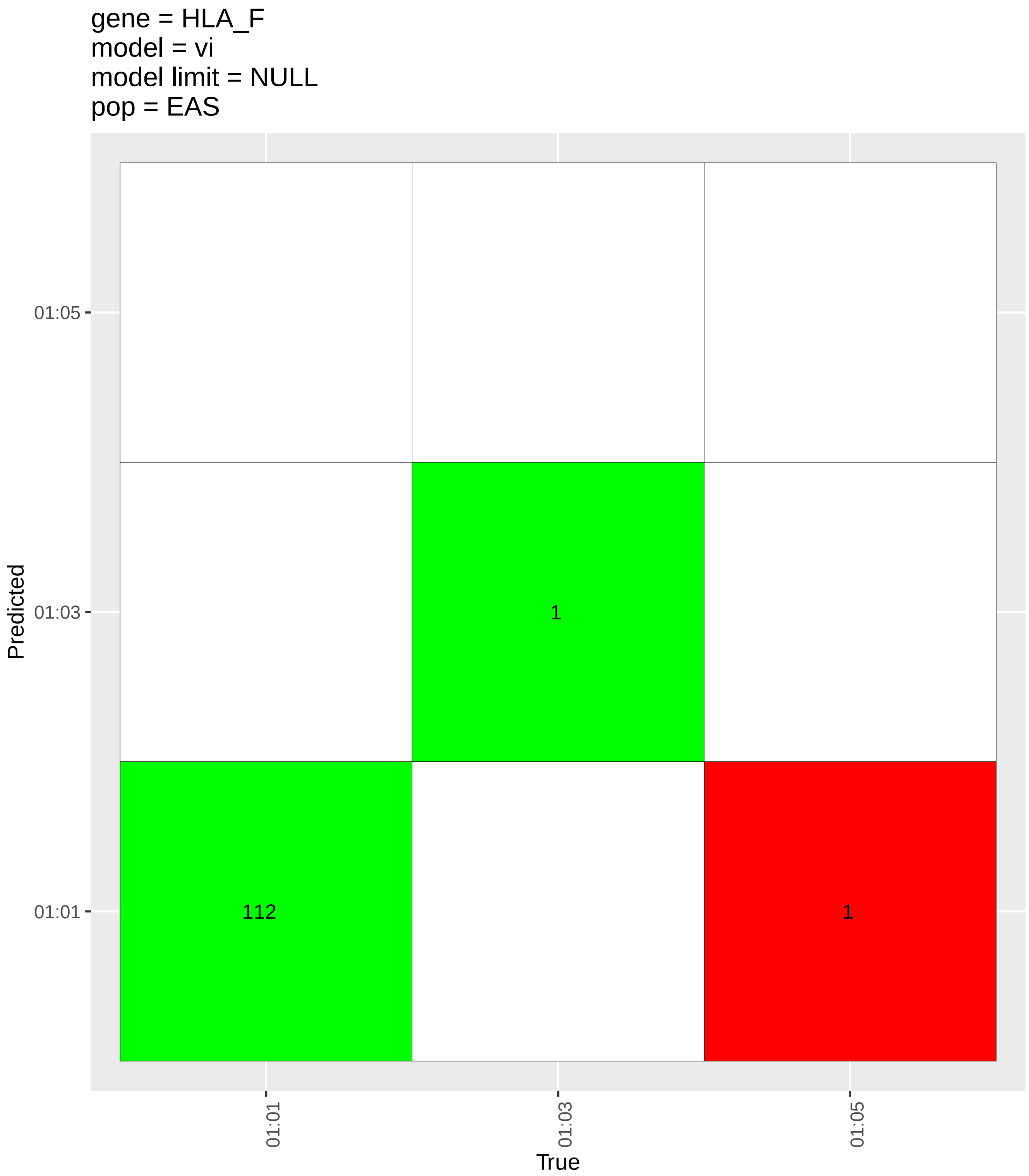
gene = HLA_F
model = vi
model limit = NULL
pop = EAS
Predicted
01:05
01:03
01:01
1
112
1
01:01
01:03
01:05
True

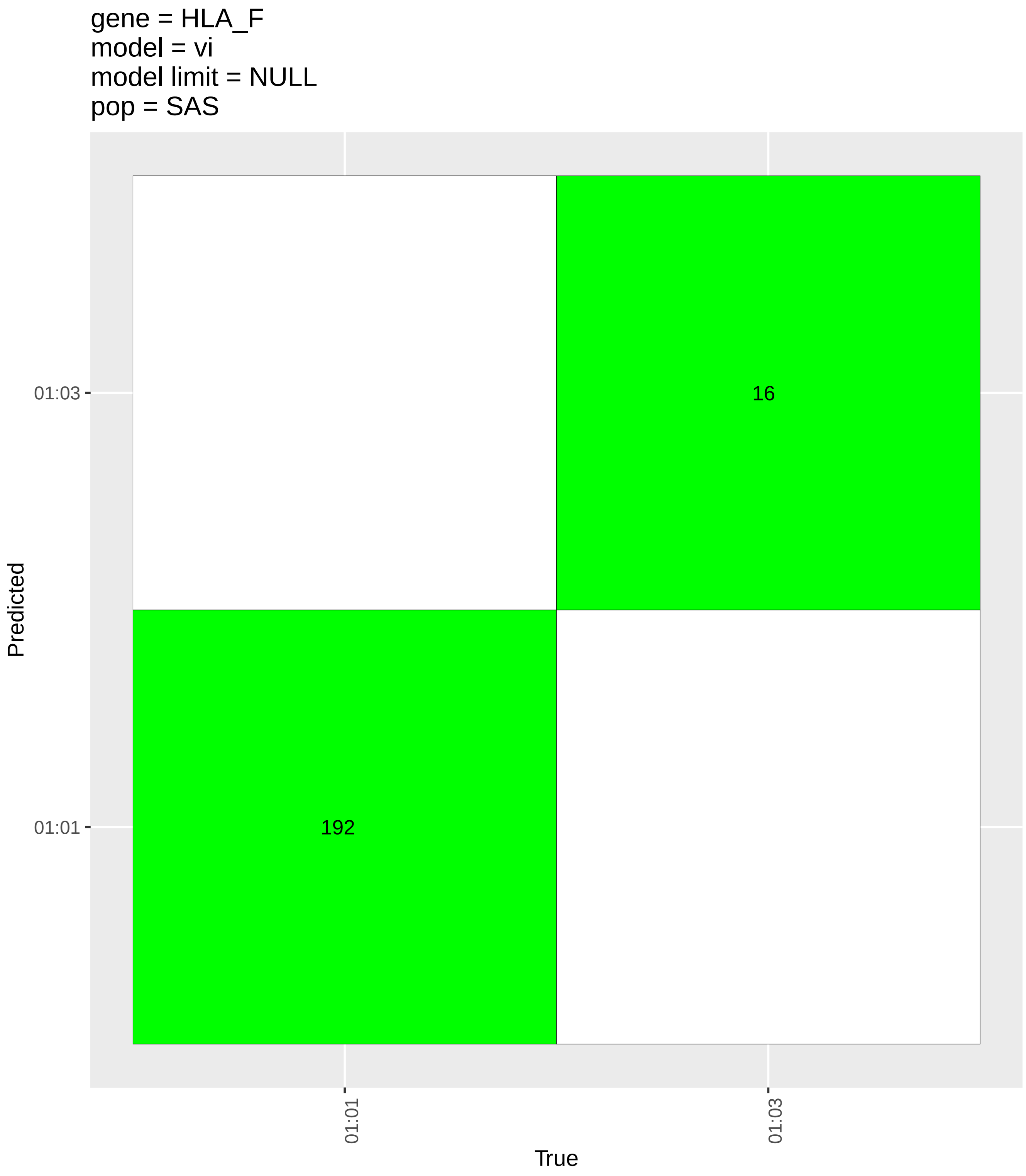
gene = HLA_F
model = vi
model limit = NULL
pop = SAS
16
192
01:03
01:01
Predicted
01:01
01:03
True

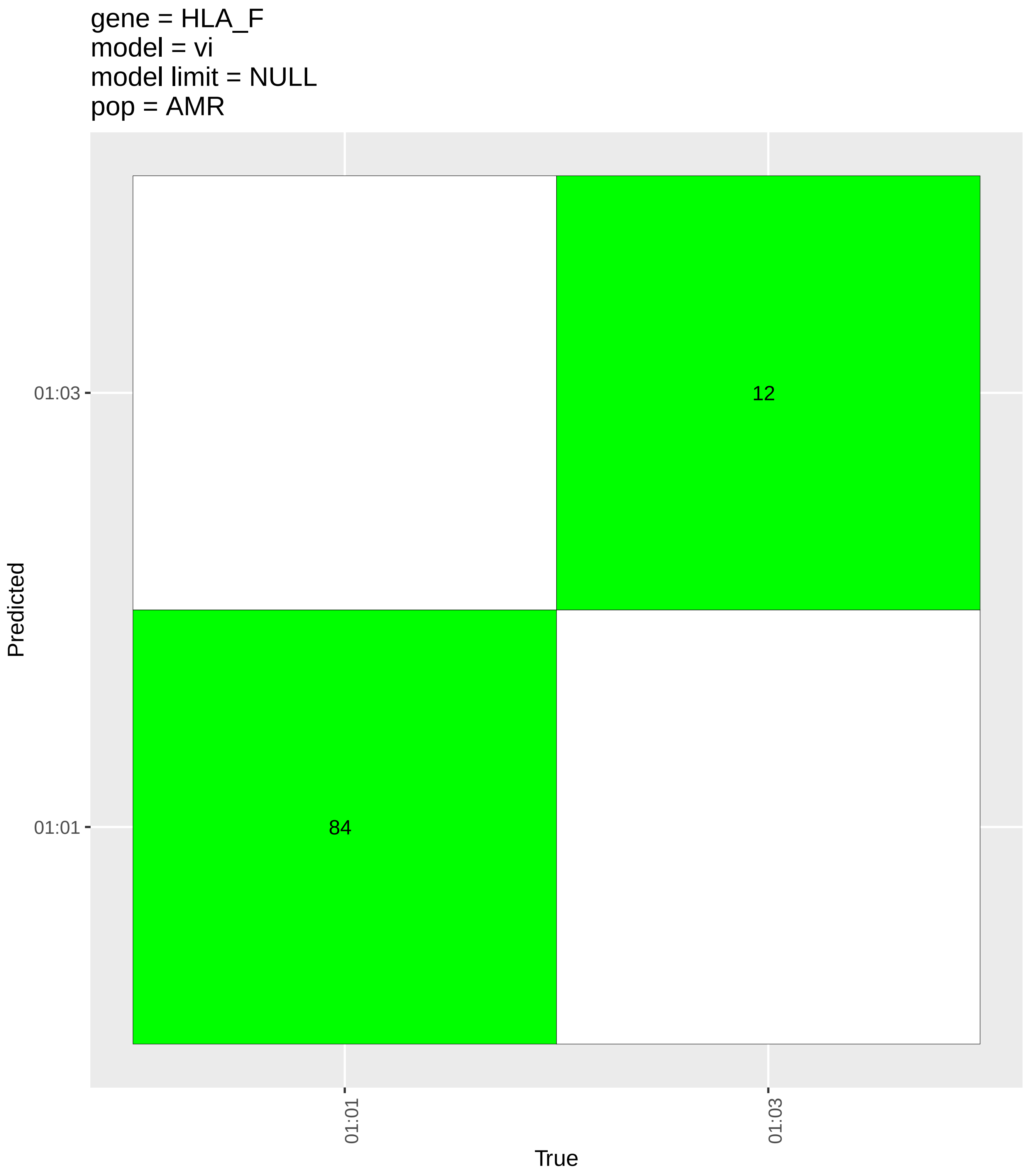
gene = HLA_F
model = vi
model limit = NULL
pop = AMR
12
84
01:03
01:01
Predicted
01:01
01:03
True

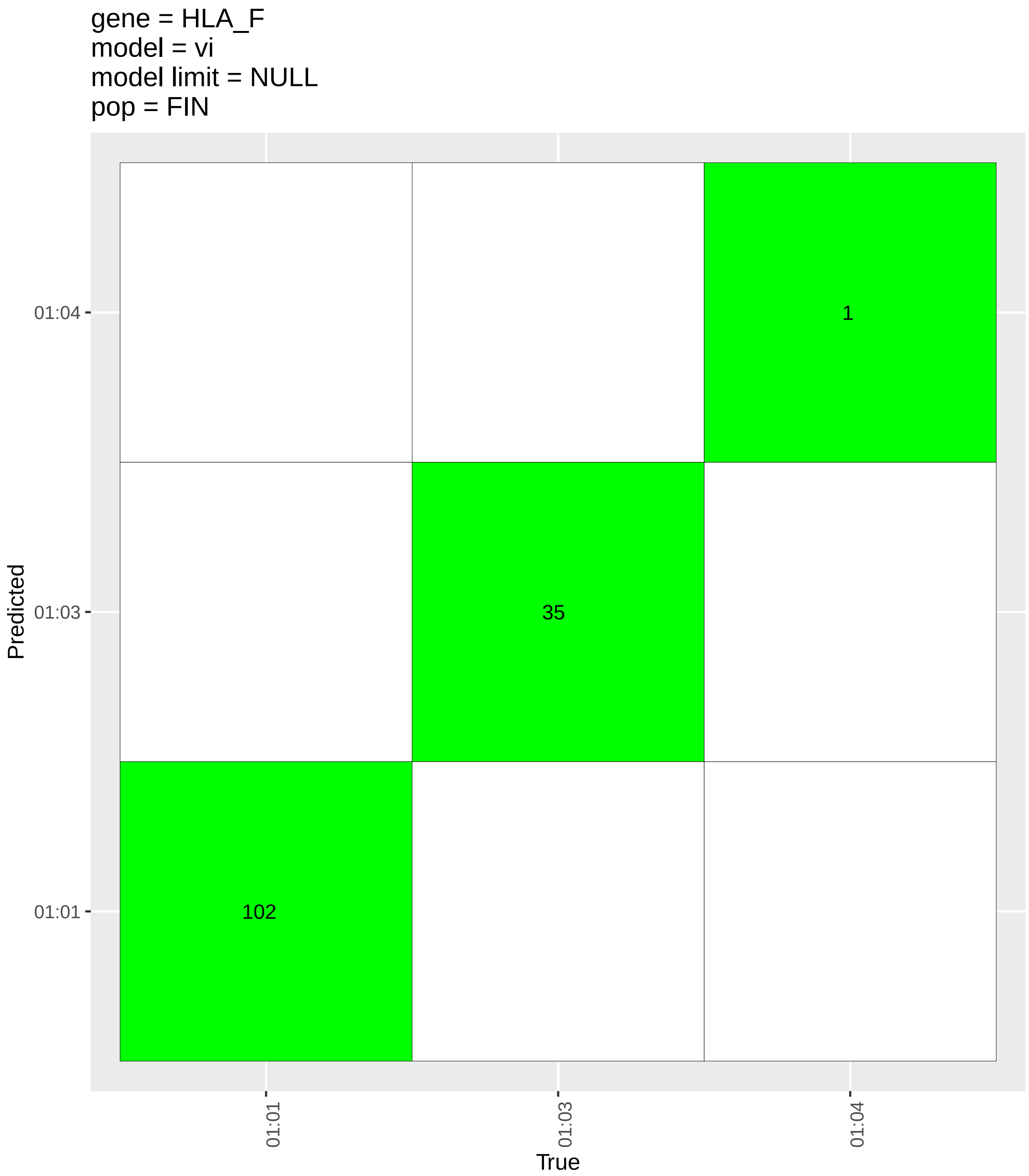
gene = HLA_F
model = vi
model limit = NULL
pop = FIN
Predicted
01:04
01:03
01:01
1
35
102
01:01
01:03
01:04
True

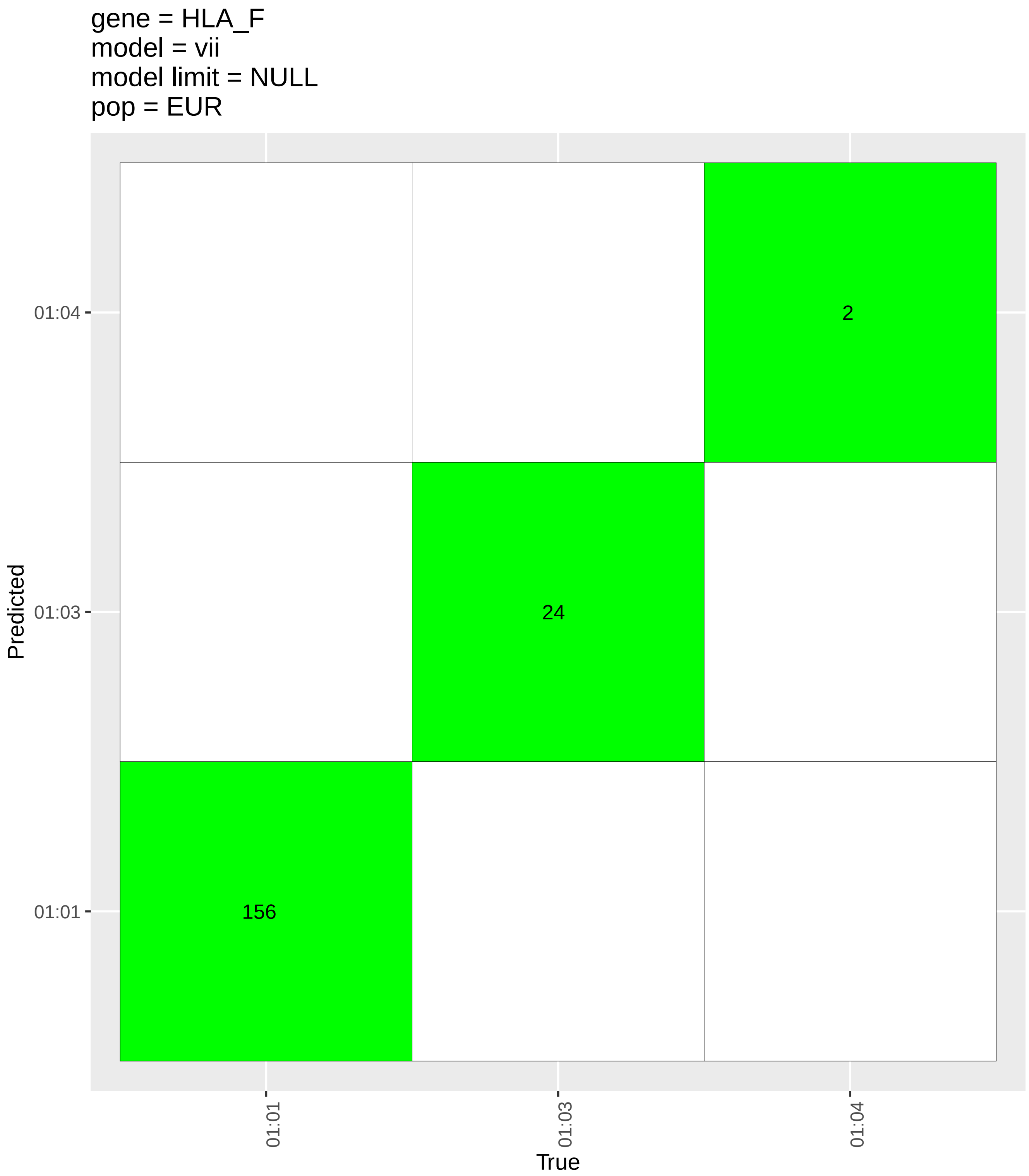
gene = HLA_F
model = vii
model limit = NULL
pop = EUR
Predicted
01:04
01:03
01:01
2
24
156
01:01
01:03
01:04
True

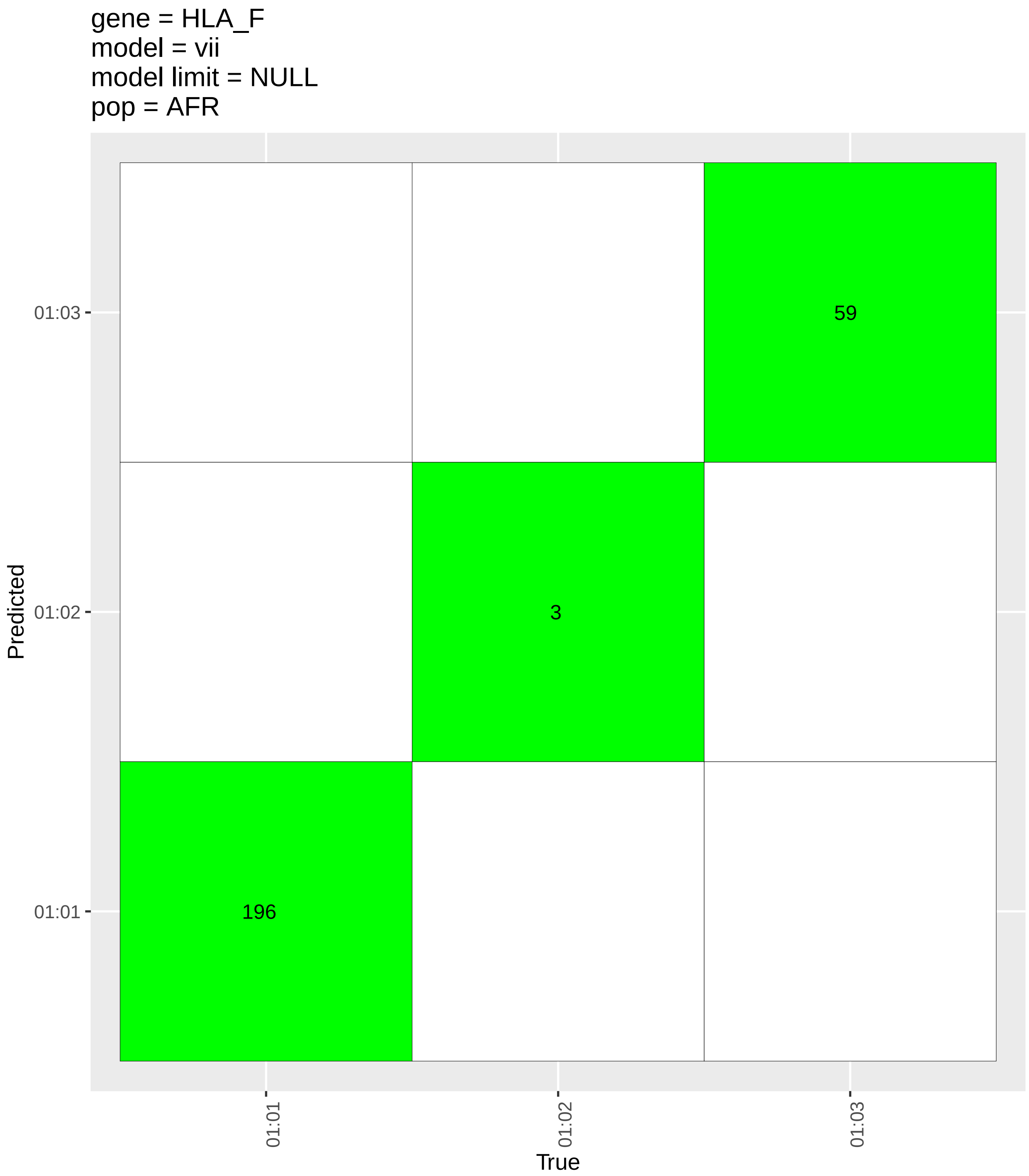
gene = HLA_F
model = vii
model limit = NULL
pop = AFR
Predicted
01:03
01:02
01:01
59
3
196
01:01
01:02
01:03
True

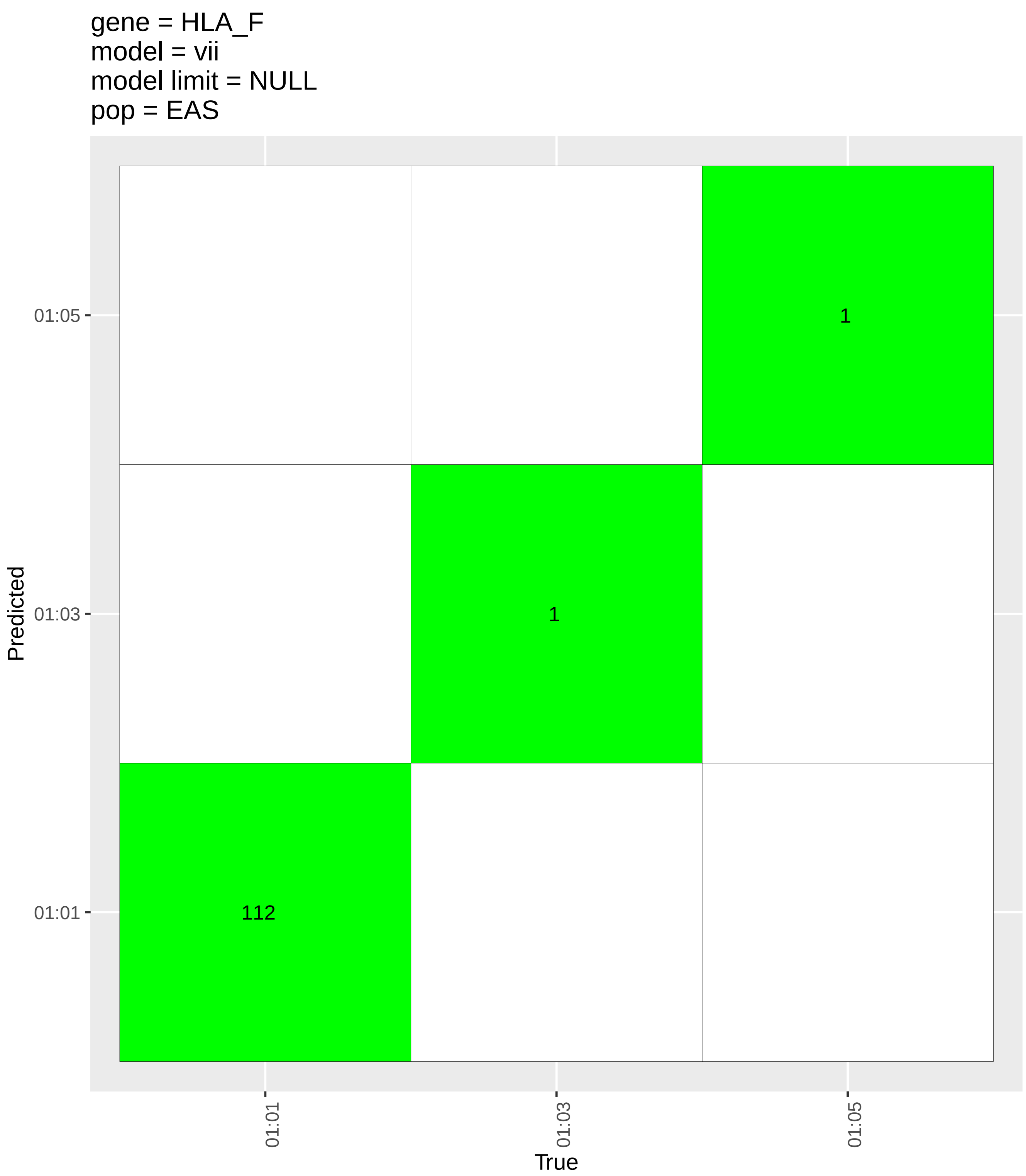
gene = HLA_F
model = vii
model limit = NULL
pop = EAS
Predicted
01:05
01:03
01:01
1
1
112
01:01
01:03
01:05
True

gene = HLA_F
model = vii
model limit = NULL
pop = SAS

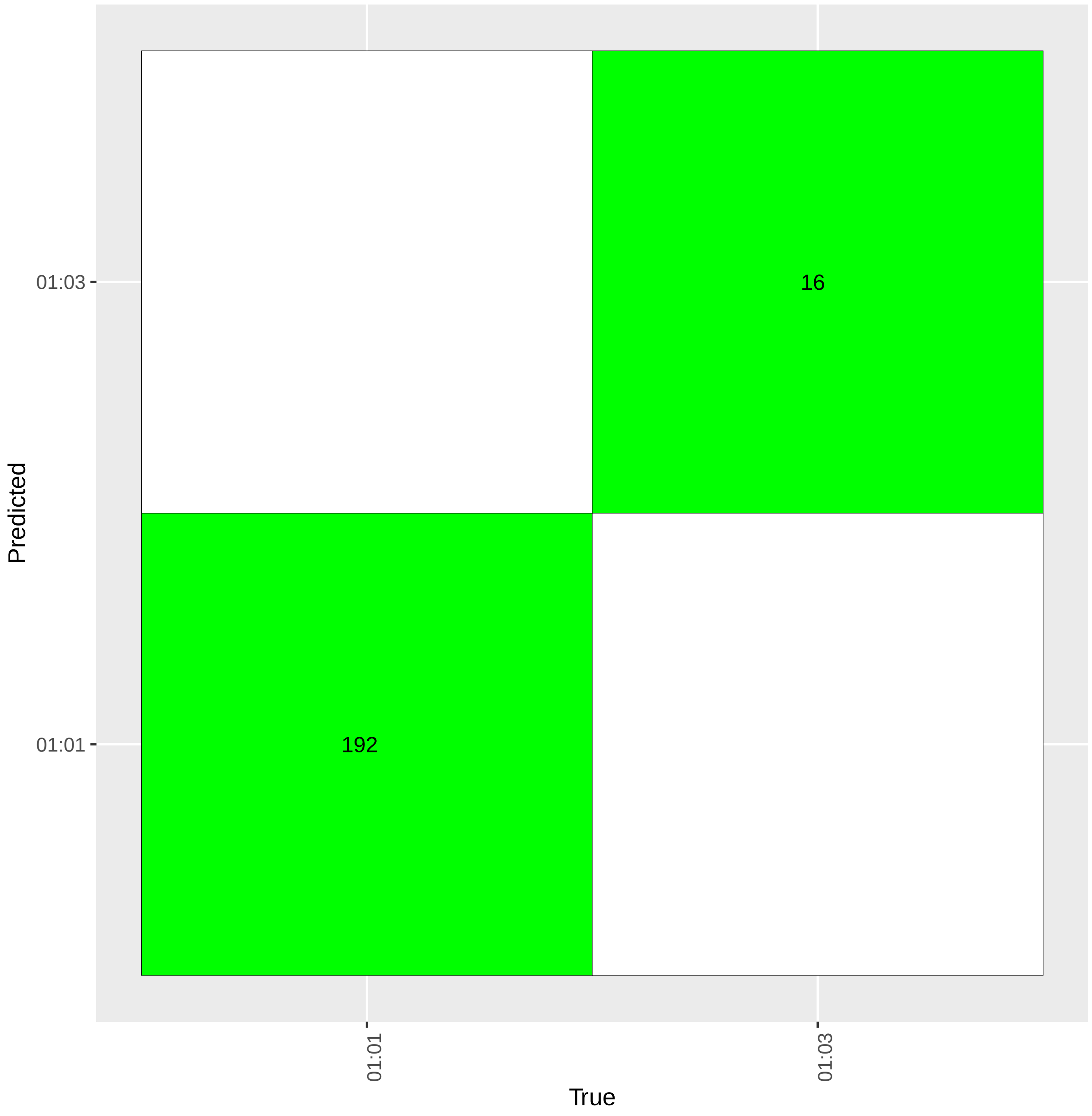

gene = HLA_F
model = vii
model limit = NULL
pop = AMR

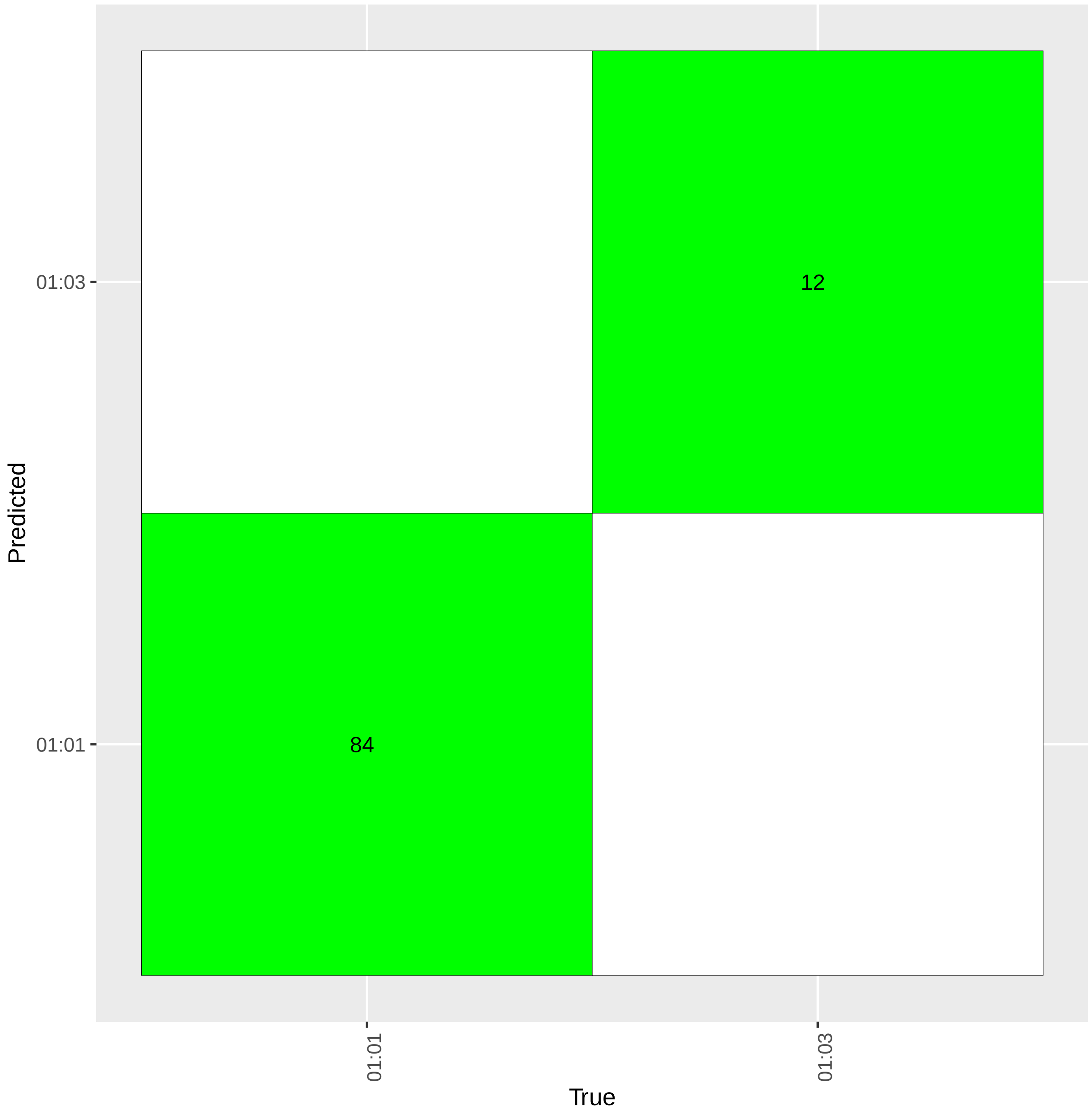

gene = HLA_F
model = vii
model limit = NULL
pop = FIN

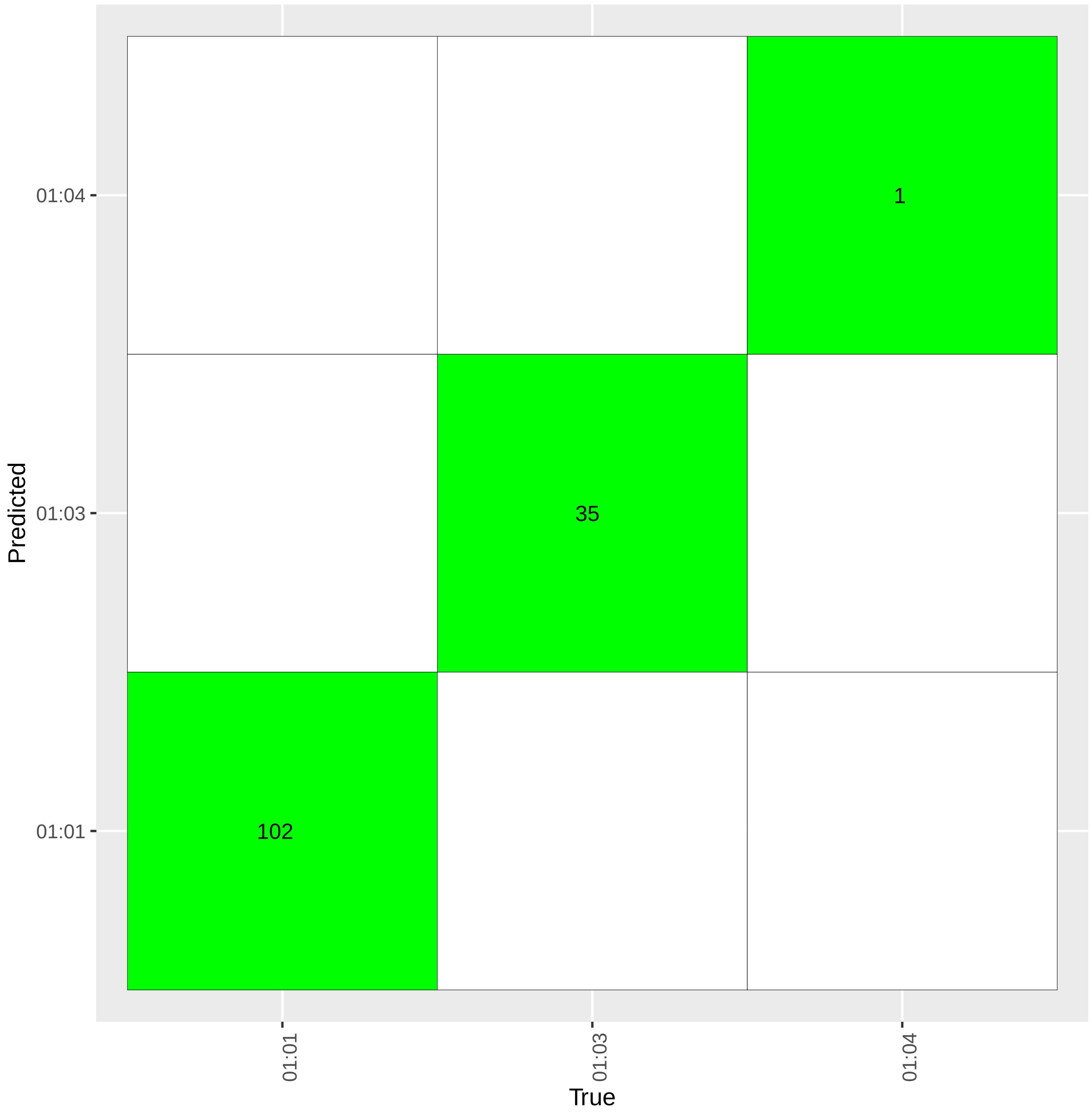

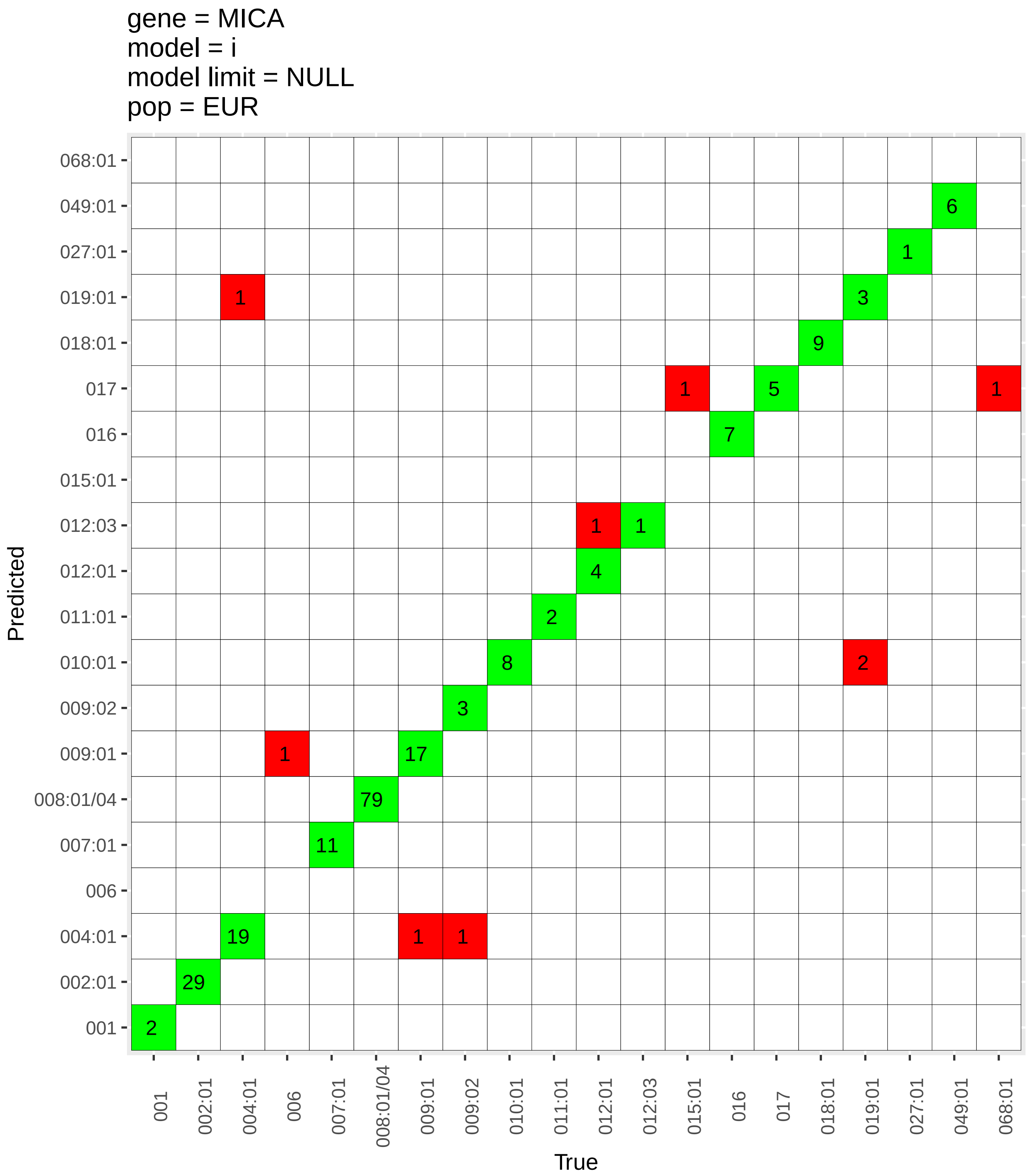
gene = MICA
model = i
model limit = NULL
pop = EUR
Predicted
068:01
049:01
027:01
019:01
018:01
017
016
015:01
012:03
012:01
011:01
010:01
009:02
009:01
008:01/04
007:01
006
004:01
002:01
001
True
001
002:01
004:01
006
007:01
008:01/04
009:01
009:02
010:01
011:01
012:01
012:03
015:01
016
017
018:01
019:01
027:01
049:01
068:01
6
1
1
3
9
1
5
1
7
1
1
4
2
8
2
3
1
17
79
11
19
1
1
29
2

gene = MICA
model = i
model limit = NULL
pop = AFR

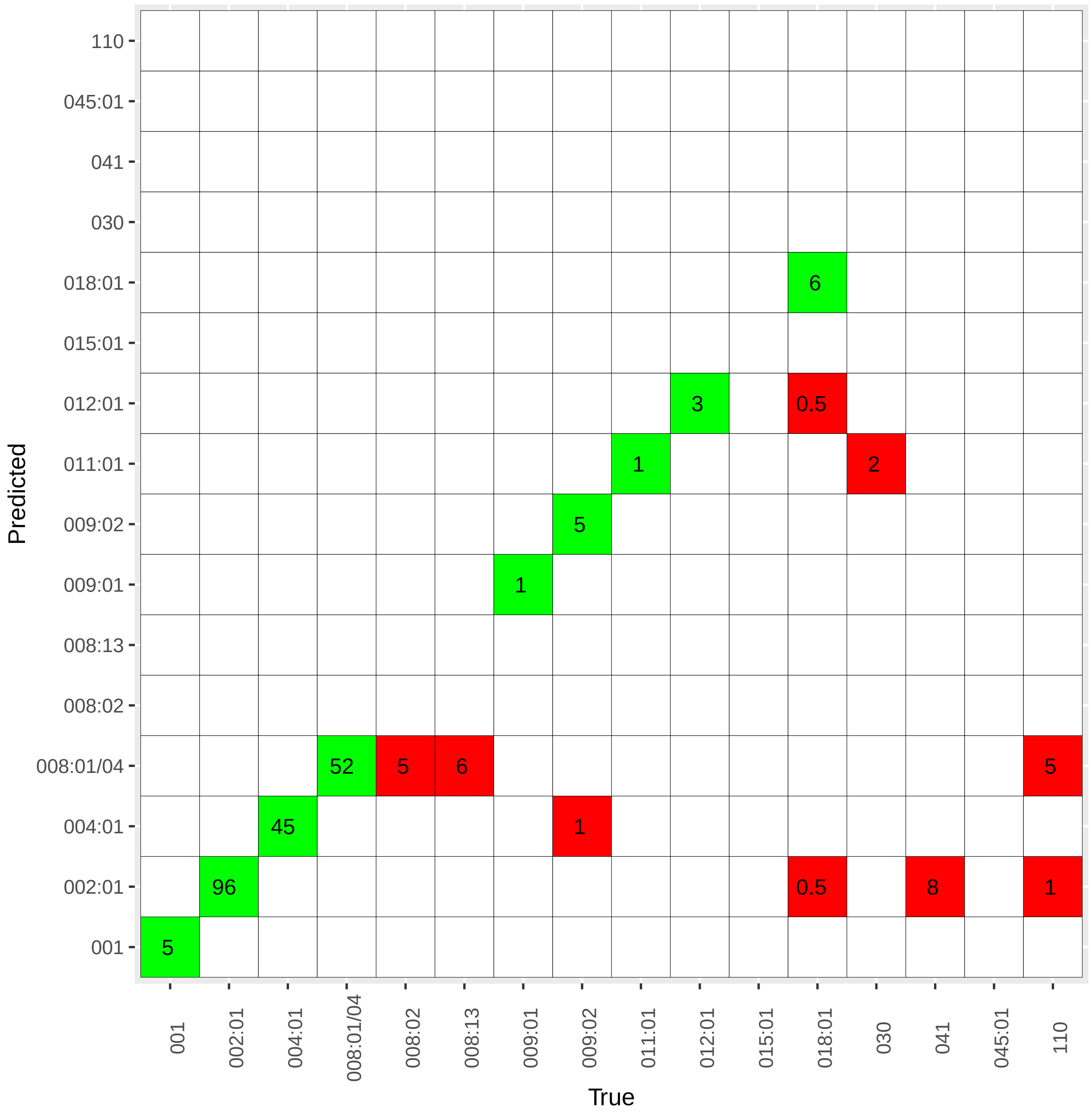

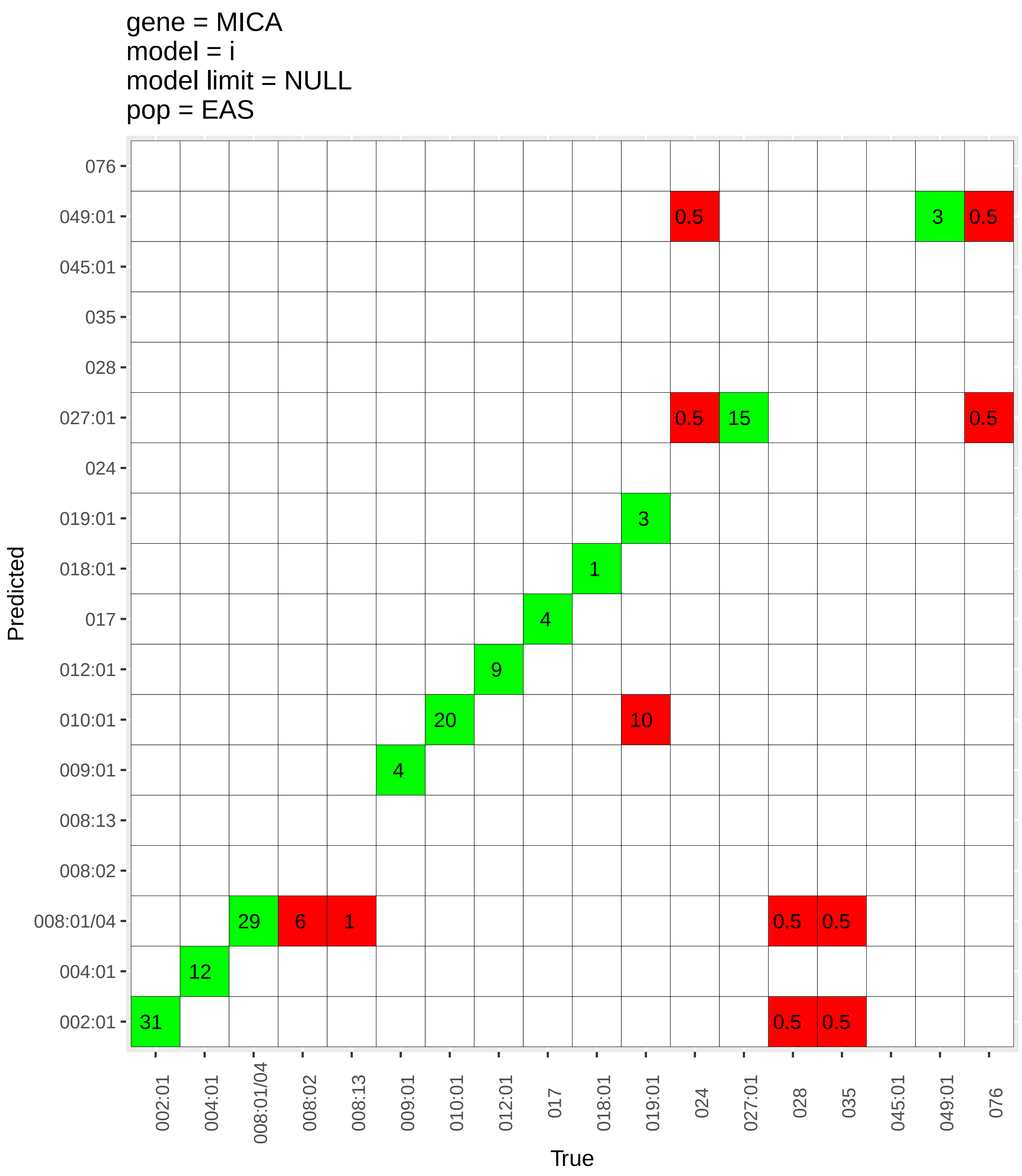
gene = MICA
model = i
model limit = NULL
pop = EAS
Predicted
True
076
049:01
045:01
035
028
027:01
024
019:01
018:01
017
012:01
010:01
009:01
008:13
008:02
008:01/04
004:01
002:01
0.5
3
0.5
0.5
15
0.5
3
1
4
9
20
10
4
29
6
1
0.5
0.5
12
31
0.5
0.5

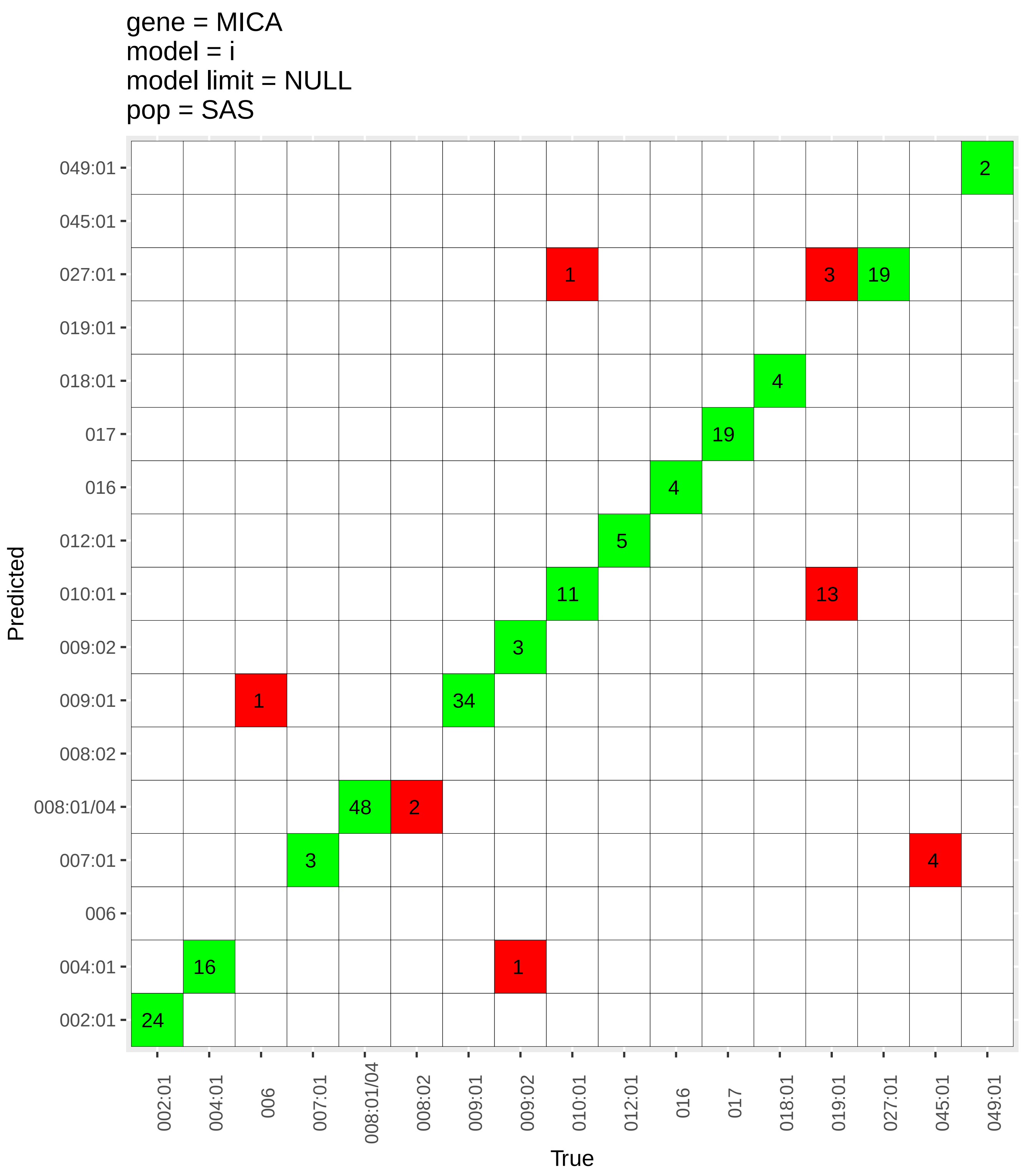
gene = MICA
model = i
model limit = NULL
pop = SAS
Predicted
049:01
045:01
027:01
019:01
018:01
017
016
012:01
010:01
009:02
009:01
008:02
008:01/04
007:01
006
004:01
002:01
2
1
3
19
4
19
4
5
11
13
3
1
34
48
2
3
4
16
1
24
002:01
004:01
006
007:01
008:01/04
008:02
009:01
009:02
010:01
012:01
016
017
018:01
019:01
027:01
045:01
049:01
True

gene = MICA
model = i
model limit = NULL
pop = AMR

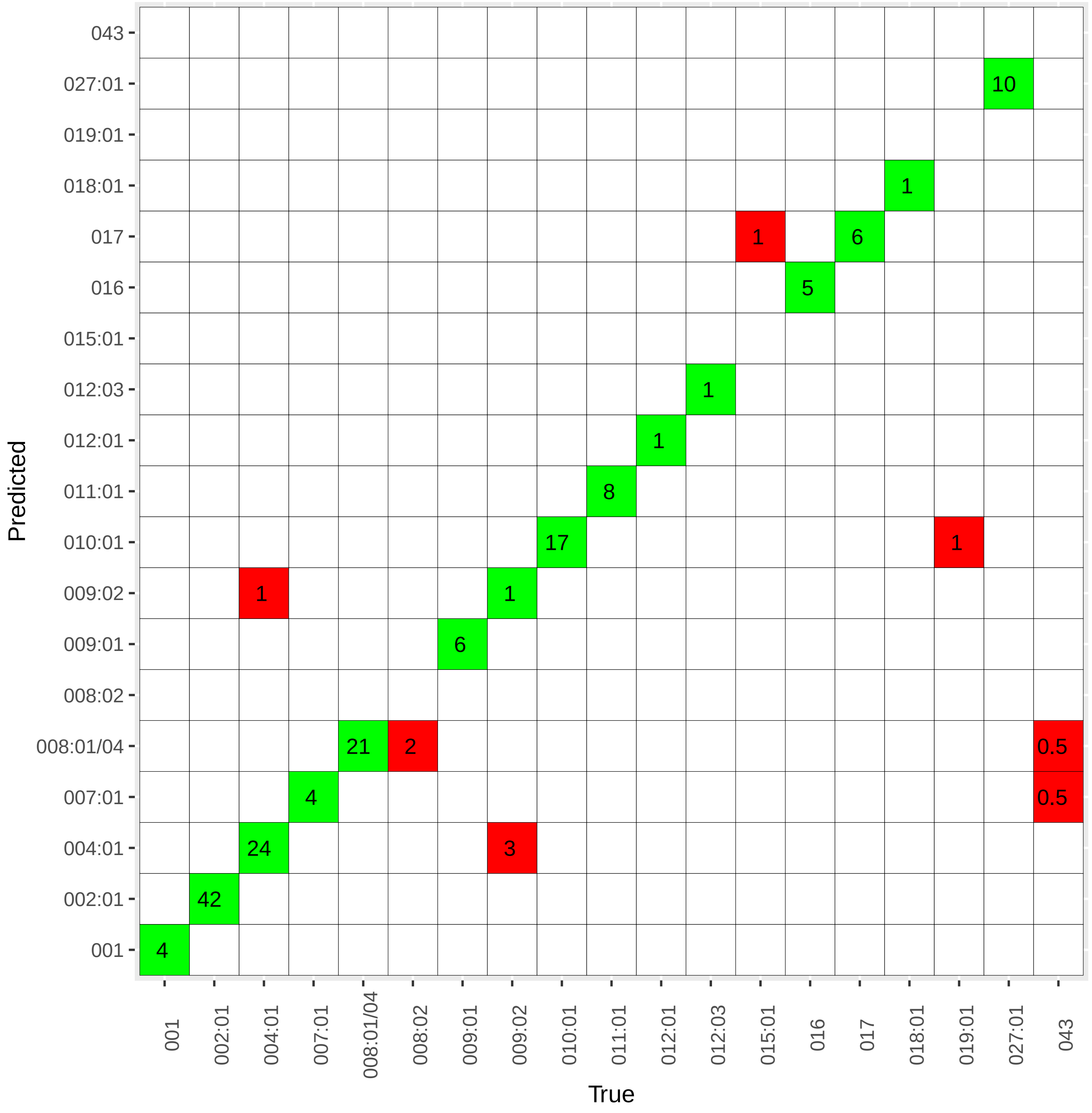

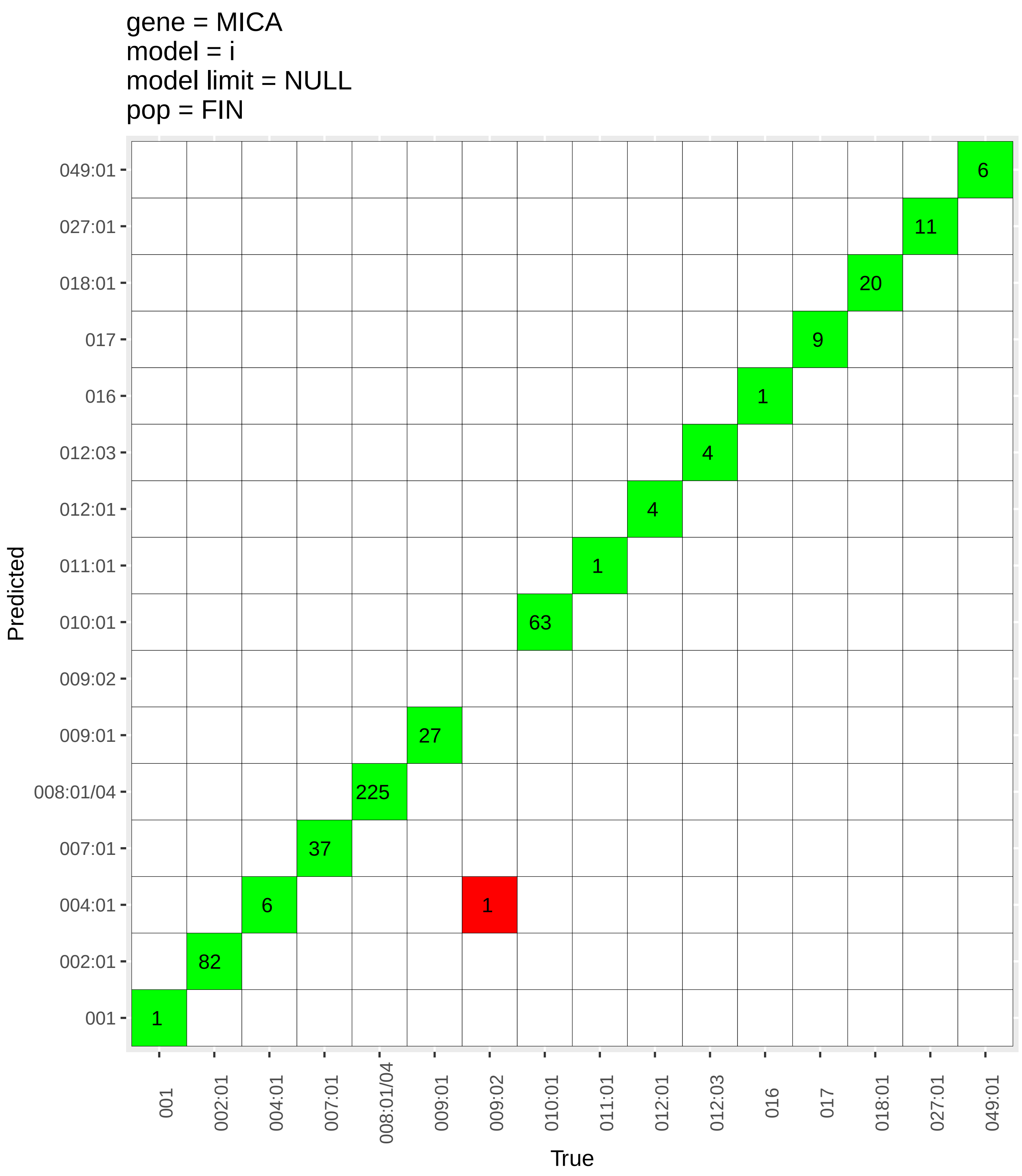
gene = MICA
model = i
model limit = NULL
pop = FIN
Predicted
049:01
027:01
018:01
017
016
012:03
012:01
011:01
010:01
009:02
009:01
008:01/04
007:01
004:01
002:01
001
6
11
20
9
1
4
4
1
63
27
225
37
6
1
82
1
001
002:01
004:01
007:01
008:01/04
009:01
009:02
010:01
011:01
012:01
012:03
016
017
018:01
027:01
049:01
True

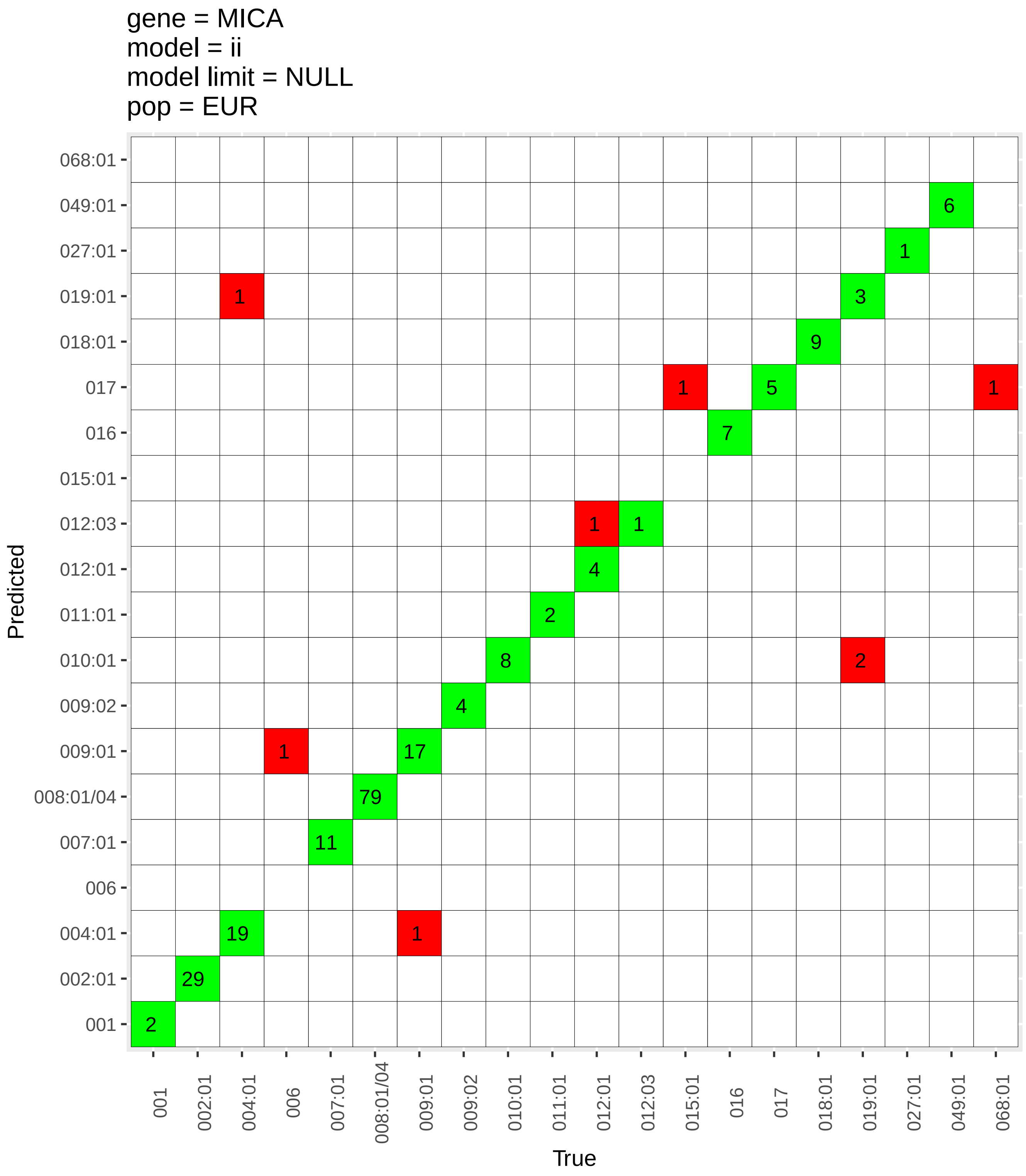
gene = MICA
model = ii
model limit = NULL
pop = EUR
Predicted
068:01
049:01
027:01
019:01
018:01
017
016
015:01
012:03
012:01
011:01
010:01
009:02
009:01
008:01/04
007:01
006
004:01
002:01
001
True
001
002:01
004:01
006
007:01
008:01/04
009:01
009:02
010:01
011:01
012:01
012:03
015:01
016
017
018:01
019:01
027:01
049:01
068:01
6
1
1
3
9
1
5
1
7
1
1
4
2
8
2
4
1
17
79
11
19
1
29
2

gene = MICA
model = ii
model limit = NULL
pop = AFR

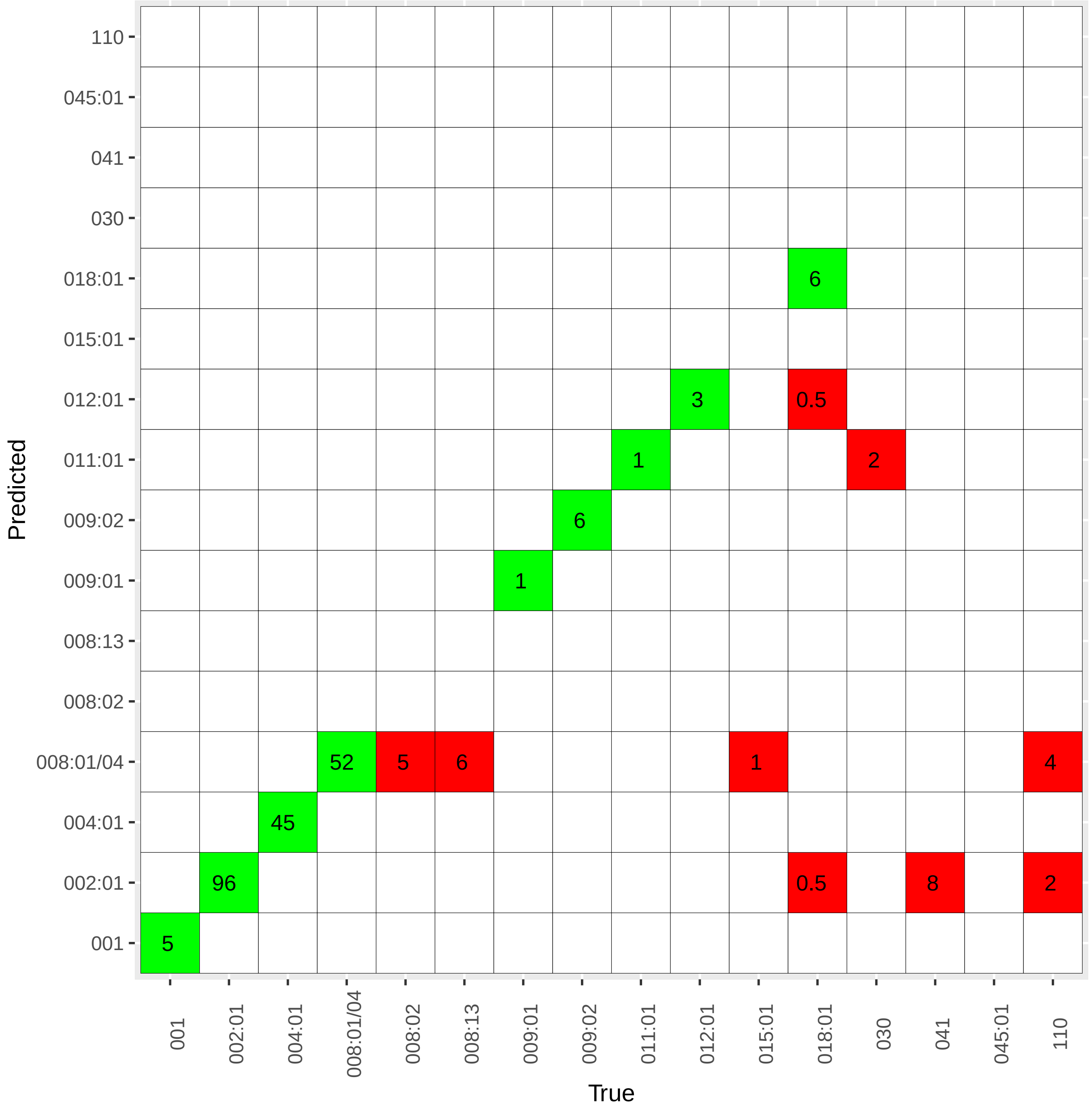

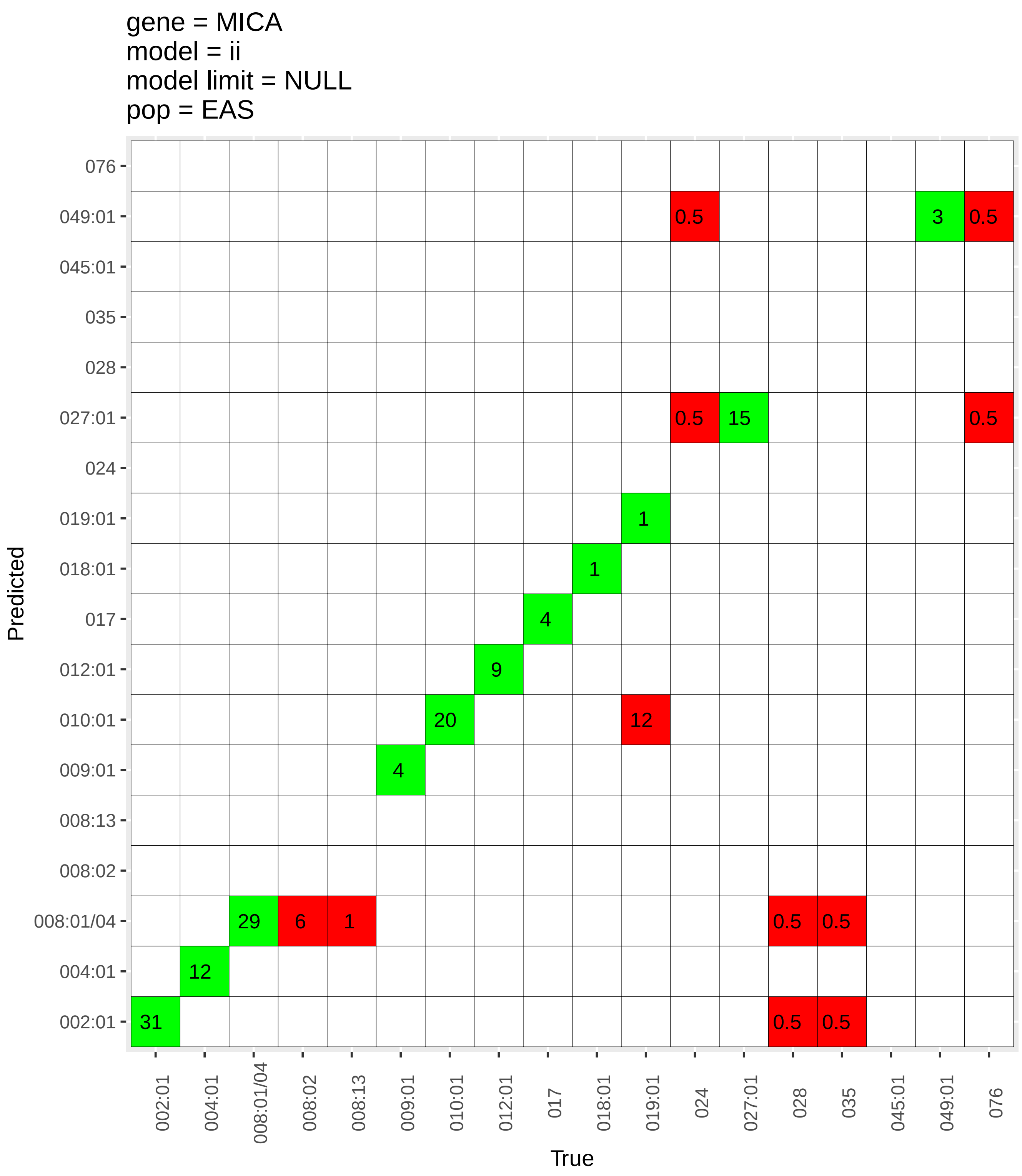
gene = MICA
model = ii
model limit = NULL
pop = EAS
Predicted
True
076
049:01
045:01
035
028
027:01
024
019:01
018:01
017
012:01
010:01
009:01
008:13
008:02
008:01/04
004:01
002:01
0.5
3
0.5
0.5
15
0.5
1
1
4
9
20
12
4
29
6
1
0.5
0.5
12
31
0.5
0.5

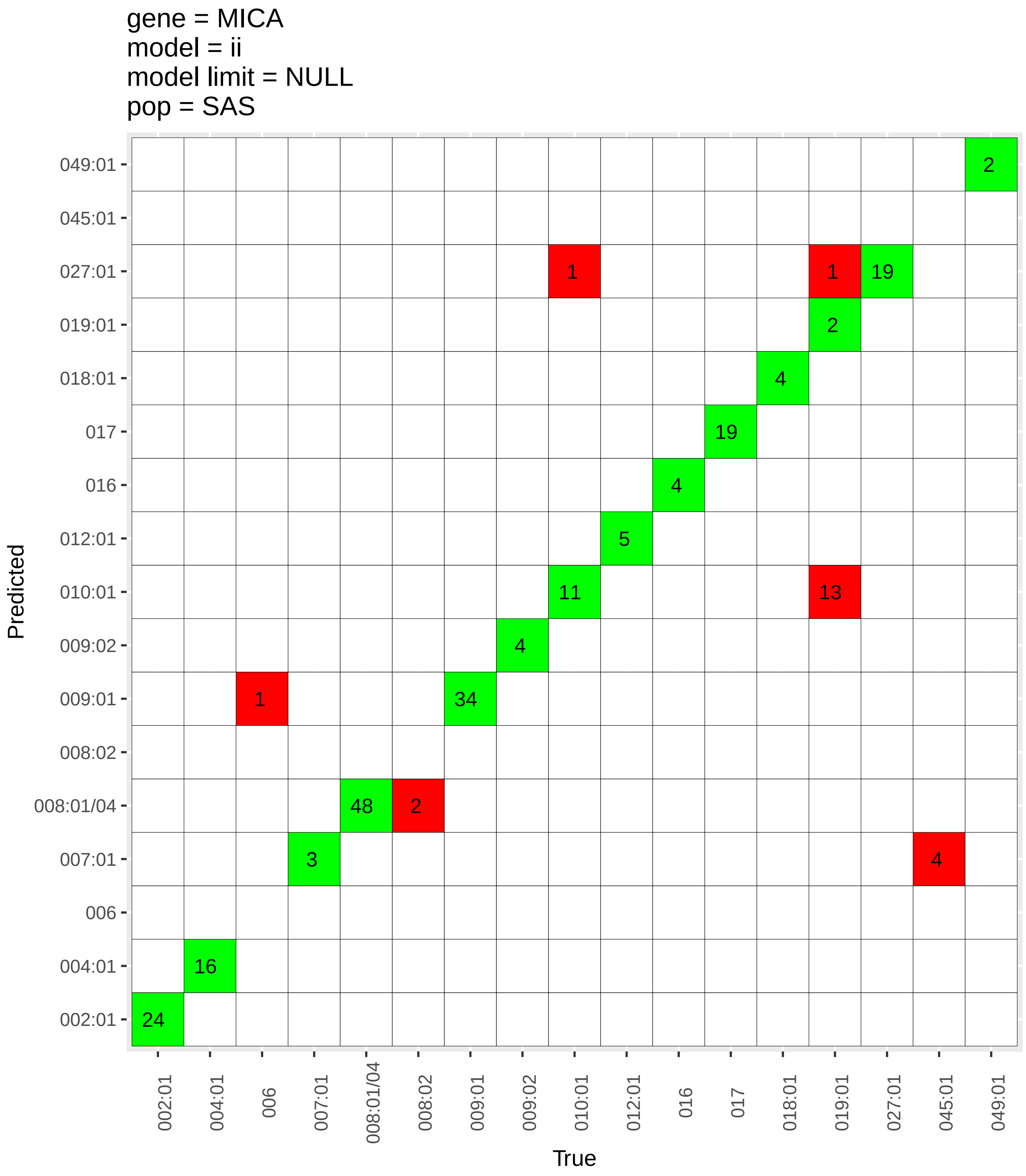
gene = MICA
model = ii
model limit = NULL
pop = SAS
Predicted
049:01
045:01
027:01
019:01
018:01
017
016
012:01
010:01
009:02
009:01
008:02
008:01/04
007:01
006
004:01
002:01
002:01
004:01
006
007:01
008:01/04
008:02
009:01
009:02
010:01
012:01
016
017
018:01
019:01
027:01
045:01
049:01
True

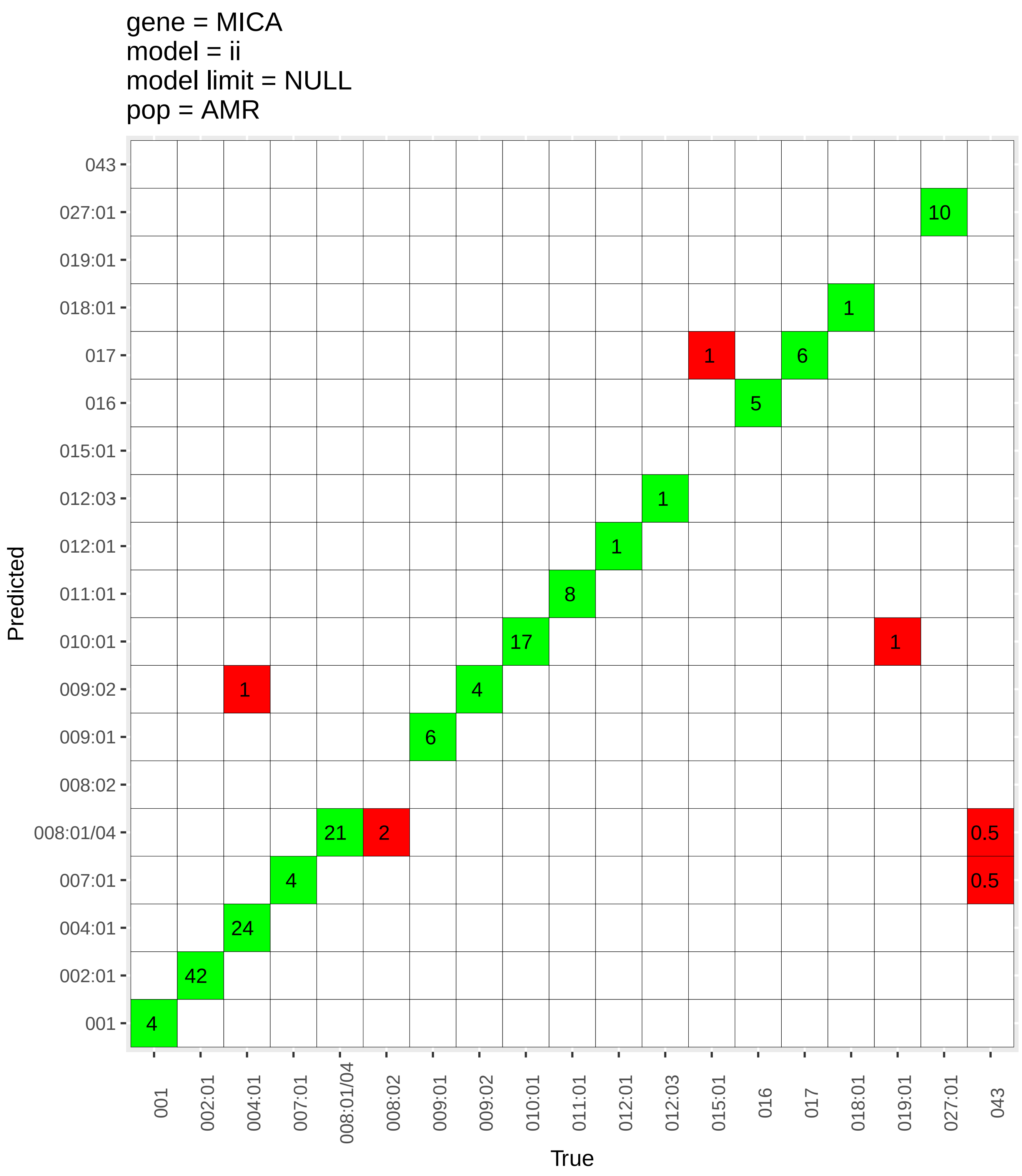
gene = MICA
model = ii
model limit = NULL
pop = AMR
Predicted
True
043
027:01
019:01
018:01
017
016
015:01
012:03
012:01
011:01
010:01
009:02
009:01
008:02
008:01/04
007:01
004:01
002:01
001
10
1
1
6
5
1
1
8
17
1
1
4
6
21
2
0.5
4
0.5
24
42
4

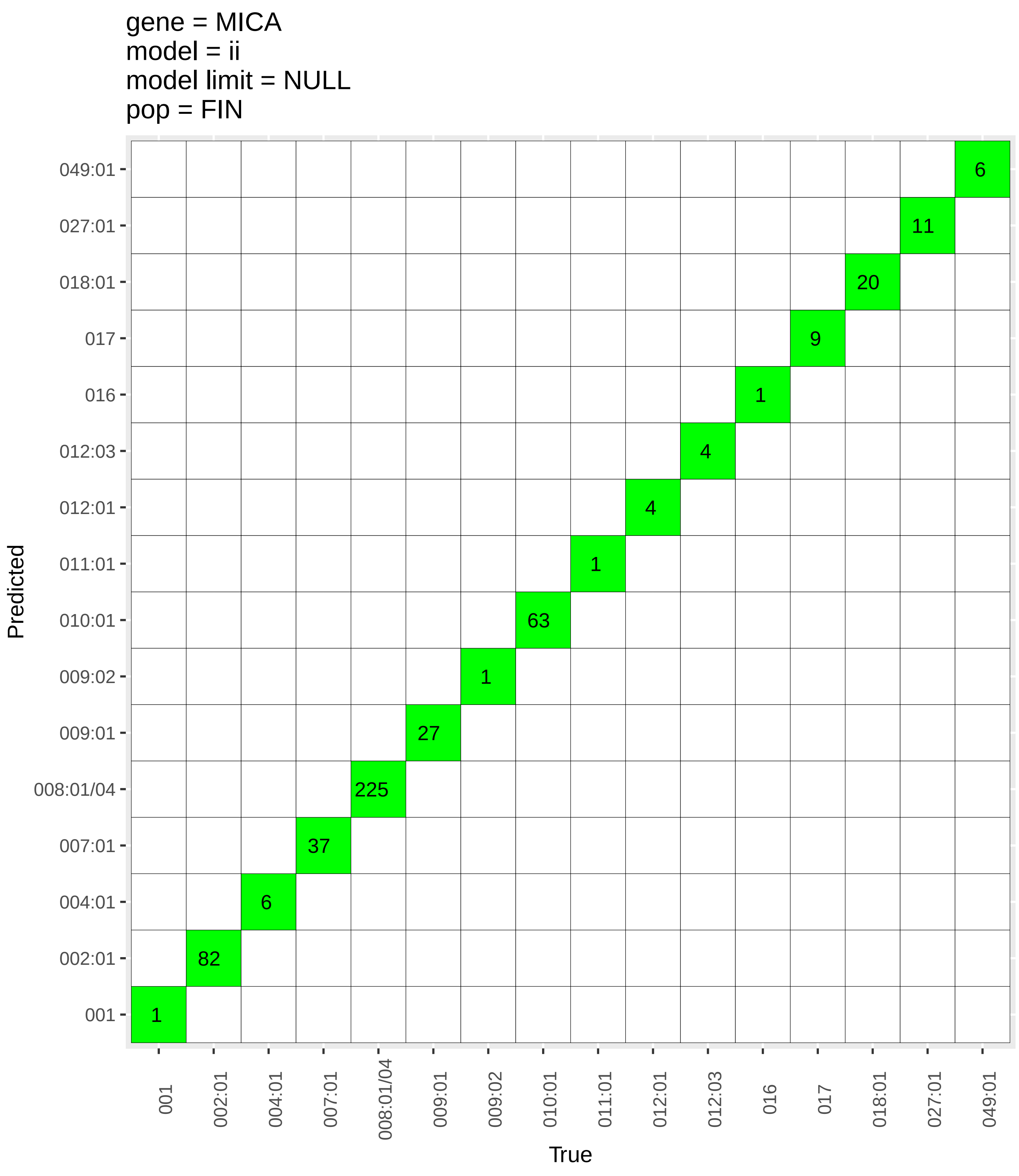

gene = MICA
model = ii
model limit = NULL
pop = FIN
Predicted
049:01
027:01
018:01
017
016
012:03
012:01
011:01
010:01
009:02
009:01
008:01/04
007:01
004:01
002:01
001
1
82
6
37
225
27
1
63
1
4
4
1
9
20
11
6
001
002:01
004:01
007:01
008:01/04
009:01
009:02
010:01
011:01
012:01
012:03
016
017
018:01
027:01
049:01
True

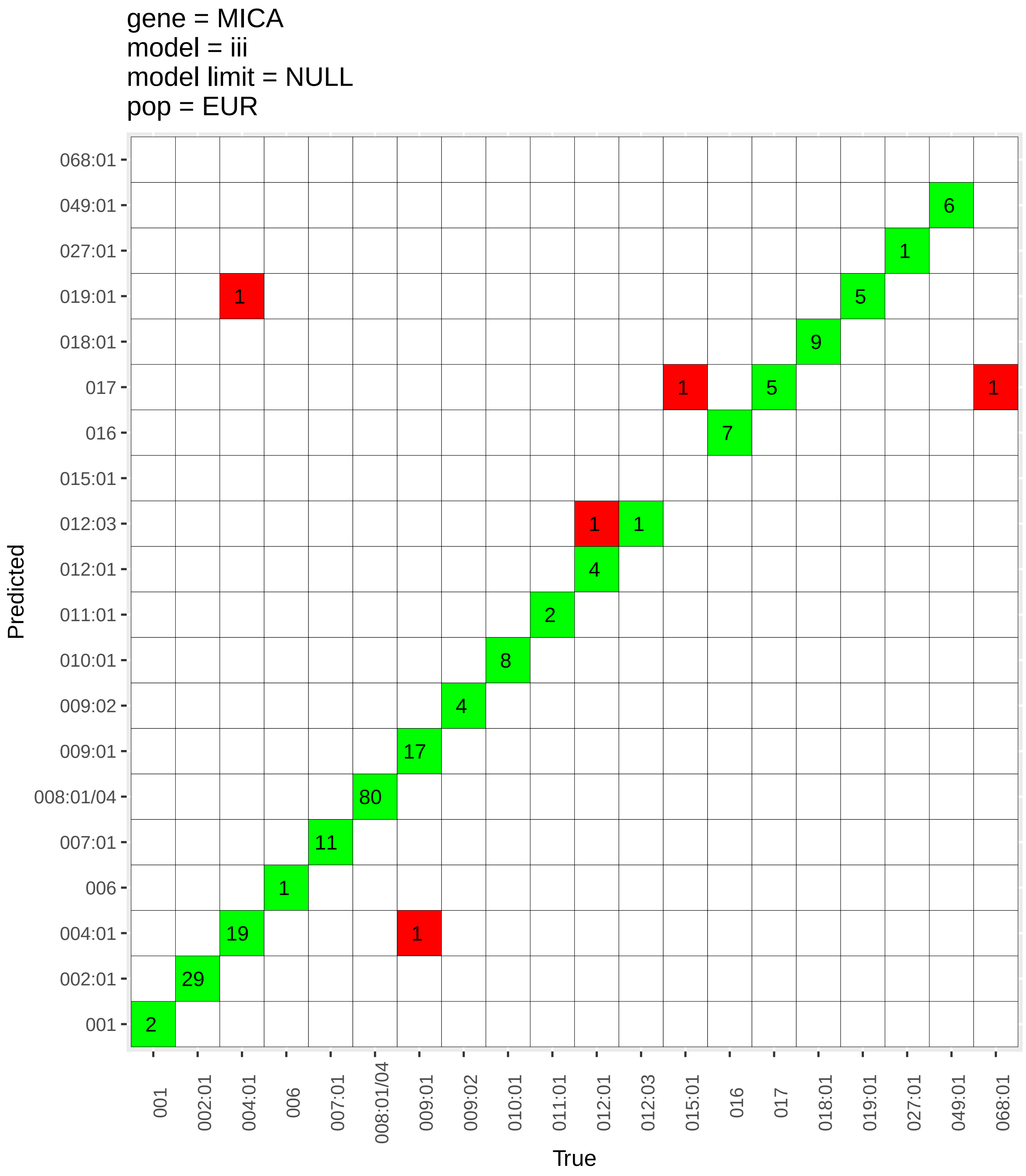
gene = MICA
model = iii
model limit = NULL
pop = EUR
Predicted
068:01
049:01
027:01
019:01
018:01
017
016
015:01
012:03
012:01
011:01
010:01
009:02
009:01
008:01/04
007:01
006
004:01
002:01
001
6
1
1
5
9
1
5
1
7
1
1
4
2
8
4
17
80
11
1
19
1
29
2
001
002:01
004:01
006
007:01
008:01/04
009:01
009:02
010:01
011:01
012:01
012:03
015:01
016
017
018:01
019:01
027:01
049:01
068:01
True

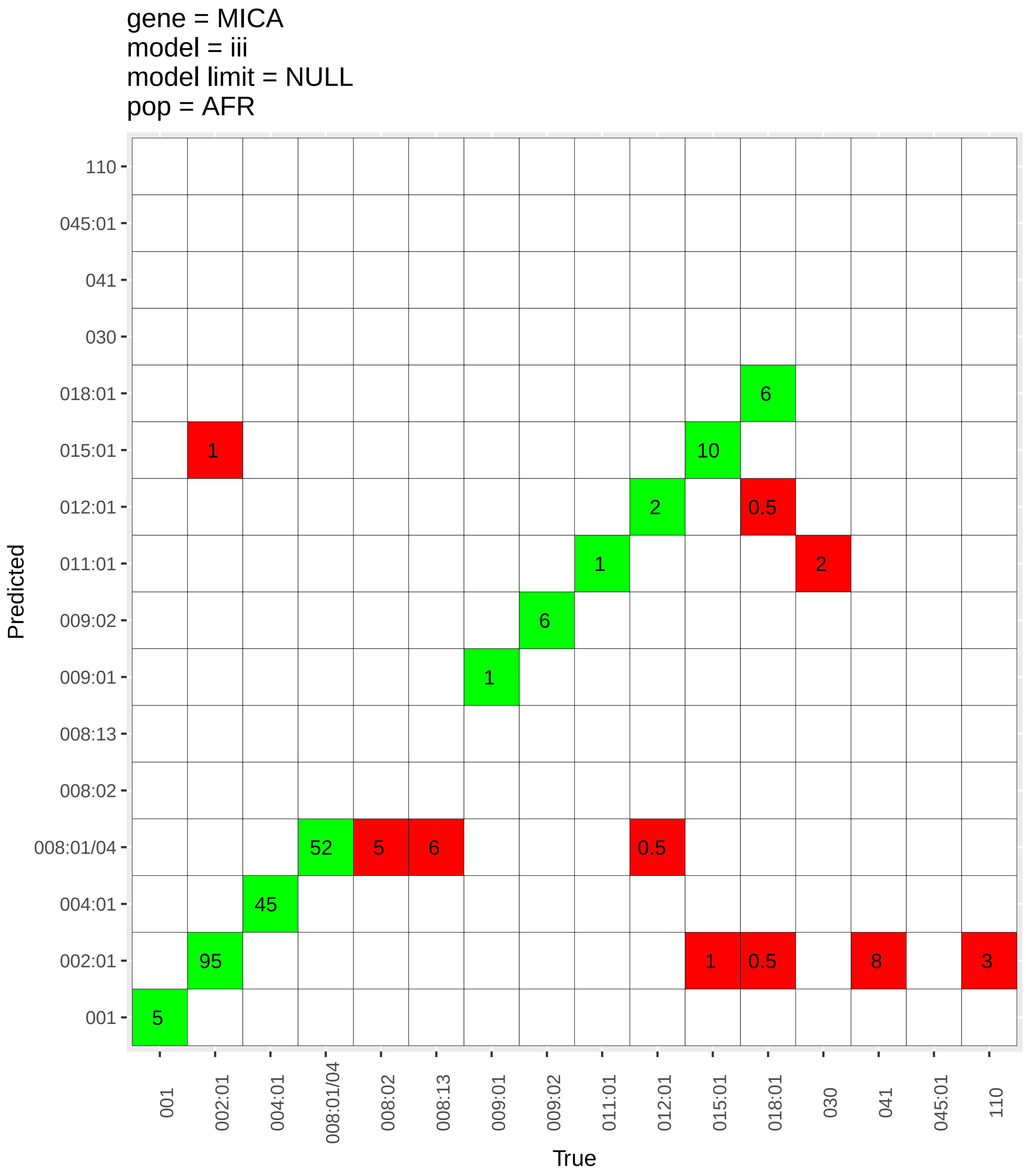
gene = MICA
model = iii
model limit = NULL
pop = AFR
Predicted
110
045:01
041
030
018:01
015:01
012:01
011:01
009:02
009:01
008:13
008:02
008:01/04
004:01
002:01
001
6
1
10
2
0.5
1
2
6
1
52
5
6
0.5
45
95
1
0.5
8
3
5
001
002:01
004:01
008:01/04
008:02
008:13
009:01
009:02
011:01
012:01
015:01
018:01
030
041
045:01
110
True

gene = MICA
model = iii
model limit = NULL
pop = EAS

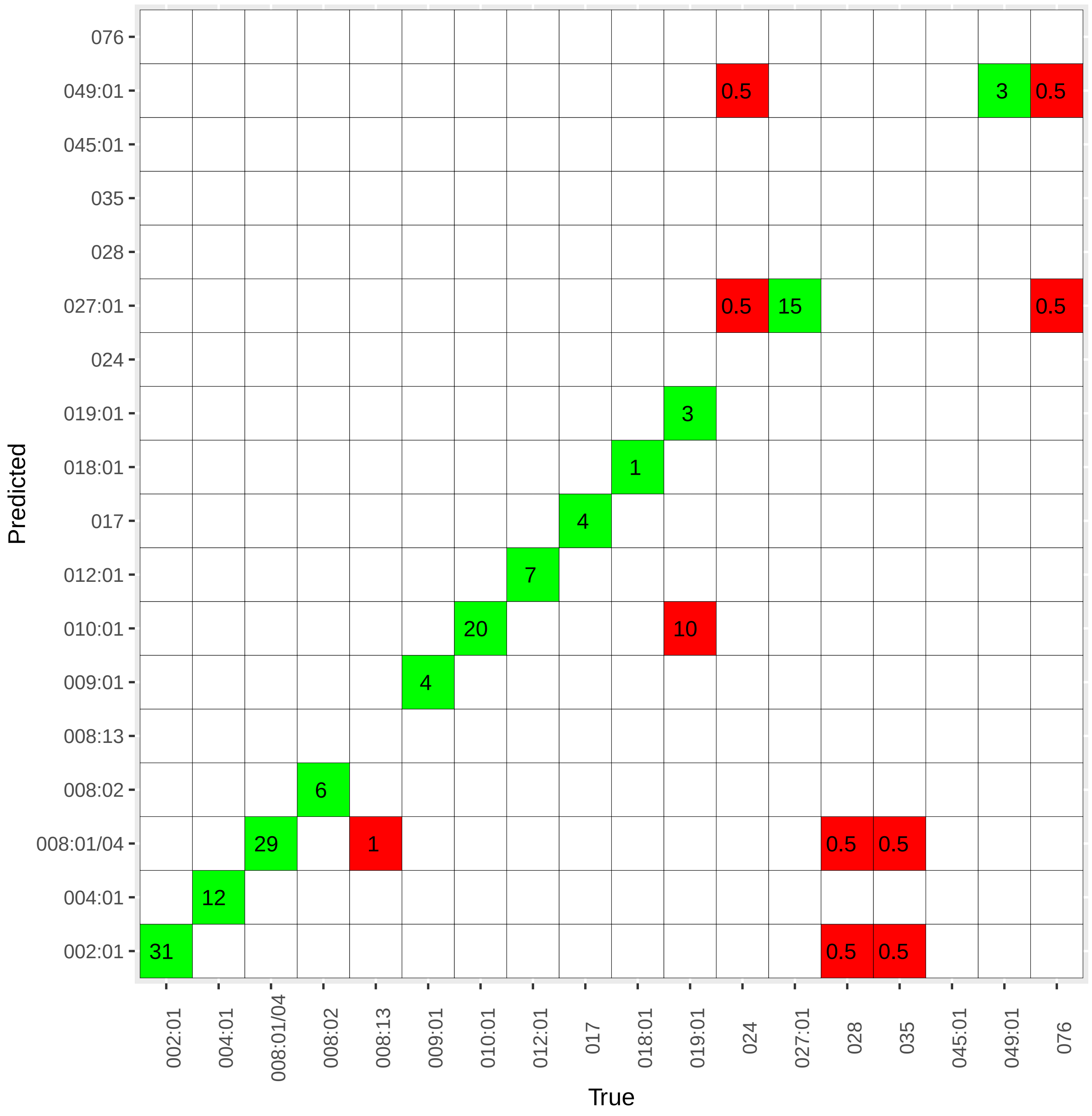

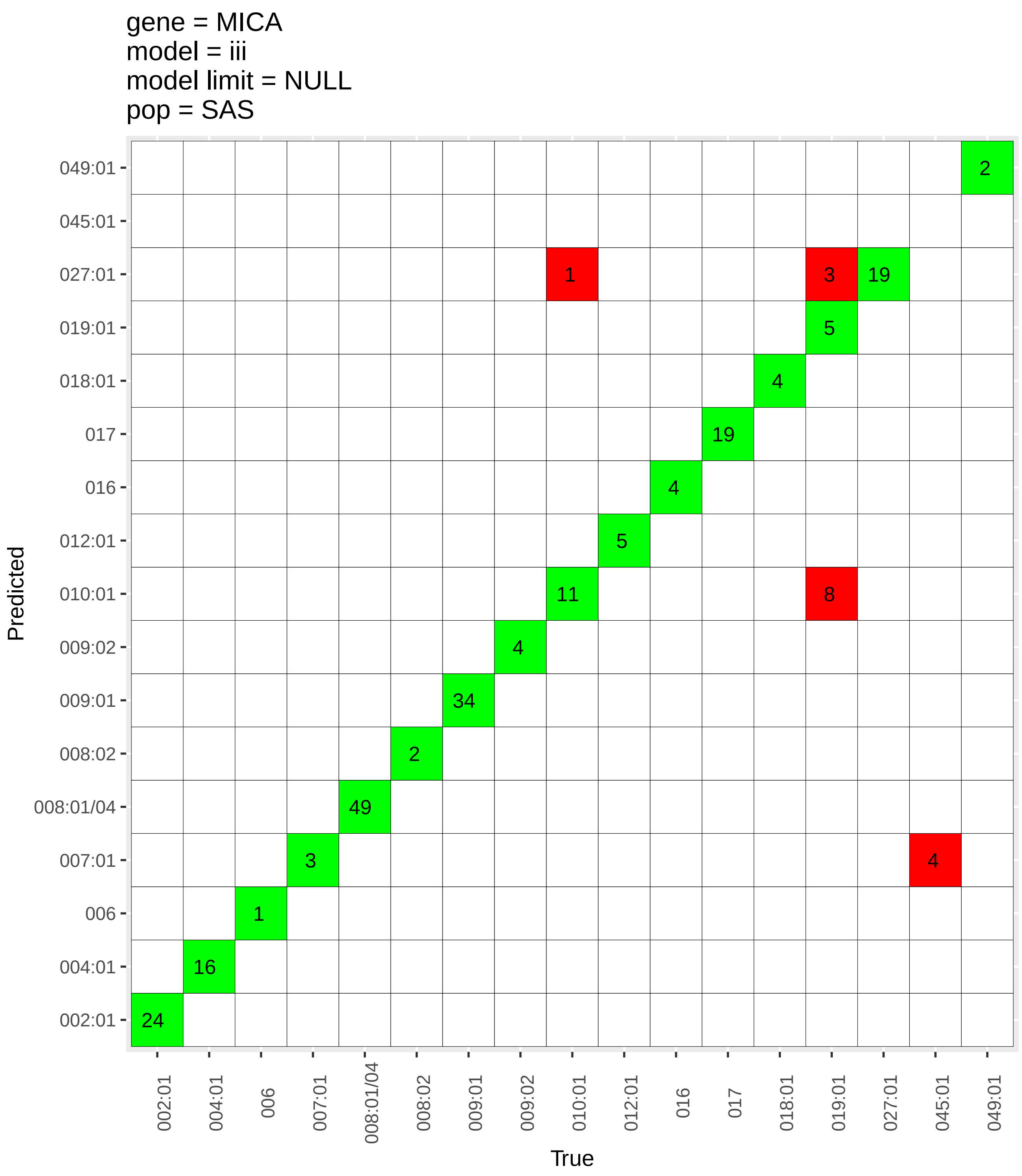
gene = MICA
model = iii
model limit = NULL
pop = SAS
Predicted
True
049:01
045:01
027:01
019:01
018:01
017
016
012:01
010:01
009:02
009:01
008:02
008:01/04
007:01
006
004:01
002:01
2
1
3
19
5
4
19
4
5
11
8
4
34
2
49
3
4
1
16
24

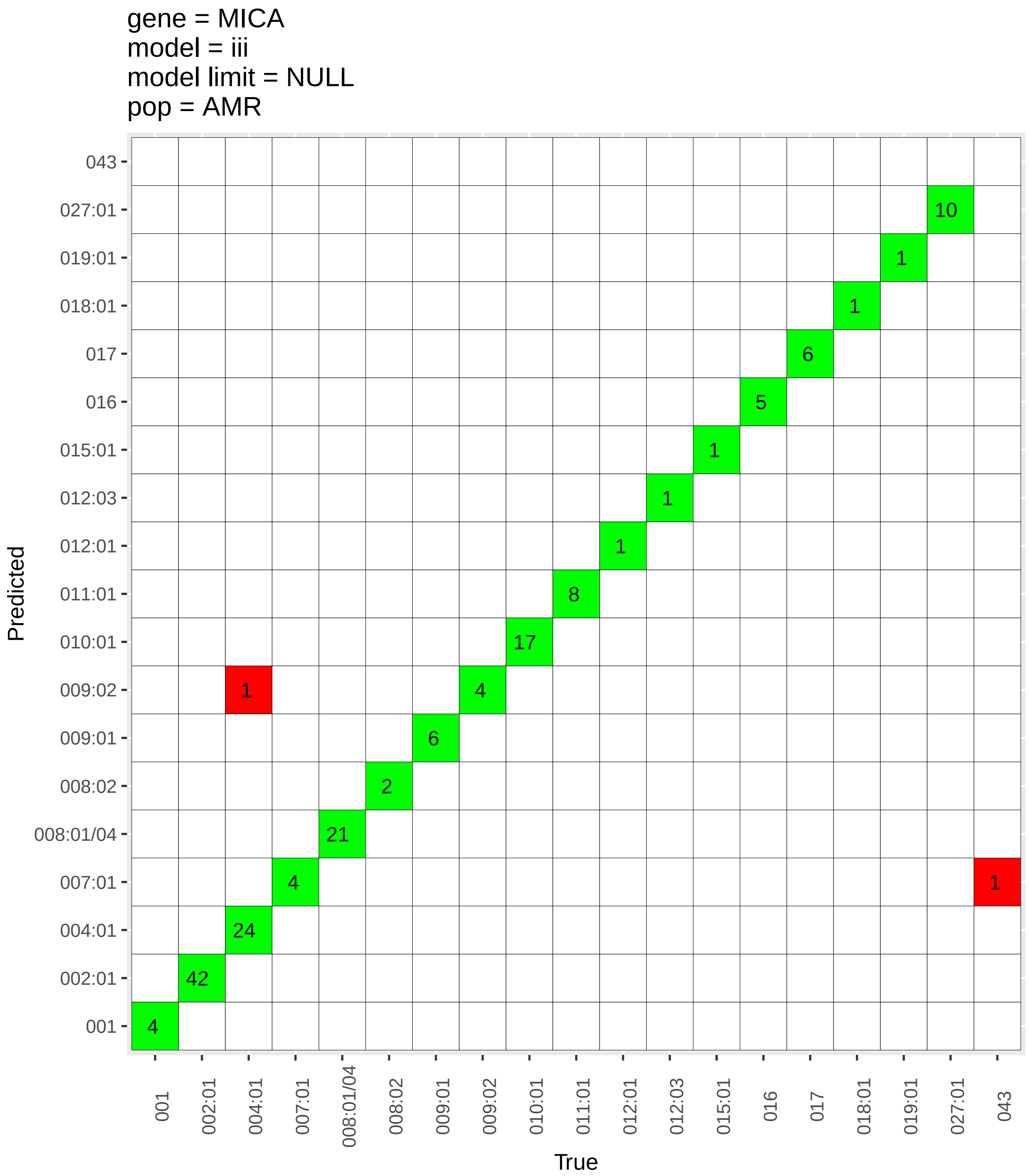
gene = MICA
model = iii
model limit = NULL
pop = AMR
Predicted
True
043
027:01
019:01
018:01
017
016
015:01
012:03
012:01
011:01
010:01
009:02
009:01
008:02
008:01/04
007:01
004:01
002:01
001
10
1
1
6
5
1
1
1
8
17
1
4
6
2
21
4
1
24
42
4
001
002:01
004:01
007:01
008:01/04
008:02
009:01
009:02
010:01
011:01
012:01
012:03
015:01
016
017
018:01
019:01
027:01
043

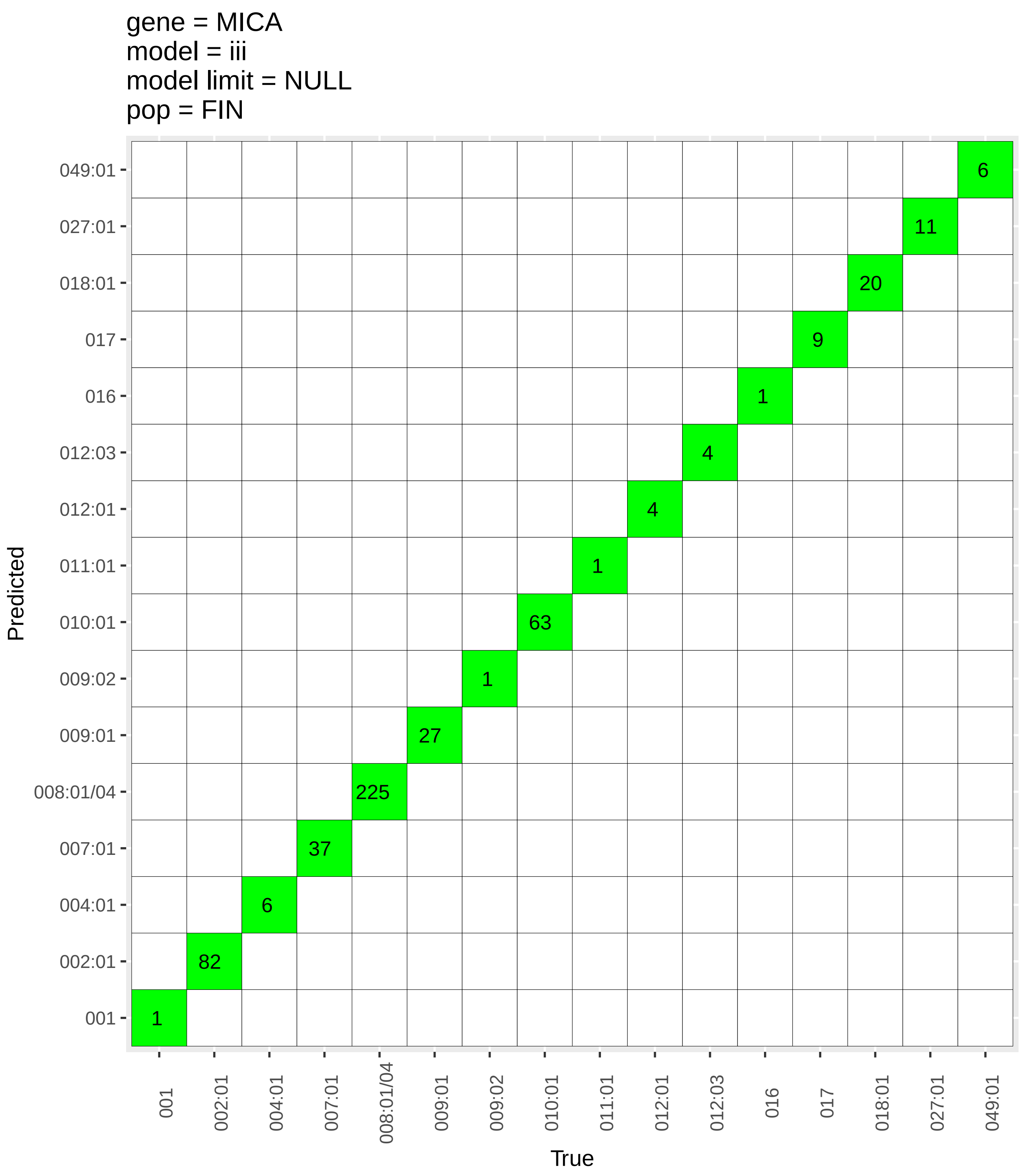
gene = MICA
model = iii
model limit = NULL
pop = FIN
Predicted
049:01
027:01
018:01
017
016
012:03
012:01
011:01
010:01
009:02
009:01
008:01/04
007:01
004:01
002:01
001
6
11
20
9
1
4
4
1
63
1
27
225
37
6
82
1
001
002:01
004:01
007:01
008:01/04
009:01
009:02
010:01
011:01
012:01
012:03
016
017
018:01
027:01
049:01
True

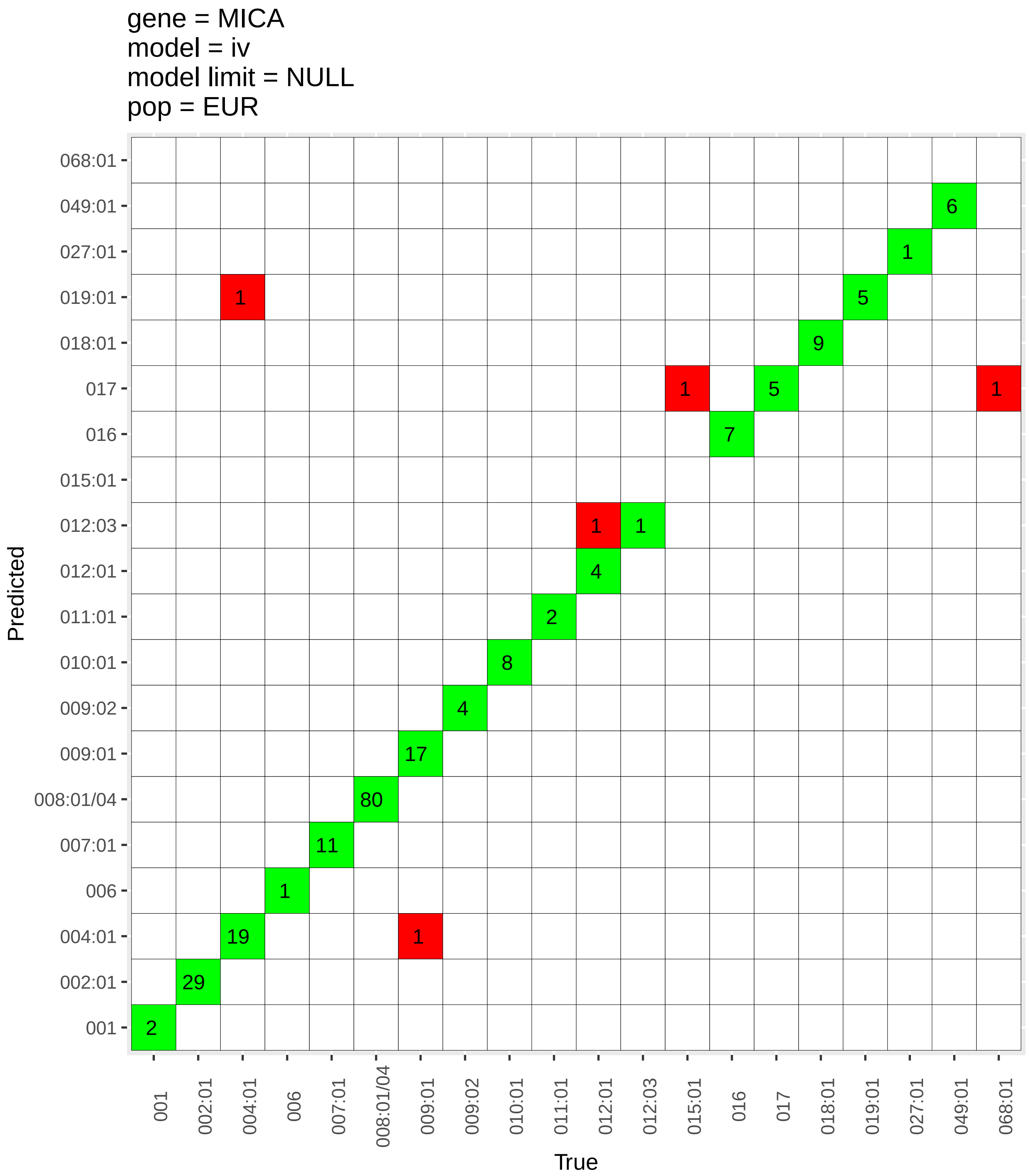
gene = MICA
model = iv
model limit = NULL
pop = EUR
Predicted
068:01
049:01
027:01
019:01
018:01
017
016
015:01
012:03
012:01
011:01
010:01
009:02
009:01
008:01/04
007:01
006
004:01
002:01
001
6
1
1
5
9
1
5
1
7
1
1
4
2
8
4
17
80
11
1
19
1
29
2
001
002:01
004:01
006
007:01
008:01/04
009:01
009:02
010:01
011:01
012:01
012:03
015:01
016
017
018:01
019:01
027:01
049:01
068:01
True

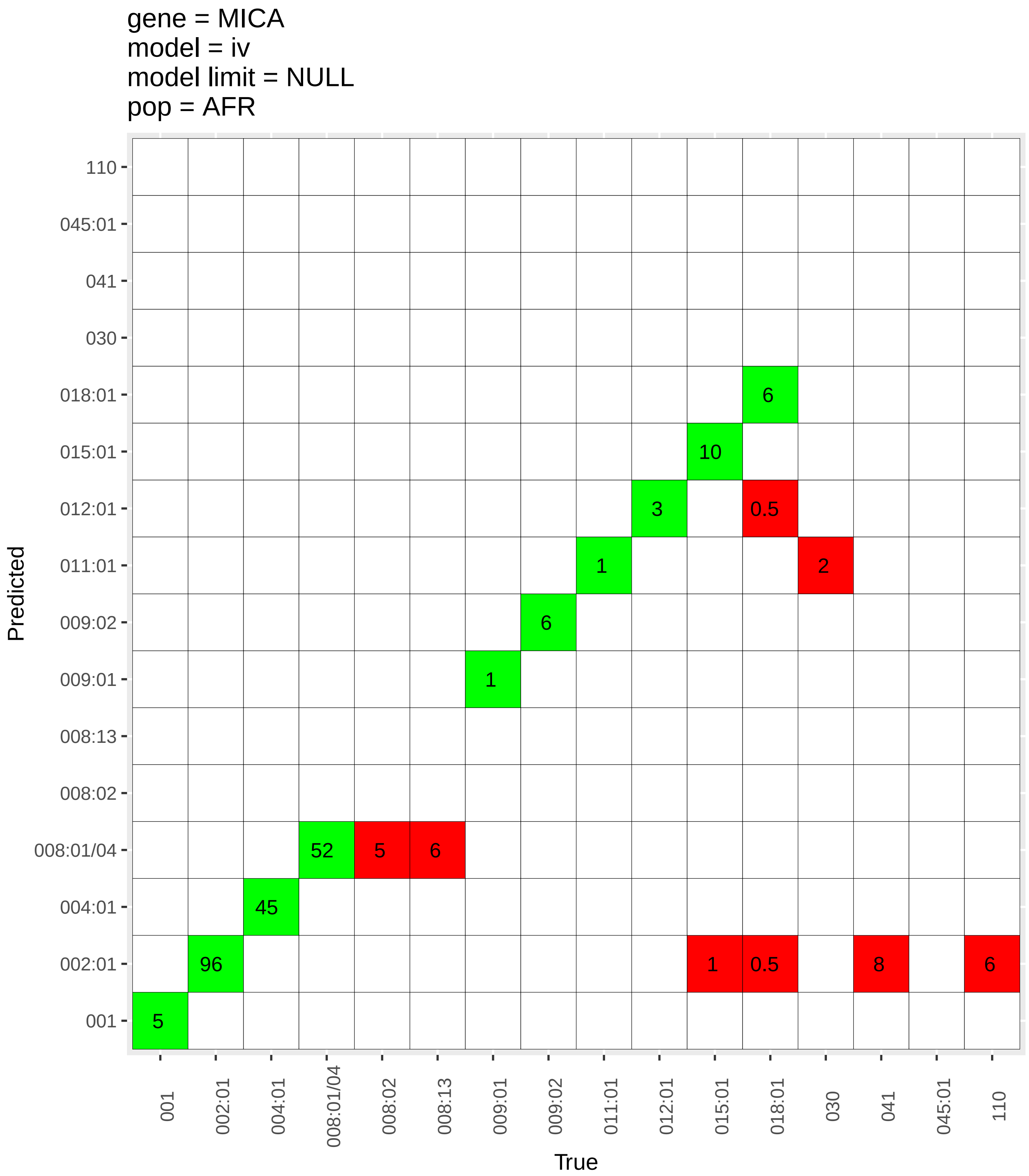
gene = MICA
model = iv
model limit = NULL
pop = AFR
Predicted
True
110
045:01
041
030
018:01
015:01
012:01
011:01
009:02
009:01
008:13
008:02
008:01/04
004:01
002:01
001
5
96
45
52
5
6
1
6
1
3
0.5
2
10
6
1
0.5
8
6

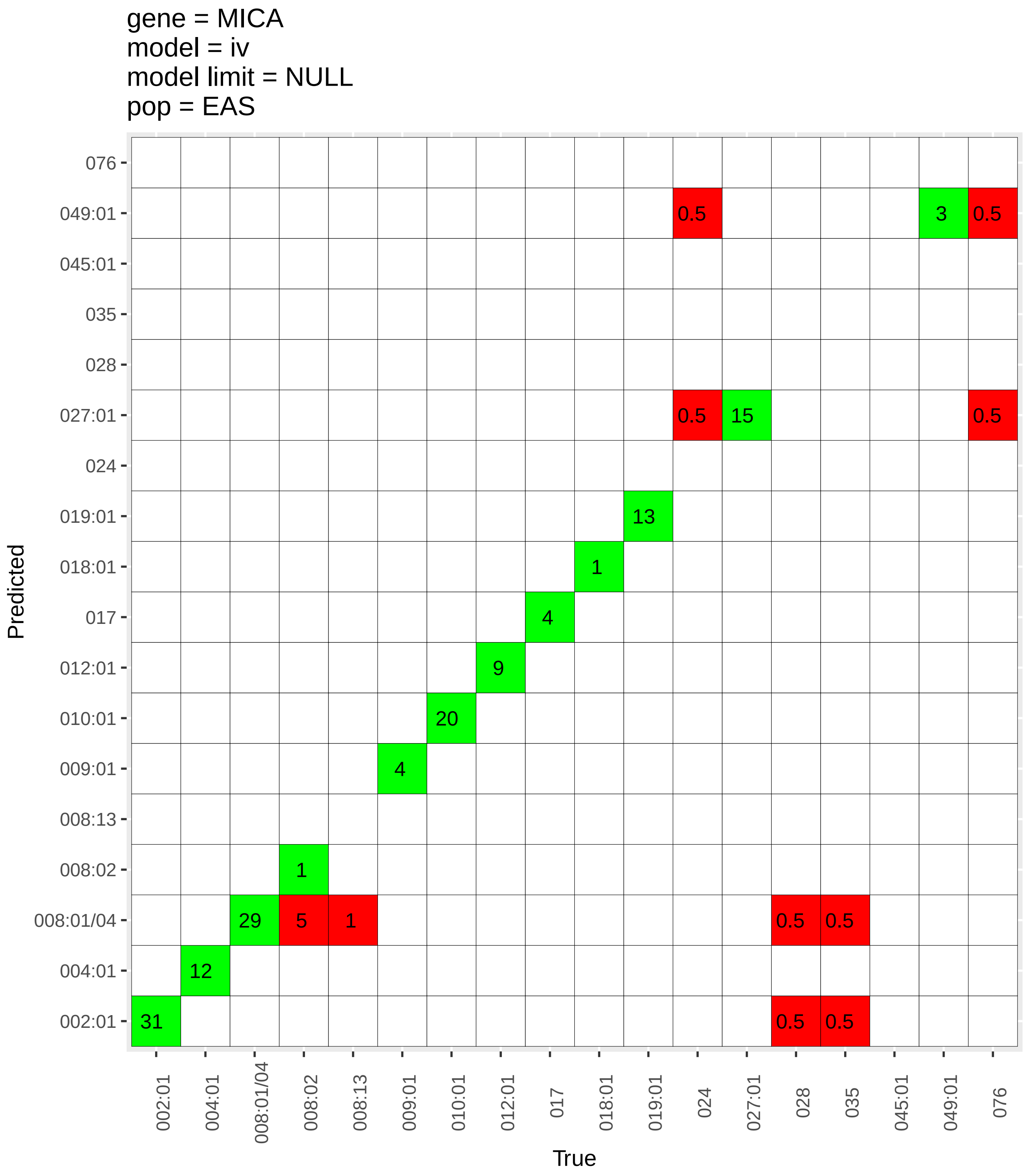
gene = MICA
model = iv
model limit = NULL
pop = EAS
Predicted
True
076
049:01
045:01
035
028
027:01
024
019:01
018:01
017
012:01
010:01
009:01
008:13
008:02
008:01/04
004:01
002:01
0.5
3
0.5
0.5
15
0.5
13
1
4
9
20
4
1
29
5
1
0.5
0.5
12
31
0.5
0.5
002:01
004:01
008:01/04
008:02
008:13
009:01
010:01
012:01
017
018:01
019:01
024
027:01
028
035
045:01
049:01
076

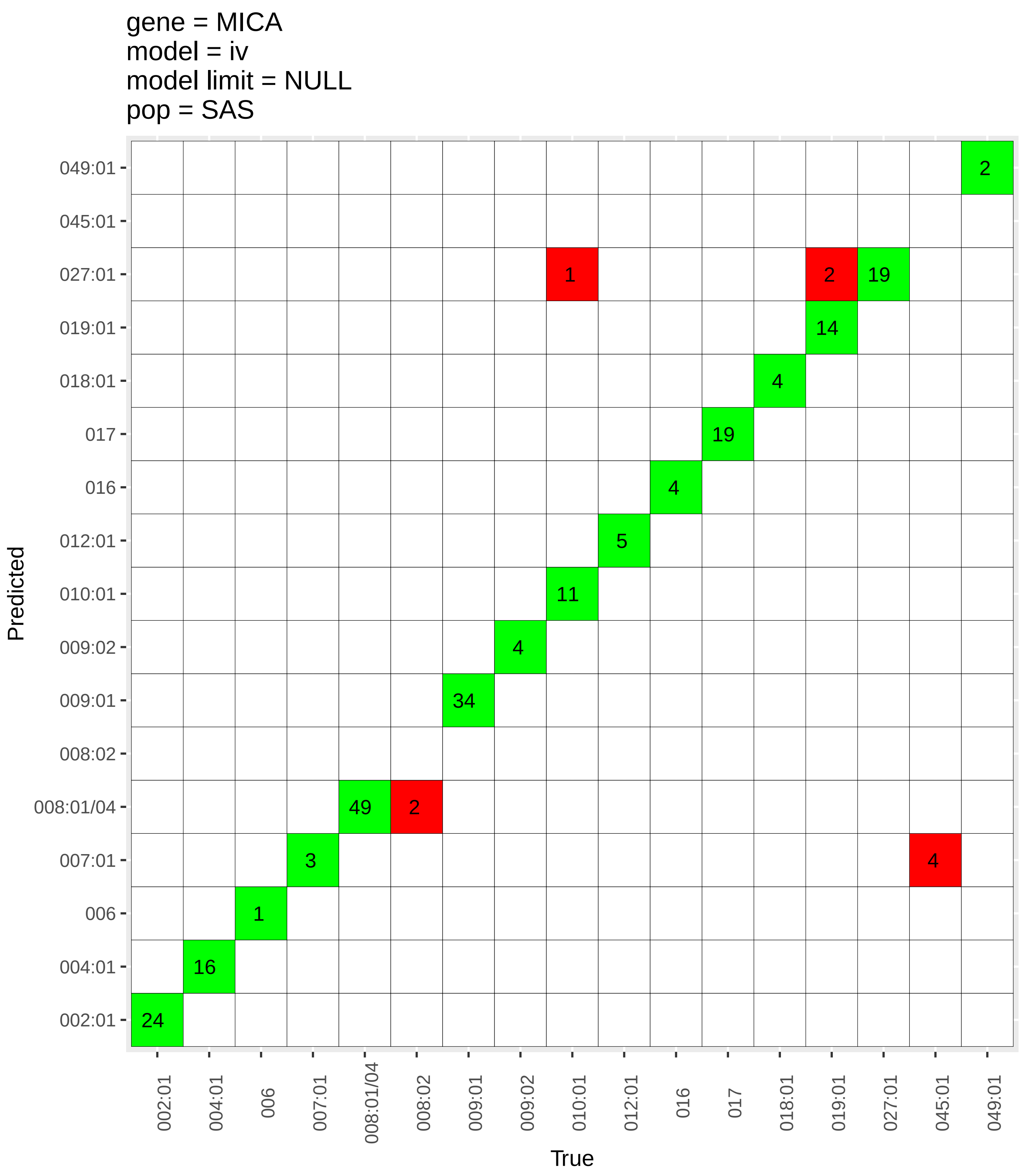
gene = MICA
model = iv
model limit = NULL
pop = SAS
Predicted
049:01
045:01
027:01
019:01
018:01
017
016
012:01
010:01
009:02
009:01
008:02
008:01/04
007:01
006
004:01
002:01
2
1
2
19
14
4
19
4
5
11
4
34
49
2
3
4
1
16
24
002:01
004:01
006
007:01
008:01/04
008:02
009:01
009:02
010:01
012:01
016
017
018:01
019:01
027:01
045:01
049:01
True

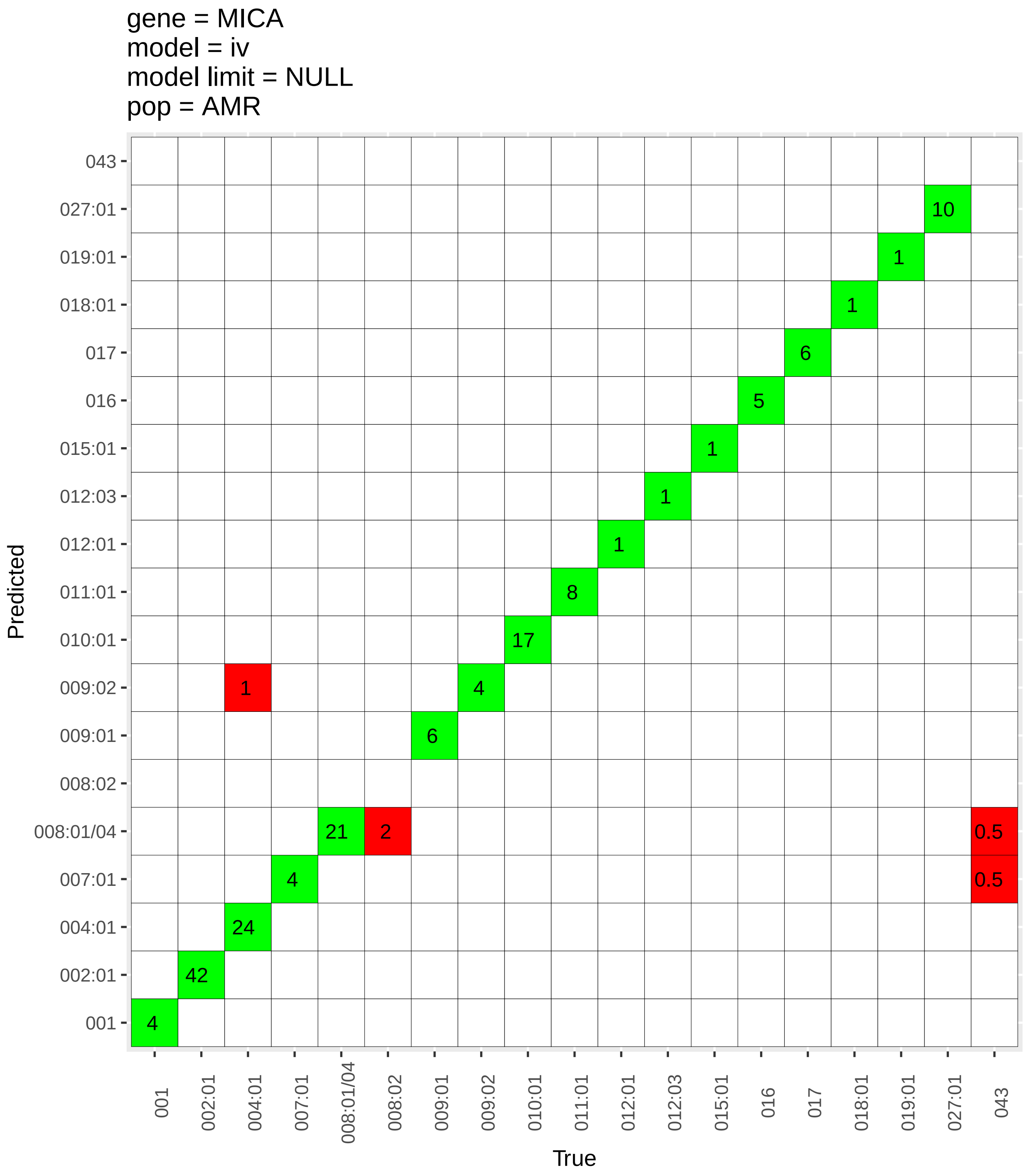
gene = MICA
model = iv
model limit = NULL
pop = AMR
Predicted
043
027:01
019:01
018:01
017
016
015:01
012:03
012:01
011:01
010:01
009:02
009:01
008:02
008:01/04
007:01
004:01
002:01
001
10
1
1
6
5
1
1
1
8
17
1
4
6
21
2
0.5
4
0.5
24
42
4
001
002:01
004:01
007:01
008:01/04
008:02
009:01
009:02
010:01
011:01
012:01
012:03
015:01
016
017
018:01
019:01
027:01
043
True

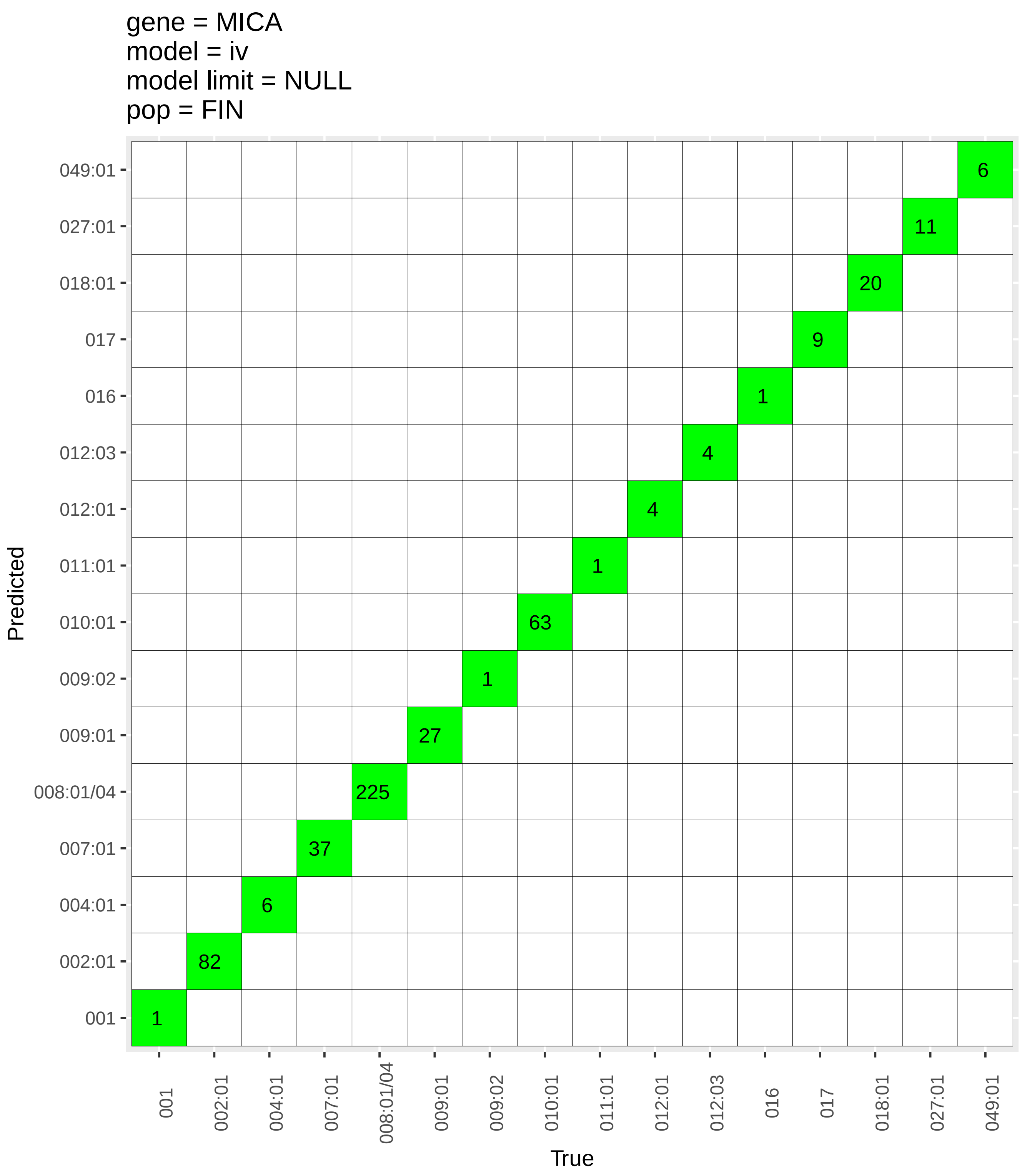
gene = MICA
model = iv
model limit = NULL
pop = FIN
Predicted
True
049:01
027:01
018:01
017
016
012:03
012:01
011:01
010:01
009:02
009:01
008:01/04
007:01
004:01
002:01
001
1
82
6
37
225
27
1
63
1
4
4
1
9
20
11
6

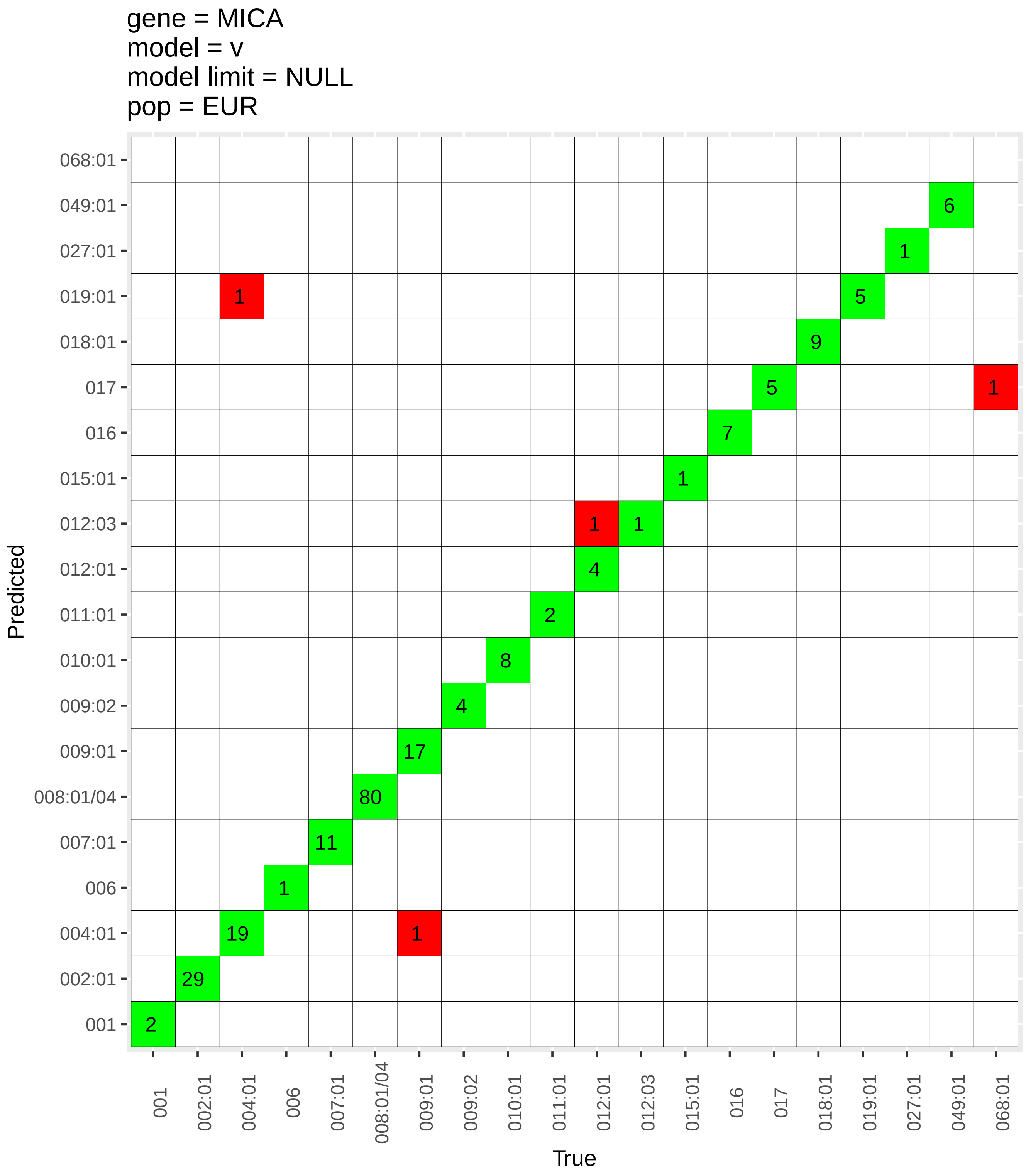
gene = MICA
model = v
model limit = NULL
pop = EUR
Predicted
True
068:01
049:01
027:01
019:01
018:01
017
016
015:01
012:03
012:01
011:01
010:01
009:02
009:01
008:01/04
007:01
006
004:01
002:01
001

gene = MICA
model = v
model limit = NULL
pop = AFR

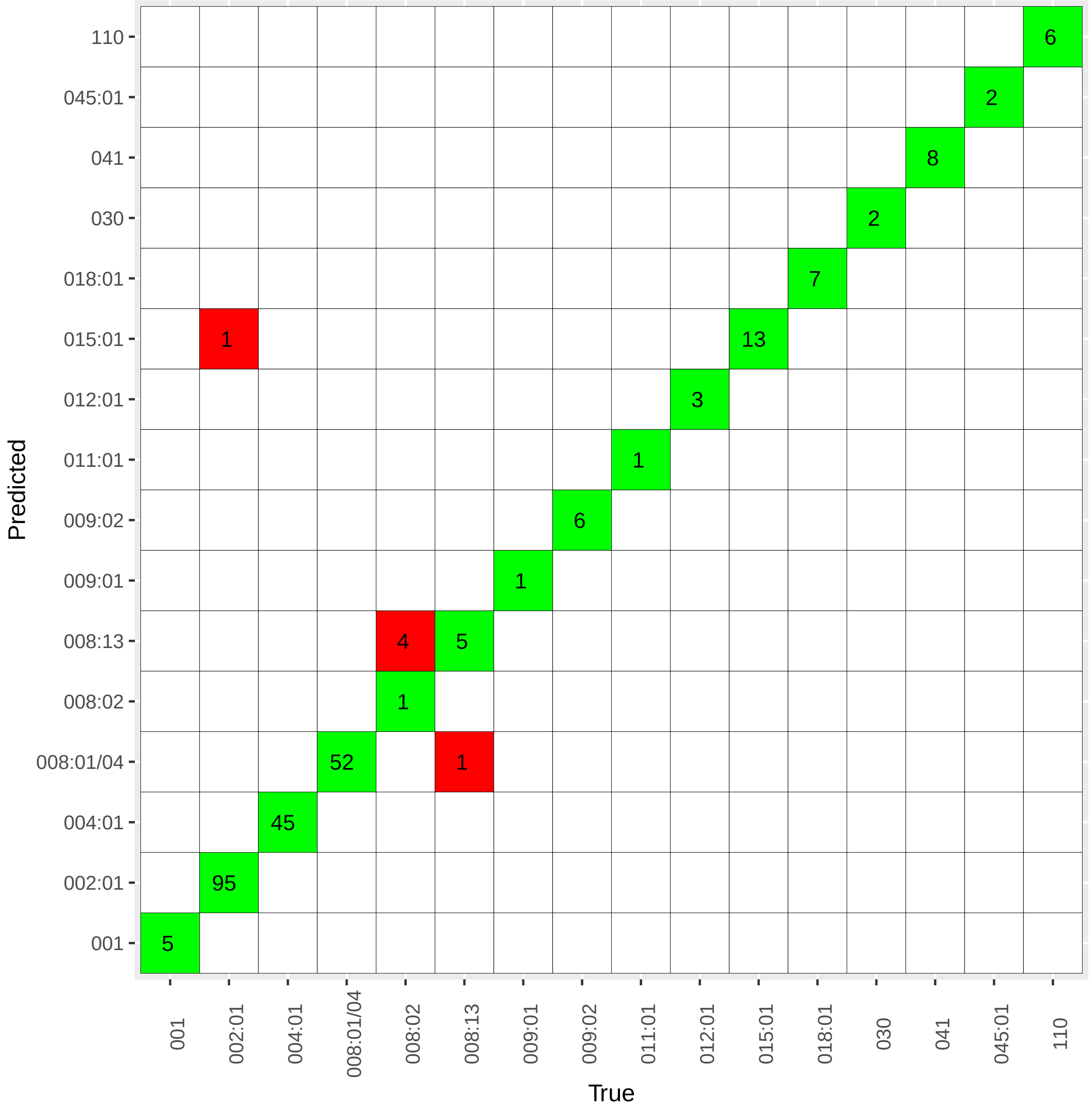

gene = MICA
model = v
model limit = NULL
pop = EAS

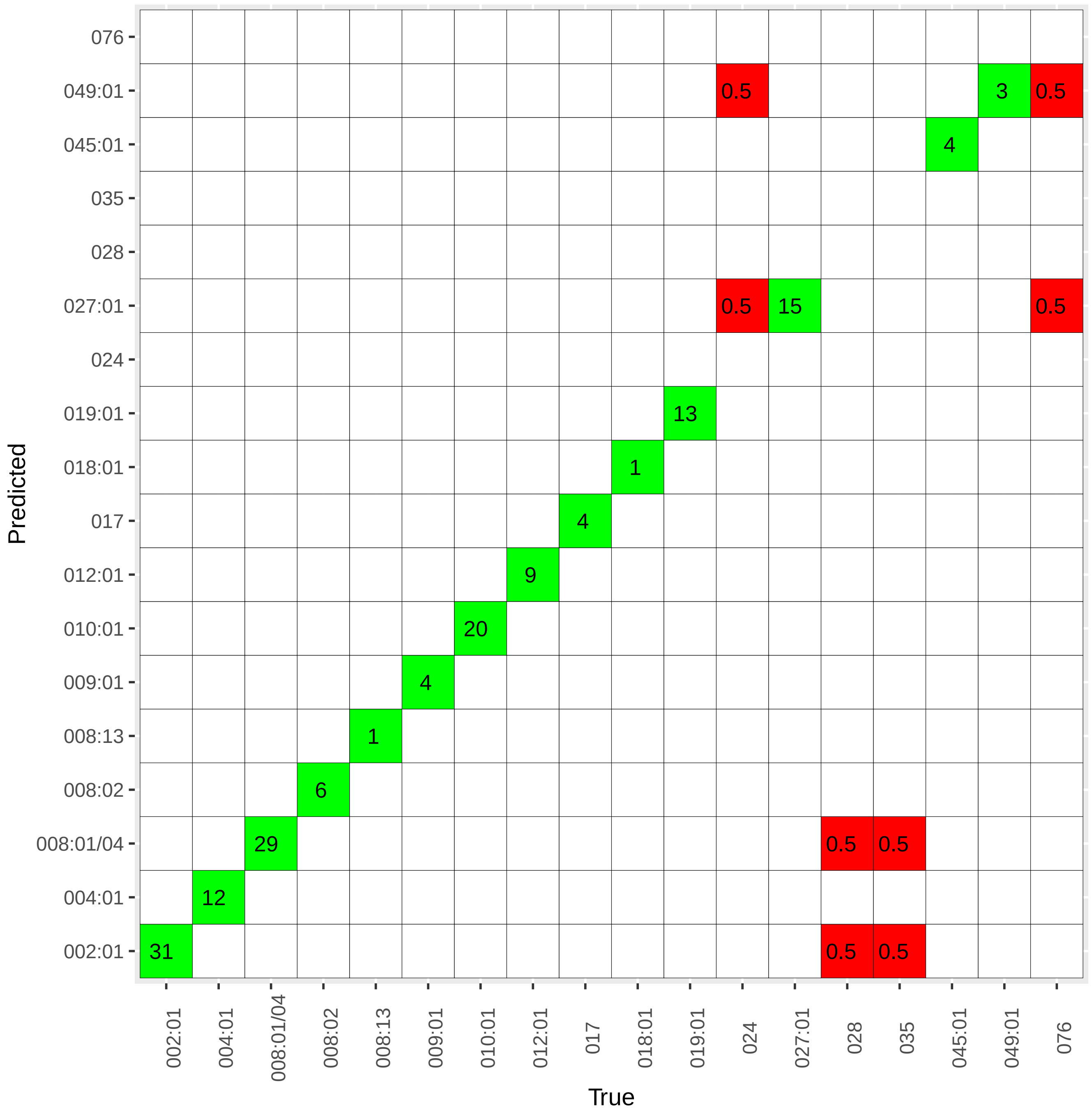

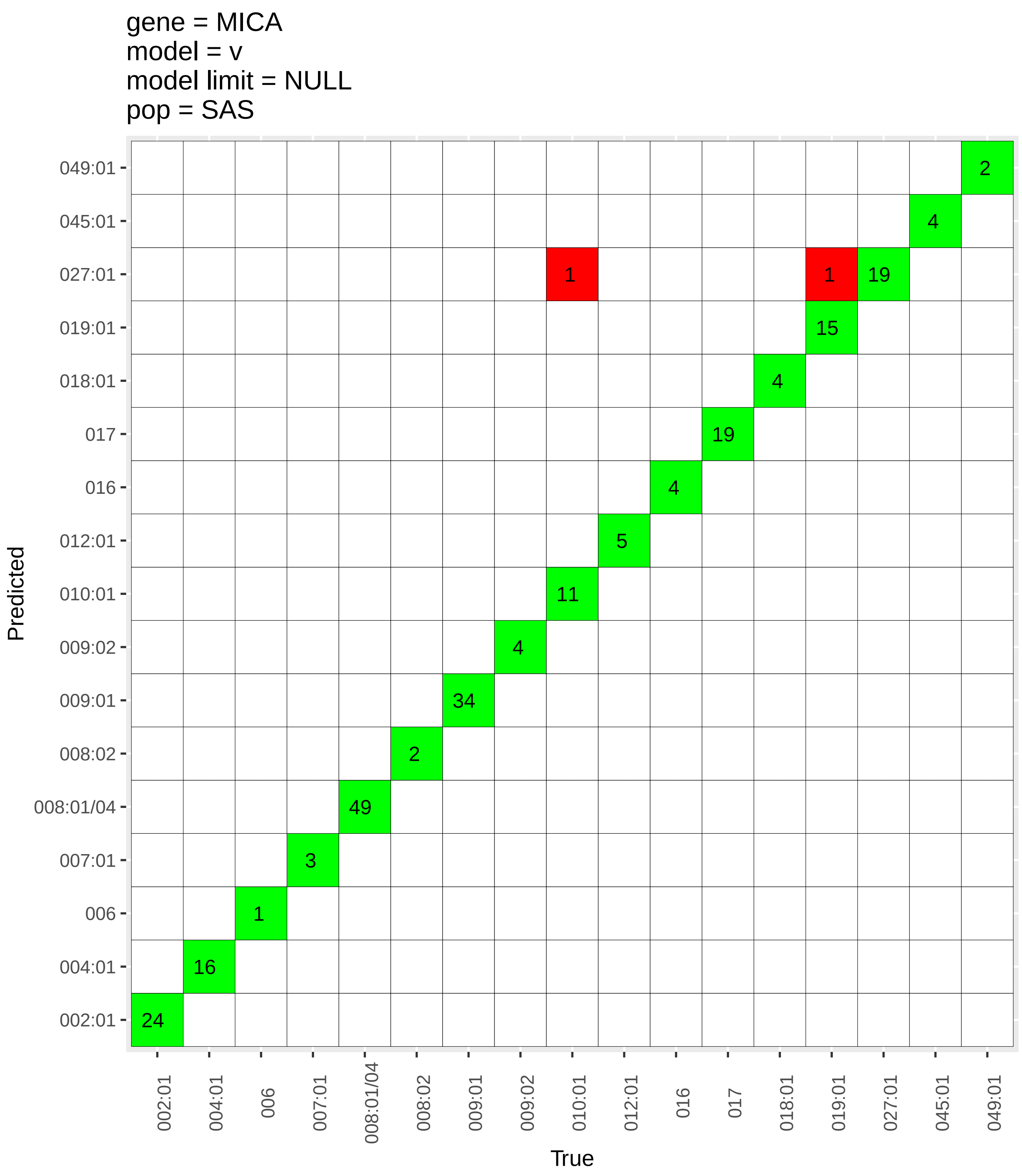
gene = MICA
model = v
model limit = NULL
pop = SAS
Predicted
True
049:01
045:01
027:01
019:01
018:01
017
016
012:01
010:01
009:02
009:01
008:02
008:01/04
007:01
006
004:01
002:01
24
16
1
3
49
2
34
4
11
5
4
19
4
15
1
1
19
4
2

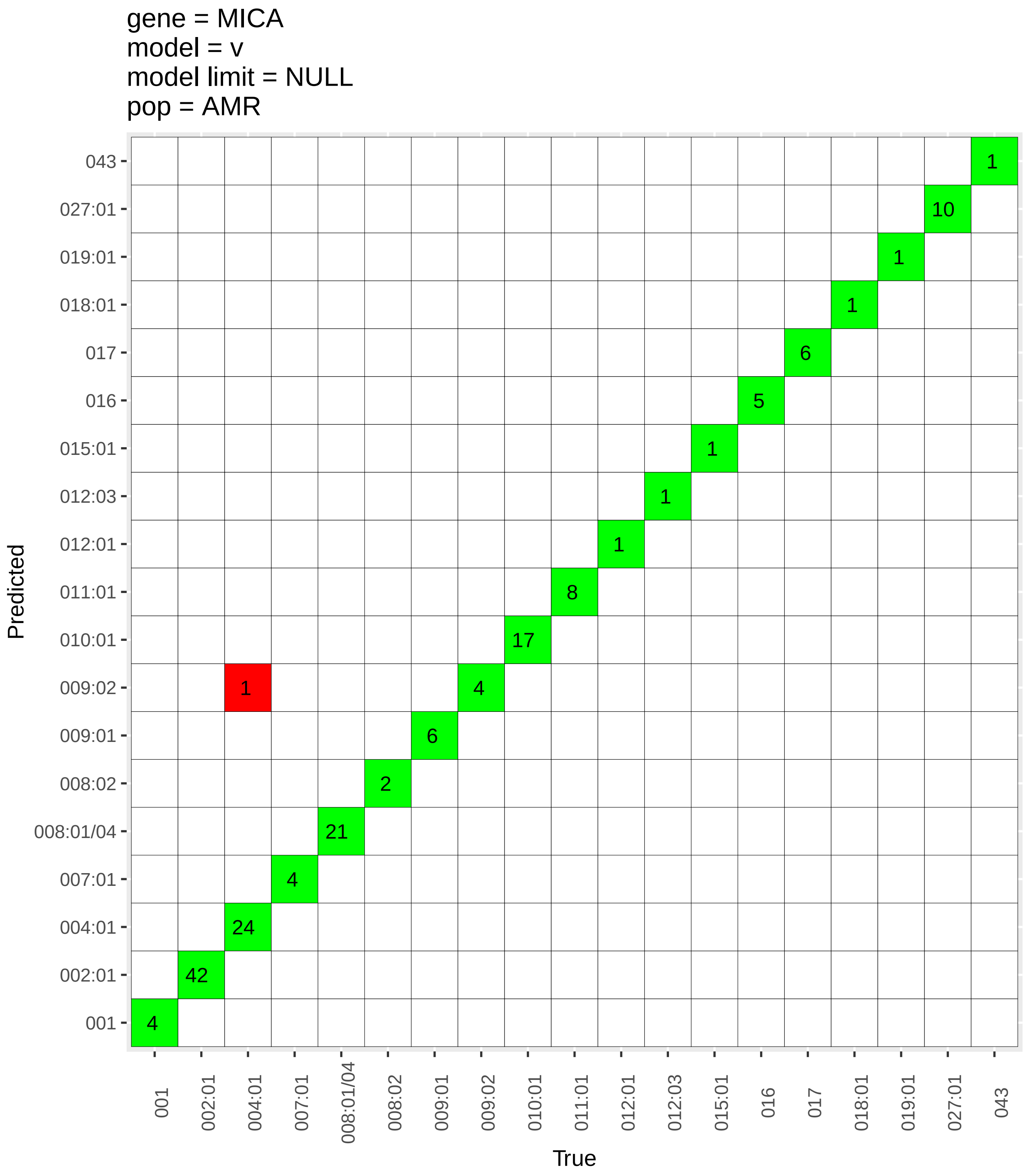
gene = MICA
model = v
model limit = NULL
pop = AMR
Predicted
043
027:01
019:01
018:01
017
016
015:01
012:03
012:01
011:01
010:01
009:02
009:01
008:02
008:01/04
007:01
004:01
002:01
001
True
001
002:01
004:01
007:01
008:01/04
008:02
009:01
009:02
010:01
011:01
012:01
012:03
015:01
016
017
018:01
019:01
027:01
043
4
42
24
4
21
2
6
1
4
17
8
1
1
1
5
6
1
1
10
1

gene = MICA
model = v
model limit = NULL
pop = FIN

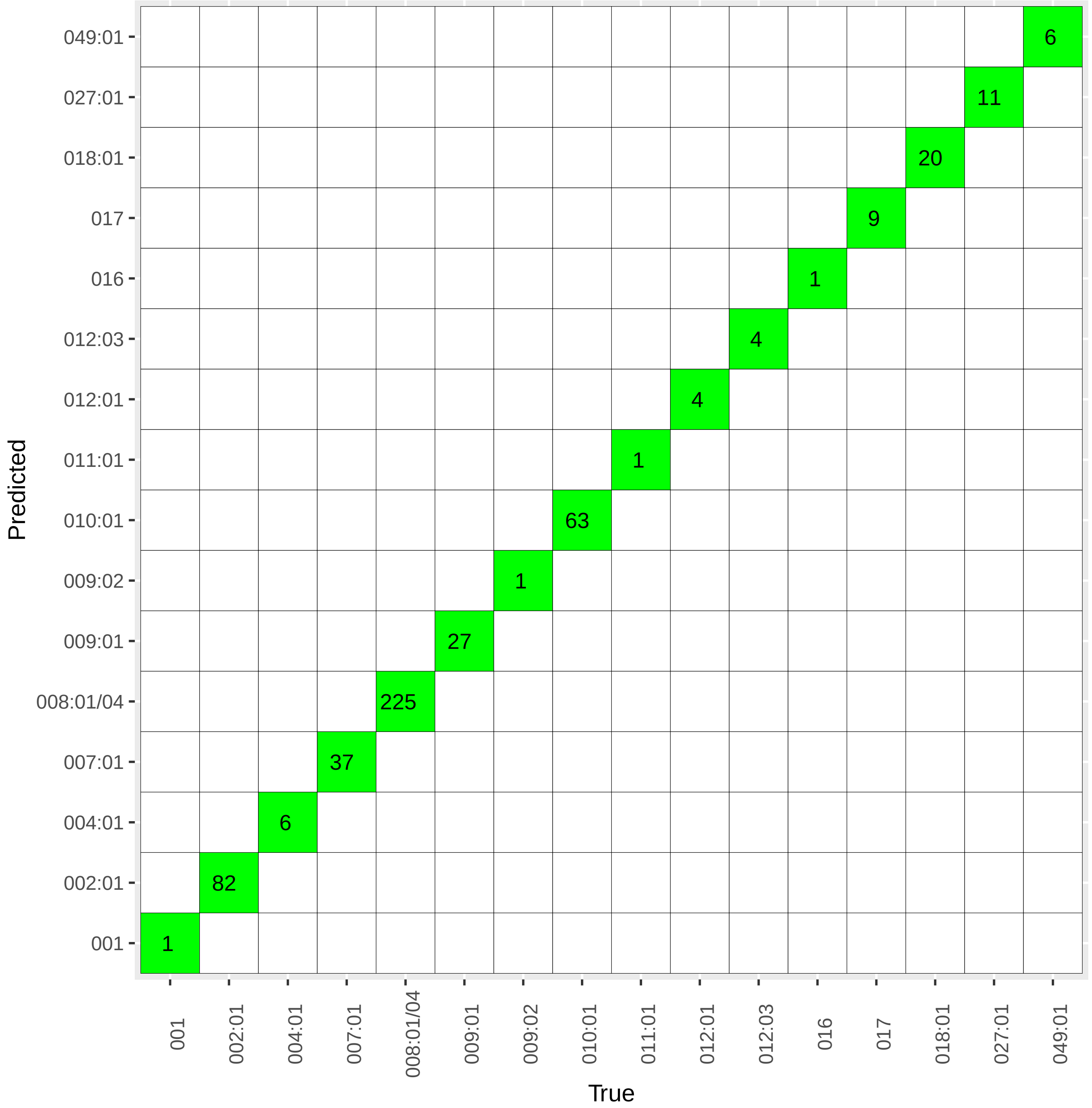

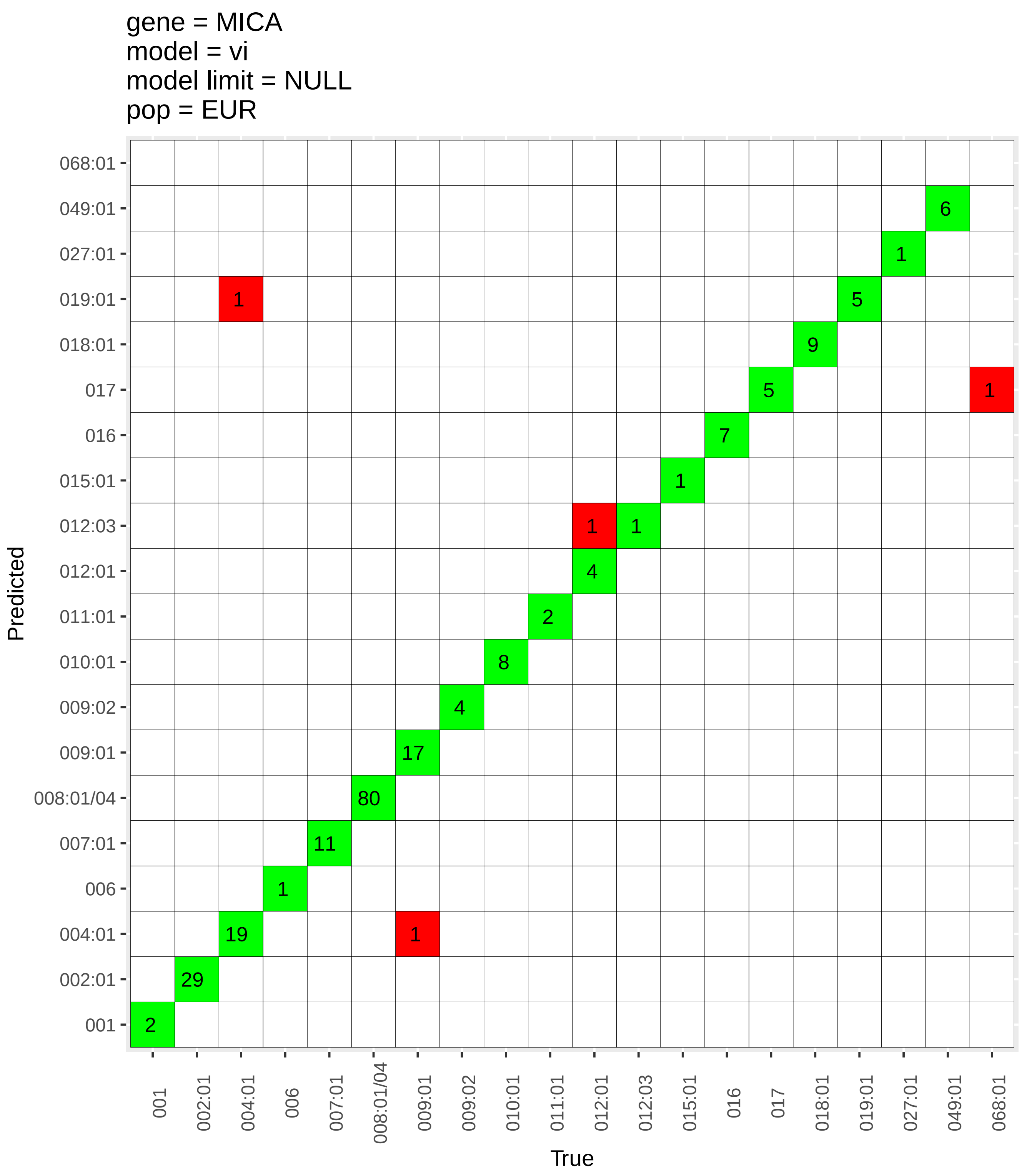
gene = MICA
model = vi
model limit = NULL
pop = EUR
Predicted
True
068:01
049:01
027:01
019:01
018:01
017
016
015:01
012:03
012:01
011:01
010:01
009:02
009:01
008:01/04
007:01
006
004:01
002:01
001
6
1
1
5
9
5
1
7
1
1
1
4
2
8
4
17
80
11
1
19
1
29
2

gene = MICA
model = vi
model limit = NULL
pop = AFR

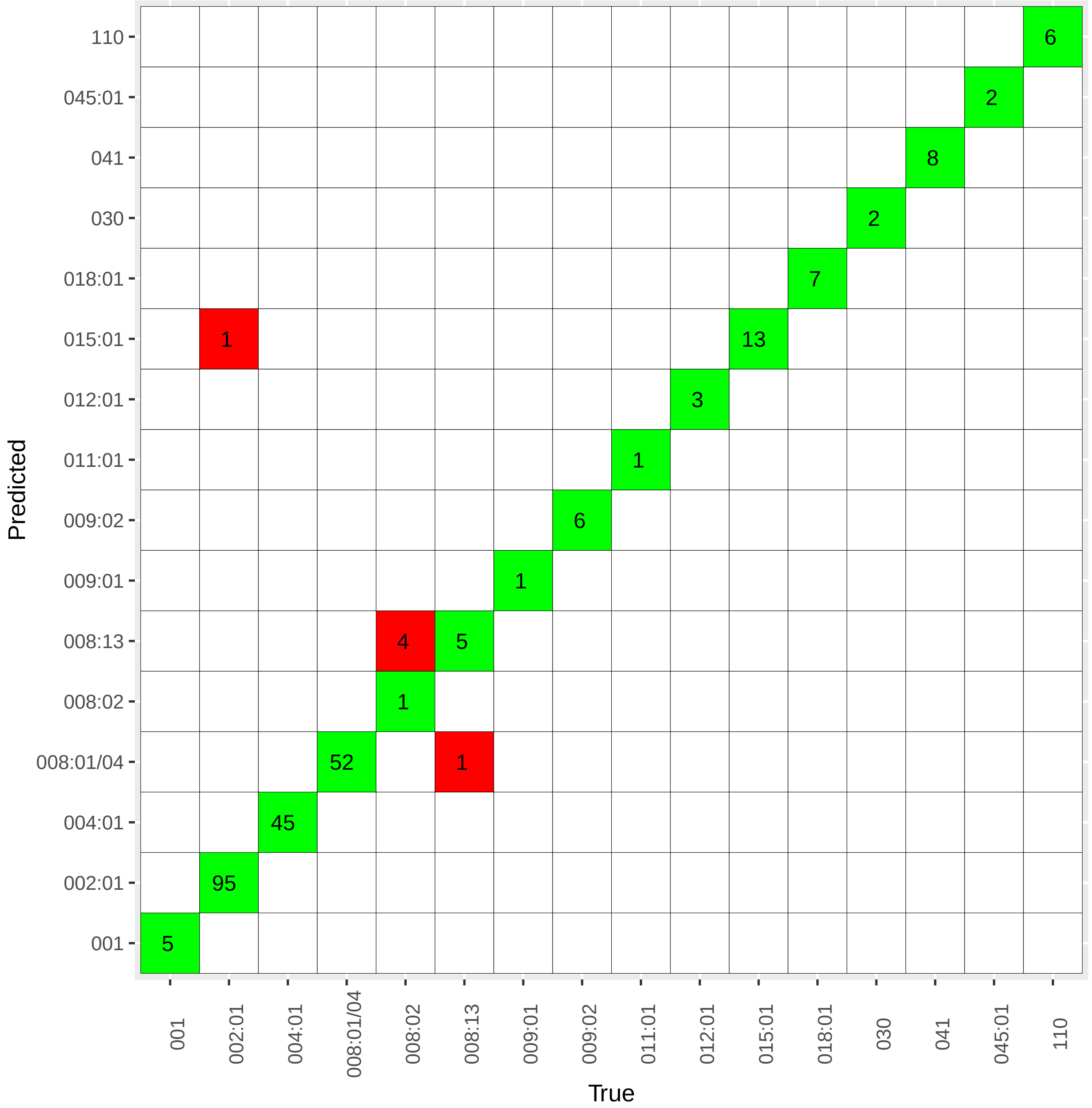

gene = MICA
model = vi
model limit = NULL
pop = EAS

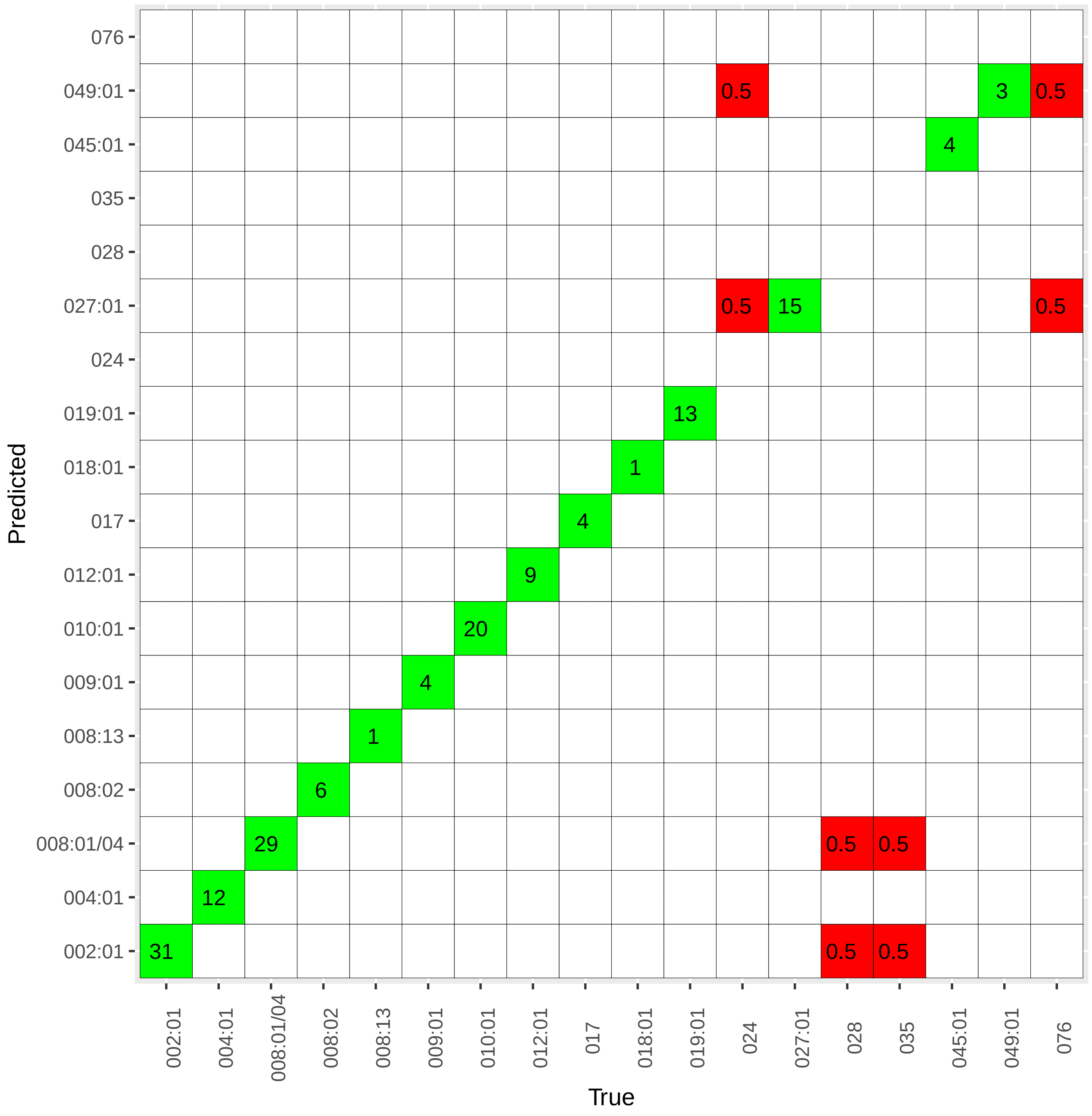

gene = MICA
model = vi
model limit = NULL
pop = SAS

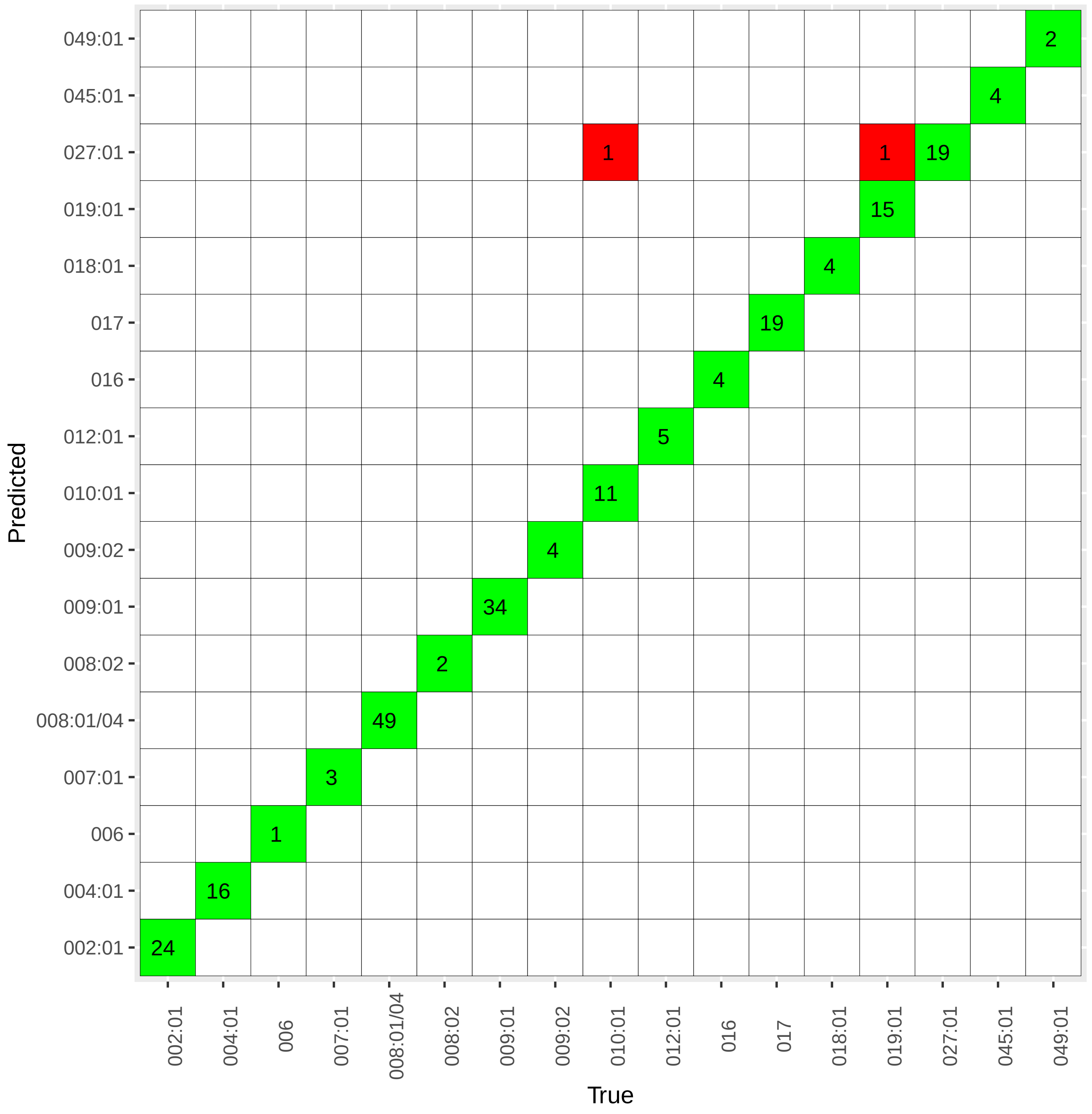

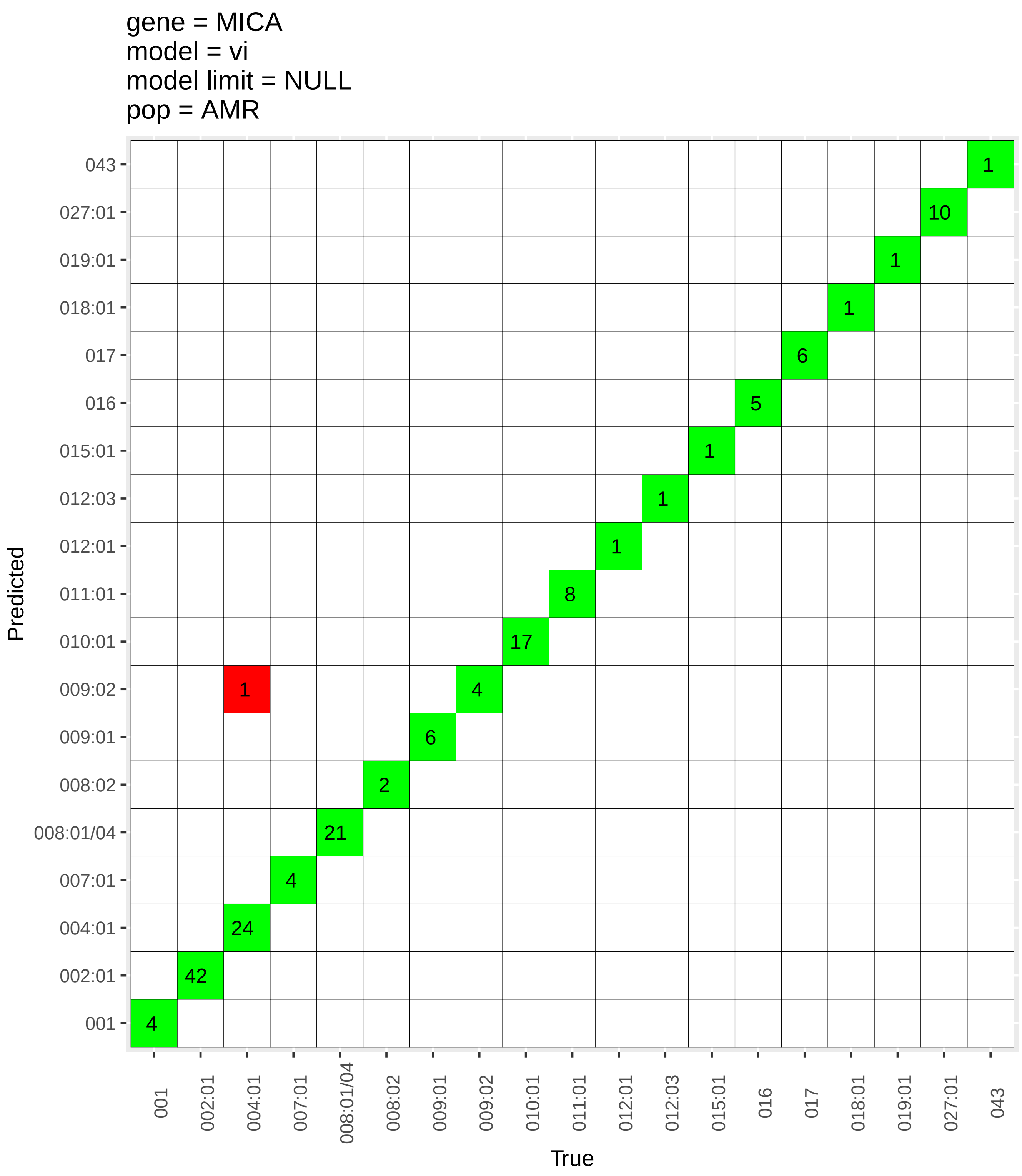
gene = MICA
model = vi
model limit = NULL
pop = AMR
Predicted
043
027:01
019:01
018:01
017
016
015:01
012:03
012:01
011:01
010:01
009:02
009:01
008:02
008:01/04
007:01
004:01
002:01
001
True
001
002:01
004:01
007:01
008:01/04
008:02
009:01
009:02
010:01
011:01
012:01
012:03
015:01
016
017
018:01
019:01
027:01
043
4
42
24
4
21
2
6
1
4
17
8
1
1
1
5
6
1
1
10
1

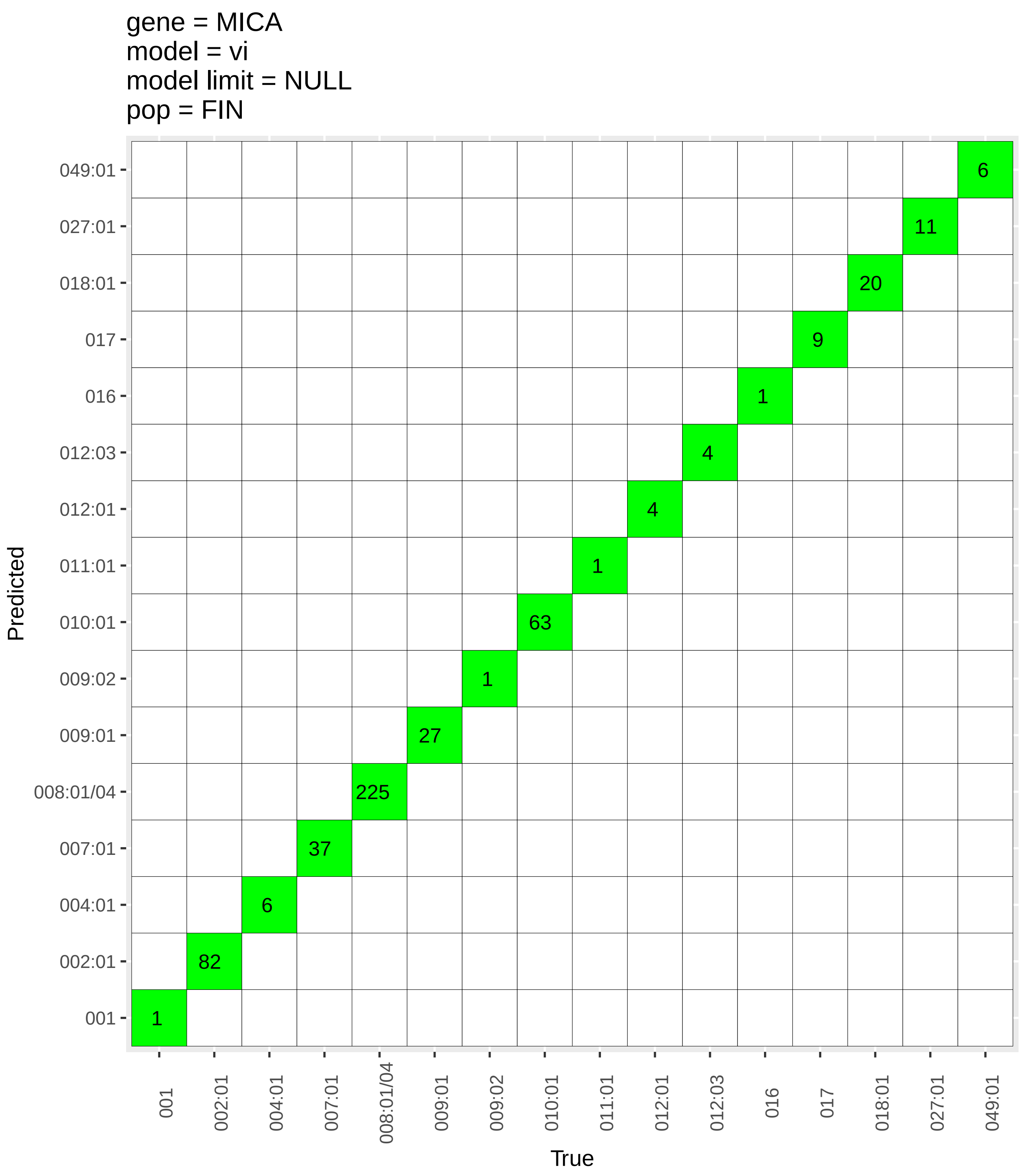
gene = MICA
model = vi
model limit = NULL
pop = FIN
Predicted
True
049:01
027:01
018:01
017
016
012:03
012:01
011:01
010:01
009:02
009:01
008:01/04
007:01
004:01
002:01
001
1
82
6
37
225
27
1
63
1
4
4
1
9
20
11
6

gene = MICA
model = vii
model limit = NULL
pop = EUR

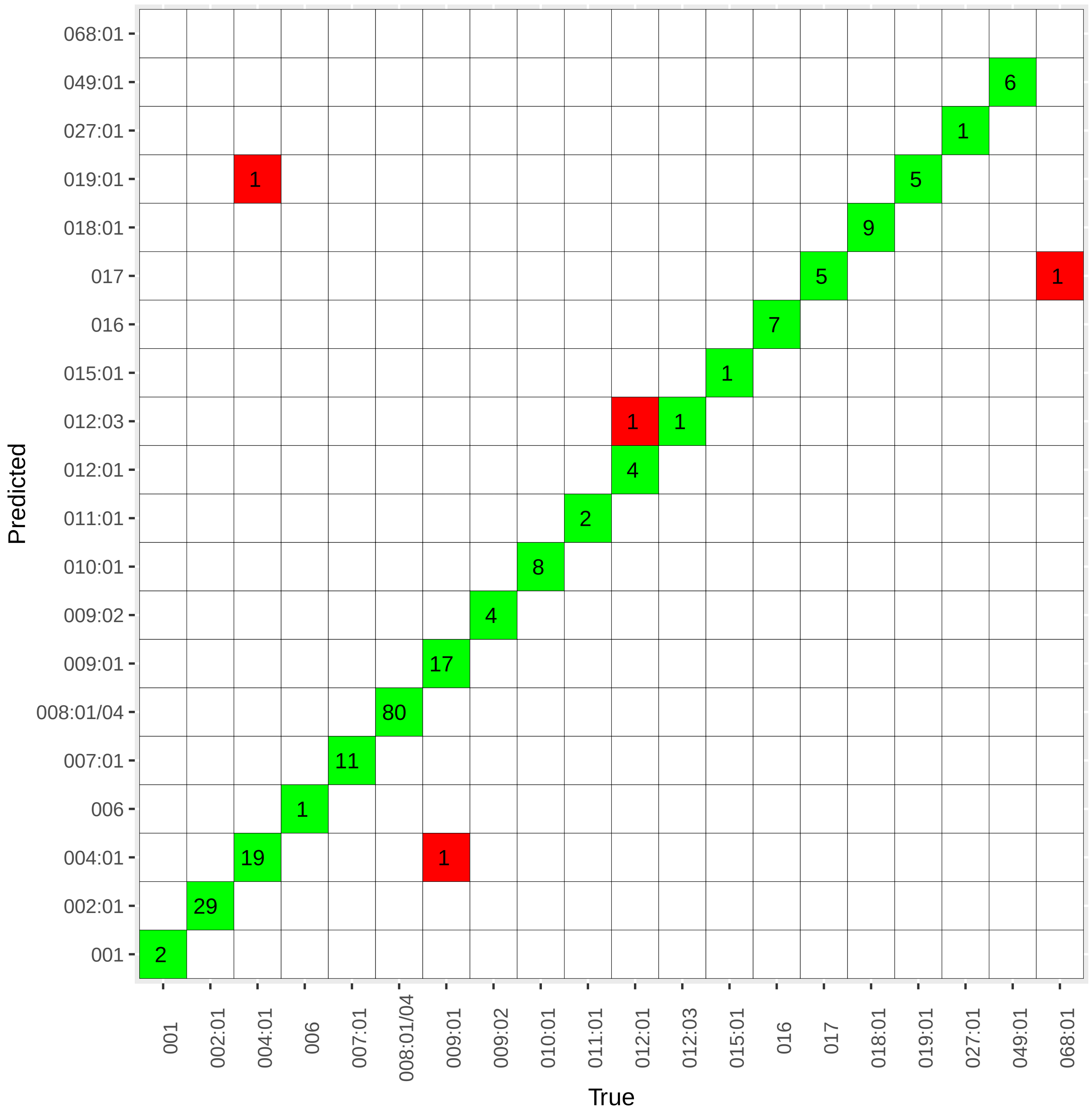

gene = MICA
model = vii
model limit = NULL
pop = AFR

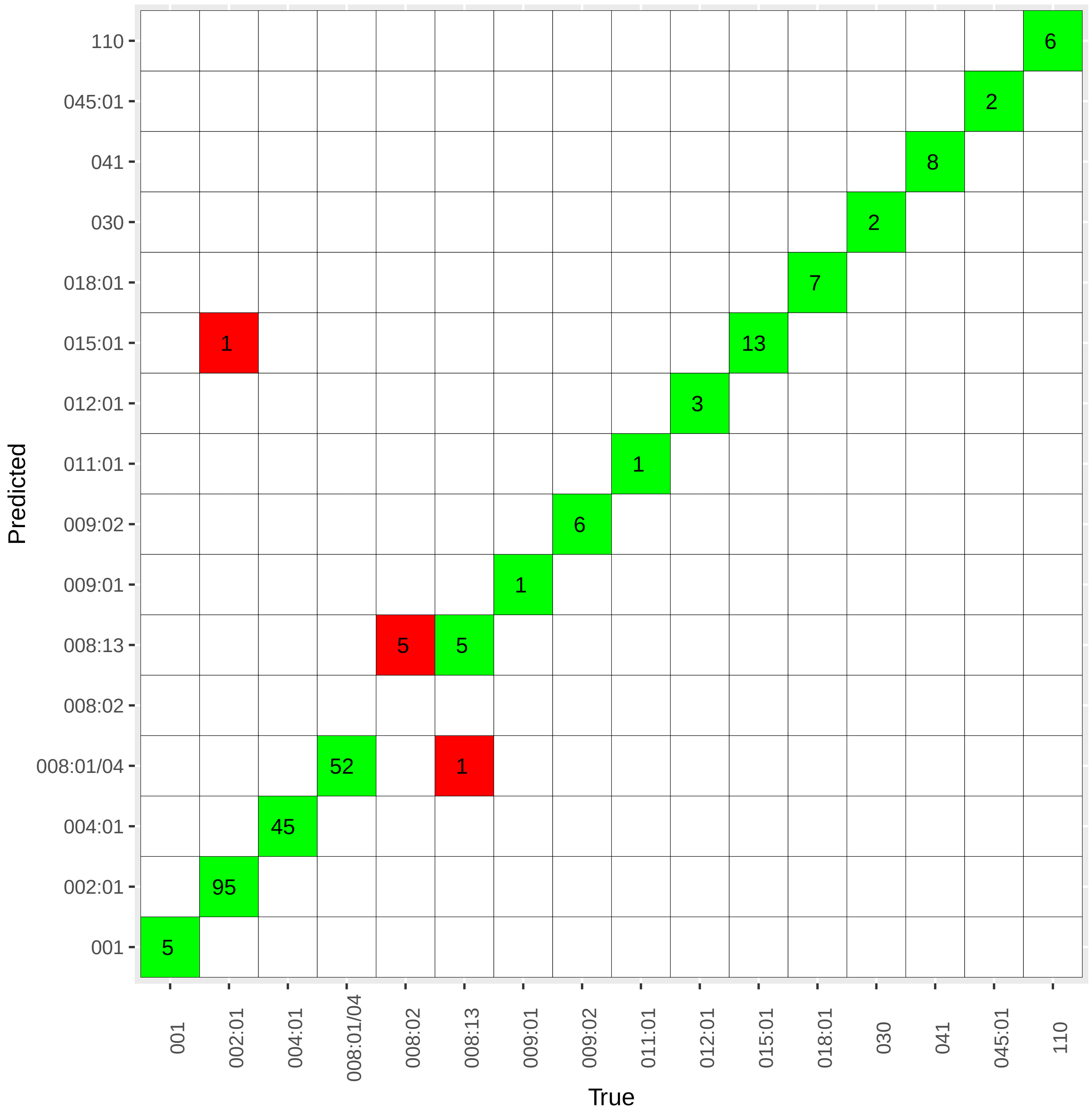

gene = MICA
model = vii
model limit = NULL
pop = EAS

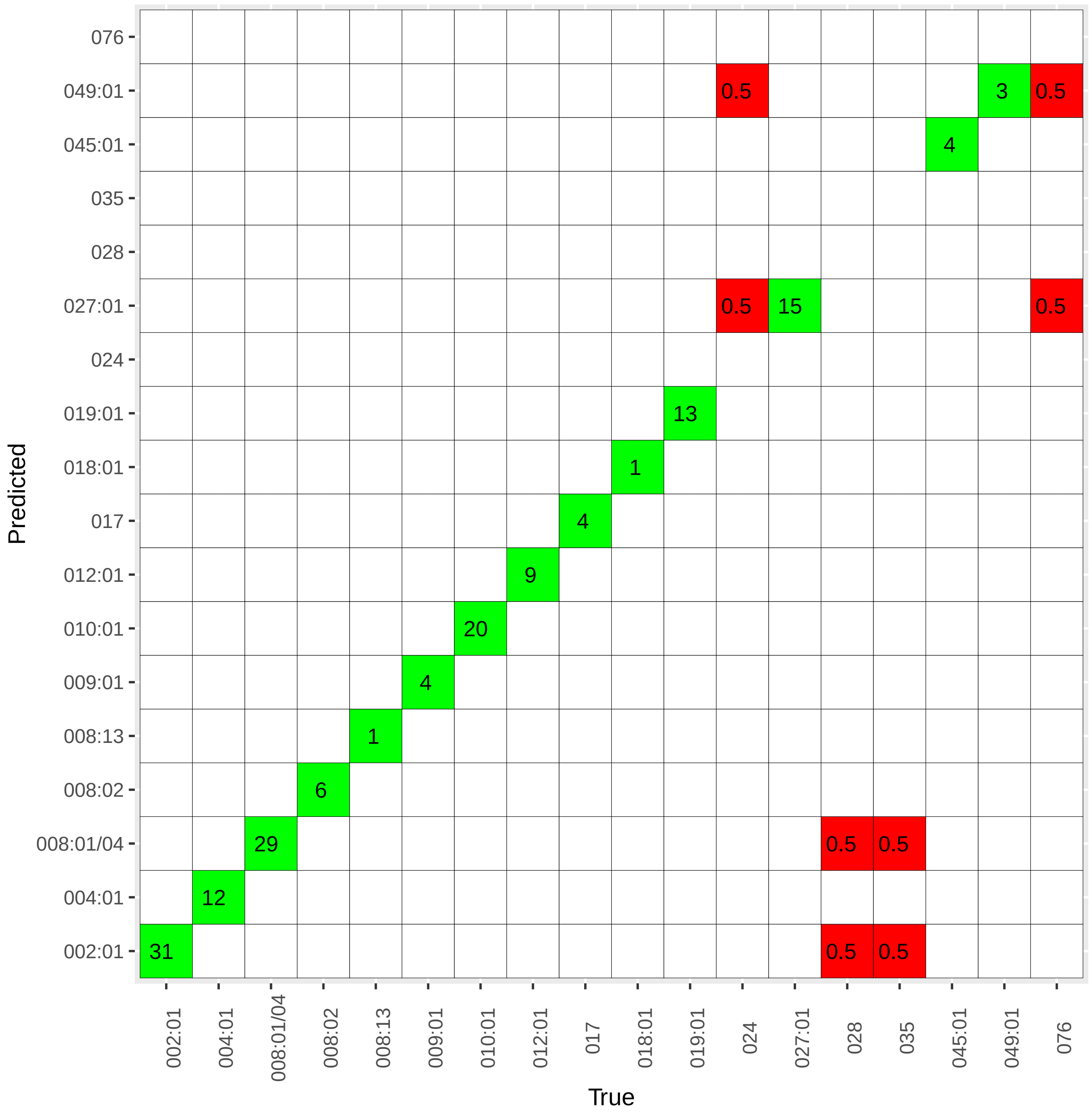

gene = MICA
model = vii
model limit = NULL
pop = SAS

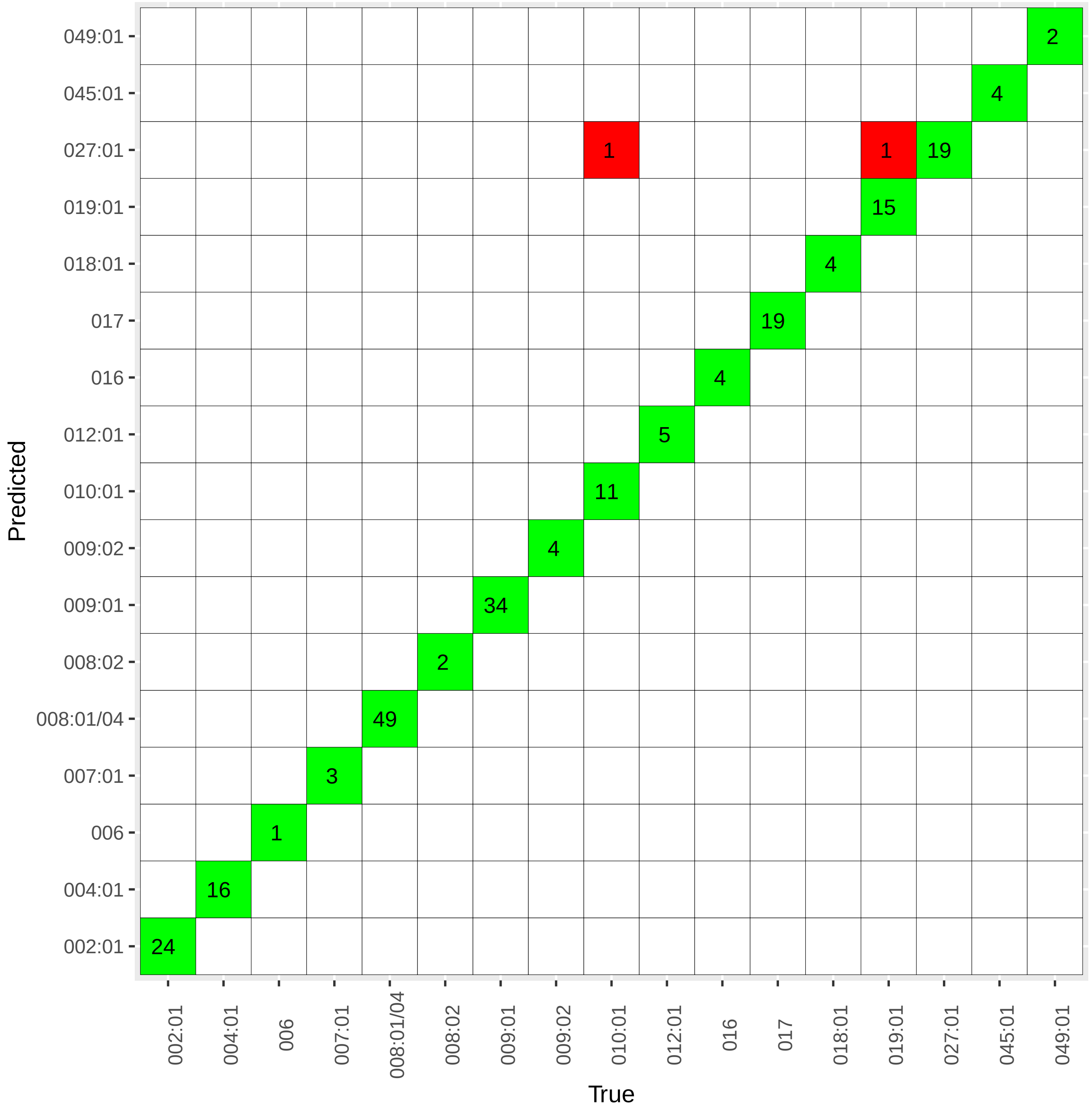

gene = MICA
model = vii
model limit = NULL
pop = AMR

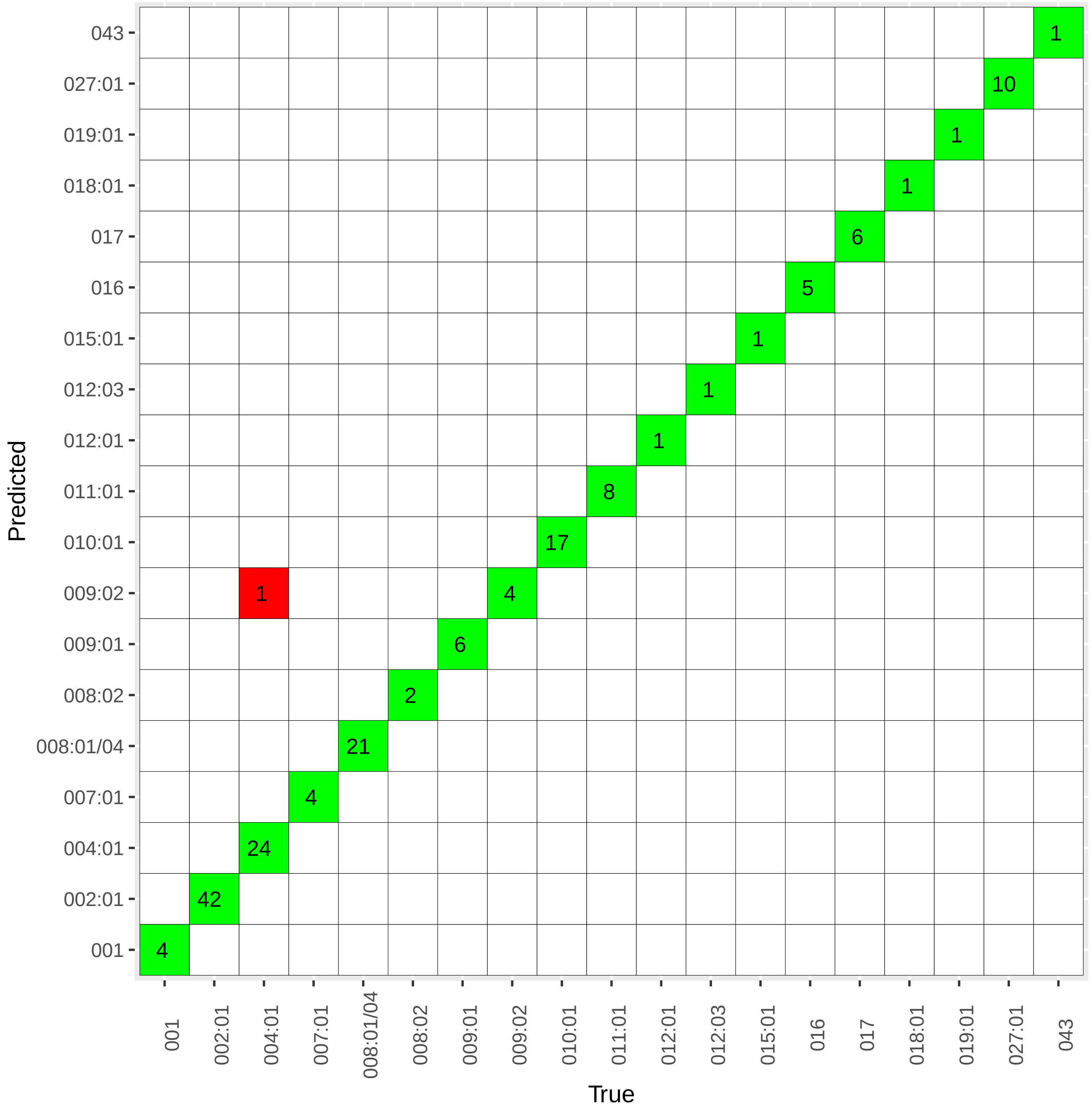

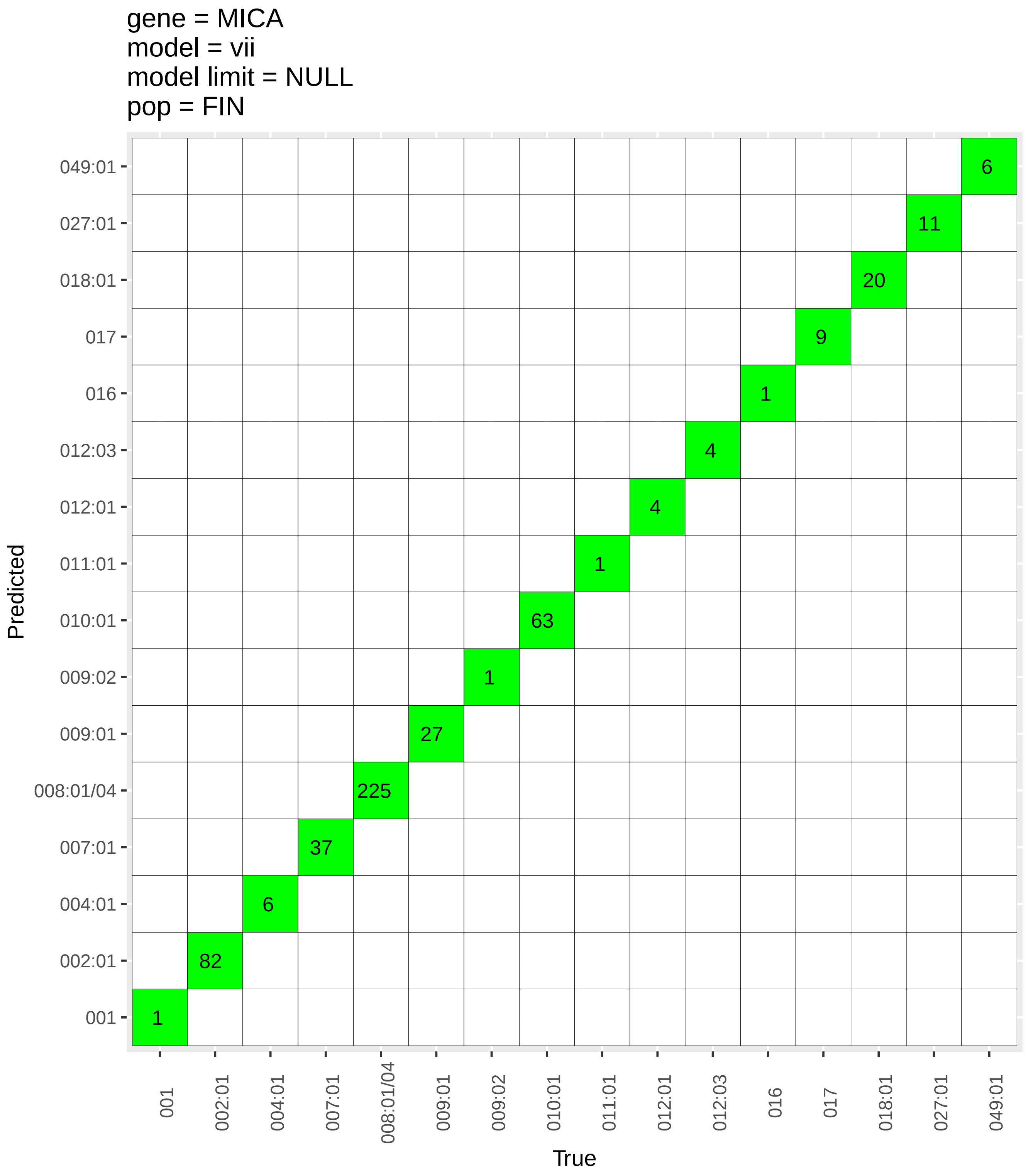
gene = MICA
model = vii
model limit = NULL
pop = FIN
Predicted
049:01
027:01
018:01
017
016
012:03
012:01
011:01
010:01
009:02
009:01
008:01/04
007:01
004:01
002:01
001
6
11
20
9
1
4
4
1
63
1
27
225
37
6
82
1
001
002:01
004:01
007:01
008:01/04
009:01
009:02
010:01
011:01
012:01
012:03
016
017
018:01
027:01
049:01
True

gene = MICB
model = i
model limit = NULL
pop = EUR

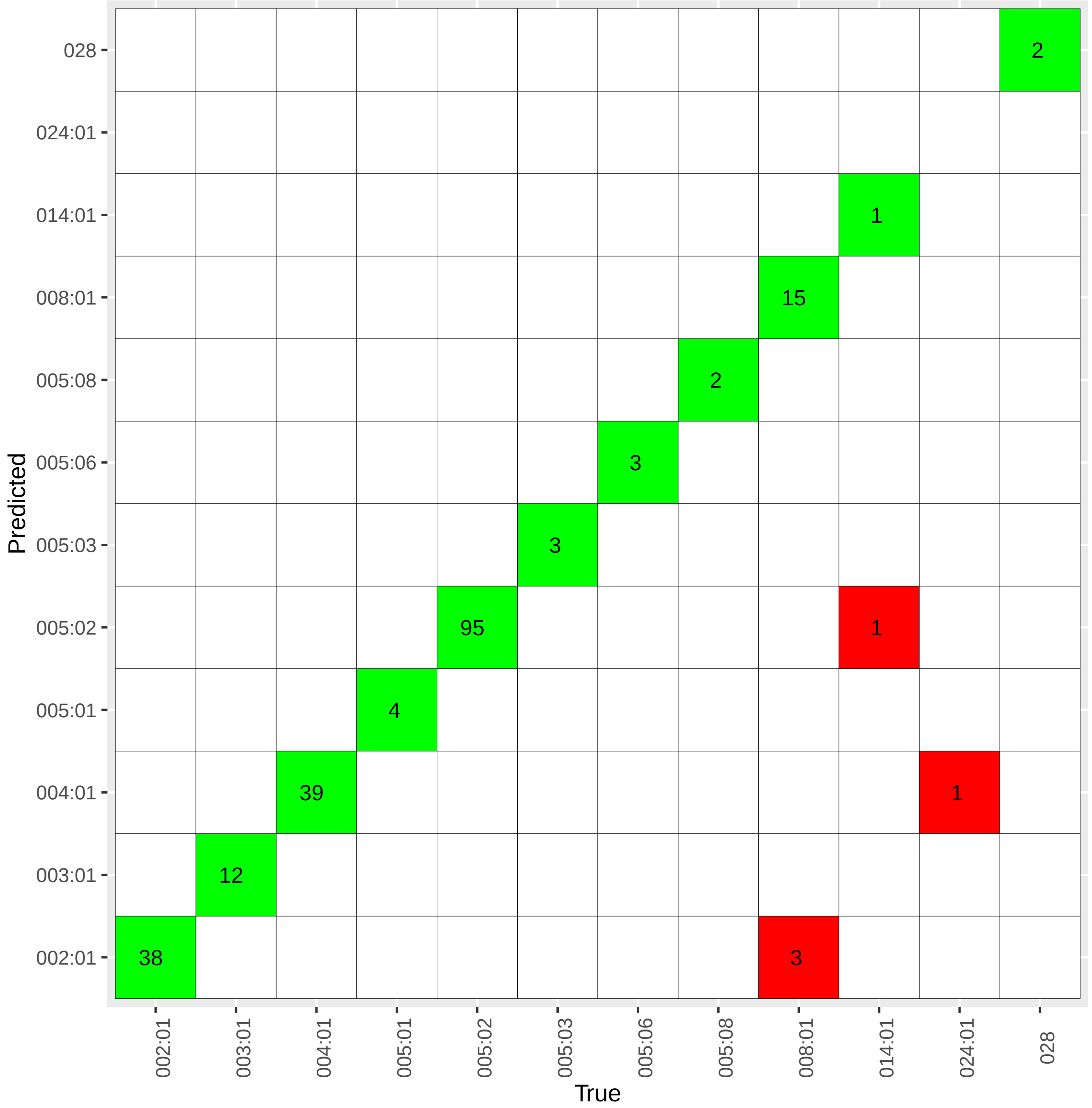

gene = MICB
model = i
model limit = NULL
pop = AFR

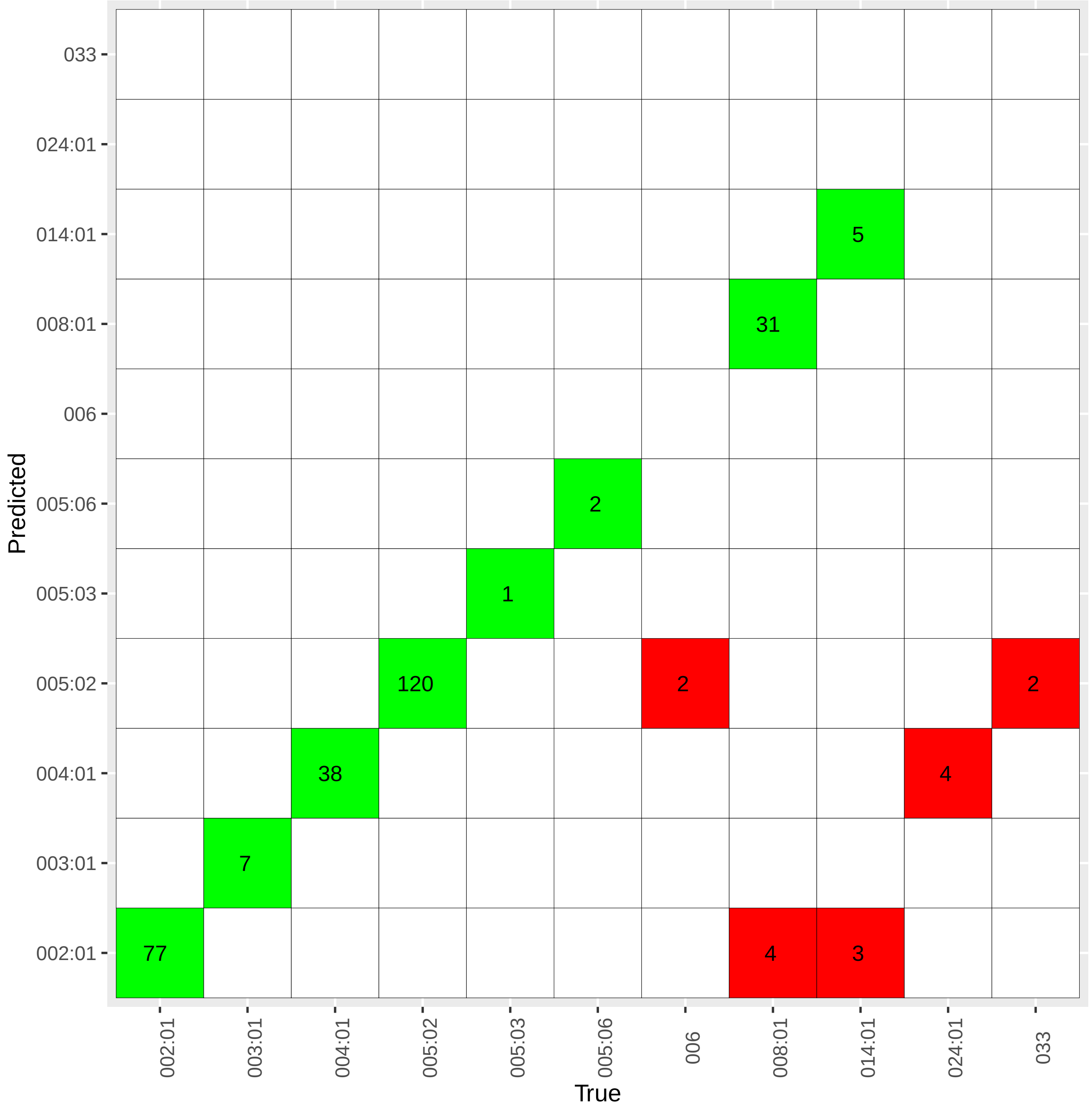

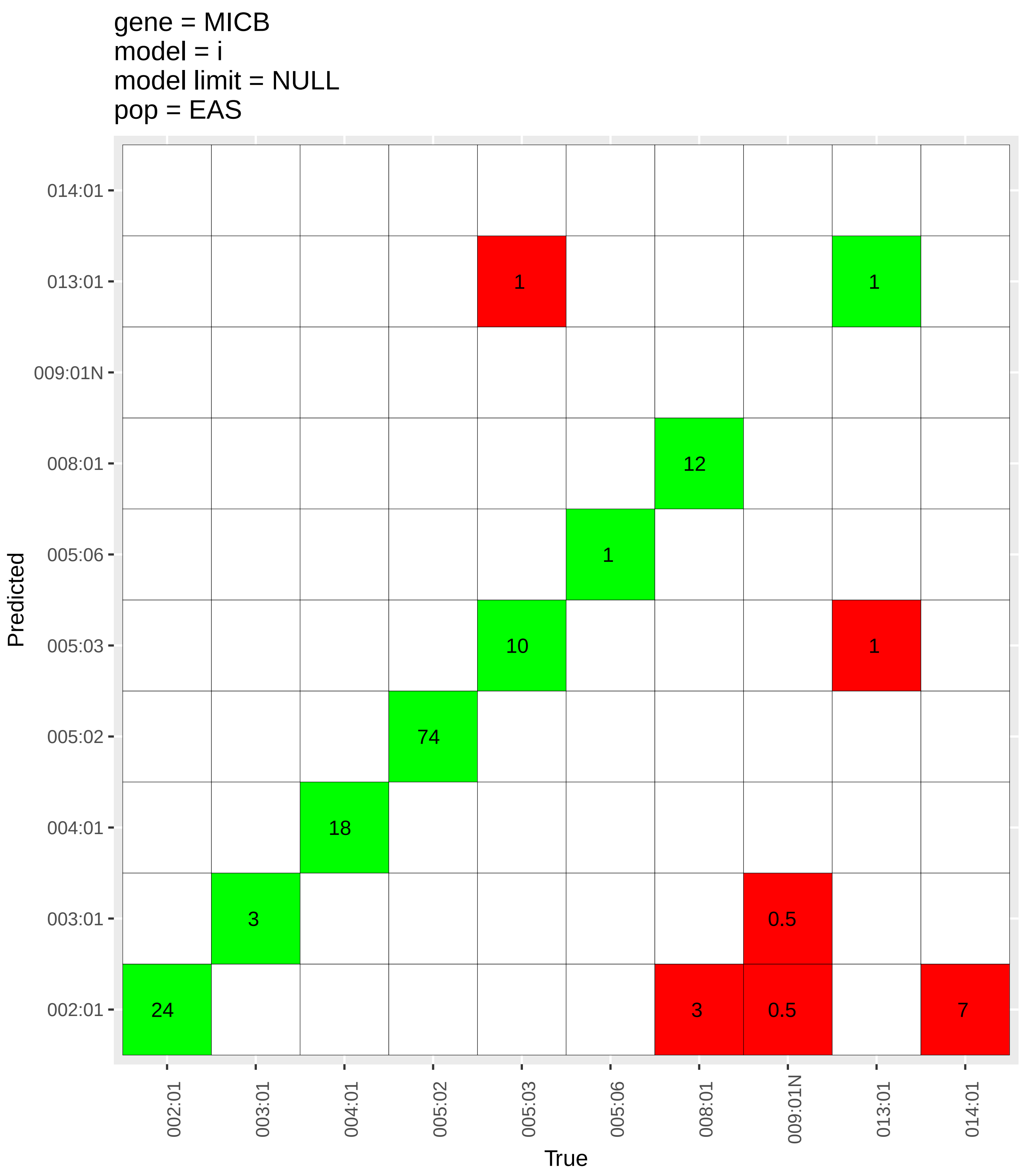
gene = MICB
model = i
model limit = NULL
pop = EAS
Predicted
014:01
013:01
009:01N
008:01
005:06
005:03
005:02
004:01
003:01
002:01
1
1
12
1
10
1
74
18
3
0.5
24
3
0.5
7
002:01
003:01
004:01
005:02
005:03
005:06
008:01
009:01N
013:01
014:01
True

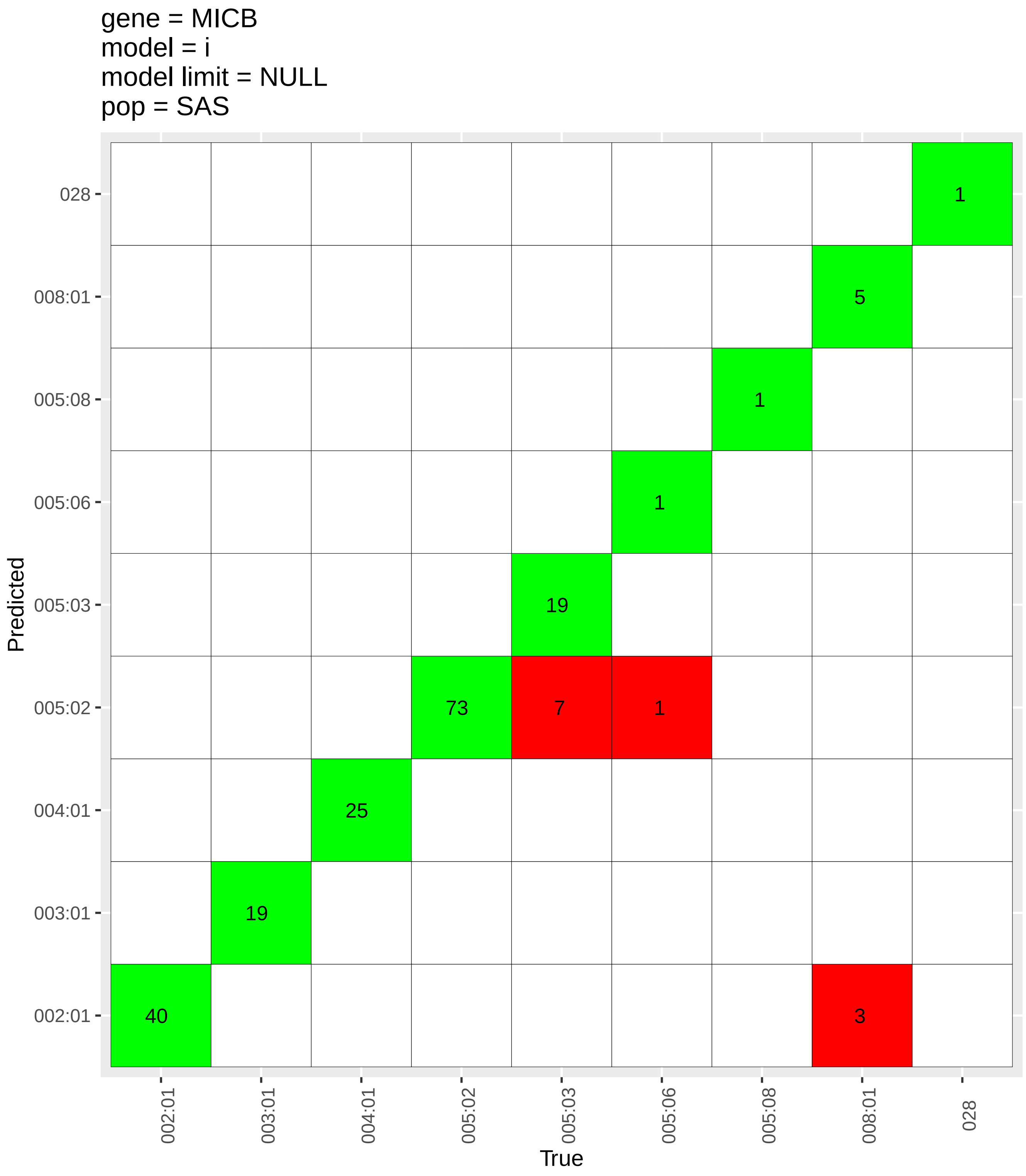

gene = MICB
model = i
model limit = NULL
pop = SAS
Predicted
True
028
008:01
005:08
005:06
005:03
005:02
004:01
003:01
002:01
1
5
1
1
19
73
7
1
25
19
40
3

gene = MICB
model = i
model limit = NULL
pop = AMR

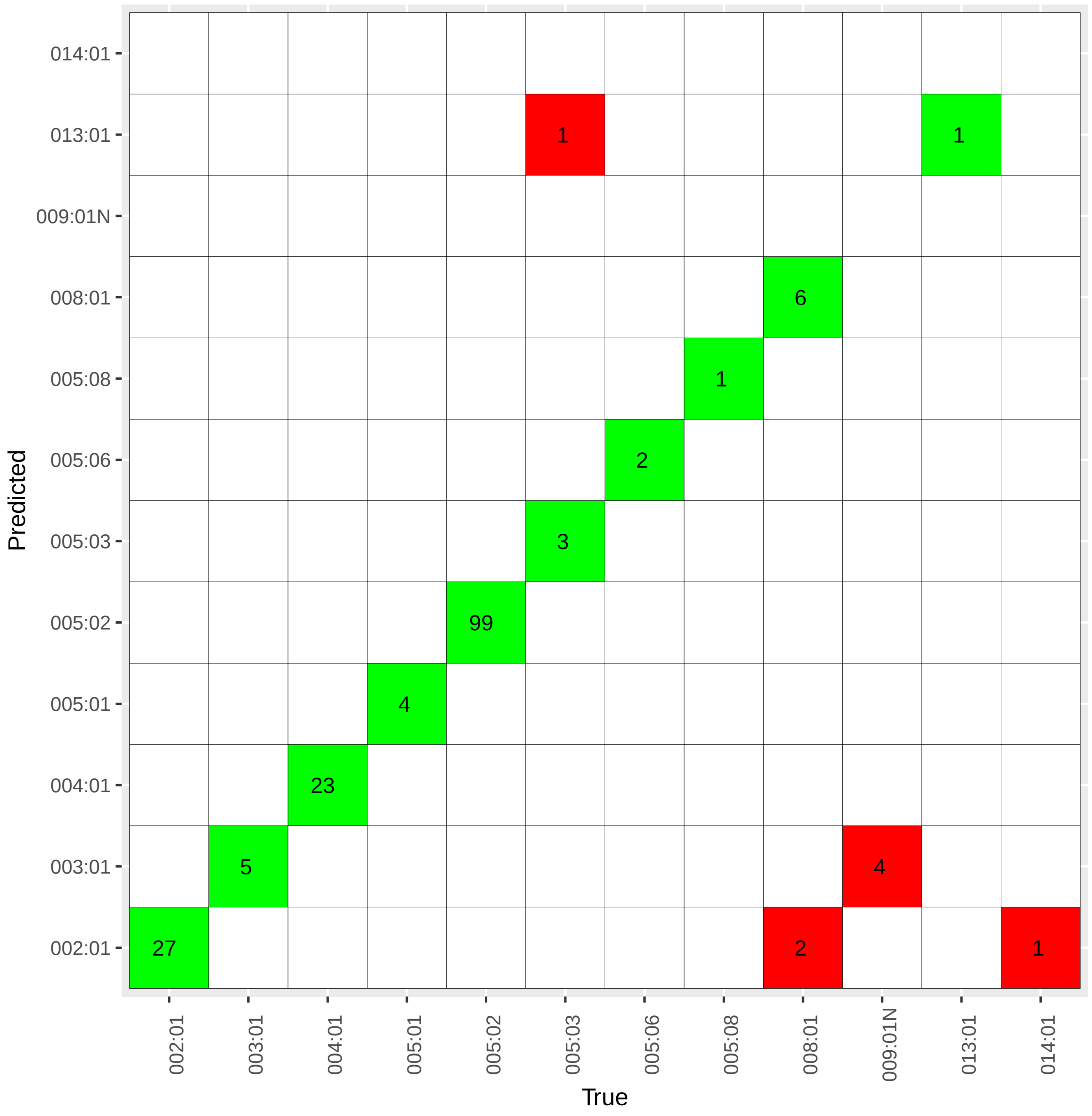

gene = MICB
model = i
model limit = NULL
pop = FIN

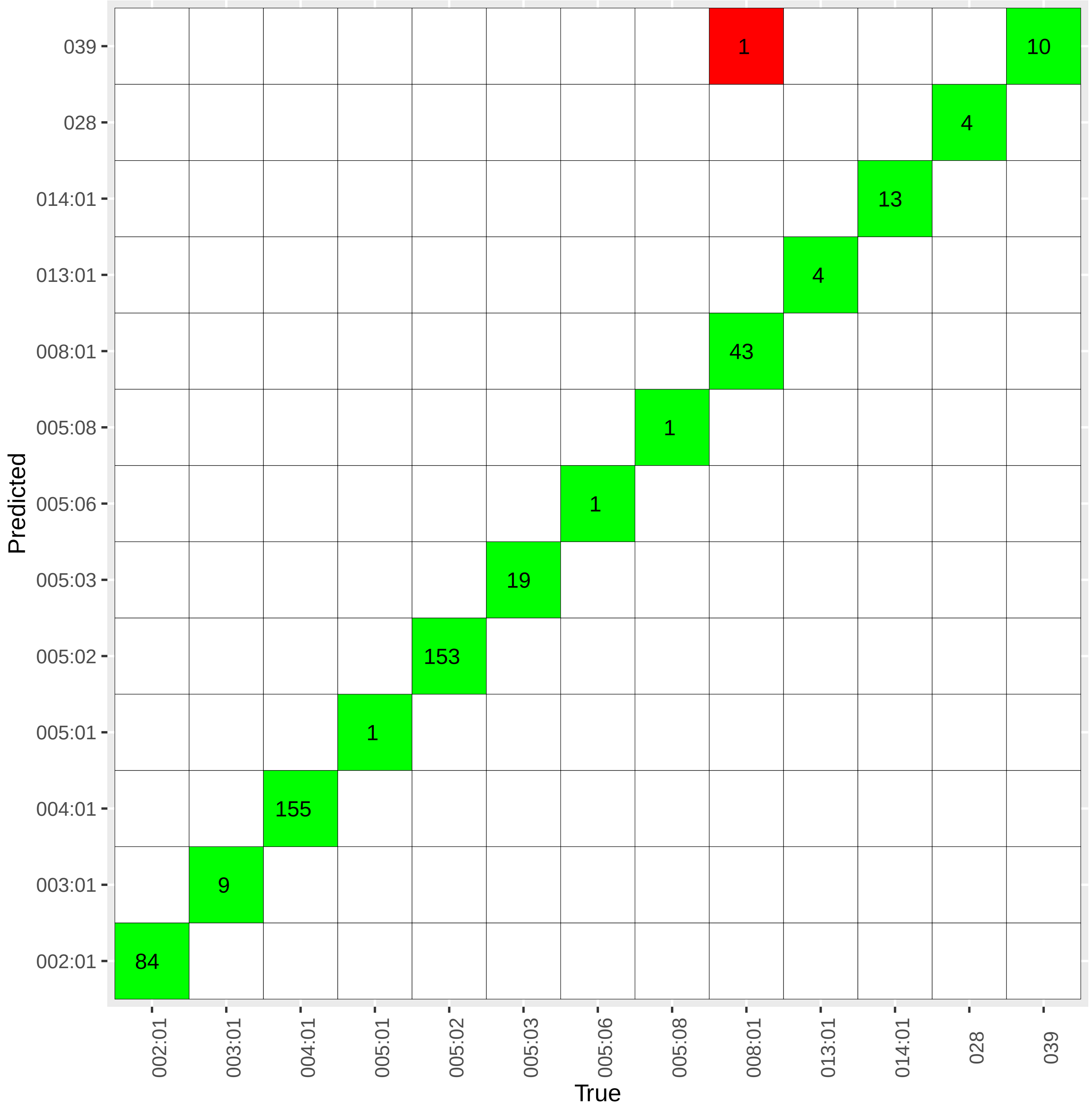

gene = MICB
model = ii
model limit = NULL
pop = EUR

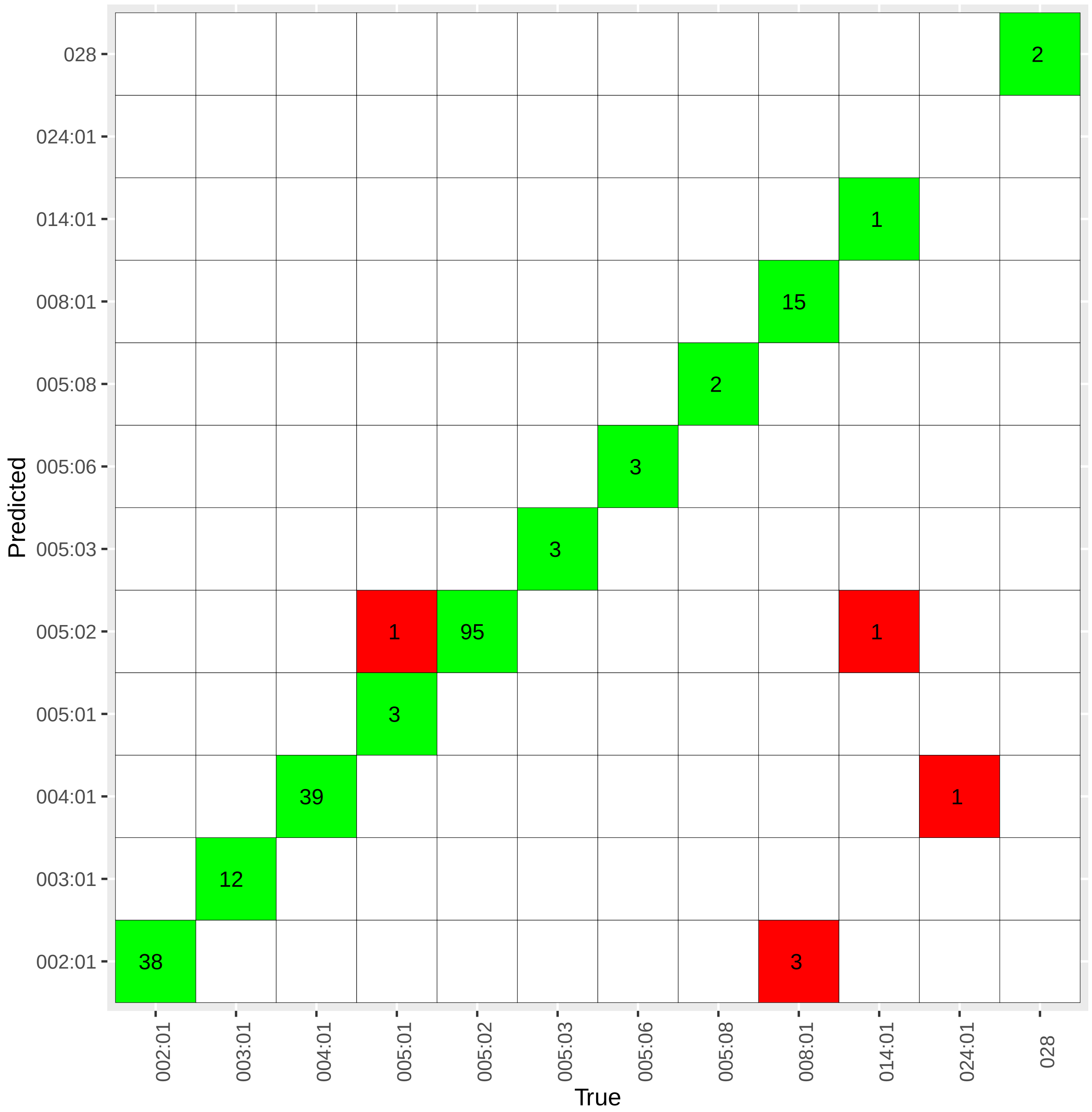

gene = MICB
model = ii
model limit = NULL
pop = AFR

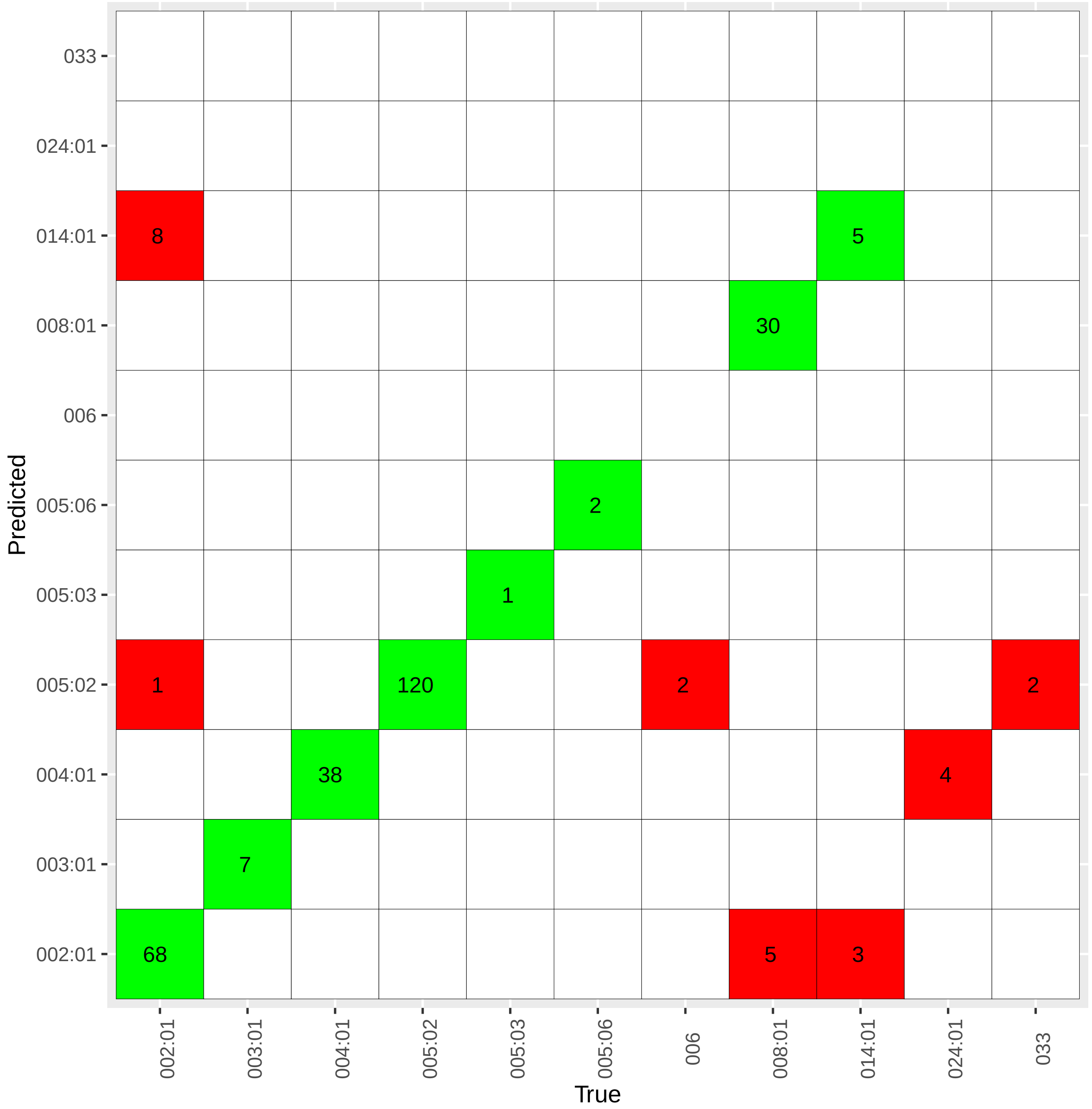

gene = MICB
model = ii
model limit = NULL
pop = EAS

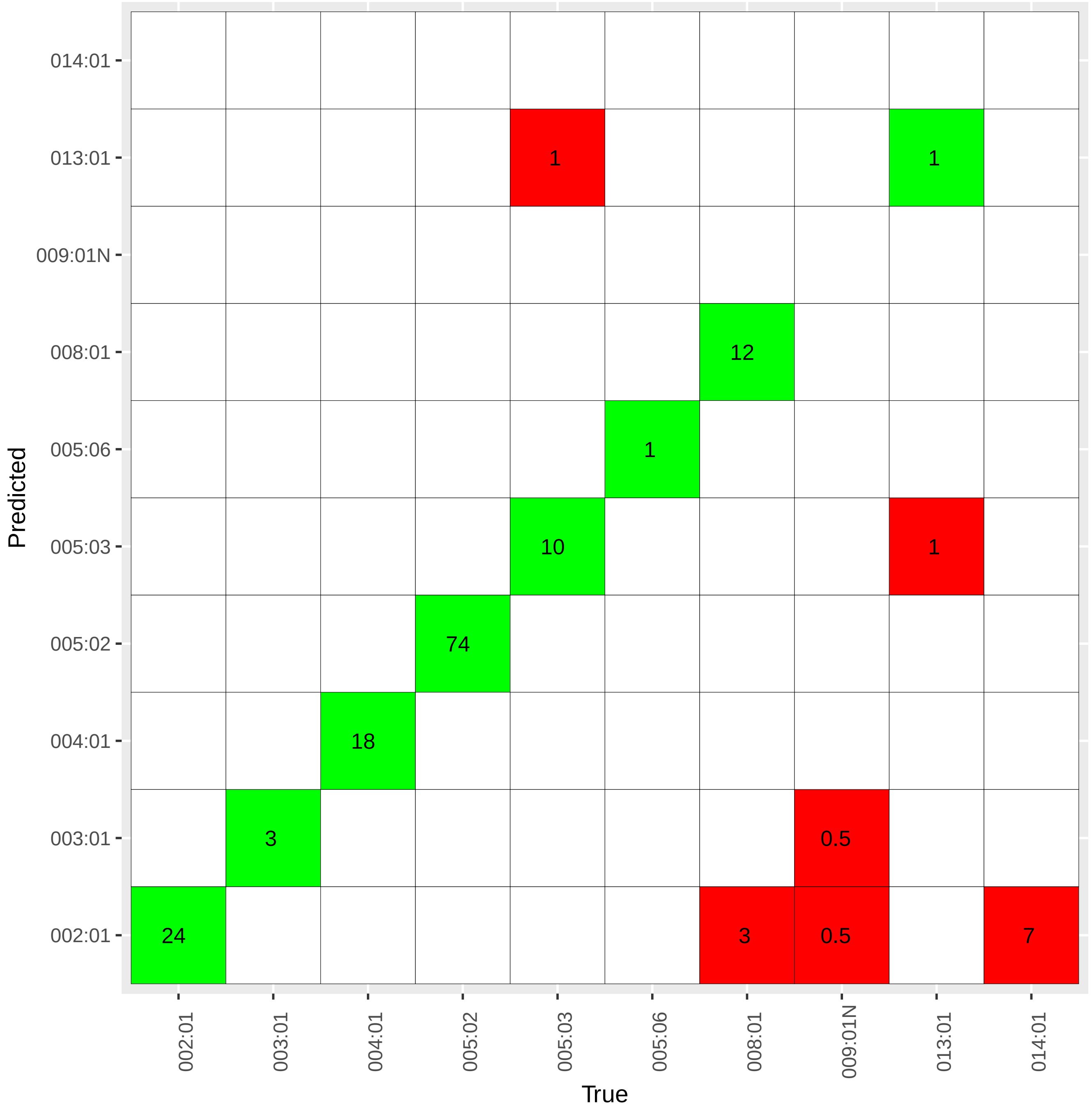

gene = MICB
model = ii
model limit = NULL
pop = SAS

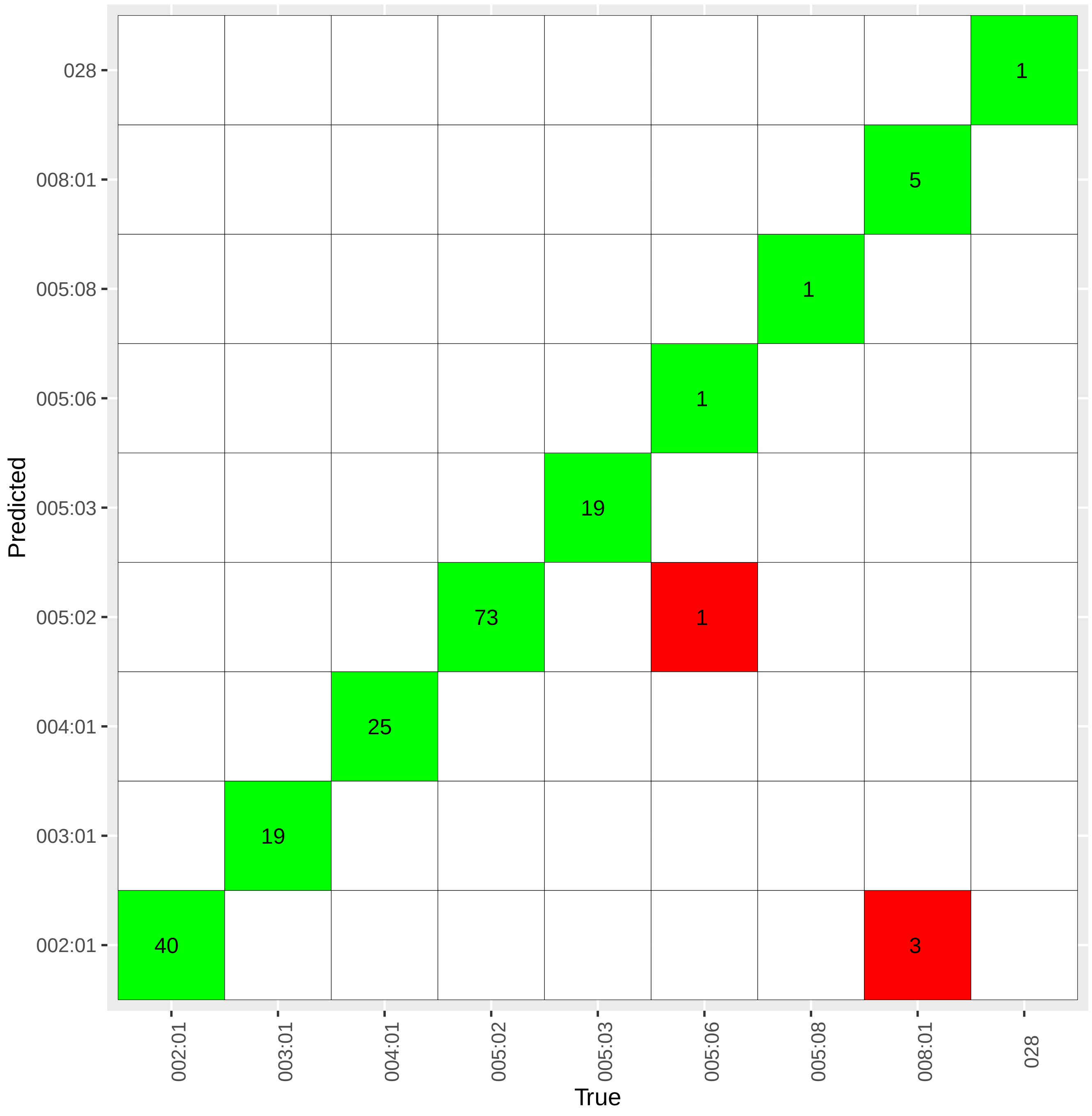

gene = MICB
model = ii
model limit = NULL
pop = AMR

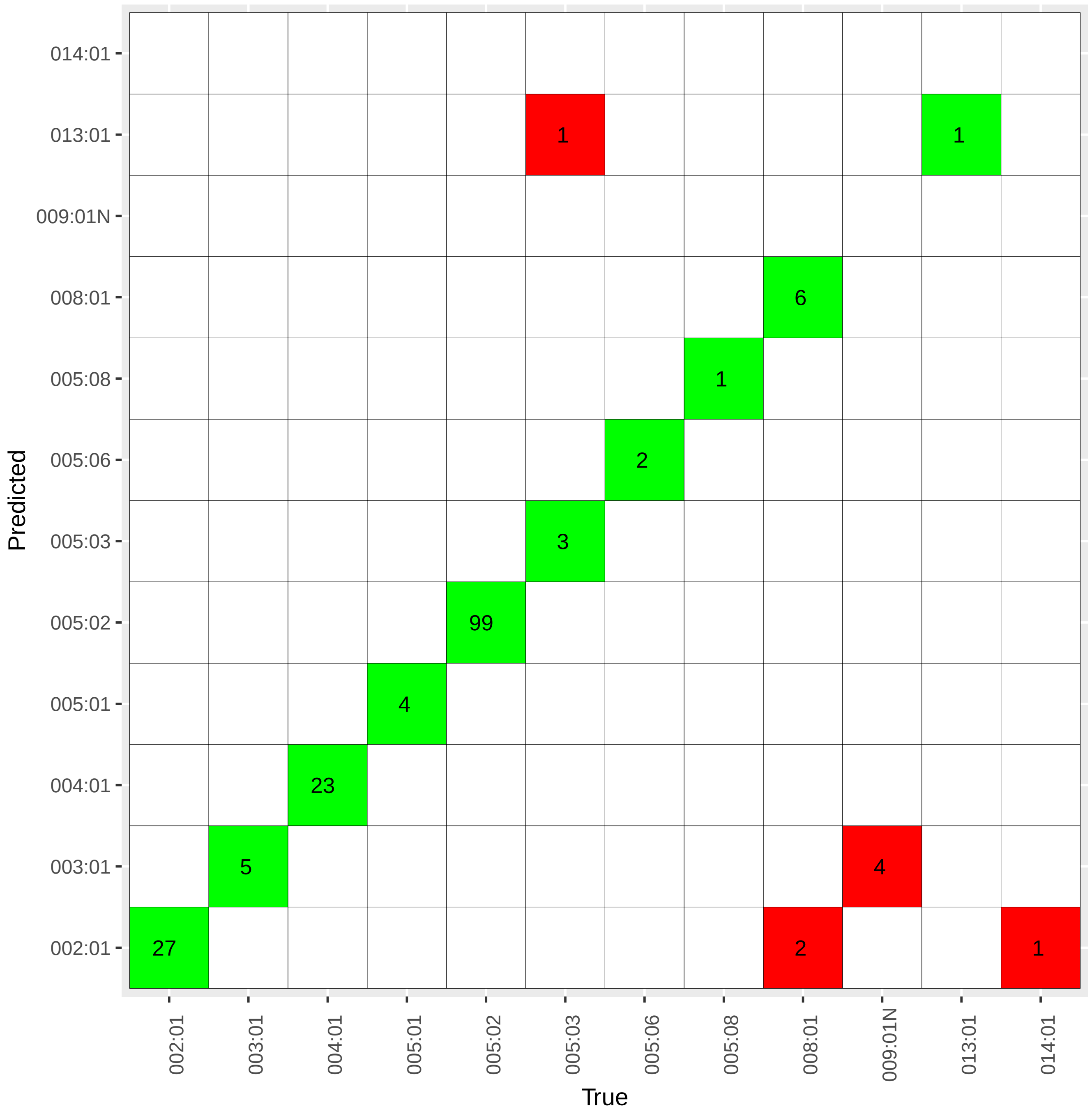

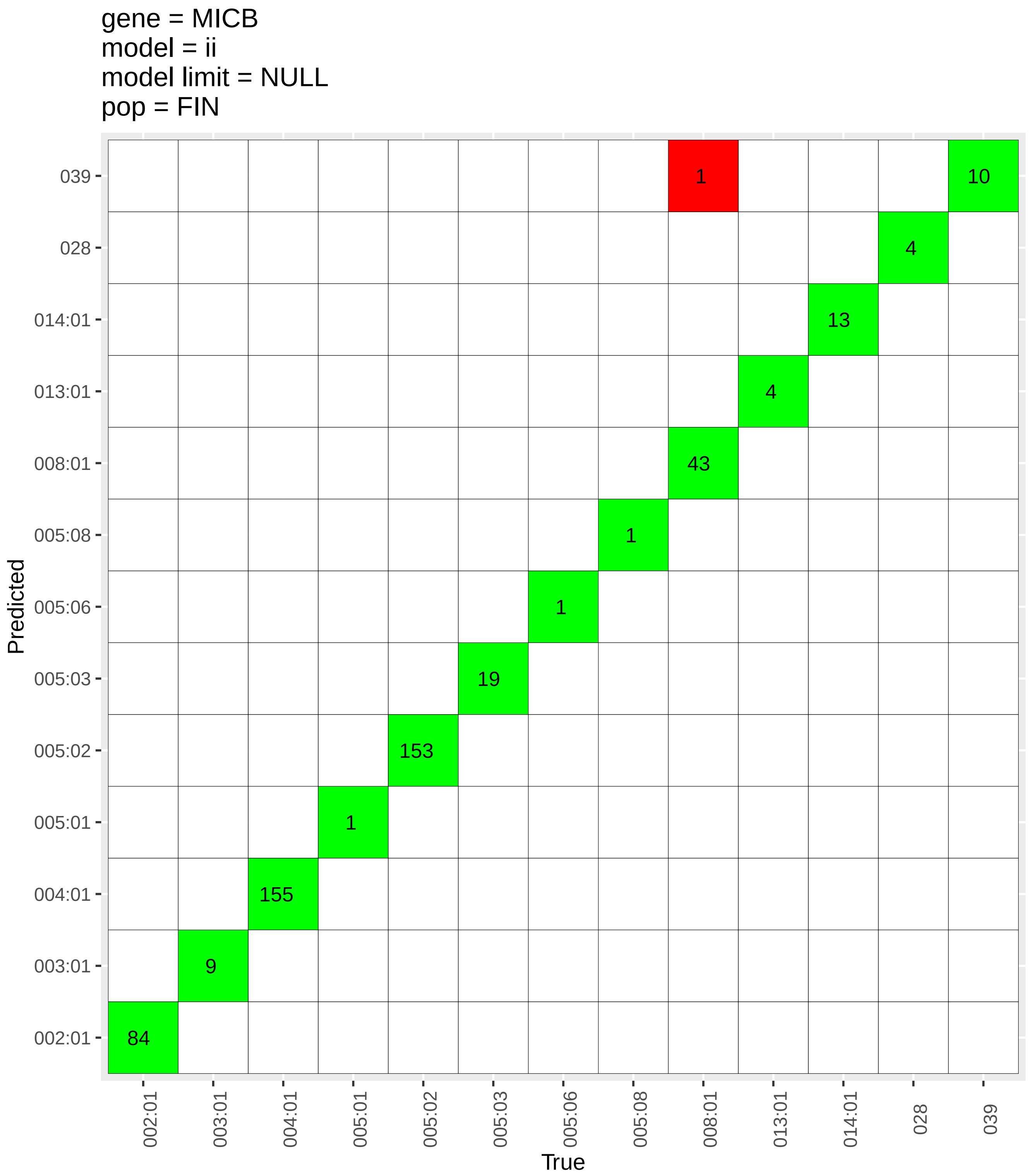
gene = MICB
model = ii
model limit = NULL
pop = FIN
Predicted
039
028
014:01
013:01
008:01
005:08
005:06
005:03
005:02
005:01
004:01
003:01
002:01
1
10
4
13
4
43
1
1
19
153
1
155
9
84
True
002:01
003:01
004:01
005:01
005:02
005:03
005:06
005:08
008:01
013:01
014:01
028
039

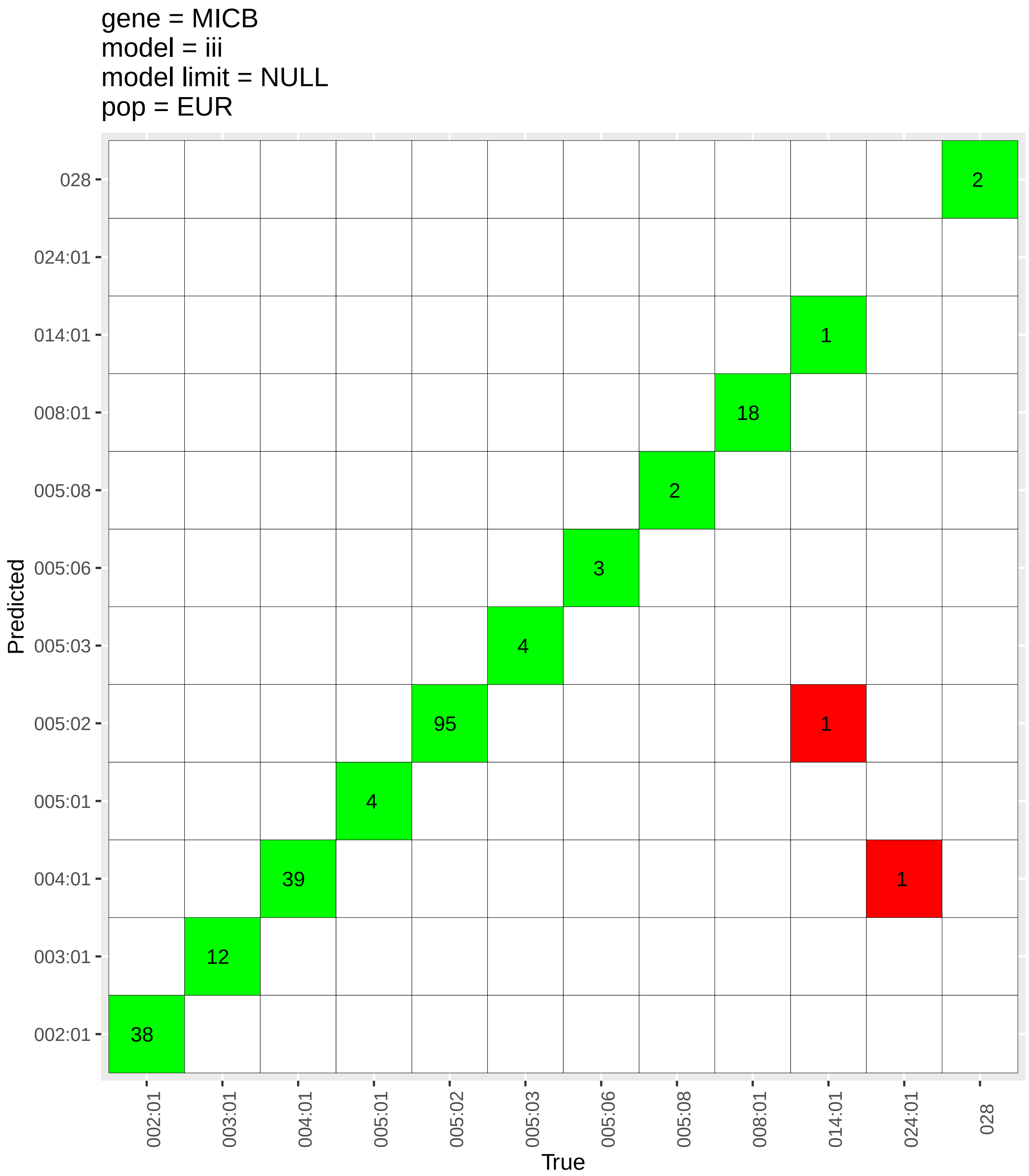
gene = MICB
model = iii
model limit = NULL
pop = EUR
Predicted
028
024:01
014:01
008:01
005:08
005:06
005:03
005:02
005:01
004:01
003:01
002:01
2
1
18
2
3
4
95
1
4
39
1
12
38
002:01
003:01
004:01
005:01
005:02
005:03
005:06
005:08
008:01
014:01
024:01
028
True

gene = MICB
model = iii
model limit = NULL
pop = AFR

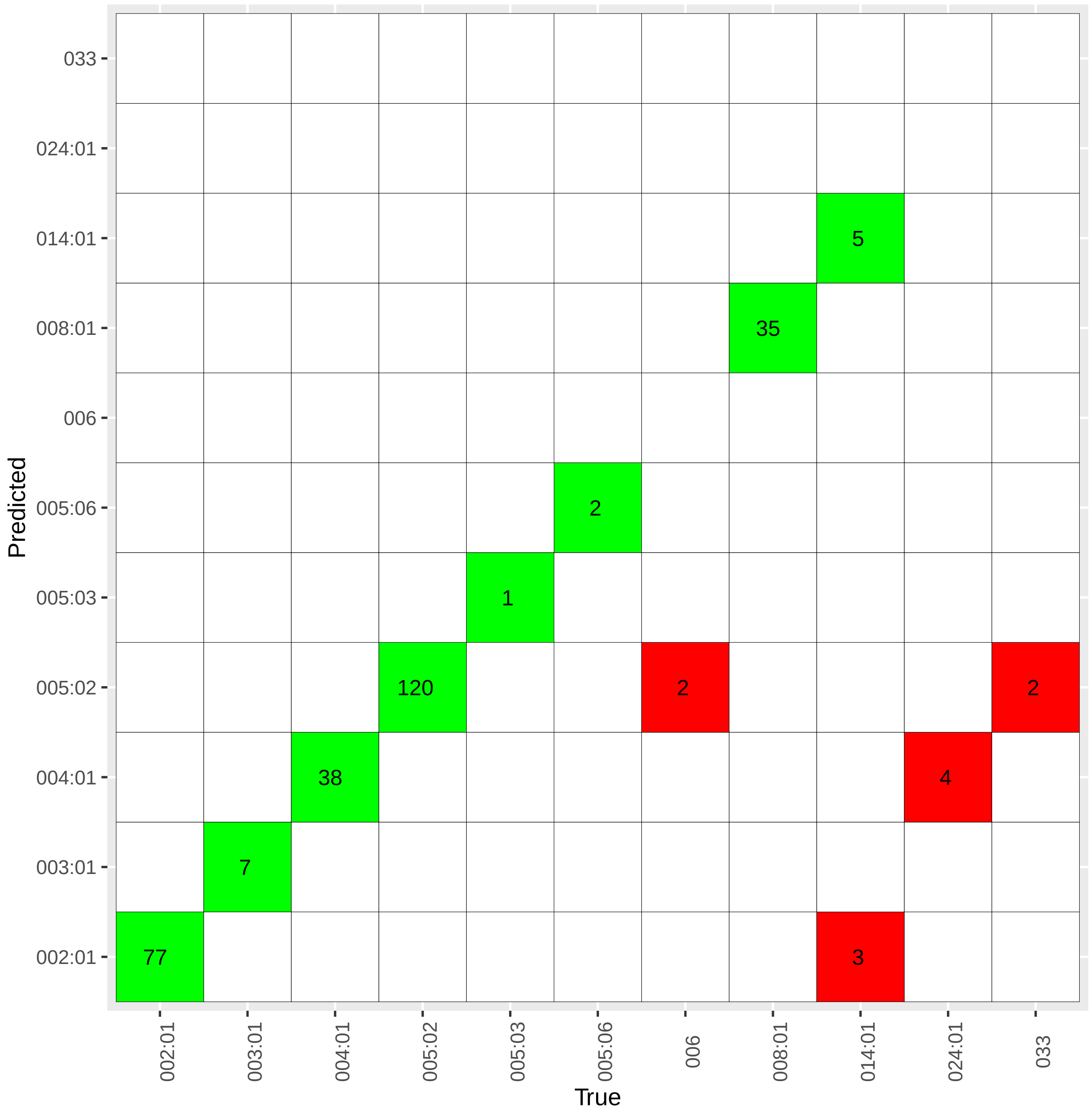

gene = MICB
model = iii
model limit = NULL
pop = EAS

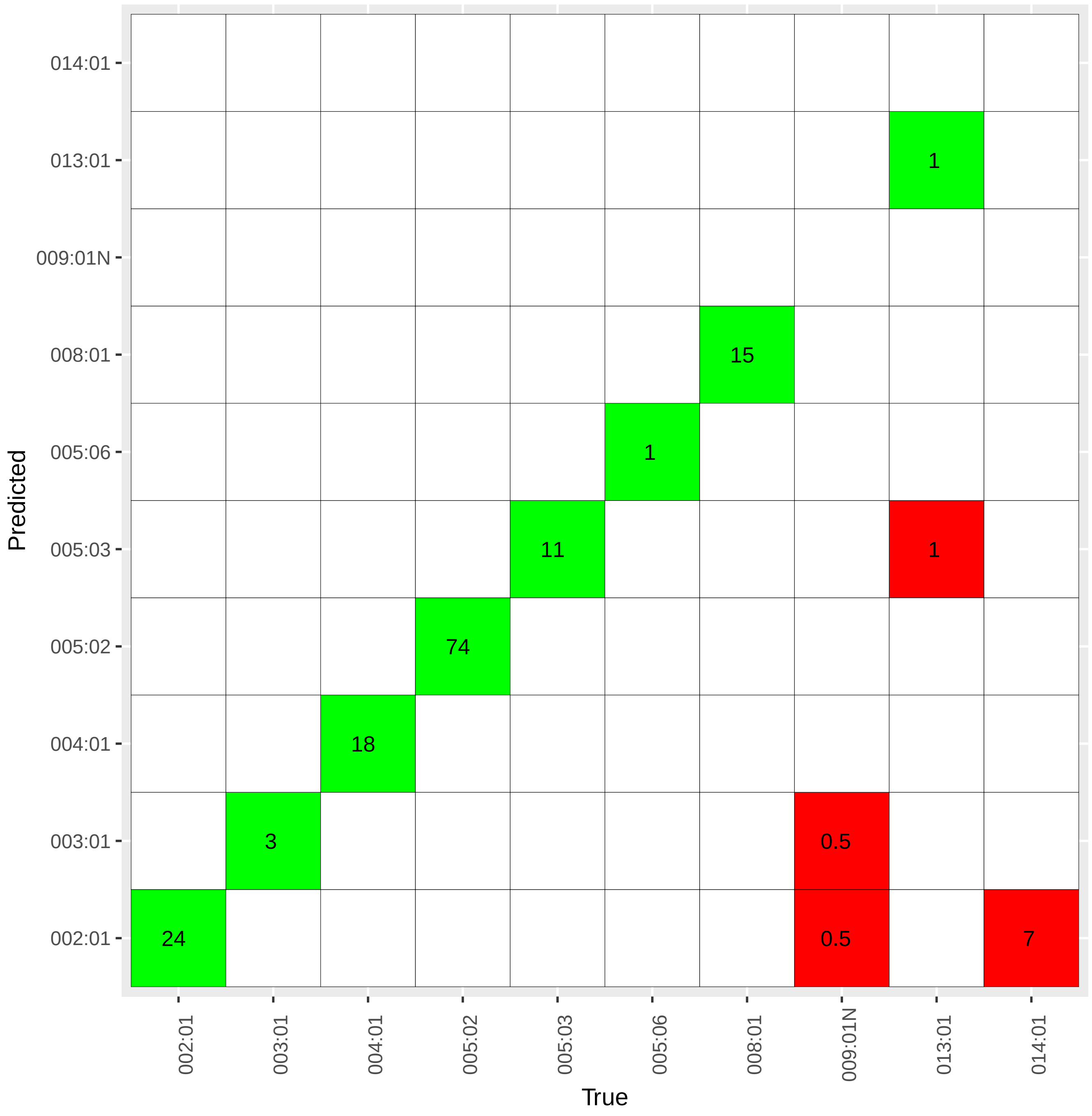

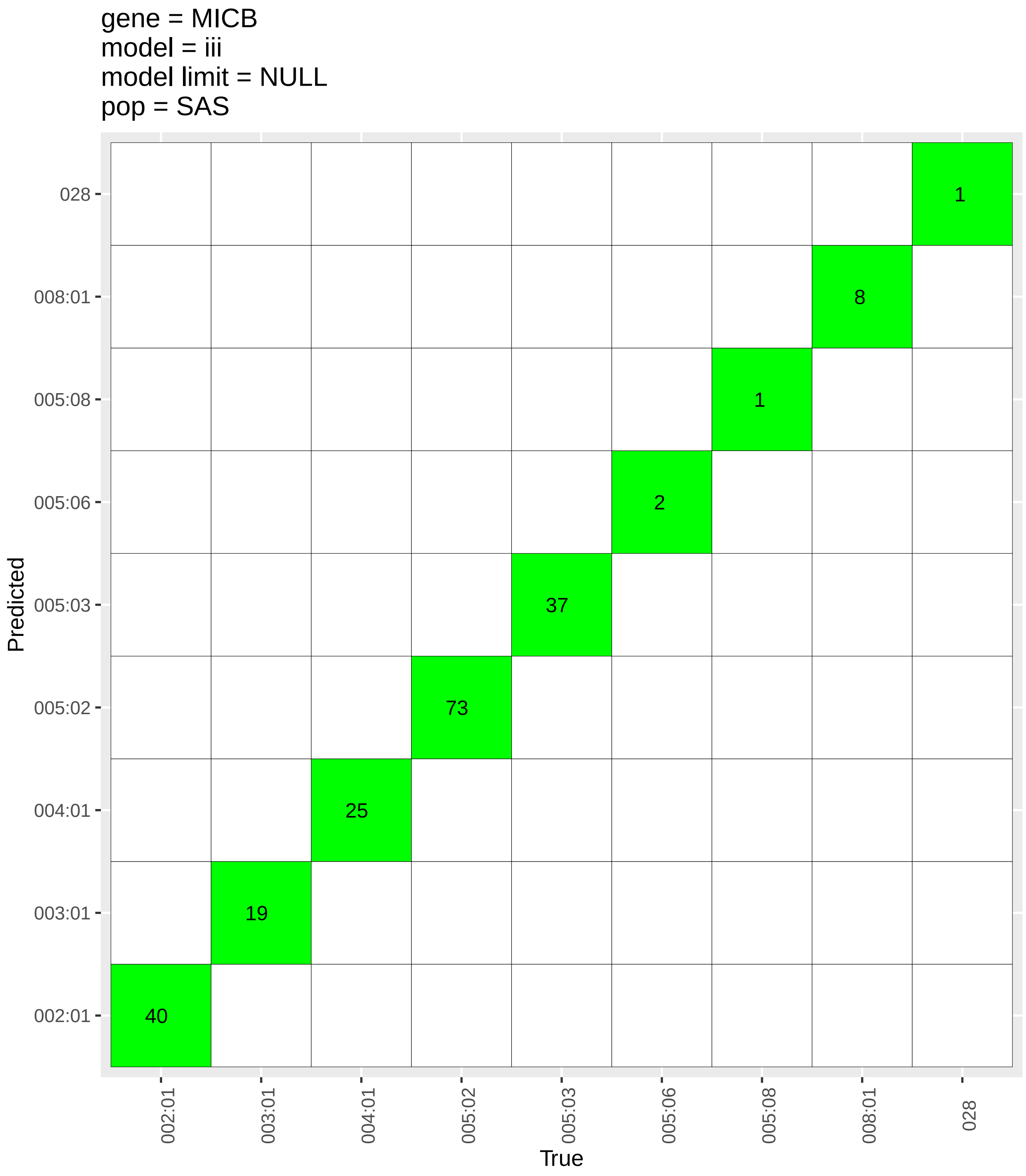
gene = MICB
model = iii
model limit = NULL
pop = SAS
Predicted
True
028
008:01
005:08
005:06
005:03
005:02
004:01
003:01
002:01
1
8
1
2
37
73
25
19
40

gene = MICB
model = iii
model limit = NULL
pop = AMR

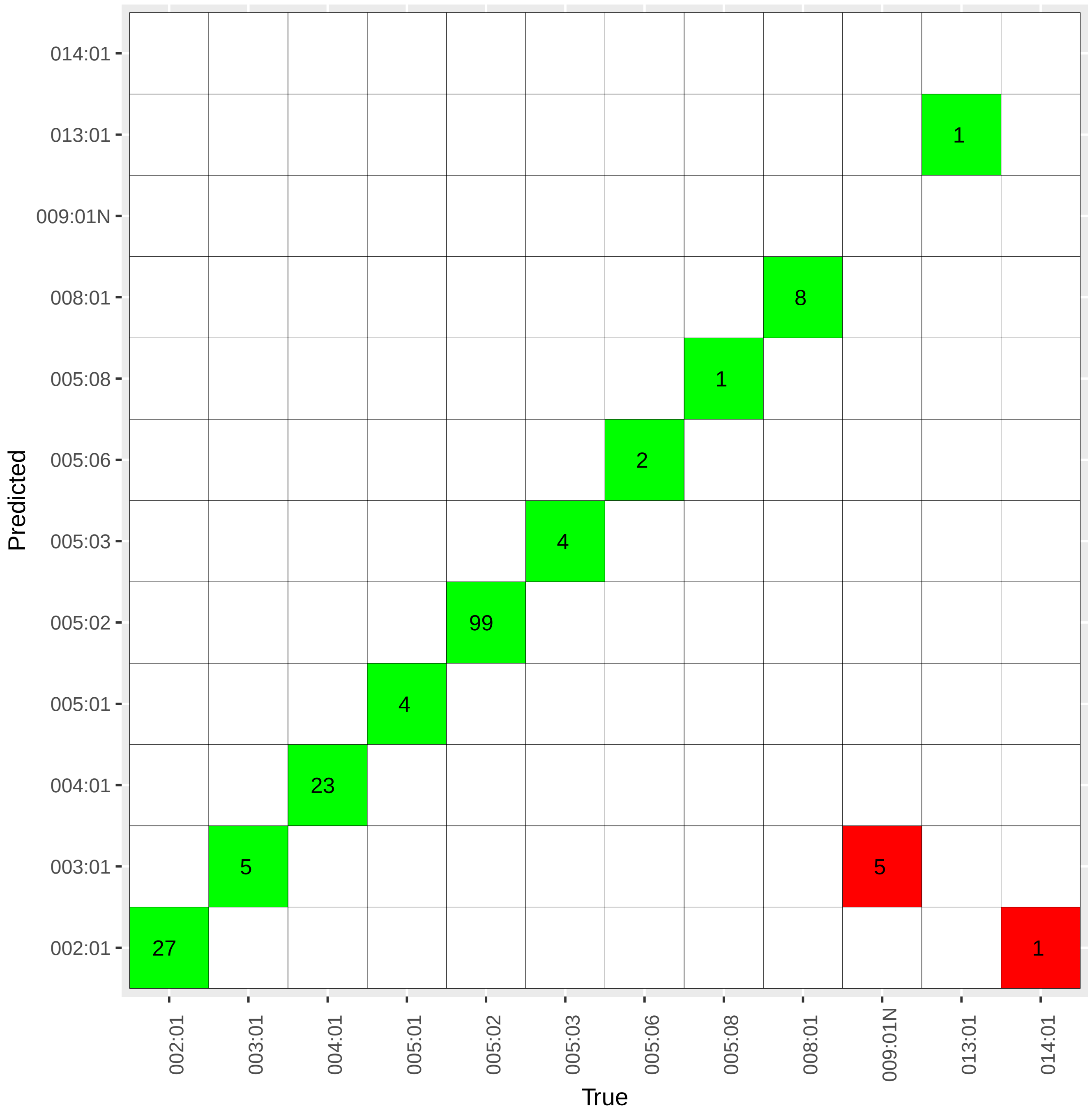

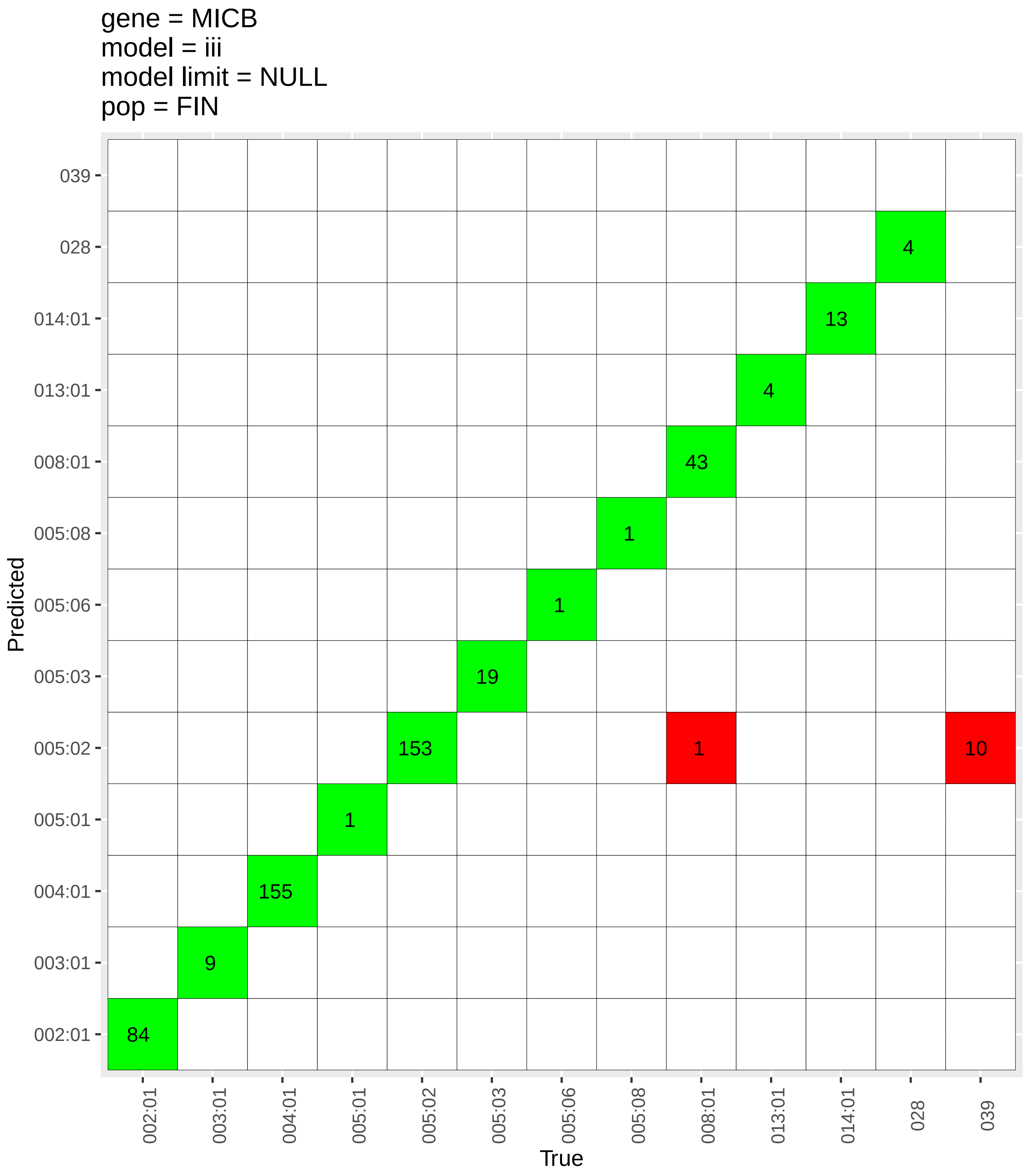
gene = MICB
model = iii
model limit = NULL
pop = FIN
Predicted
039
028
014:01
013:01
008:01
005:08
005:06
005:03
005:02
005:01
004:01
003:01
002:01
4
13
4
43
1
1
19
153
1
10
1
155
9
84
002:01
003:01
004:01
005:01
005:02
005:03
005:06
005:08
008:01
013:01
014:01
028
039
True

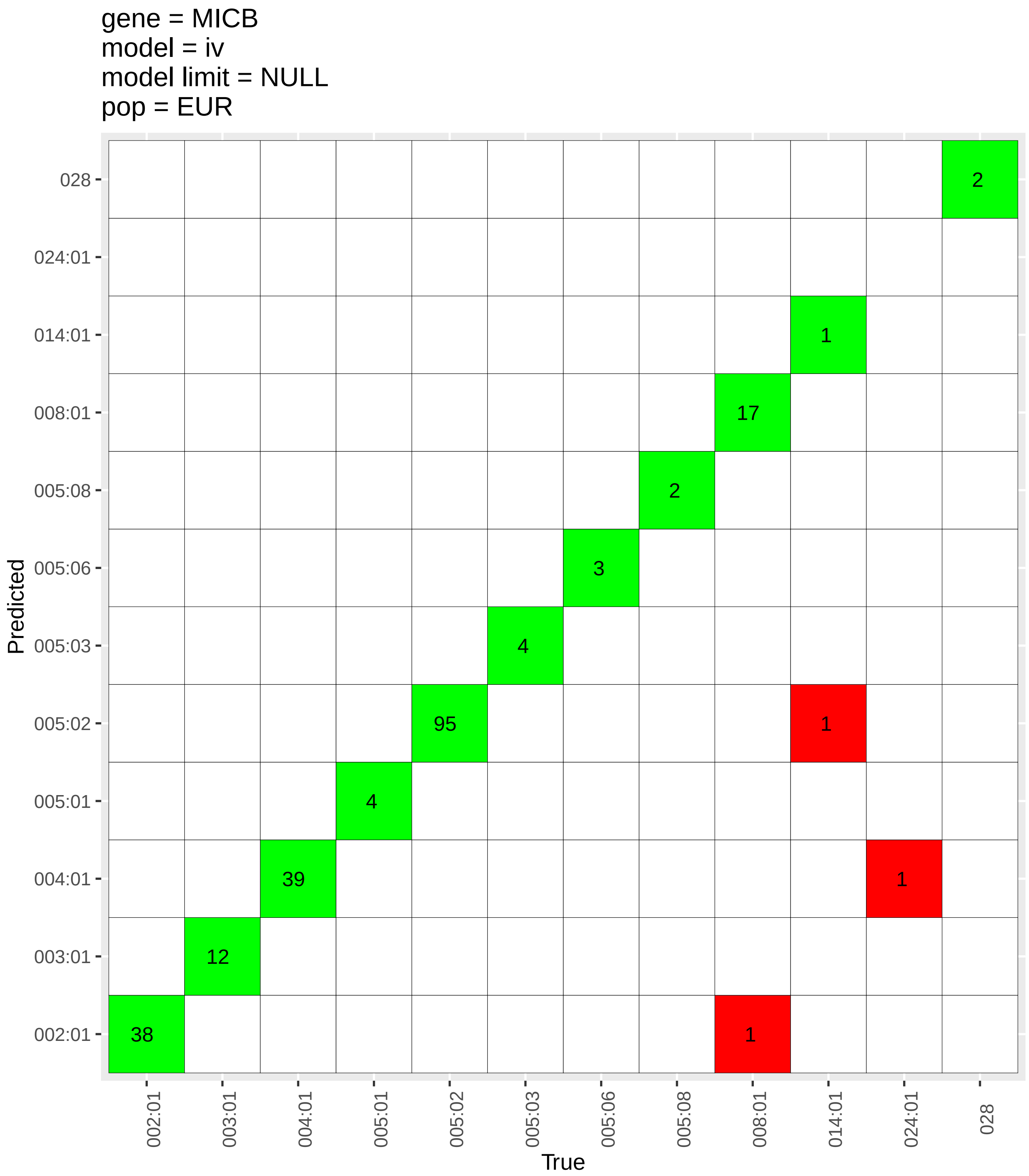
gene = MICB
model = iv
model limit = NULL
pop = EUR
Predicted
028
024:01
014:01
008:01
005:08
005:06
005:03
005:02
005:01
004:01
003:01
002:01
2
1
17
2
3
4
95
1
4
39
1
12
38
1
002:01
003:01
004:01
005:01
005:02
005:03
005:06
005:08
008:01
014:01
024:01
028
True

gene = MICB
model = iv
model limit = NULL
pop = AFR

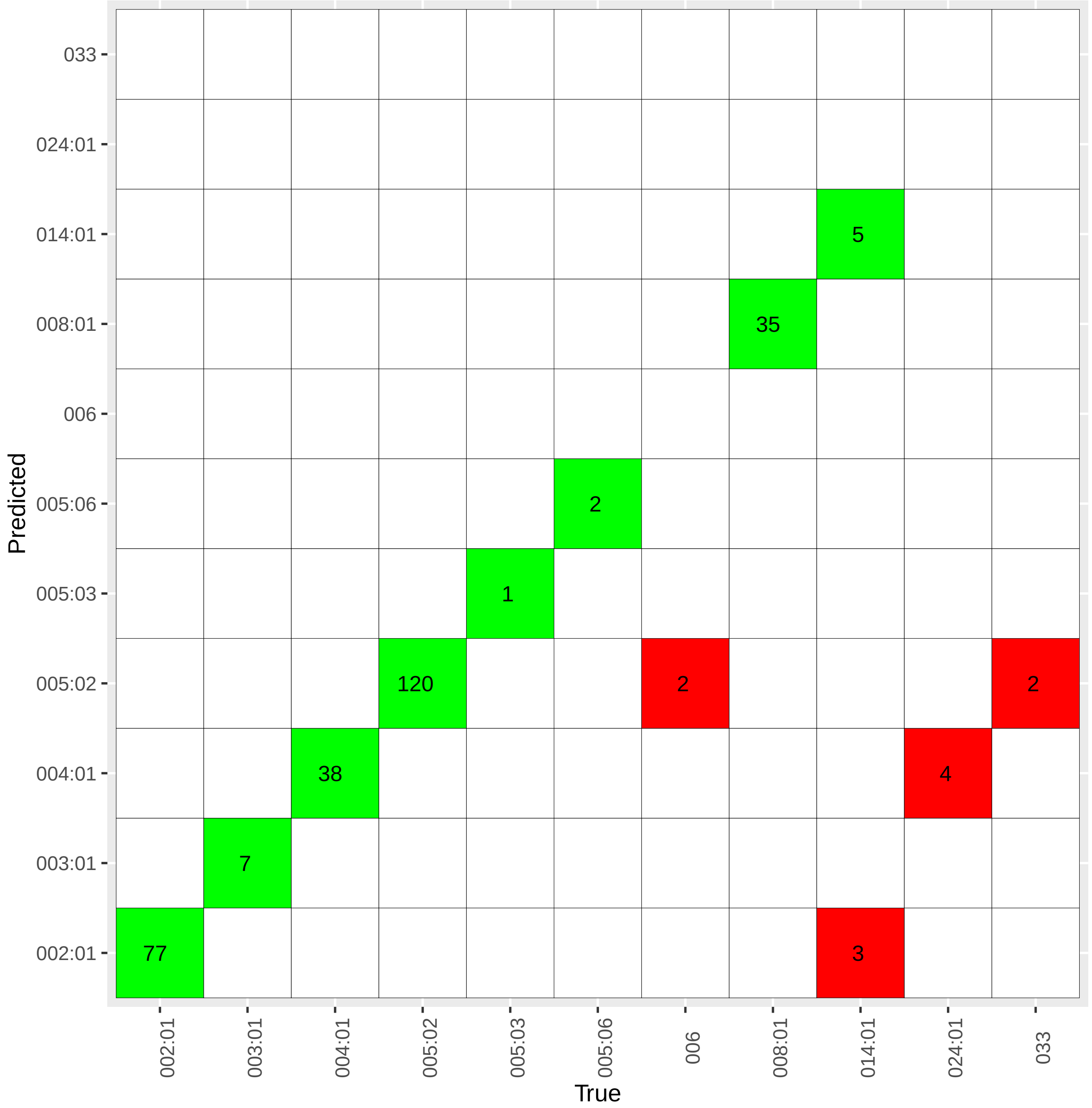

gene = MICB
model = iv
model limit = NULL
pop = EAS

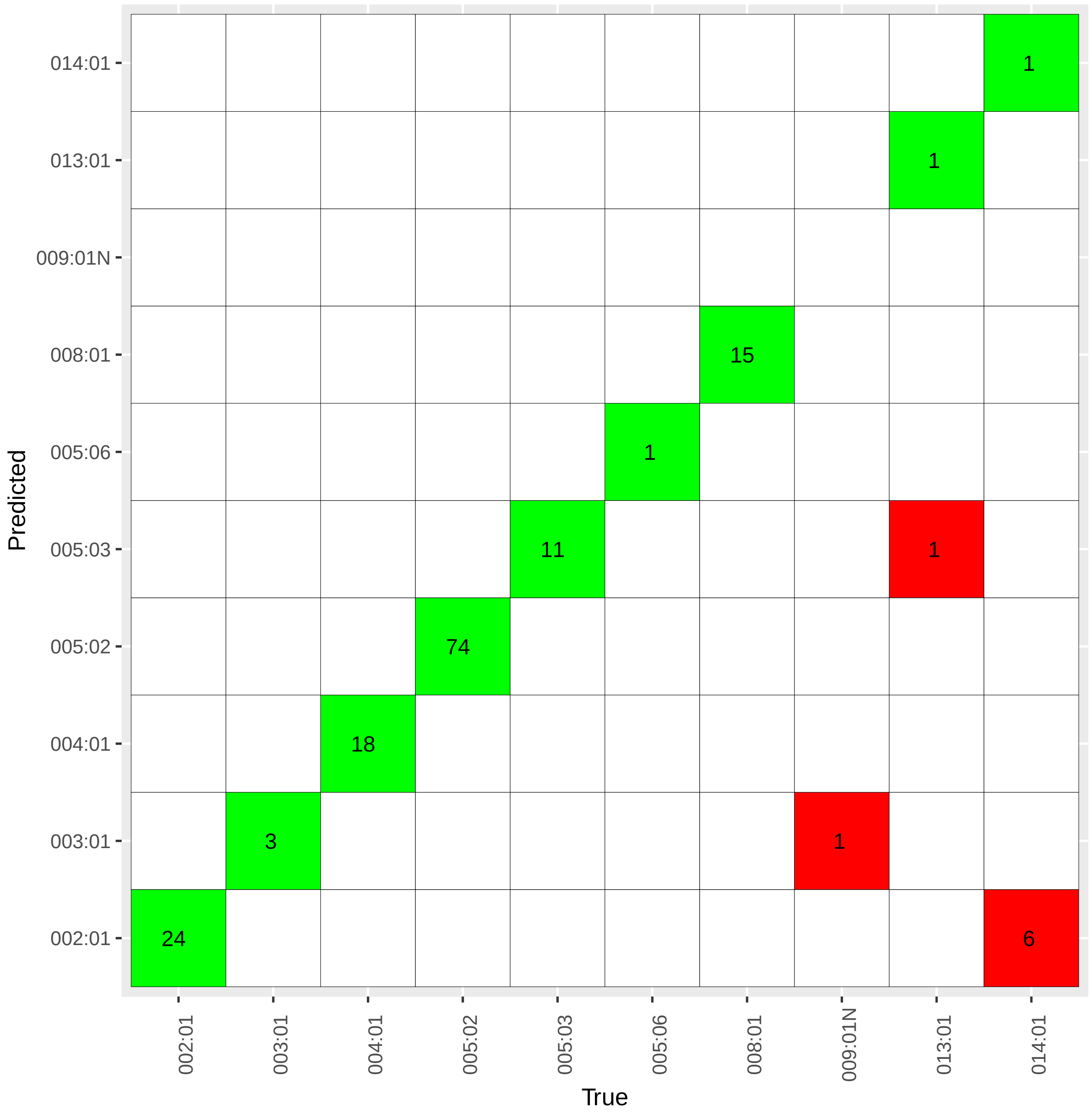

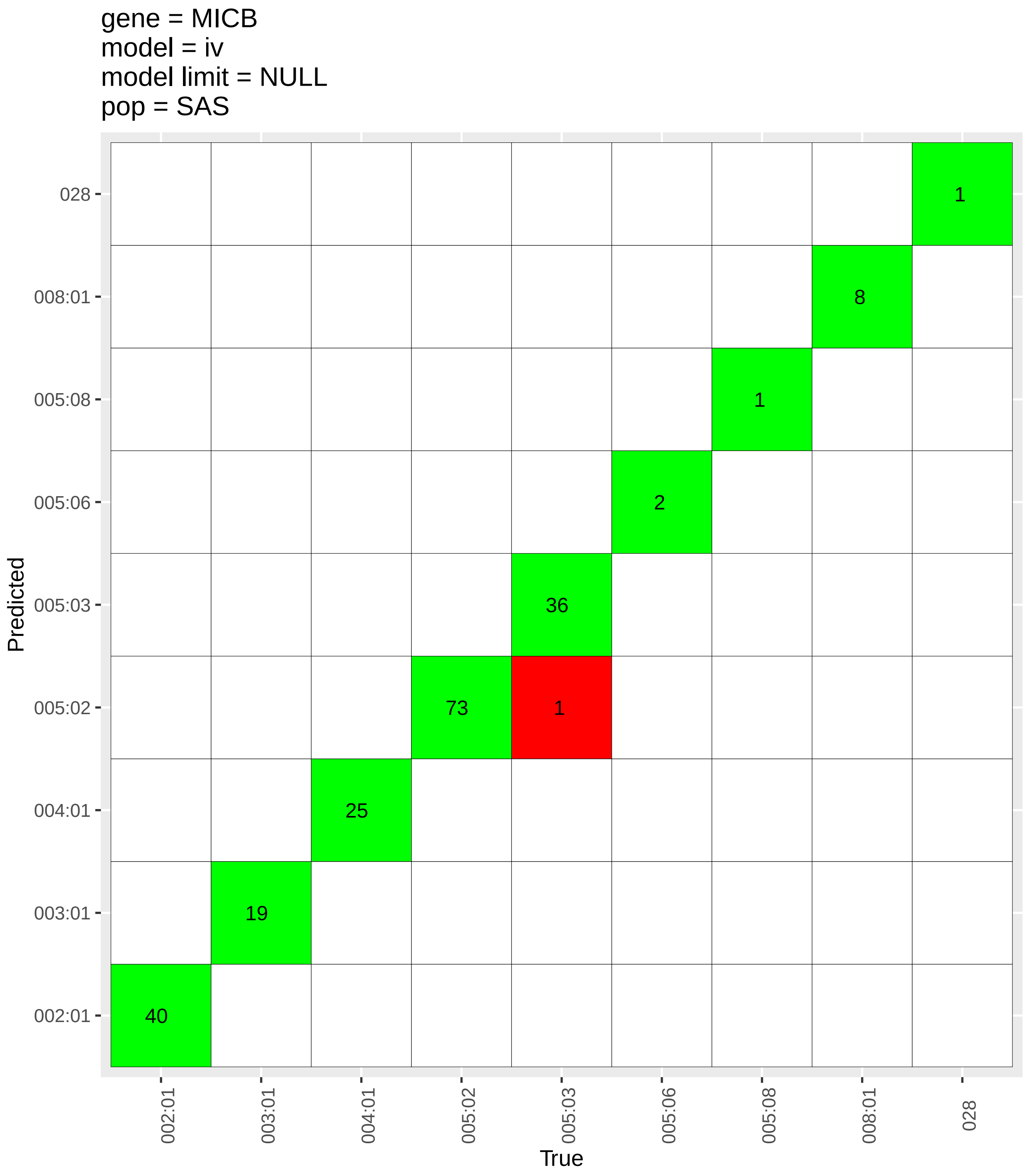
gene = MICB
model = iv
model limit = NULL
pop = SAS
Predicted
True
028
008:01
005:08
005:06
005:03
005:02
004:01
003:01
002:01
1
8
1
2
36
73
1
25
19
40

gene = MICB
model = iv
model limit = NULL
pop = AMR

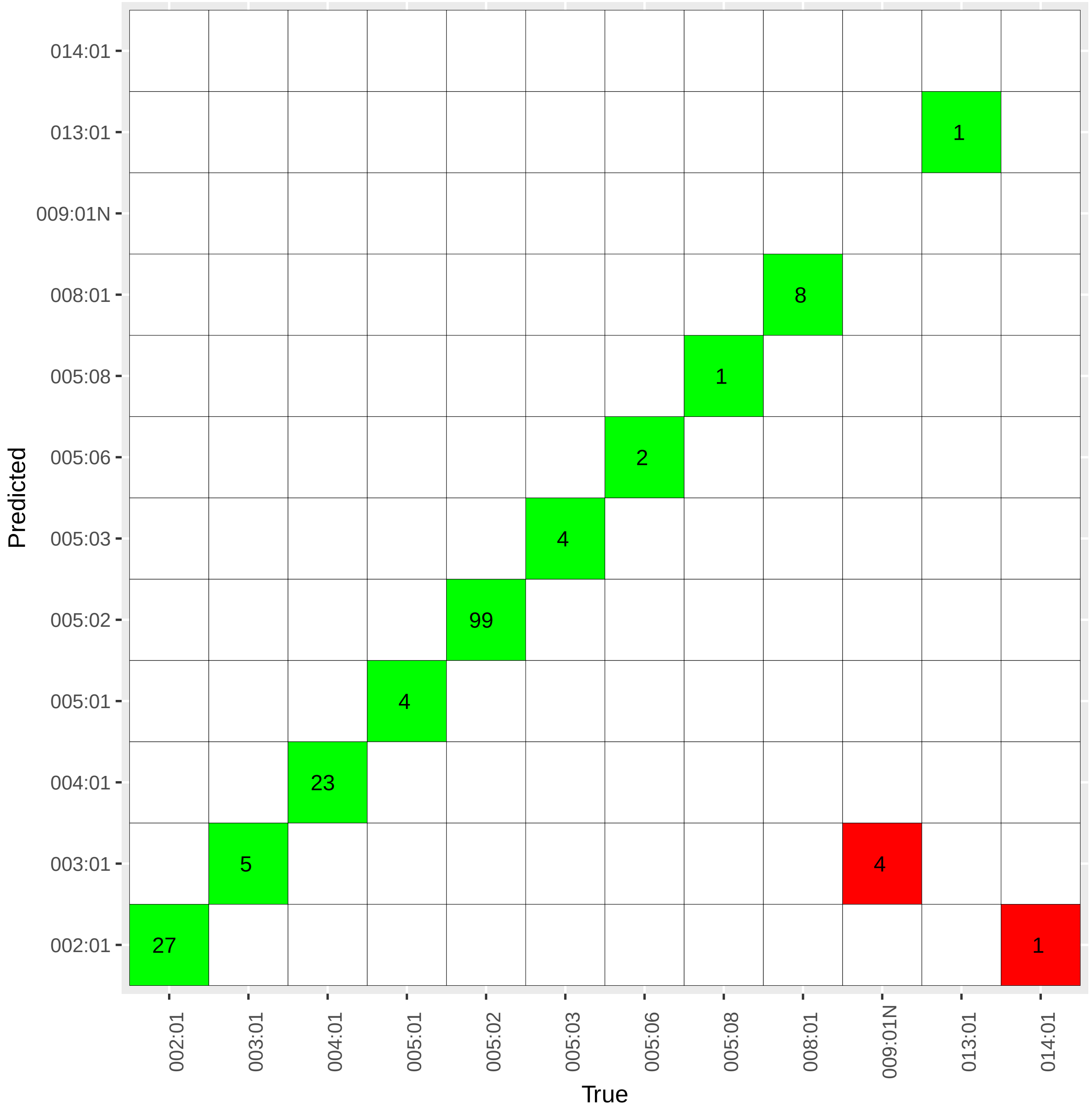

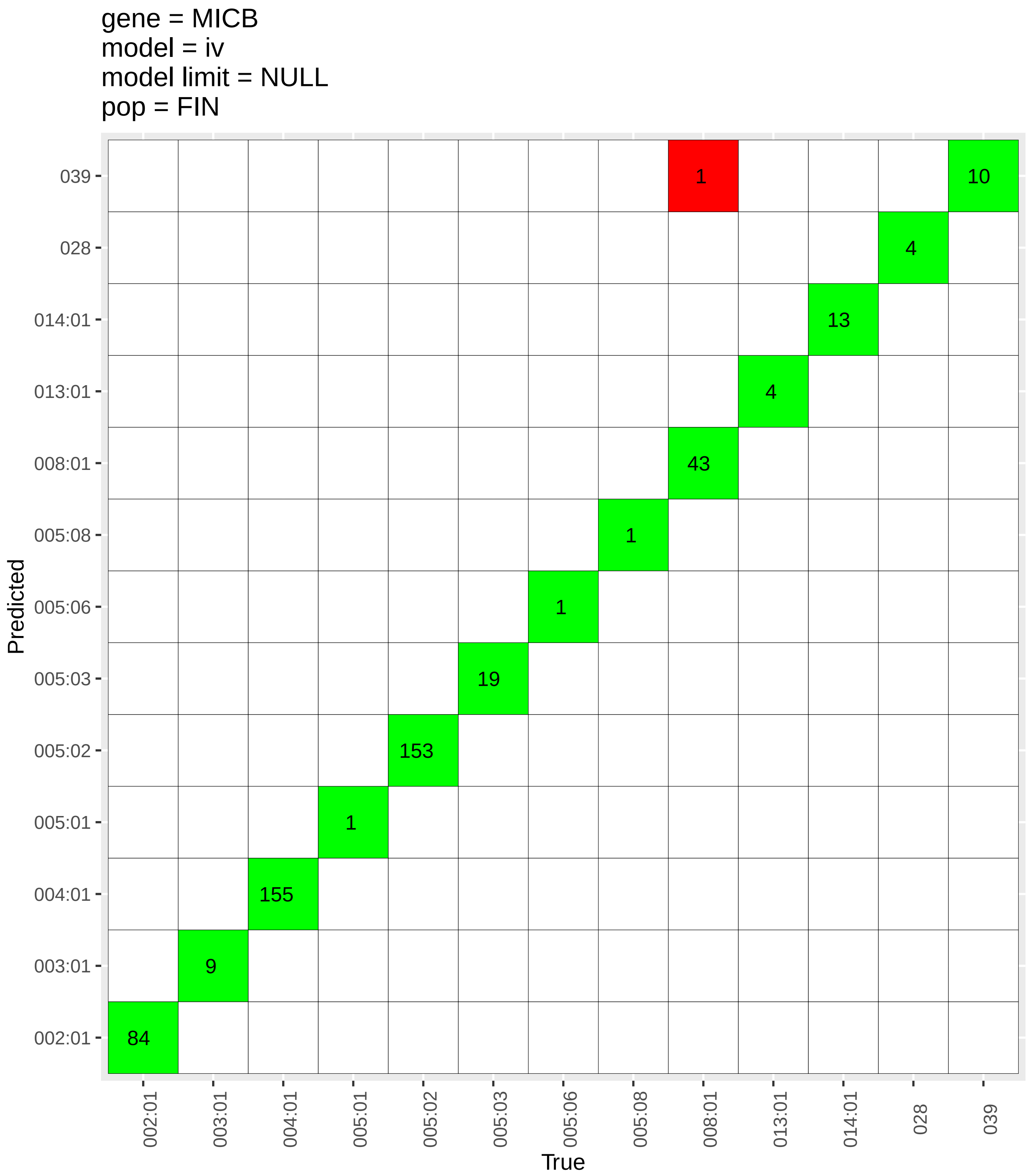
gene = MICB
model = iv
model limit = NULL
pop = FIN
Predicted
039
028
014:01
013:01
008:01
005:08
005:06
005:03
005:02
005:01
004:01
003:01
002:01
1
10
4
13
4
43
1
1
19
153
1
155
9
84
True
002:01
003:01
004:01
005:01
005:02
005:03
005:06
005:08
008:01
013:01
014:01
028
039

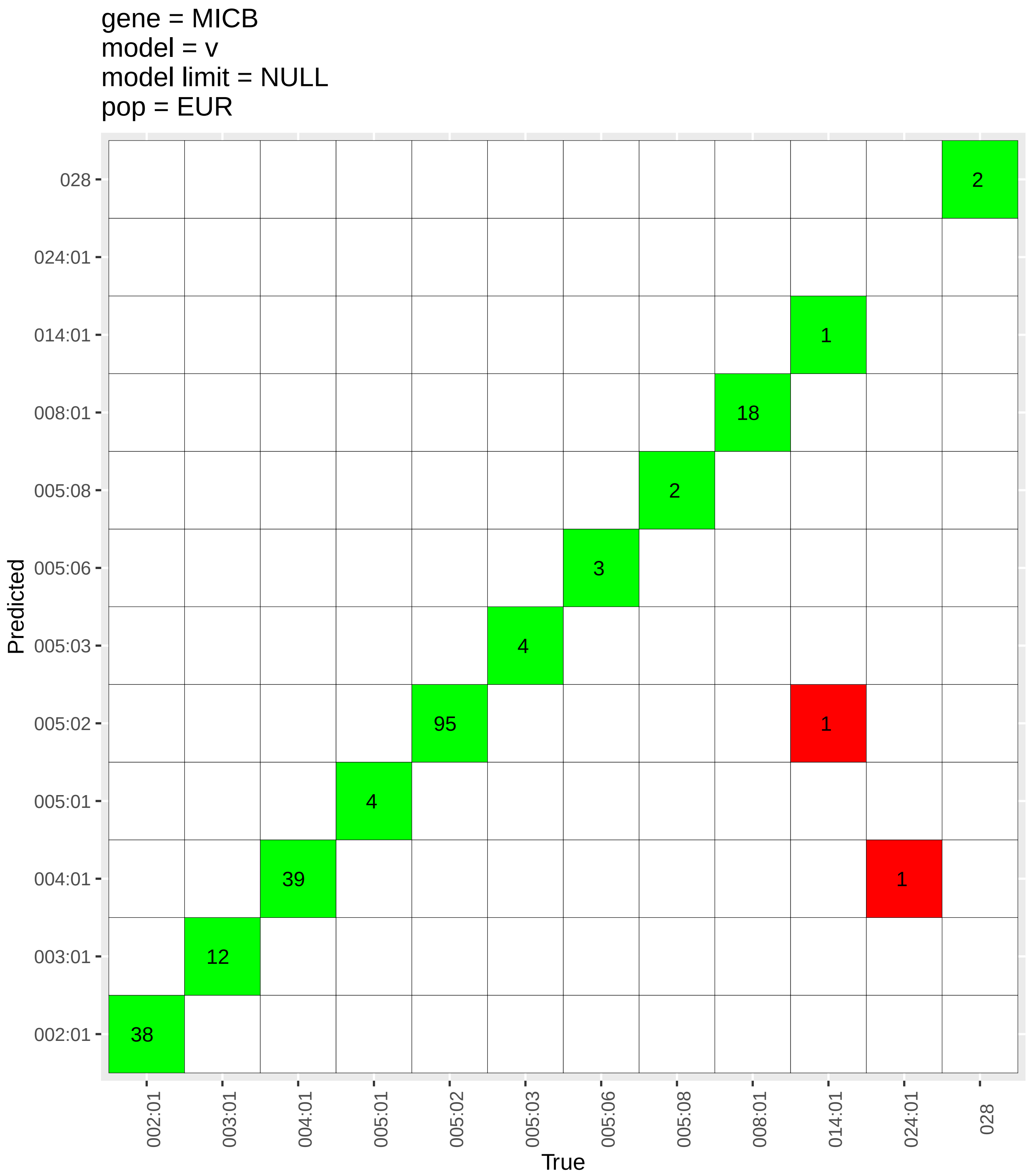
gene = MICB
model = v
model limit = NULL
pop = EUR
Predicted
True
028
024:01
014:01
008:01
005:08
005:06
005:03
005:02
005:01
004:01
003:01
002:01
38
12
39
4
95
4
3
2
18
1
1
1
2

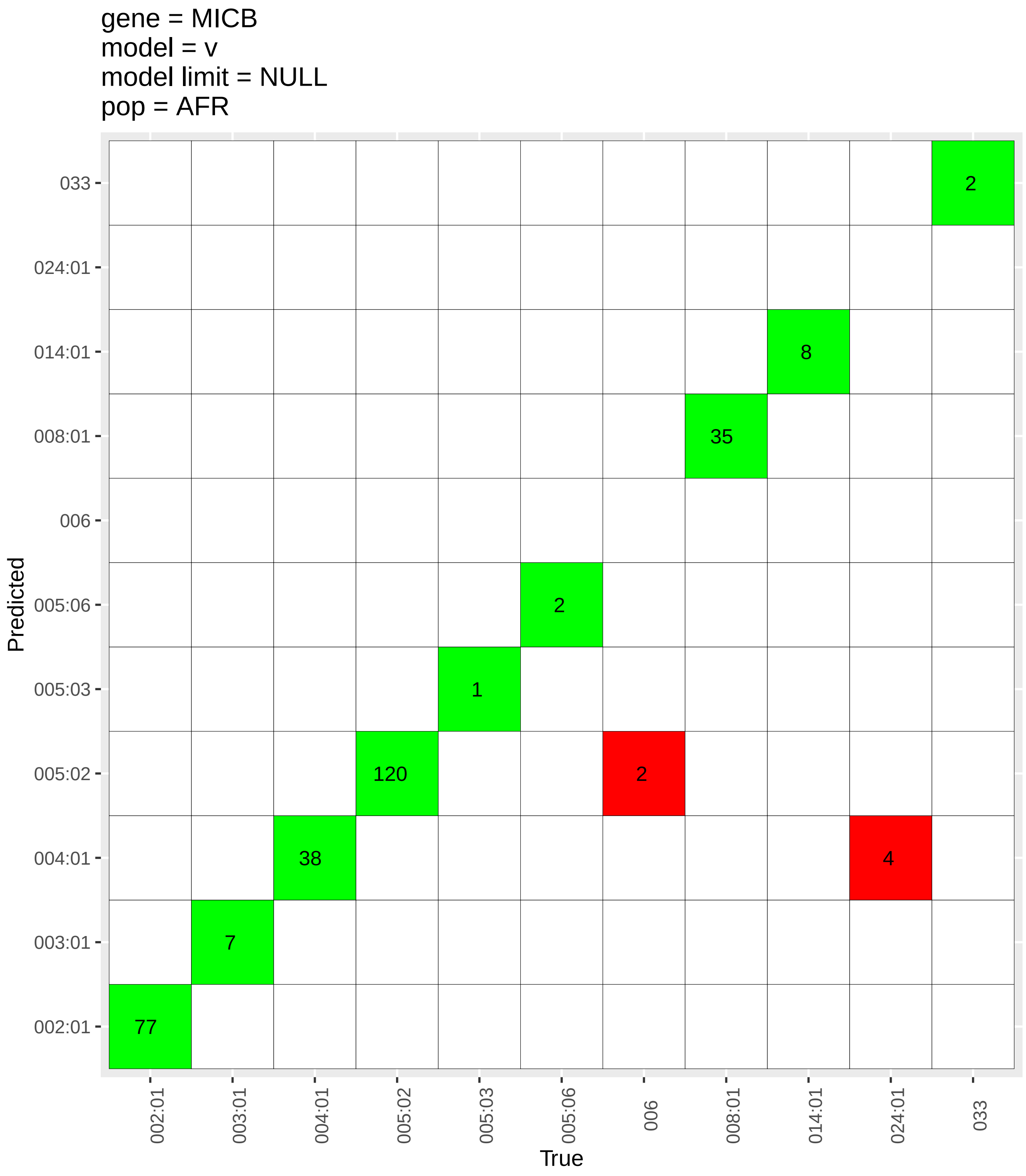

gene = MICB
model = v
model limit = NULL
pop = AFR
Predicted
True
033
024:01
014:01
008:01
006
005:06
005:03
005:02
004:01
003:01
002:01
2
8
35
2
1
120
2
38
4
7
77

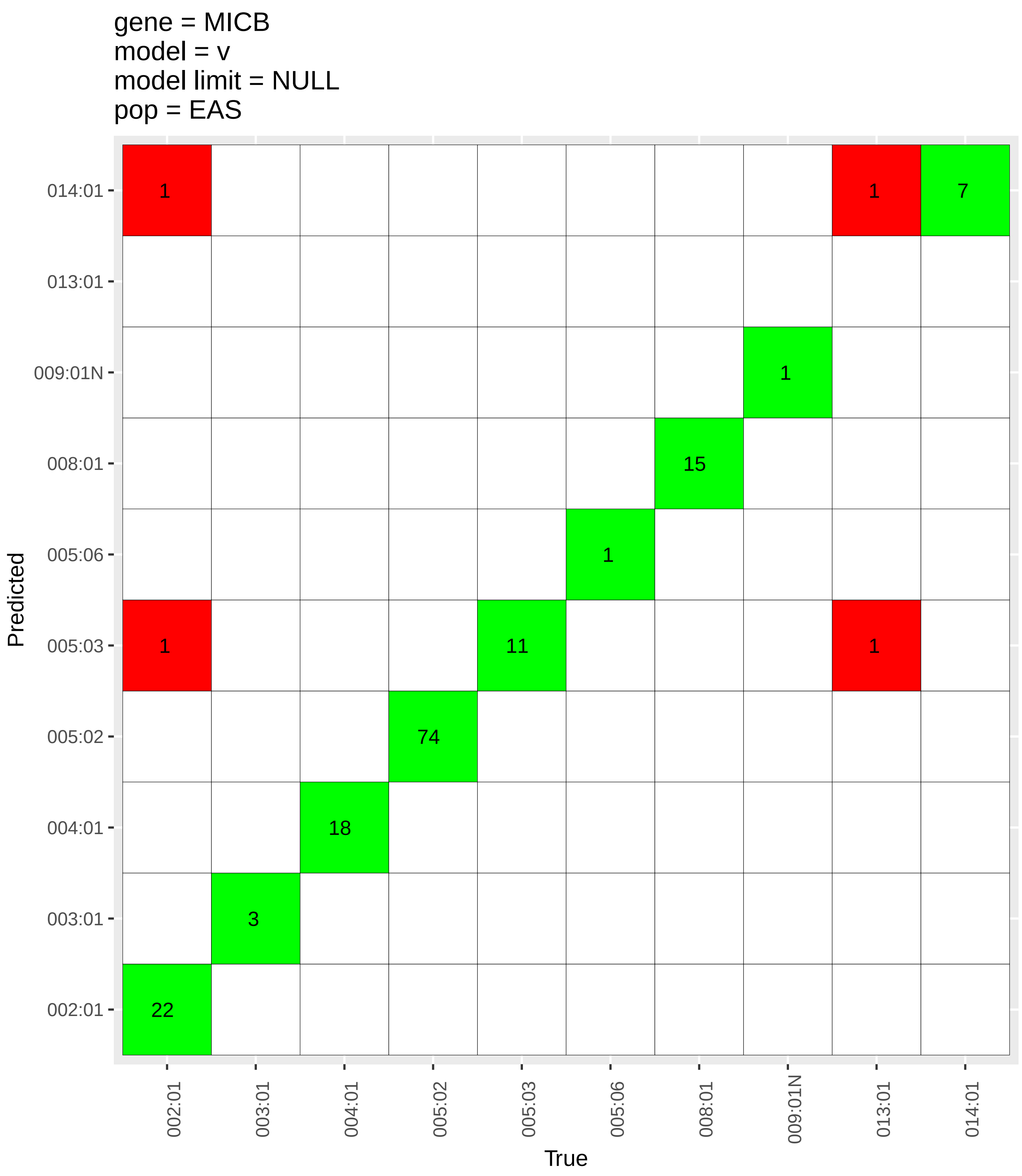
gene = MICB
model = v
model limit = NULL
pop = EAS
Predicted
True
014:01
013:01
009:01N
008:01
005:06
005:03
005:02
004:01
003:01
002:01
1
1
7
1
15
1
1
11
1
74
18
3
22

gene = MICB
model = v
model limit = NULL
pop = SAS

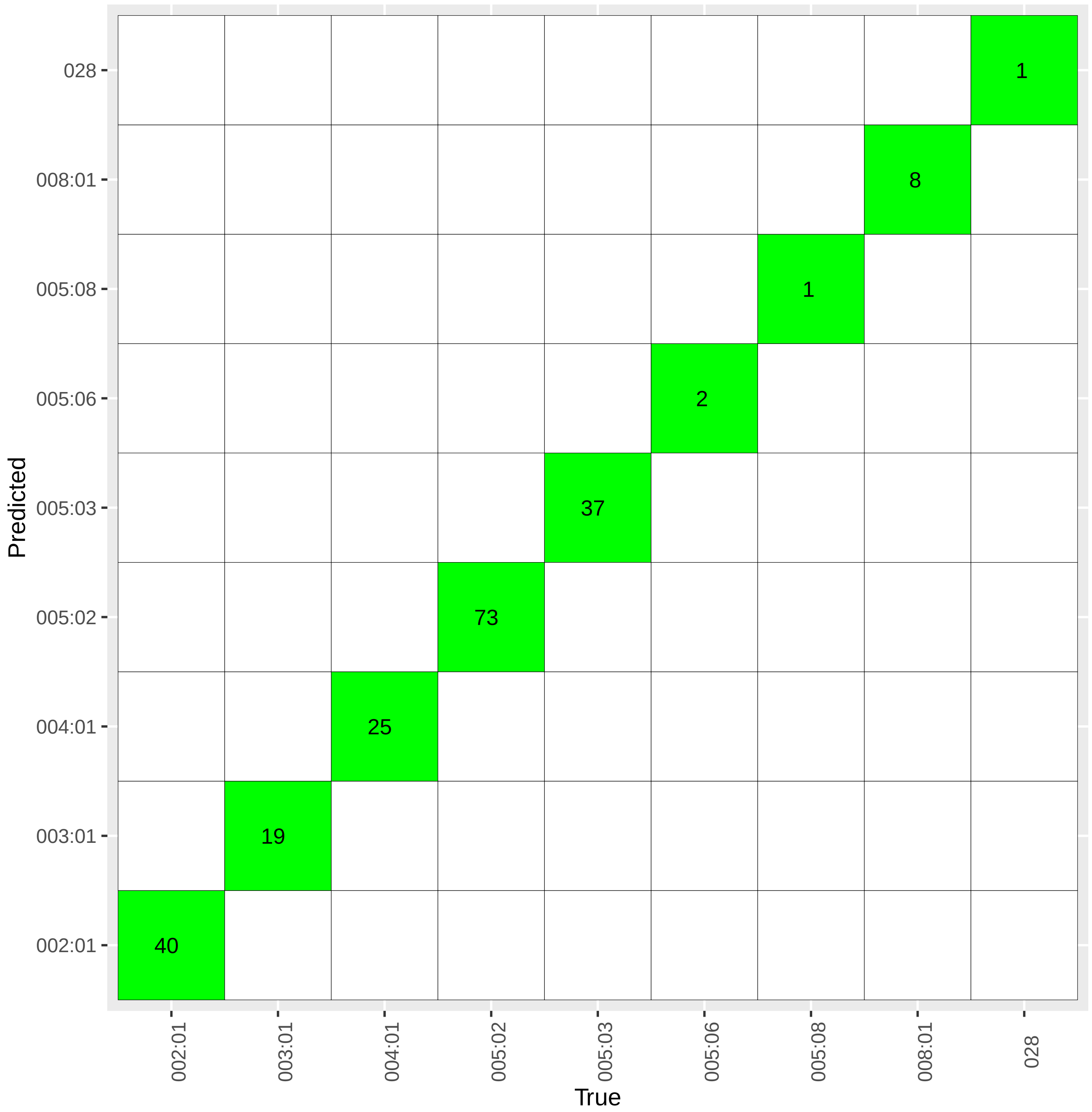

gene = MICB
model = v
model limit = NULL
pop = AMR

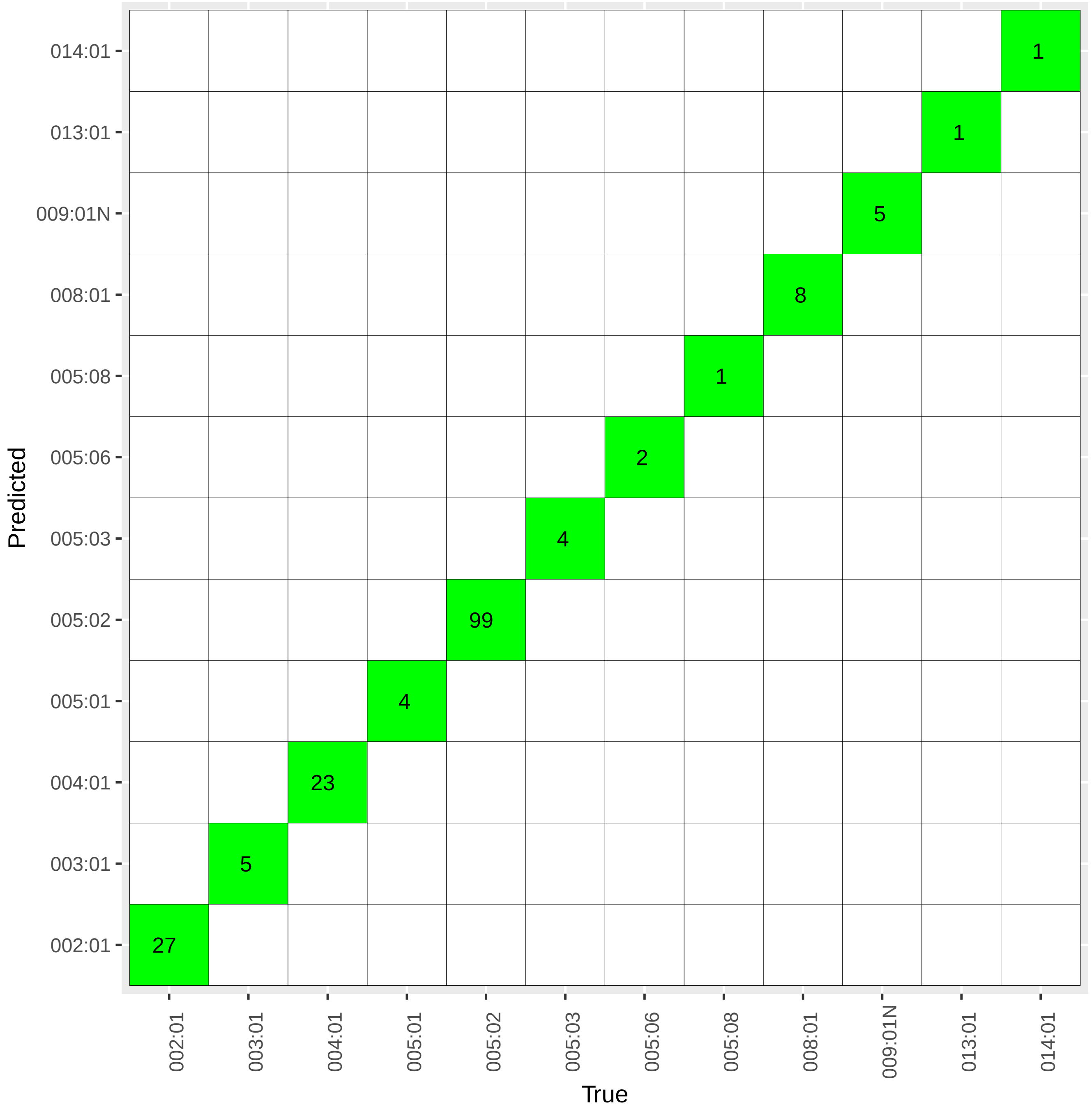

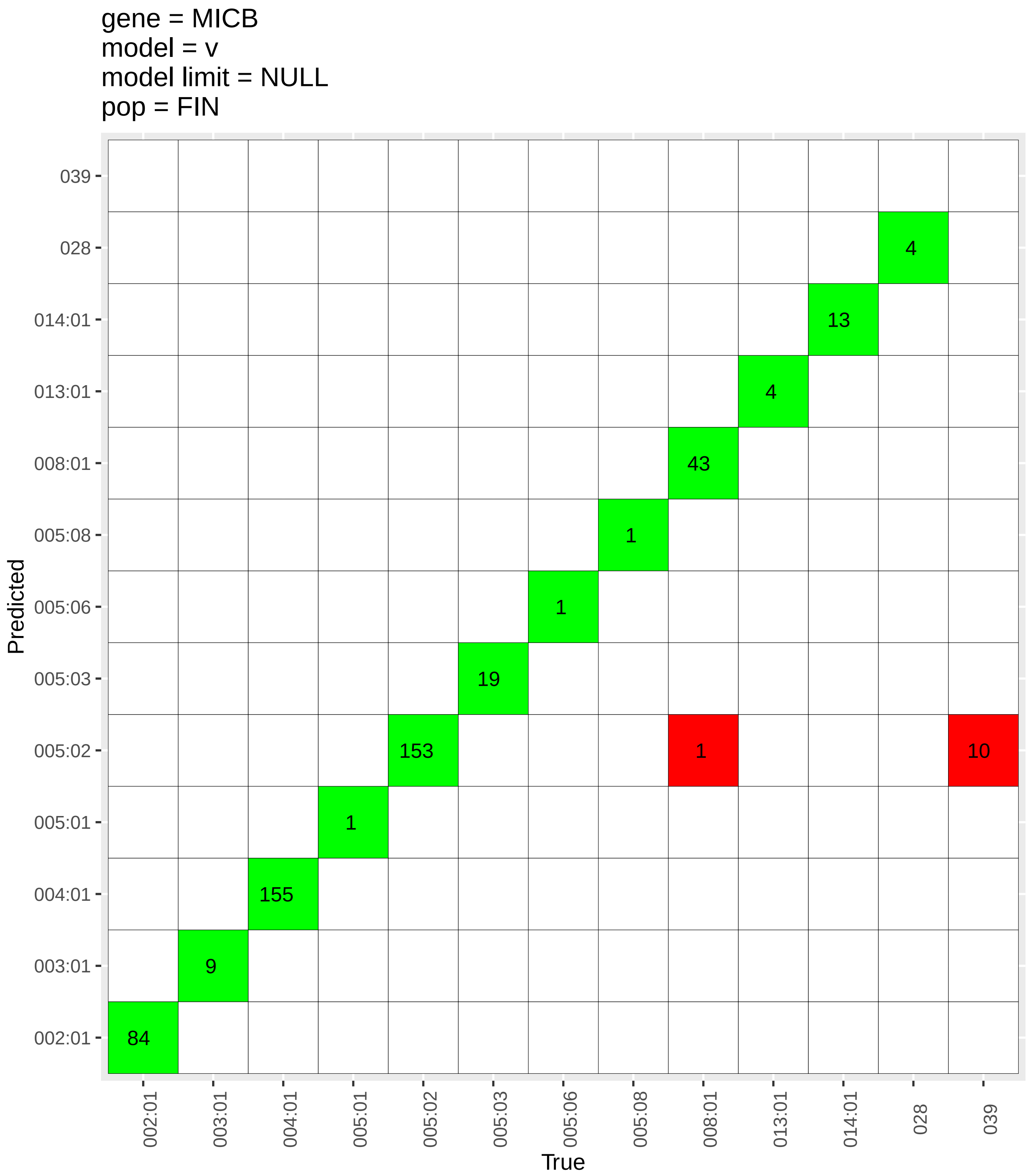
gene = MICB
model = v
model limit = NULL
pop = FIN
Predicted
039
028
014:01
013:01
008:01
005:08
005:06
005:03
005:02
005:01
004:01
003:01
002:01
True
002:01
003:01
004:01
005:01
005:02
005:03
005:06
005:08
008:01
013:01
014:01
028
039
84
9
155
1
153
19
1
1
43
4
13
4
1
10

gene = MICB
model = vi
model limit = NULL
pop = EUR

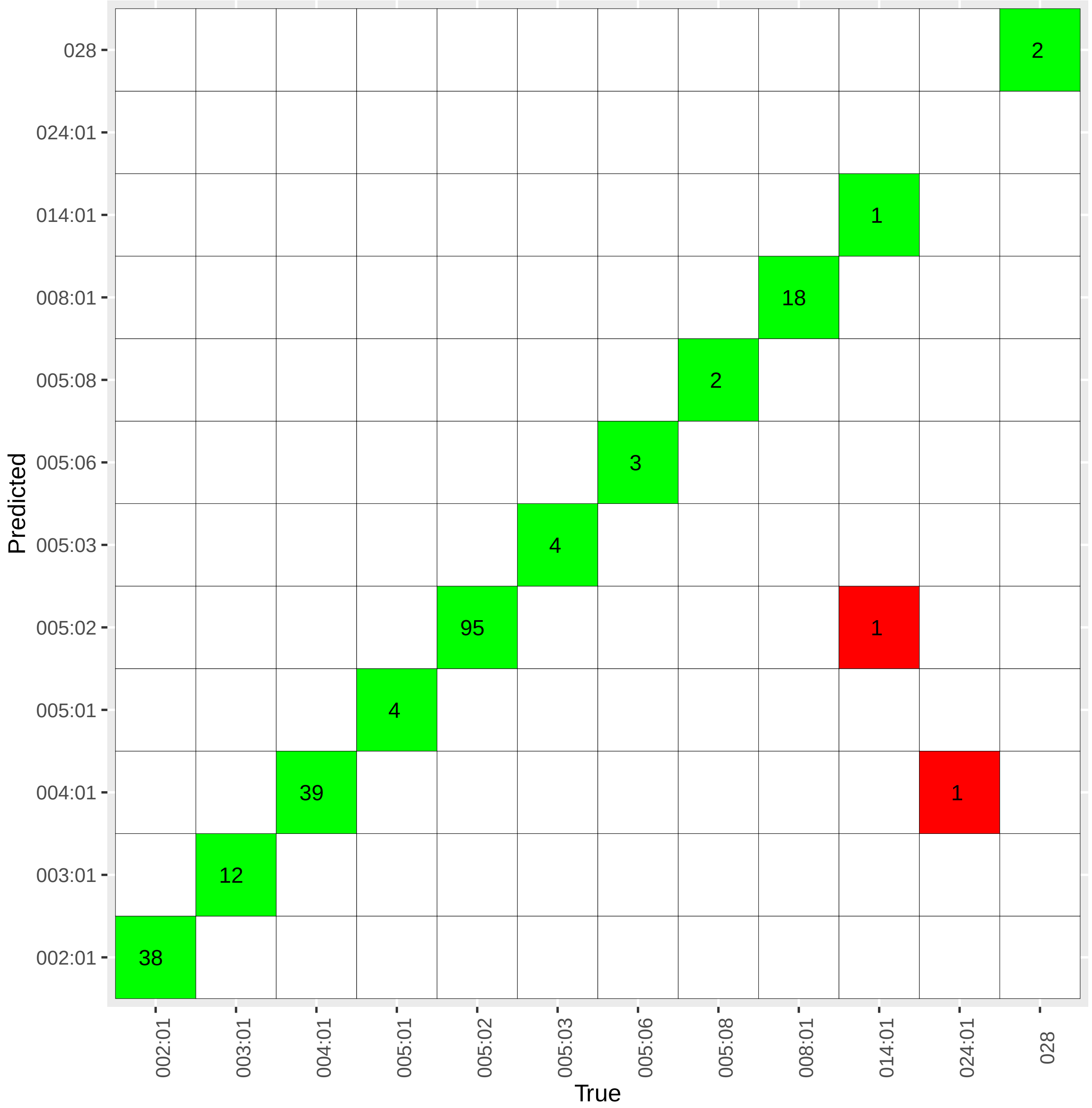

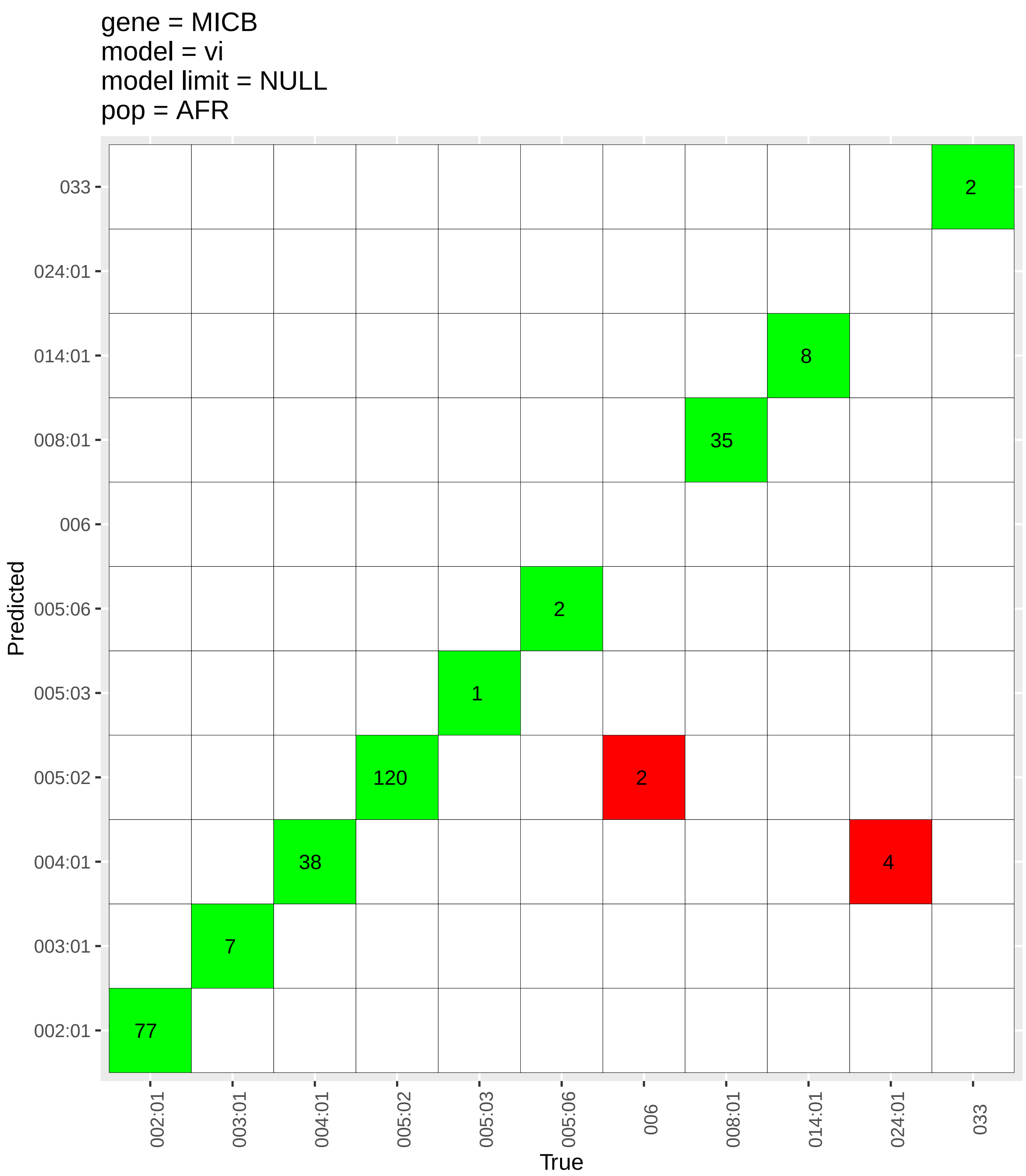
gene = MICB
model = vi
model limit = NULL
pop = AFR
Predicted
True
033
024:01
014:01
008:01
006
005:06
005:03
005:02
004:01
003:01
002:01
2
8
35
2
1
120
2
38
4
7
77

gene = MICB
model = vi
model limit = NULL
pop = EAS

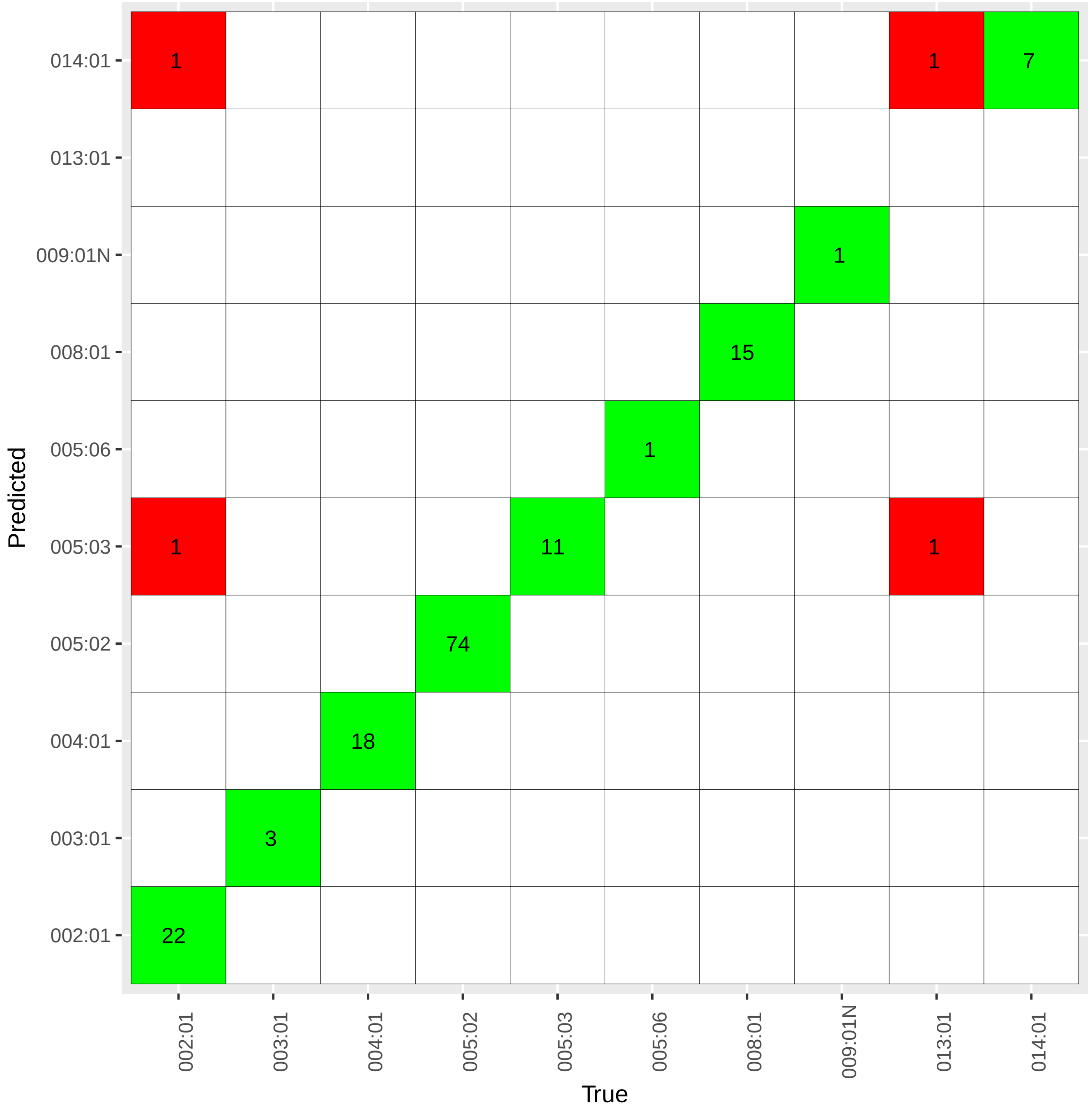

gene = MICB
model = vi
model limit = NULL
pop = SAS

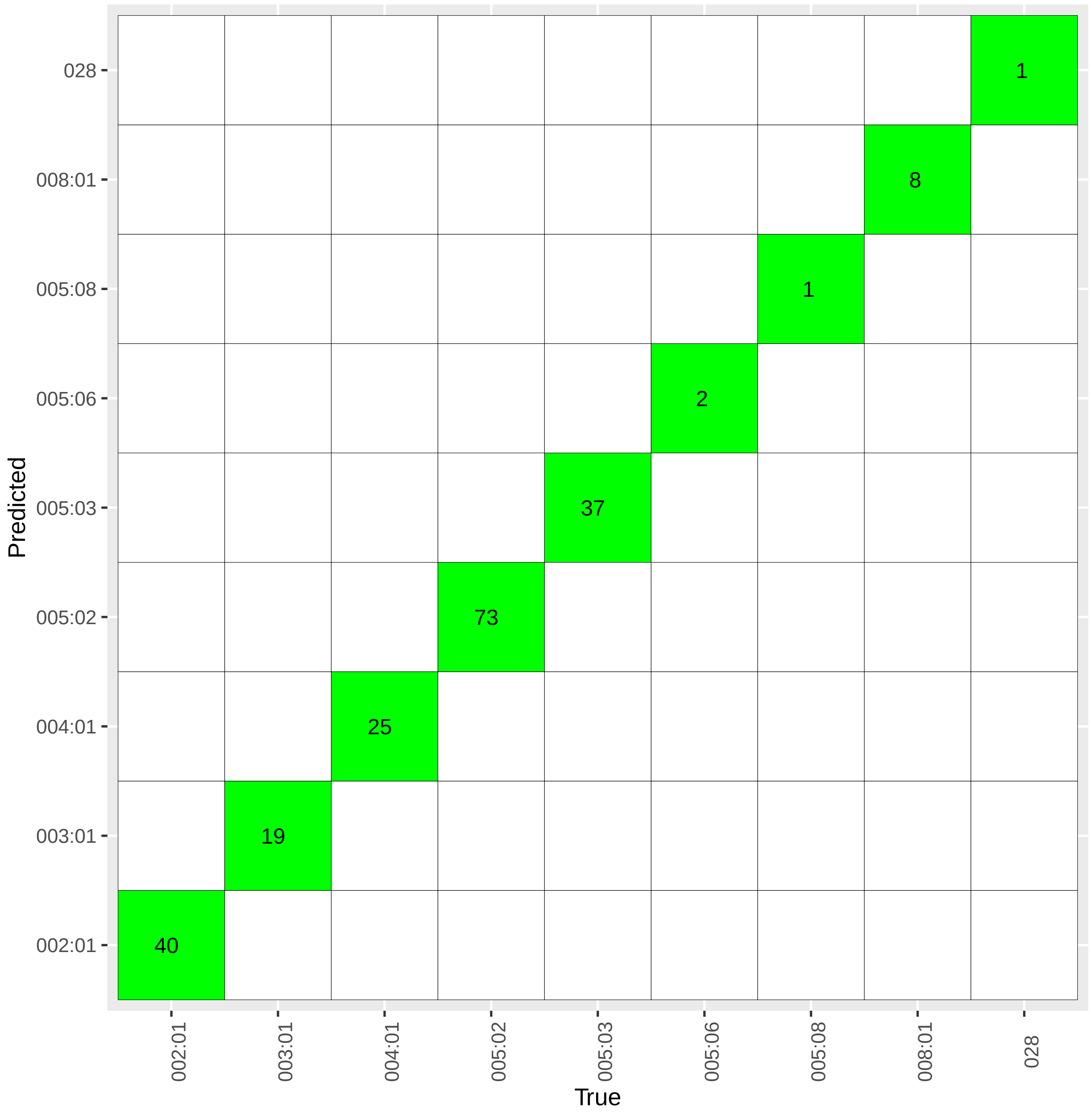

gene = MICB
model = vi
model limit = NULL
pop = AMR

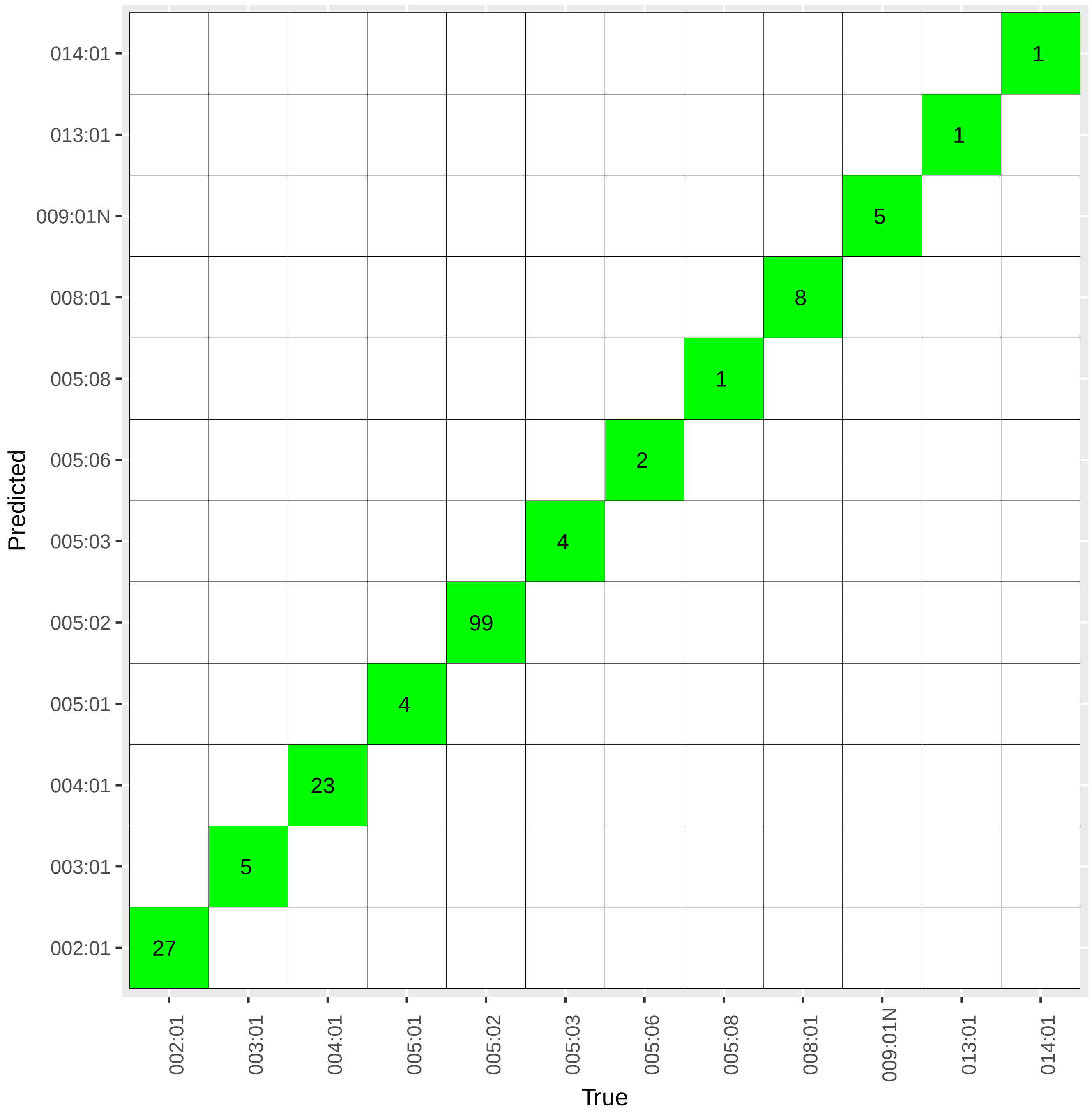

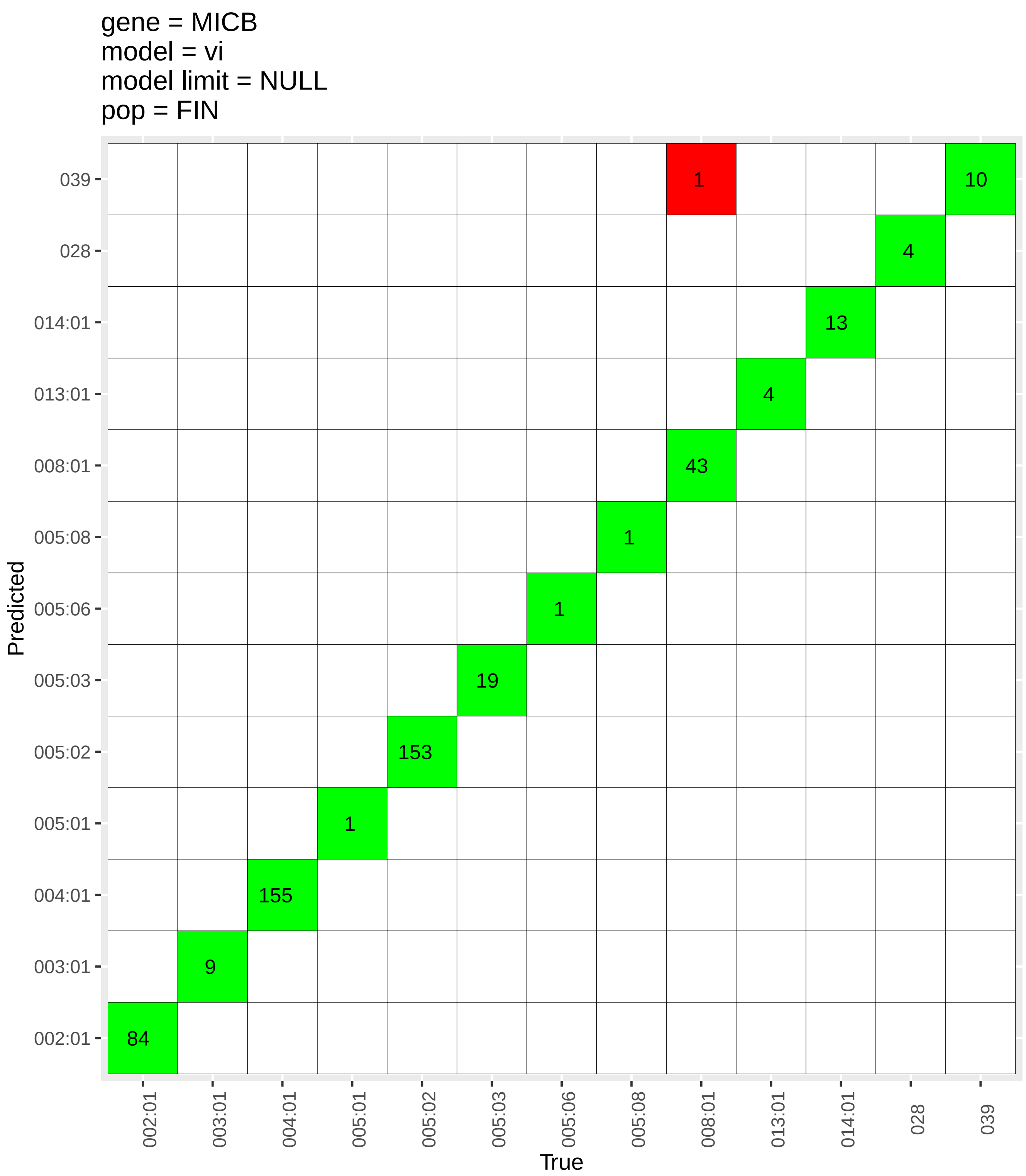
gene = MICB
model = vi
model limit = NULL
pop = FIN
Predicted
039
028
014:01
013:01
008:01
005:08
005:06
005:03
005:02
005:01
004:01
003:01
002:01
1
10
4
13
4
43
1
1
19
153
1
155
9
84
002:01
003:01
004:01
005:01
005:02
005:03
005:06
005:08
008:01
013:01
014:01
028
039
True

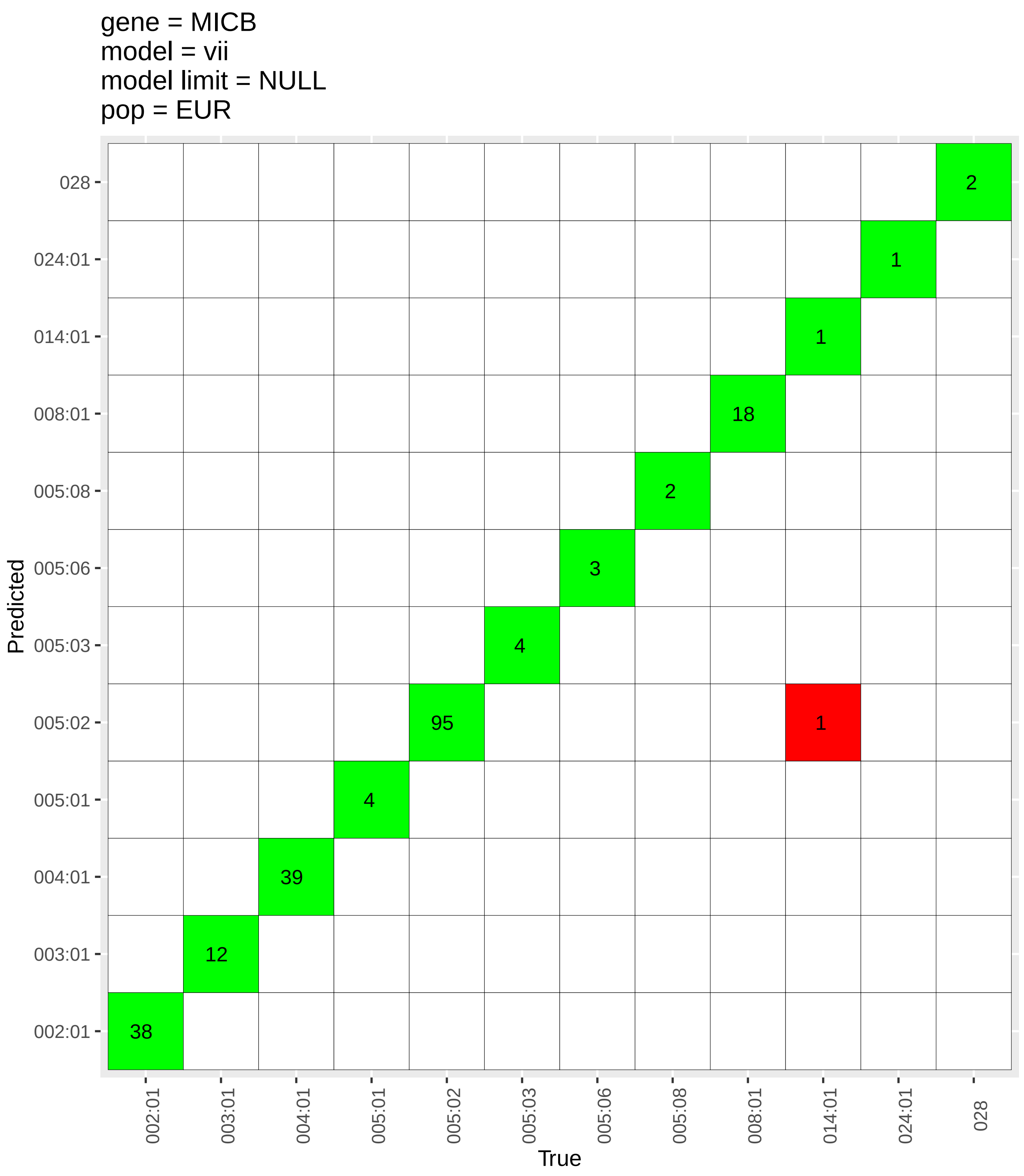
gene = MICB
model = vii
model limit = NULL
pop = EUR
Predicted
028
024:01
014:01
008:01
005:08
005:06
005:03
005:02
005:01
004:01
003:01
002:01
True
002:01
003:01
004:01
005:01
005:02
005:03
005:06
005:08
008:01
014:01
024:01
028
38
12
39
4
95
4
3
2
18
1
1
1
2

gene = MICB
model = vii
model limit = NULL
pop = AFR

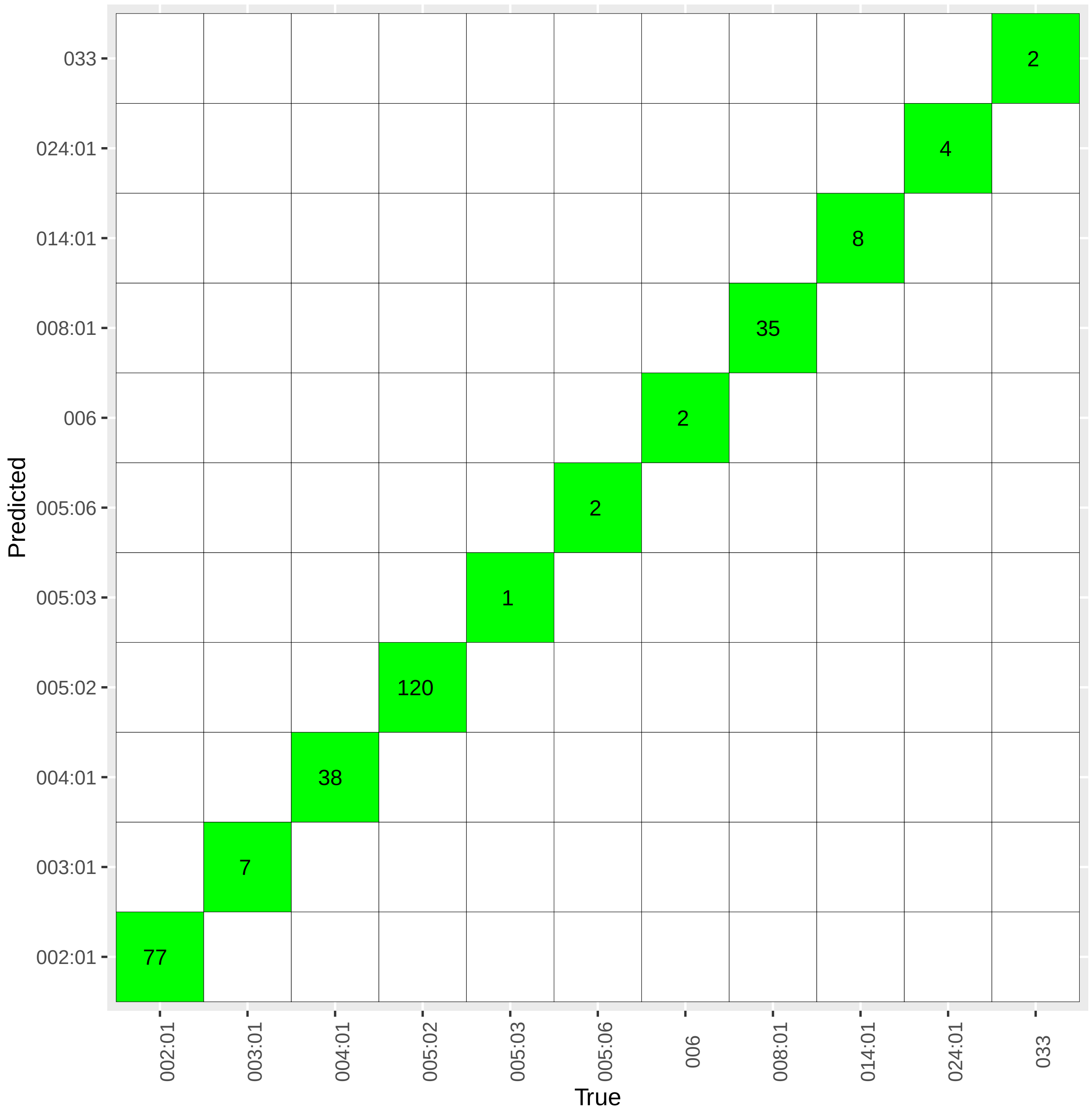

gene = MICB
model = vii
model limit = NULL
pop = EAS

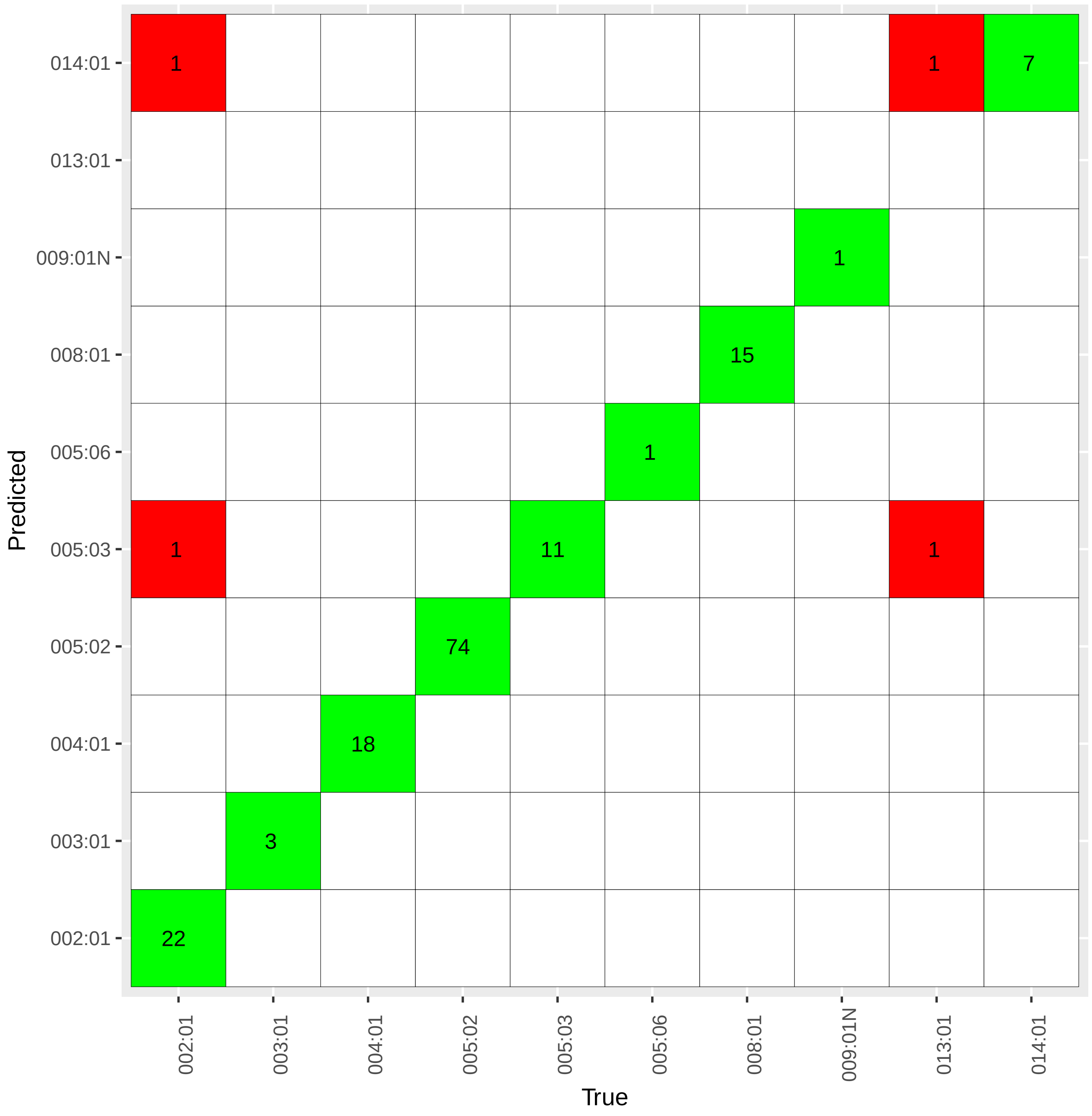

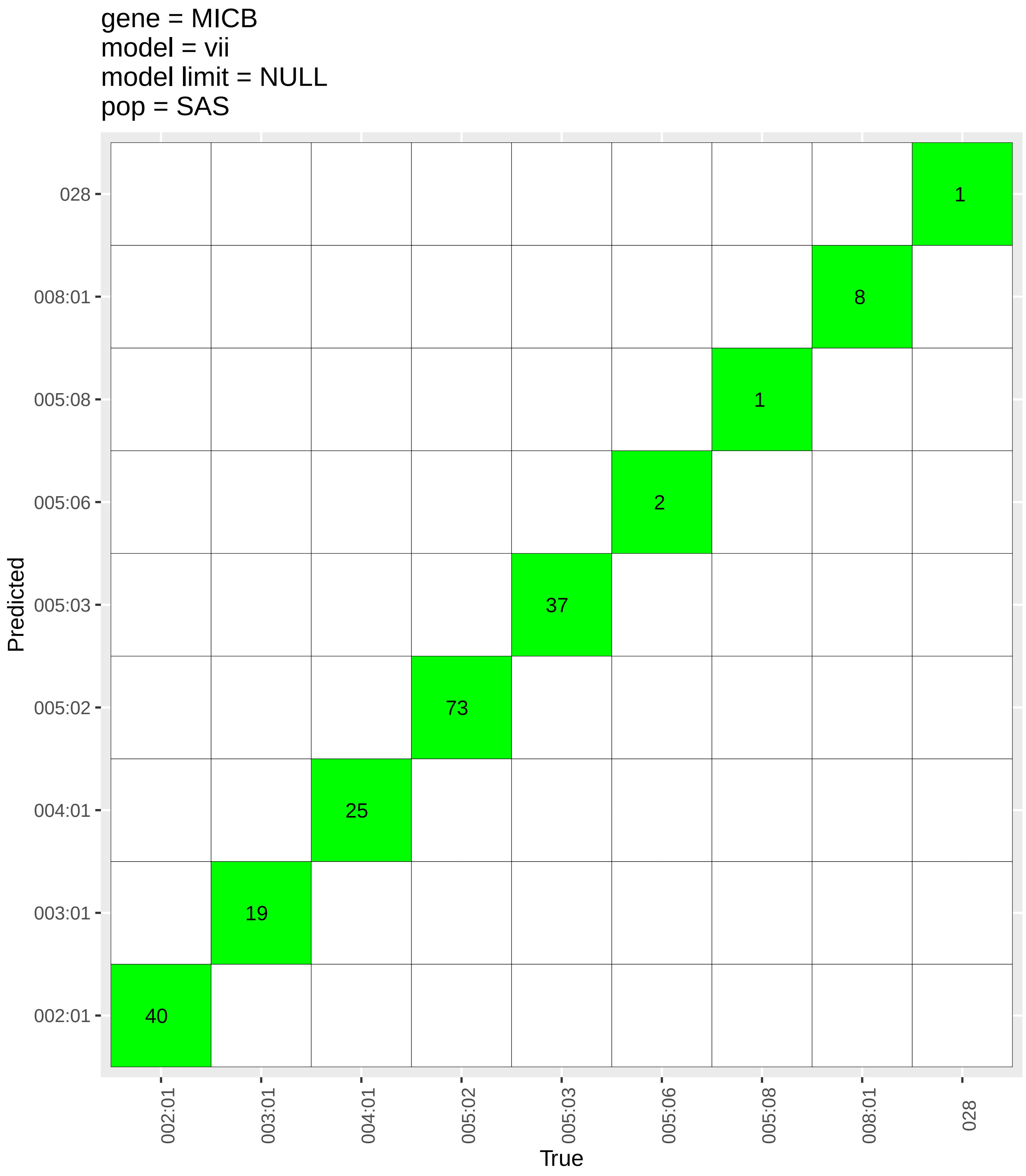
gene = MICB
model = vii
model limit = NULL
pop = SAS
Predicted
028
008:01
005:08
005:06
005:03
005:02
004:01
003:01
002:01
1
8
1
2
37
73
25
19
40
002:01
003:01
004:01
005:02
005:03
005:06
005:08
008:01
028
True

gene = MICB
model = vii
model limit = NULL
pop = AMR

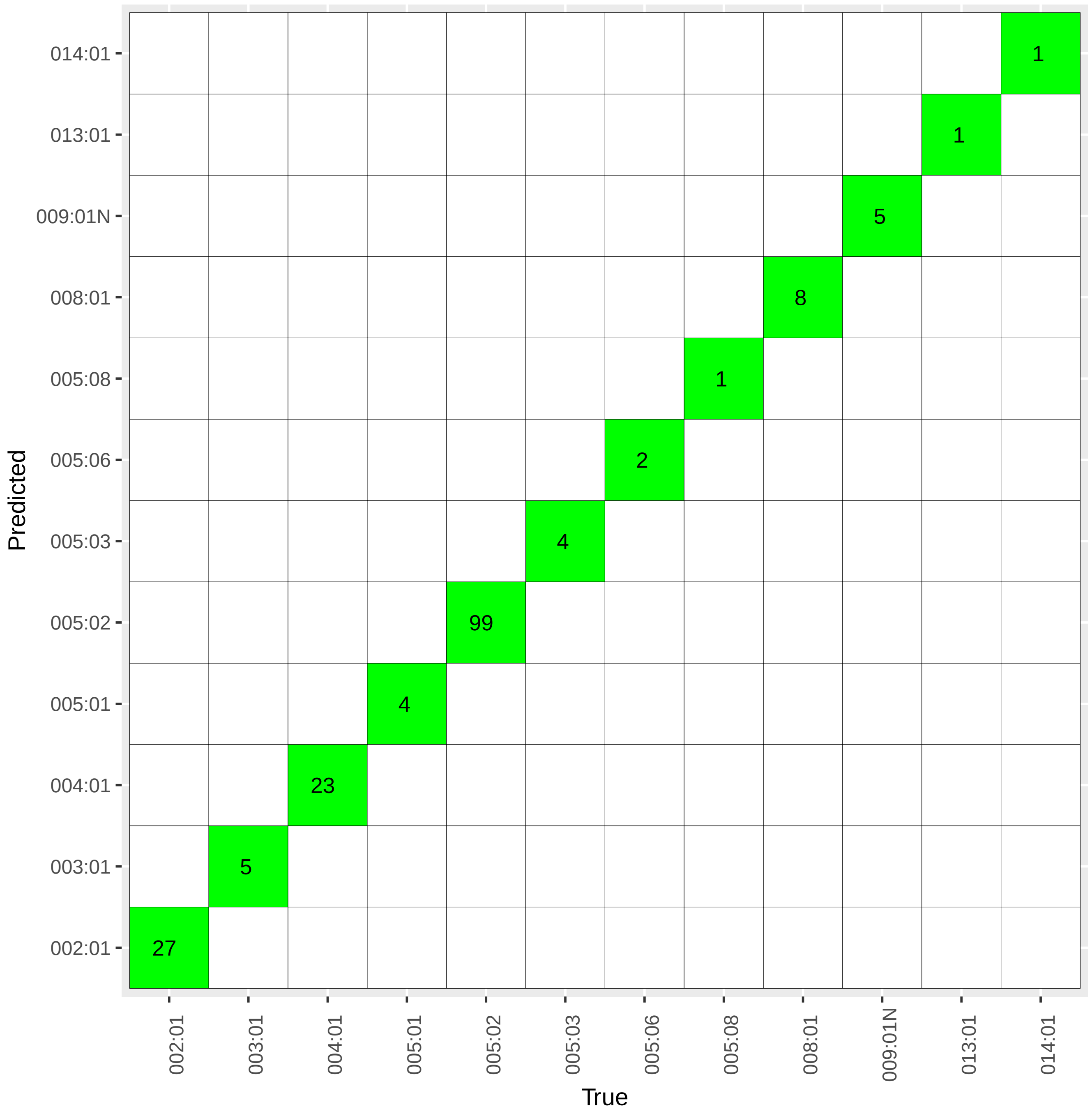

gene = MICB
model = vii
model limit = NULL
pop = FIN

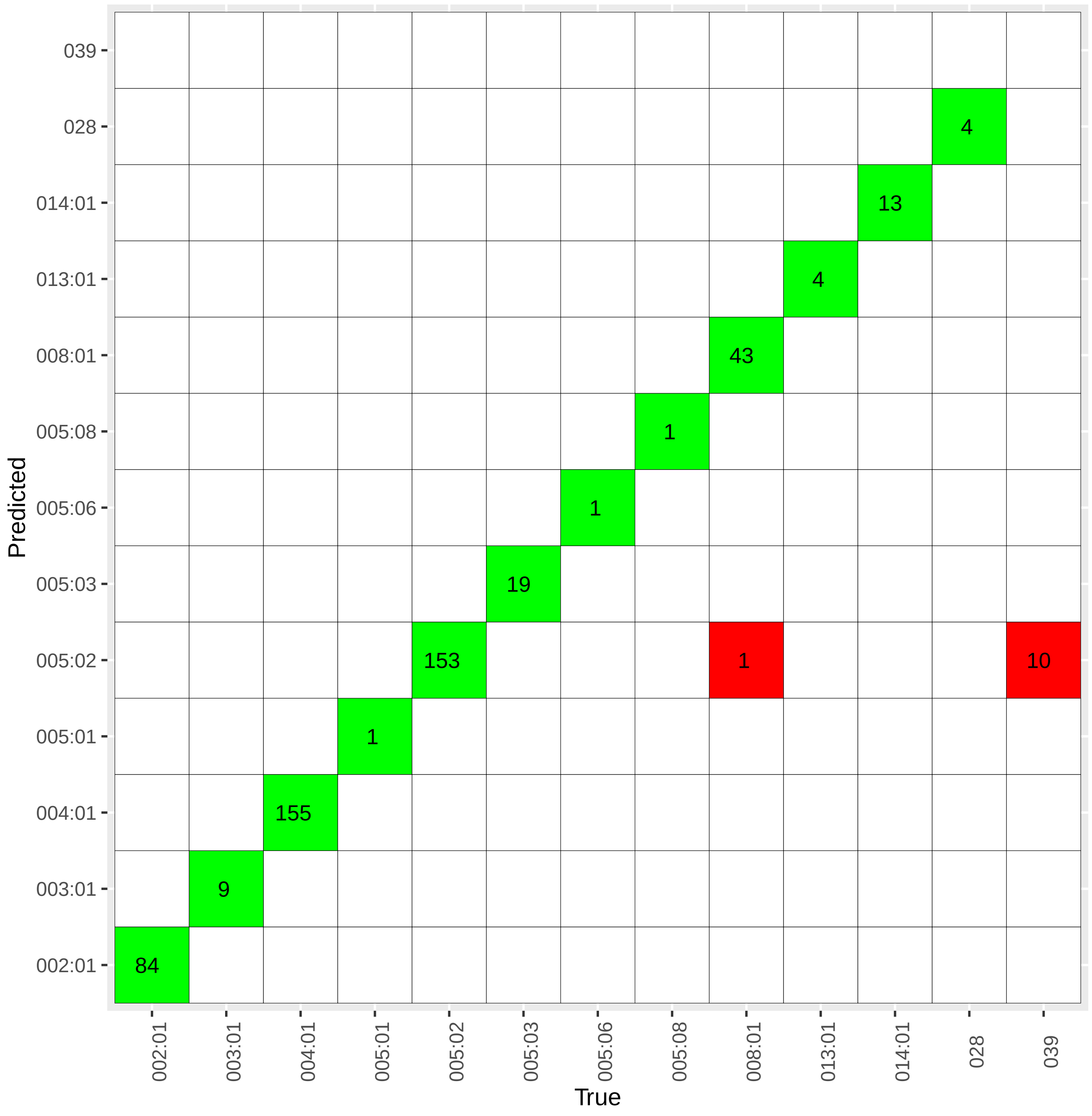

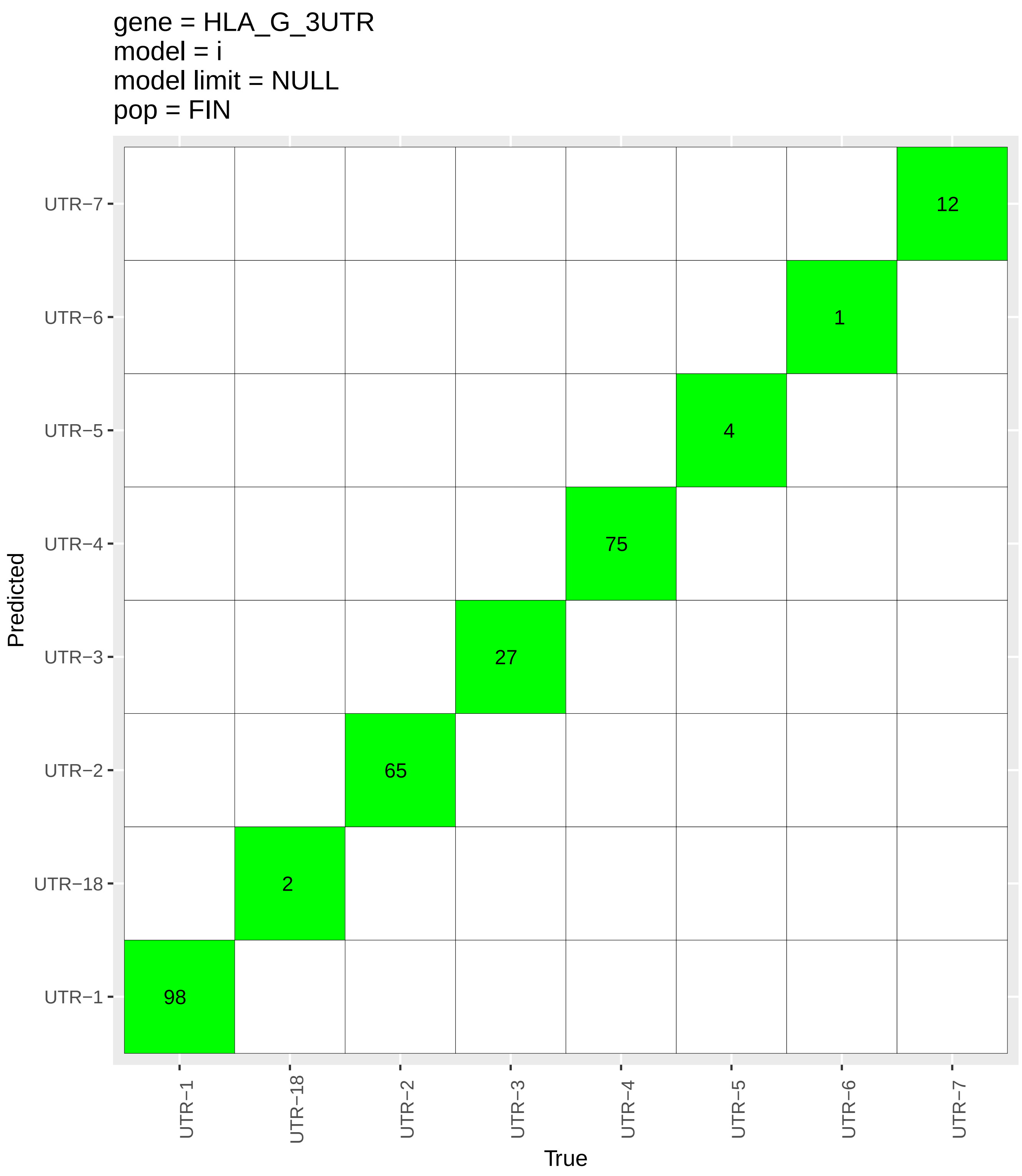
gene = HLA_G_3UTR
model = i
model limit = NULL
pop = FIN
Predicted
UTR−7
UTR−6
UTR−5
UTR−4
UTR−3
UTR−2
UTR−18
UTR−1
12
1
4
75
27
65
2
98
UTR−1
UTR−18
UTR−2
UTR−3
UTR−4
UTR−5
UTR−6
UTR−7
True

gene = HLA_G_5UTR
model = i
model limit = NULL
pop = FIN

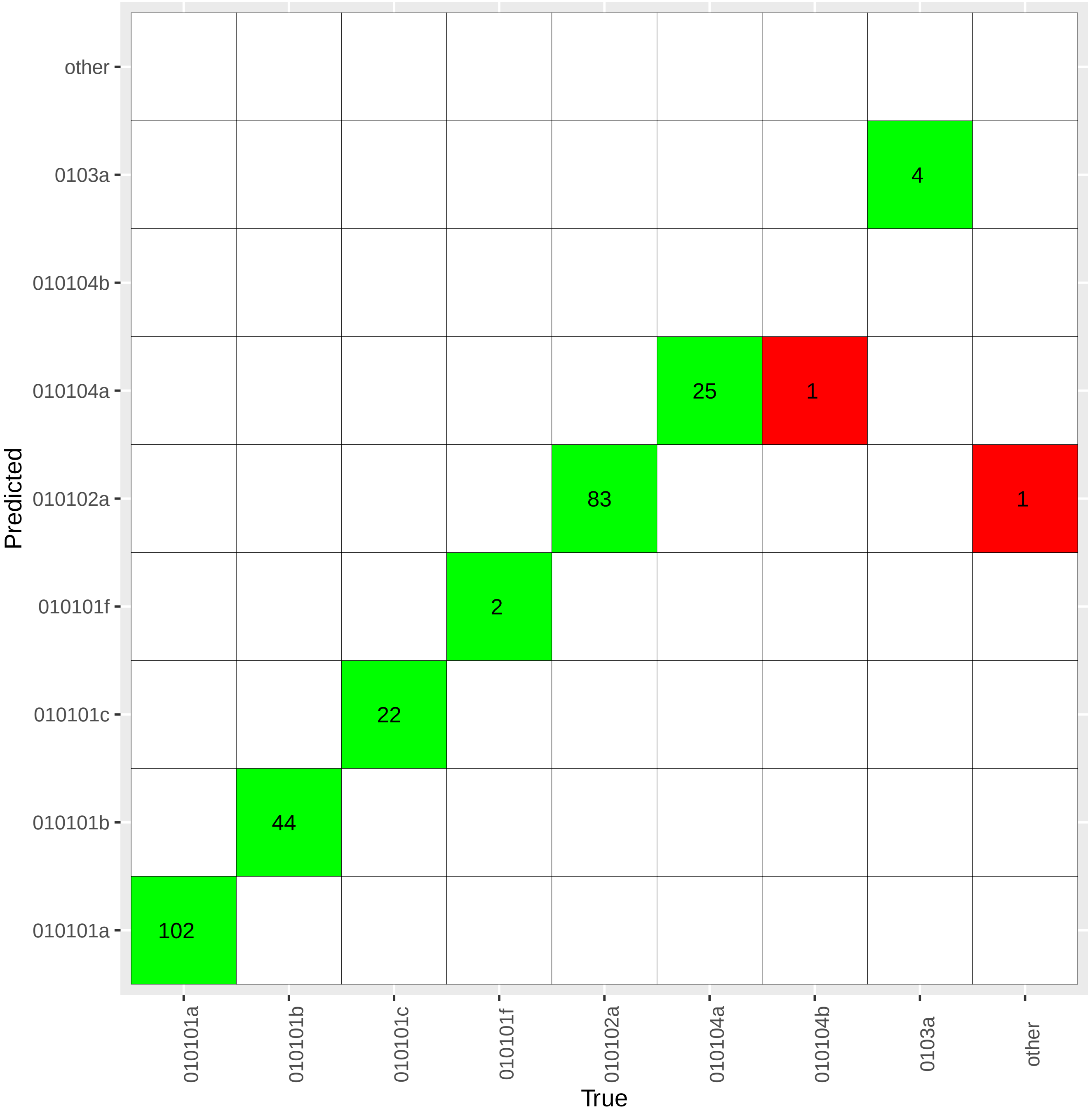

gene = HLA_G
model = i
model limit = NULL
pop = FIN

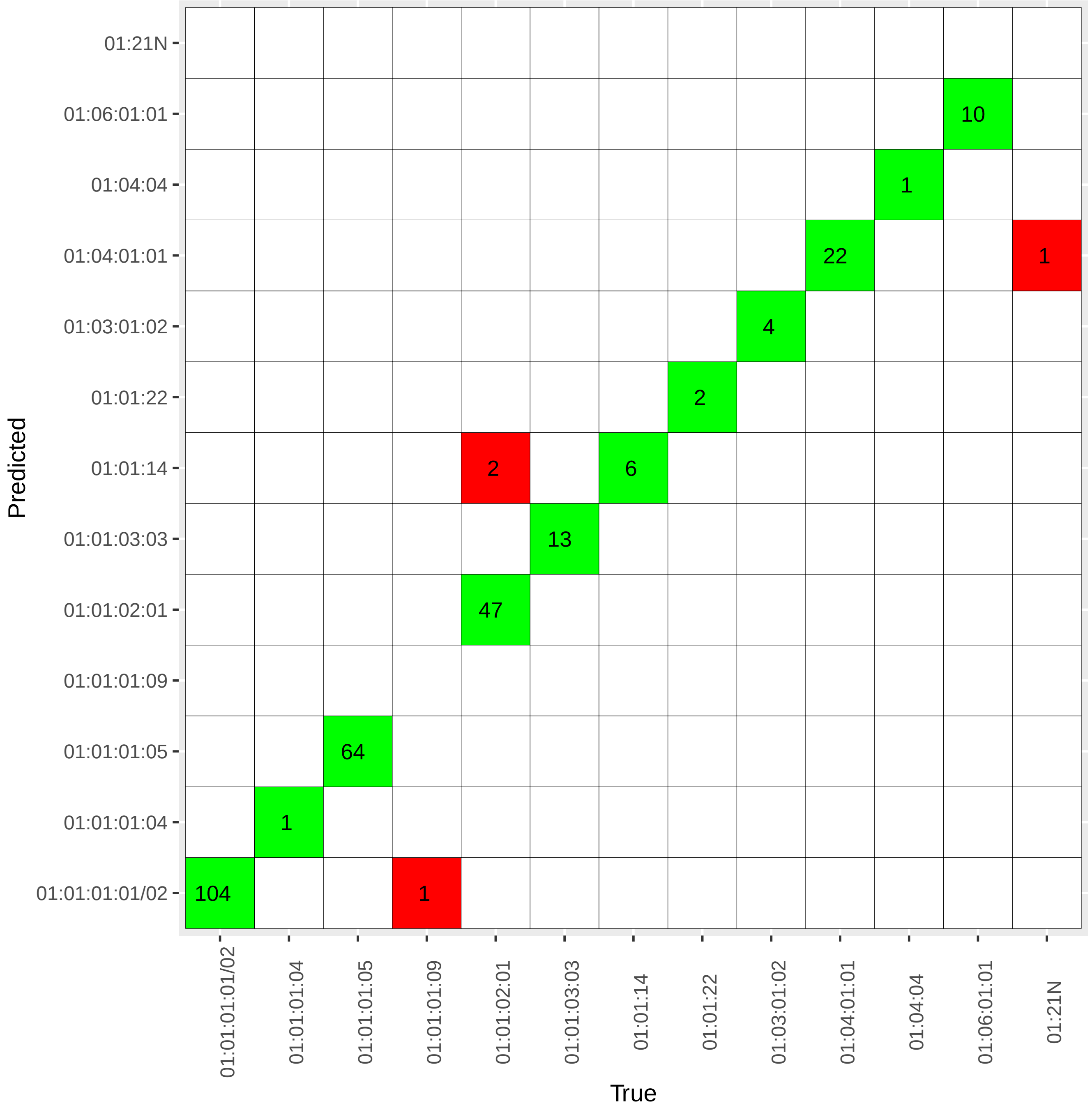

Supplement: S2 Text — (PDF) [file pcbi.1011718.s002.pdf]
