# Supplementary material for: Accurate multi-population imputation of MICA, MICB, HLA-E, HLA-F and HLA-G alleles from genome SNP data
Source: PLoS Comput Biol. 2024 Sep 16;20(9):e1011718. doi: 10.1371/journal.pcbi.1011718 (PMC11426482; doi:10.1371/journal.pcbi.1011718)
Supplement: Attachment — Submitted filename: Response to reviewers.docx [file pcbi.1011718.s004.docx]

Response to Reviewers

We would like to thank the Reviewers for all the valuable comments and suggestions and for the opportunity to revise our submission. The comments have greatly helped us improve our manuscript and make the results more useful for the scientific community. Please find our detailed responses in red below. Line numbers refer to the marked-up version of the revised manuscript.

Sincerely,
Silja Tammi
Corresponding author

Reviewer #1: In this work, Silja Tammi et al. perform a thorough investigation and development of an imputation pipeline for MICA, MICB, HLA-E, HLA-1 F and HLA-G (including UTR polymorphism). The resources used are multiethnic and include the 1000 genomes and Finn Gen. I have only a few comments:

Having an idea of the allele frequency of the predicted alleles is important as imputation can be very accurate if polymorphisms are rare. This is indirectly presented in the figures with the counts, which I particularly appreciated, together with the availability of the data.

They could have further validated their algorithm using the UKB data

There are 2 limitations of this work, one clearly stated the other less so.

Then first limitation pertains to the fact a lot of incomplete coverage sequence had to be eliminated. This could create a bias, and possibly having an effect on rare alleles, but this is acknowledged.

The second limitation has to do with the scope of the study. The title and the abstract, unless read carefully, could be misinterpreted as if the authors were establishing a high accuracy imputation method for these gene alleles usable with whole genome association genotyping arrays. This is not the case, it is an imputation method to be used for exome or whole genome sequencing reads; this also explains the high accuracy as it uses dense SNP within the gene (+/- a few kb). In theory the whole coding information needed to define each allele is there except phasing in some cases where long reads could be needed.

It does not change the fact this is useful especially now that WGS and WES is mainstream and that questionable results such as in Commun Biol. 2023; 6: 1113 are starting to be published (the paper reports high frequencies of unknown alleles in the UKB). I would suggest that the title "Accurate multi-population imputation of HLA-E, HLA-F, HLA-G, MICA and MICB alleles from genome SNP data" be changed into "Accurate multi-population imputation of HLA-E, HLA-F, HLA-G, MICA and MICB alleles from genome SNP data derived from exome or whole genome sequencing".

Also, in the abstract, the sentence "To facilitate studies of the nonclassical and non-HLA genes in large patient and biobank cohorts, we trained imputation models for MICA, MICB, HLA-E, HLA-F and HLA-G alleles on genome SNP array data" could be changed into "To facilitate studies of the nonclassical and non-HLA genes in large patient and biobank cohorts with whole genome and exome sequencing, we trained and show highly accurate imputation models for MICA, MICB, HLA-E, HLA-F and HLA-G alleles base on genome SNP array data".

This limitation and the state of imputation of these gene alleles using whole genome SNP arrays like Affy PMRA or illunina infinium could have been discussed (or attempted since FinnGen has been genotyped with these). My recollection is that only Deep-HLA a deep learning based method developed by Naito and Okada in japan (Nature Communications volume 12, Article number: 1639 (2021)) exists but I am not sure how good it is for these loci. Finally, some of these genes (MICA, etc) have rare duplication and deletions, see Front. Immunol., 14 November 2023, duplication/deletions could be also mentioned as a limitation as I am not sure how these are handled.

We agree with the reviewer’s observation and have accommodated the models also for less SNP-dense data by training additional models for the commonly used genotyping arrays, GSA and PRMA, and estimated their accuracies. The results are now reported in the Results section in the paragraph ‘Overall accuracy of imputation models fitted for GSA and PRMA array SNP content’ (lines 297-320) and in Figure F in the Supporting Information (S1 Text). The models are available in GitHub together with the original SNP-dense models. However, we have opted to keep the manuscript title and the highllighted sentence in their original forms as the models now apply to data with variable SNP density as well. We thank the reviewer for this valuable observation and believe that it improves considerably the usefulness of the work.

Works by Naito et al. 2021 and Hirata et al. 2019 indeed include the non-classical and MIC genes in the Japanese reference panels. However, the accuracies for these genes are not reported and the models are not available for other researchers. This notion has been added to the Introduction section, lines 75-76.

To our knowledge, the MICA duplications and deletions are not included in the allele definitions, and therefore the method is not able to detect them. We believe a separate imputation method would be necessary to detect possible duplications and deletions. We have added this limitation to the Discussion section, lines 381-382.

Reviewer #2: Comments to the Authors:

In this manuscript (Tammi et al.), the authors implemented imputation models for MICA, MICB, HLA-E, HLA-F and HLA-G alleles in HIBAG framework. They used clinical-grade allelic information for Finnish dataset and also WES-based estimated alleles for 1KG dataset. They showed accuracy metrics for each of the models combining both sources. These models are publicly released through github. I think the models for imputing the non-classical genes within MHC would be valuable resource for the community. I have several major comments mostly for the metrics to assess the imputation performance, and for clarity of the description of imputation and validation methods.

1. The ‘accuracy’ metrics that the authors described are known to be upwardly biased for rare alleles by random chance. I would like to see the dosage correlation of the true alleles vs. imputed alleles as another way to assess the imputation performance, especially in the section of using 1KG as a reference model and Finnish data as the target of imputation and comparison with the clinical-grade ‘true’ alleles. It would also be useful to assess the dosage correlation as a function of minor allele frequency, which I believe is the standard of the field.

We thank the reviewer for this suggestion. The plot showing Pearson’s correlation (r) between imputed and true allele dosages versus allele frequency has been added as Figure E in the Supplementary Information (S1 Text) and the figure is referenced in the manuscript lines 217, 252 and 287.

2. In the Result section (main text), I would recommend the authors to describe briefly about the Finnish dataset, including how the alleles were called, before moving onto the ‘Imputation model development’ section for readers to understand the overview of the samples used in the reference. Also, briefly describing the SNPs in the 1KG and Finnish datasets would be helpful.

We agree that adding descriptions of the allele calling in the Finnish dataset and the SNP contents of the references would increase clarity for the readers. The header ‘Allele calling in the 1000 Genomes short-read exome sequencing data’ has been changed to ‘Allele calling’ and a description of the allele calling in the Finnish dataset has been added to lines 86-93. Description of the SNP content in both reference datasets has been added to lines 141-145 and as a column in Table 1.

3. Has the imputation performance been compared between flanking regions 50kb vs. 500kb (default of HIBAG)?

Since the reference data was very SNP-dense, using the HIBAG default flanking region of 500kb would have resulted in over 15000 SNPs in the Finnish reference data and over 30000 SNPs in the 1000G reference data for MICA alone. The phasing of this large number of SNPs would not have been computationally feasible. We chose therefore to limit the flanking region testing to 50kb, since it did not result in better performance of the models compared to 10 kb. However, we have now fitted the models also for the less SNP-dense genotyping arrays, GSA and PMRA, and these models have been trained using the HIBAG default 500 kb flanking region, which resulted in a computationally feasible numbers of approximately 1500 – 2000 SNPs (Figure F in the Supporting Information S1 Text).

4. Considering realistic setting of the MHC imputation, it would be nice to assess the imputation performance using SNPs from commonly used genotyping array (e.g., Illumina MEGA/GSA). Using the SNPs from 1KG, the authors could create ‘mock’ genotyping data for these arrays (target) and assess the performance using Finnish-based model as a reference.

We agree with the reviewer’s observation and have accommodated the models also for less SNP-dense data by training additional models for the commonly used genotyping arrays, GSA and PRMA, and estimated their accuracies. The results are now reported in Results section in the paragraph ‘Overall accuracy of imputation models fitted for GSA and PRMA array SNP content’ (lines 297-320) and in Figure F in the Supporting Information (S1 Text). The models are available in GitHub together with the original SNP-dense models. We thank the reviewer for this valuable observation and believe that it improves considerably the usefulness of the work.

5. As another consideration for realistic setting of the MHC imputation, most of the investigators would be interested in fine-mapping the diseases alleles in the entire MHC region, not limiting to the MICA, MICB, HLA-E, HLA-F and HLA-G alleles. If the entire MHC region is imputed altogether (including classical HLA alleles), we can perform conditional analyses to identify the exhaustive combination of the MHC alleles independently associated with the disease. To this end, the reference models not only for the genes the authors focused on but also for the classical HLA alleles with the same reference samples should be very valuable. Would it be possible for the authors to release the models for the classical HLA alleles, or is it already publicly accessible?

We agree that combining our tool with classical HLA imputation would be most valuable for fine-mapping studies. Currently there are many publicly available imputation references for the classical HLA, both multi-ethnic and ethnic-specific, for example by HIBAG (<https://hibag.s3.amazonaws.com/index.html>) or by our previous work (Ritari et al. 2020). These models can be combined with the models of this work for fine-mapping of the alleles in the whole MHC region. To that end, we recommed that the researcher chooses the optimal classical imputation panel best suited for their needs. The imputation output VCF files can be easily merged into one with e.g. bcftools concat command. We have now added this to the GitHub host page of our tool.
